# Construction of remote dual stereocenters by electrochemical cobalt-catalyzed enantioselective desymmetrization

**DOI:** 10.1038/s41467-026-68437-w

**Published:** 2026-01-15

**Authors:** Yanjun Li, Siyu Liu, Binbin Yuan, Nico Graw, Lutz Ackermann

**Affiliations:** 1 Institut für Organische und Biomolekulare Chemie, Georg-August-Universität Göttingen, Tammannstraße 2, 37077 Göttingen, Germany; 2 Institut für naturwissenschaftliche Bildung, Rheinland-Pfälzische Technische Universität Kaiserslautern-Landau, Fortstraße 7, 76829 Landau in der Pfalz, Germany

**Keywords:** Asymmetric synthesis, Synthetic chemistry methodology

## Abstract

The enantio- and diastereoselective construction of two stereogenic centers represents a highly attractive objective in synthetic chemistry. Extensive asymmetric catalytic methods have been developed for the formation of vicinal stereocenters. In contrast, the simultaneous construction of two constitutionally distinct stereogenic centers at remote positions in a single asymmetric catalytic step remains very scarce, owing to the lack of reliable models for distant stereochemical induction for both chiral entities. Herein, we report on an electrochemical cobalt-catalyzed asymmetric hydroacylation of enynes by a desymmetrization strategy that enables the enantio- and diastereo-selective construction of remote dual stereocenters. This unified catalytic platform exhibits broad substrate generality and affords four distinct classes of chiral products, each incorporating two chiral elements: 1,6-central/C–C axial chirality, 1,6-central/C–O axial chirality, 1,5-central/[2.2]paracyclophane planar chirality, and 1,5-central/ferrocene planar chirality.

## Abstract (web summary)

The simultaneous construction of two constitutionally distinct stereogenic centers at remote positions in a single asymmetric catalytic step remains scarce, owing to the lack of reliable models for distant stereochemical induction for both chiral entities. Herein, the authors report an electrochemical cobalt-catalyzed asymmetric hydroacylation of enynes by a desymmetrization strategy that enables the enantio- and diastereo-selective construction of remote dual stereocenters.

## Introduction

**Fig. 1:**
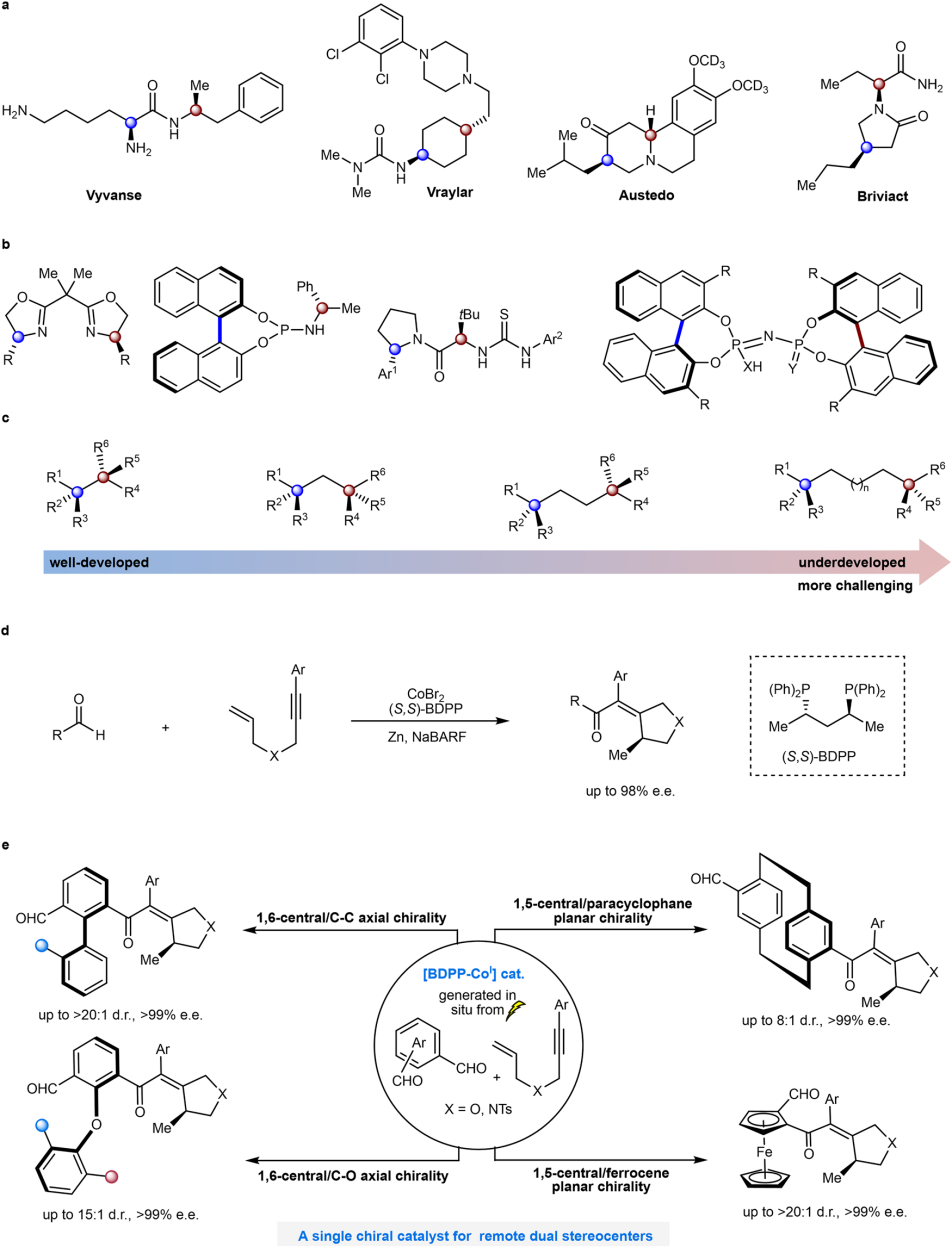
Motivation and strategy for the construction of remote dual stereocenters. **a** Examples of drugs bearing remote dual stereocenters (four of the top 100 drugs by retail sales in 2023). **b** Examples of ligands/catalysts bearing remote dual stereocenters. **c** The difficulty of one-step asymmetric construction of two stereocenters. **d** Lautens’s work: cobalt-catalyzed enantioselective intermolecular hydroacylation of 1,6-enynes. **e** This work: construction of remote dual stereocenters by electrochemical cobalt-catalyzed enantioselective desymmetrization. *d.r.* diastereoselectivity. *e.e.* enantiomeric excess. *NaBArF* sodium tetrakis[3,5-bis(trifluoromethyl)phenyl]borate.

Asymmetric catalysis has achieved significant progress over the past decades. Numerous synthetic approaches have been developed for the enantio- and diastereoselective construction of molecules containing 1,2-diastereogenic centers^1–3^. Remote dual stereocenters are prevalent in chiral ligands/catalysts^4–8^ and chiral drugs^9^, including four of the top 100 drugs by retail sales in 2023 (Fig. 1a, b). However, catalytic construction of two nonadjacent and constitutionally distinct stereocenters has long remained a formidable challenge, particularly for 1,n-nonadjacent (n ≥ 5) stereocenters^10^. Furthermore, the larger the distance between two stereocenters, the more difficult it becomes to achieve asymmetric induction (Fig. 1c)^11^. The merger of synergistic catalysis that employs two distinct chiral catalysts to control double chiral induction at two 1,n-nonadjacent (n ≥ 5) stereocenters has only recently been demonstrated^10–13^. However, synergistic catalytic systems frequently face challenges such as incompatibility between multiple catalysts and substrates, as well as ensuring the correct sequence of each process, which greatly limits both the range of possible reactions and the diversity of products. The use of a single catalyst to control chiral induction at remote dual stereocenters bearing different chiral elements has thus far remained elusive^14^.

Achieving substrate generality remains a formidable challenge in asymmetric catalysis^15–17^, particularly in the synthesis of products containing different chiral elements. Most asymmetric catalytic systems are effective only for a limited range of substrates and are typically confined to establishing a single type of chirality. Usually, accessing different chiral elements by asymmetric catalysis requires distinct synthetic strategies and substantial effort to optimize chiral catalysts and reaction conditions. Although catalytic strategies for the synthesis of compounds with two types of chiral elements^18^, such as central-axial^19–23^, central-planar^24^, central-helical^25^, axial-planar^26,27^, and axial-helical^21^, have been devised individually, it remains difficult to access a broader variety of chiral elements in a unified catalytic system because of their distinct topologies.

Transition-metal-catalyzed enantioselective hydroacylation of alkenes has emerged as a powerful strategy for the synthesis of chiral ketones through the asymmetric addition of aldehyde C–H bonds across alkenes^28–34^. Pioneering studies on cobalt-catalyzed enantioselective intramolecular hydroacylation of alkenes were achieved by Yoshikai^30,31^. Subsequently, Lautens disclosed a cobalt-catalyzed intermolecular hydroacylation of 1,6-enynes with aldehydes (Fig. 1d)^34^. Enantioselective desymmetrization transforms prochiral or *meso* substrates into chiral products by generating one or more stereogenic centers, providing efficient access to structurally complex molecules from readily available starting materials. This strategy has been widely applied in various transformations, including aldol reactions, epoxidations, reductions, cross-coupling reactions, and ring-closing metathesis^35–39^.

More recently, asymmetric electrochemical transformations have attracted considerable attention as a versatile platform for the construction of chiral molecules^40,41^. Within our continuous interest in electrochemical enantioselective C–H functionalization^22,42–44^, we have now identified the electrochemical cobalt-catalyzed asymmetric hydroacylation of enynes for the construction of remote dual stereocenters by a desymmetrization strategy, on which we report herein (Fig. 1e). This unified catalytic platform exhibits substrate generality and affords four distinct classes of chiral products, each containing two chiral elements. These include 1,6-central/C–C axial chirality, 1,6-central/C–O axial chirality, 1,5-central/[2.2]paracyclophane planar chirality, and 1,5-central/ferrocene planar chirality.

## Results

**Fig. 2:**
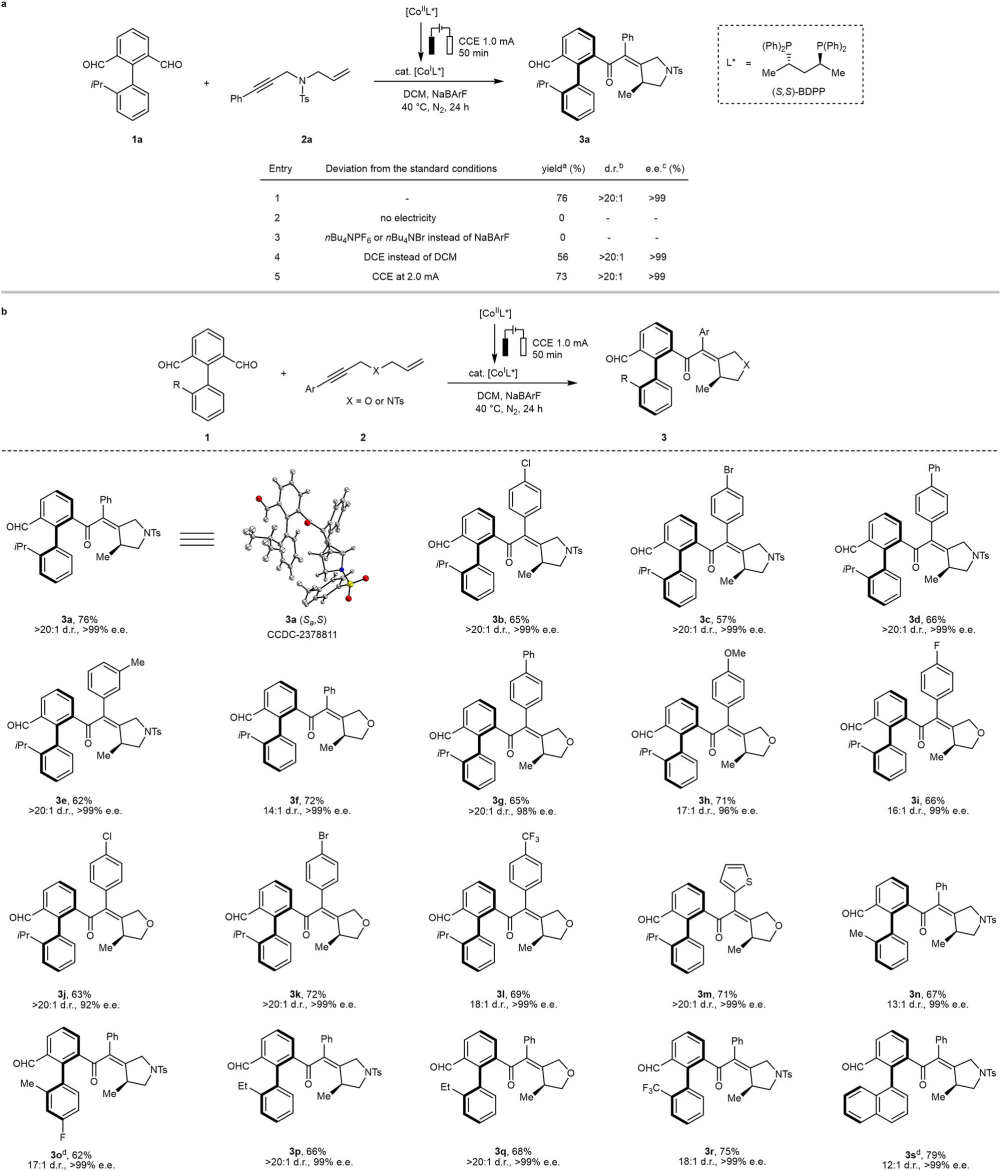
Reaction development and establishing 1,6-central/C−C axial chirality. **a** Reaction development. Standard reaction conditions: [(*S*,*S*)-BDPP]CoBr_2_ (0.010 mmol), NaBArF (0.050 mmol), DCM (2.0 mL), undivided cell, constant current at 1.0 mA, zinc plate anode and nickel foam cathode, 40 °C, 50 min; then **1a** (0.11 mmol), **2a** (0.10 mmol), 40 °C, 24 h. ^a^Isolated yield. ^b^d.r. is determined by ^1^H-NMR. ^c^e.e. is determined by chiral HPLC analysis. ^d^At room temperature (RT) for 48 h. **b** The construction of 1,6-central/C−C axial chirality. *DCM* dichloromethane, *DCE* 1,2-dichloroethane, *CCE* constant current electrolysis.

Our investigation was initiated with the reaction of biaryl dialdehyde **1a** with 1,6-enyne **2a**. After intensive optimization studies (see details in Supplementary Table 1), the optimal conditions were identified as follows: a catalytic amount of [(*S*,*S*)-BDPP]CoBr_2_ was electrochemically reduced with a constant current of 1.0 mA in an undivided cell at 40 °C for 50 min; then substrates **1a** and **2a** were added, and the reaction mixture was stirred at 40 °C. The standard conditions afforded the desired product **3a** in 76% yield with > 20:1 d.r. and > 99% e.e. (Fig. 2a, entry 1). The structure of product **3a** was unambiguously confirmed by X-ray diffraction analysis. The atropostability of the C–C axis in product **3a** was calculated via density functional theory, showing a rotational barrier of 32.7 kcal mol^−1^, indicative of a half-life *t*_1/2_ of 112.3 years (see Supplementary Information section 7 for details). Control experiments revealed that electricity was essential for the reaction (Fig. 2a, entry 2). When *n*Bu_4_NPF_6_ or *n*Bu_4_NBr were used as the electrolytes, no desired product was generated (Fig. 2a, entry 3). The choice of solvent was key to success, as evidenced by the low yield (0–56%) obtained when using other solvents (Fig. 2a, entry 4; Supplementary Table 1). At a constant current of 2.0 mA, a slightly decreased yield was noted (Fig. 2a, entry 5). Other chiral phosphine ligands were tested but failed to provide the desired product (see details in Supplementary Table 1).

With the optimized conditions in hand, the substrate robustness of the electrocatalysis was investigated (Fig. 2b). First, various substituents on the phenyl ring of 1,6-enynes **2** were evaluated. As a result, both electron-donating and electron-withdrawing groups were found to be well-tolerated, delivering the corresponding products with 1,6-central/C−C axial chirality (**3a** − **3 l**) in good yields (57 − 76%) with excellent enantioselectivities (92% to > 99% e.e.) and good to excellent diastereoselectivities (14:1 to > 20:1 d.r.). Second, a substrate bearing a thiophene motif was successfully transformed into the corresponding product **3 m**. Third, we probed the scope of different substituted biphenyl bialdehydes. The replacement of the isopropyl group at the 2-position of the benzene ring with methyl, ethyl, or trifluoromethyl substituent was feasible, giving the desired products **3n**−**3r** in good yields with excellent enantioselectivities (up to > 99% e.e.) and good to excellent diastereoselectivities (13:1 to > 20:1 d.r.). The reaction of naphthyl-substituted dialdehyde with **2a** efficiently yielded product **3 s** with high enantio- and diastereoselectivities.

**Fig. 3:**
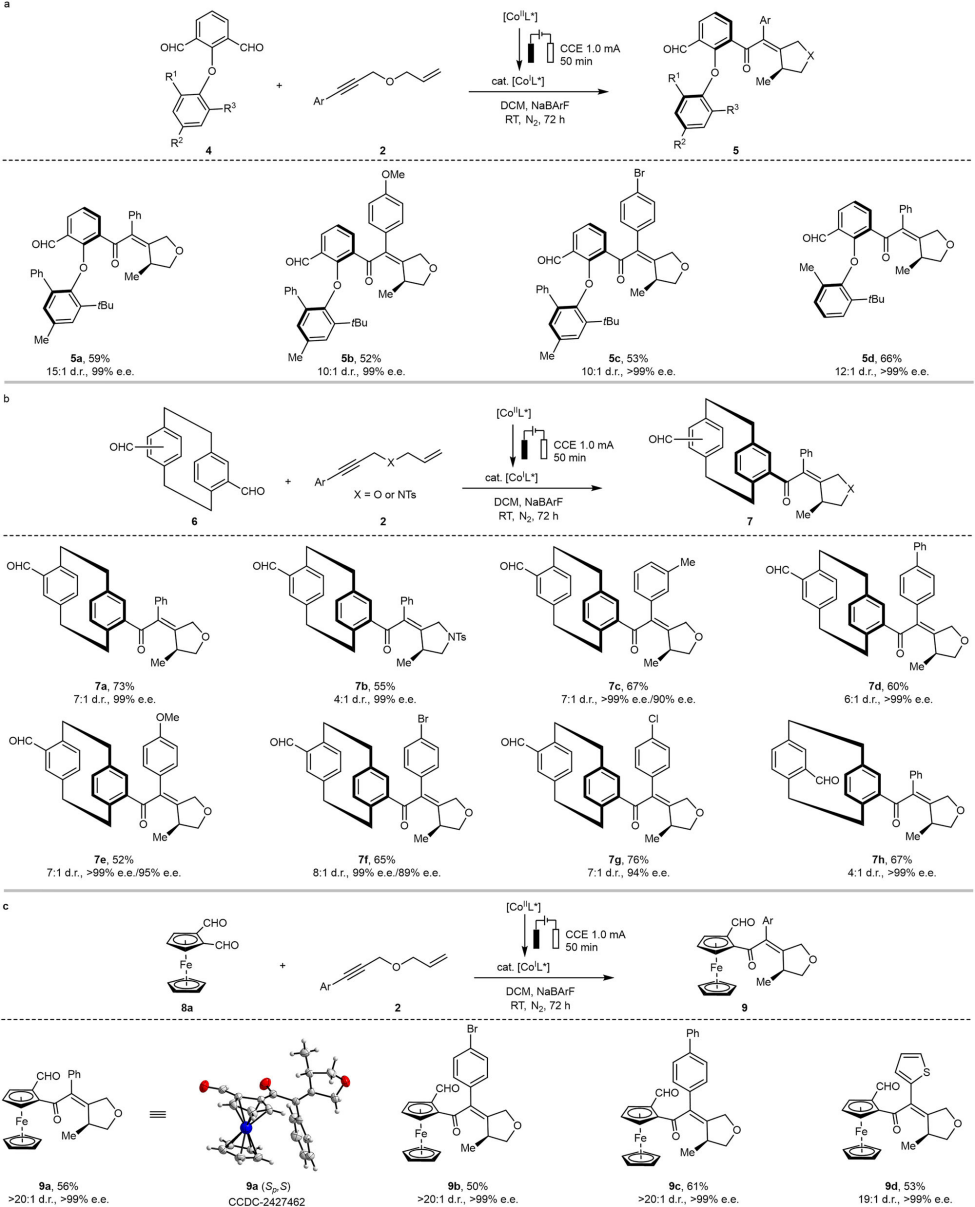
Construction of 1,6-central/C–O axial chirality and 1,5-central/planar chirality. **a** The construction of 1,6-central/C–O axial chirality. **b** The construction of 1,5-central/[2.2]paracyclophane planar chirality. **c** The construction of 1,5-central/ferrocene planar chirality.

Encouraged by the results on C–C atropisomers, we were keen to expand this electrocatalytic system to the synthesis of more challenging atropisomeric diaryl ethers. Atropisomeric diaryl ethers with restricted C−O bond rotation are found in biological molecules and serve as promising frameworks for designing chiral ligands^45^. However, limited reported methods for the synthesis of chiral diaryl ethers have significantly hindered progress in the field when compared to well-studied axially chiral biaryls^45–49^. In this context, we envisioned that if a C–O axis-containing prochiral substrate is subjected to our strategy, the [(*S*,*S*)-BDPP]-cobalt catalyst has the propensity to modulate the C–O axis steric environment and spatial distribution enabling an efficient chirality control. This can be attributed to the structural characteristics of (*S*,*S*)-BDPP, which features a flexible chiral secondary carbon backbone with 2,4-bis(diphenylphosphino) substituents, allowing the catalyst to adopt a suitable P–Co–P coordination angle. Gratifyingly, under the standard reaction conditions, the diaryl ethers substrate with 1,6-enynes gave products **5a**−**5 d** in 52 − 66% yield with excellent enantioselectivities (up to > 99% e.e.) and good to excellent diastereoselectivities (10:1 − 15:1 d.r.) (Fig. 3a).

The above results inspired us to explore the wider substrate generality. Planar chiral [2.2]paracyclophane derivatives are fascinating chiral molecules with significant applications in asymmetric catalysis and materials science^50,51^. However, current synthetic methods to obtain enantioenriched [2.2]paracyclophanes remain largely reliant on chiral chromatography or chemical resolution, with only a few examples of asymmetric catalysis reported^51–54^. Asymmetric transformations of [2.2]paracyclophane derivatives are particularly challenging due to their intrinsic structural distortions, rigidity, and pronounced steric hindrance. As such, these compounds shine as challenging molecules, and therefore excellent target motifs to assess the robustness and versatility of our framework. Notably, the reaction of paracyclophane dicarbaldehydes **6** with 1,6-enynes **2** afforded the anticipated products (**7a**–**7 h**) featuring 1,5-central/planar chirality in 44–60% yield, with excellent enantioselectivities (up to > 99% e.e.) and moderate to good diastereoselectivities (4:1–8:1 d.r.) (Fig. 3b). It is noteworthy that some of the diastereoisomeric products (**7c,** **7e,** **7f**) can be easily separated by silica gel chromatography, and the minor diastereomers were obtained with good enantiomeric excess (89%–95% e.e.). The application of our approach was next extended to another typical planar chiral structure−chiral ferrocene. The ferrocene dicarbaldehydes with 1,6-enynes gave desired products **9a**–**9d** with 1,5-central/planar chirality in 50–61% yield with excellent enantioselectivities ( > 99% e.e.) and diastereoselectivities (up to > 20:1 d.r.) (Fig. 3c). The structure of product **9a** was confirmed by single-crystal X-ray diffraction. The introduction of remote dual stereocenters into [2.2]paracyclophane or ferrocene could further extend their potential applications, for instance in the design of chiral ligands.

**Fig. 4:**
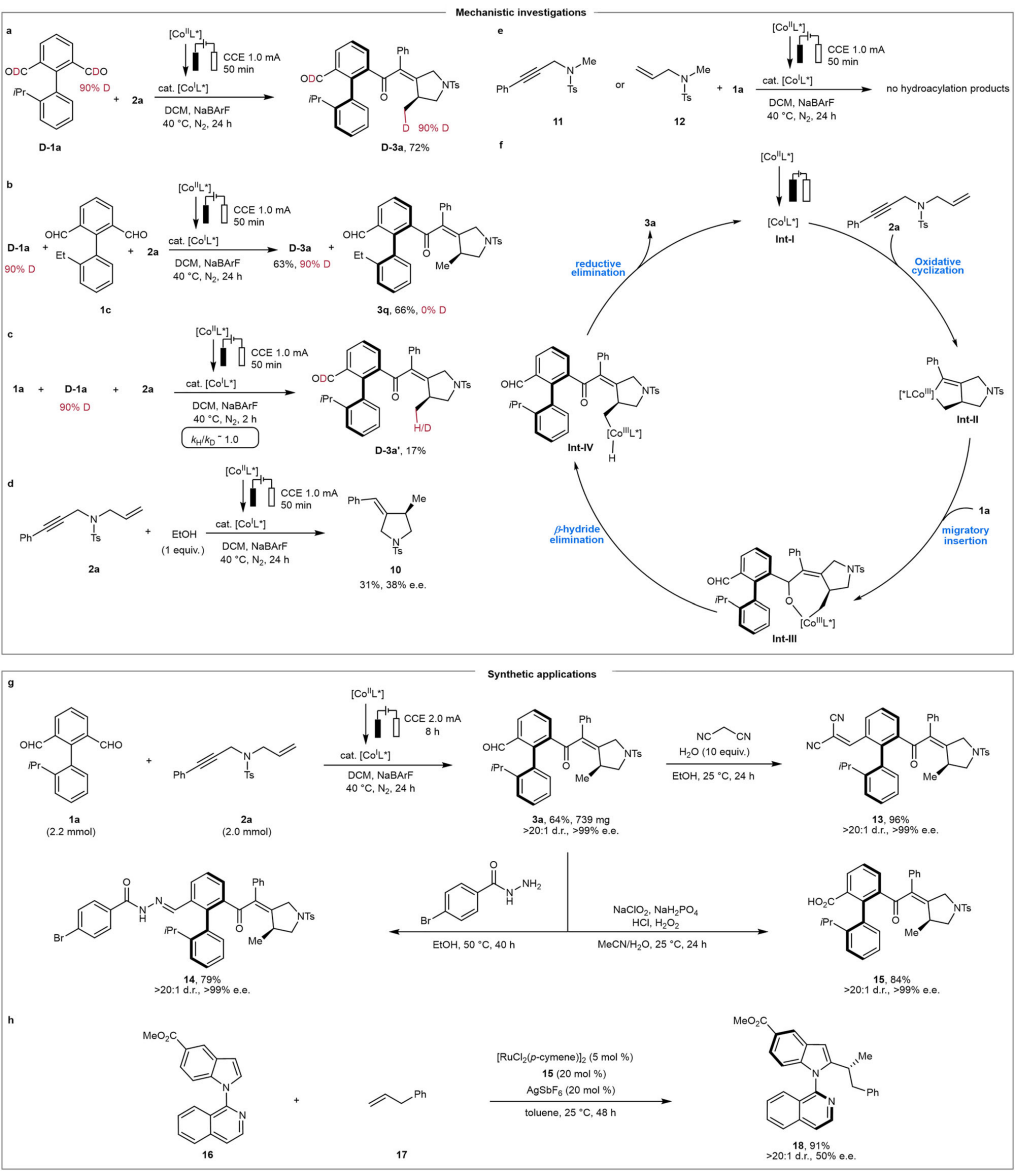
Mechanistic investigations and synthetic applications. **a** Deuterium labeling experiments. **b** Deuterium crossover experiments. **c** KIE experiments. **d** Control experiments for the reaction of enyne with ethanol. **e** Control experiments with alkyne or alkene. **f** Proposed mechanism. **g** Gram-scale synthesis and follow-up transformations of product **3a**. **h** Chiral carboxylic acid **15** was evaluated as a chiral ligand in the ruthenium-catalyzed asymmetric C−H activation reaction.

In order to shed light into the catalyst’s mode of action, we carried out several mechanistic studies (Fig. 4). When deuterated dialdehyde **D-1a** (90% D) was subjected to the standard reaction conditions (Fig. 4a), the product **D-3a** was obtained with 90% deuterium incorporation. Deuterium crossover experiments with substrates **D-1a** and **1c** revealed that deuterium was solely incorporated into **D-3a** (Fig. 4b). The absence of deuterium crossover rules out a ligand-to-ligand hydrogen transfer pathway. A KIE of *k*_H_/*k*_D_ ≈ 1.0 was suggestive of the C−H cleavage not being involved in the turnover-limiting step (Fig. 4c). The results of deuterium incorporation and deuterium crossover experiments were consistent with Lautens’s work^34^. The reaction of enyne **2a** in the presence of ethanol gave product **10**, indicating that the reaction proceeds via a cobaltacycle species (Fig. 4d)^55^. Substrates **11** and **12**, without either the alkyne or alkene moiety of the enyne, gave no hydroacylation products under the reaction conditions, being indicative of the oxidative cyclization of the enyne (Fig. 4e).

Based on these experimental findings, a proposed catalytic cycle is depicted in Fig. 4f. The reaction initiates with the formation of the low-valent chiral cobalt(I) active catalyst **Int-I** via cathodic reduction of [(*S*,*S*)-BDPP]CoBr_2_^56^. Cyclic voltammetry assigned the cobalt(II)/cobalt(I) redox couple a potential of –0.56 V (see details in Supplementary Fig. 5), indicating that the low-valent chiral cobalt species is readily generated under electroreductive conditions, thereby supporting the proposed catalyst activation pathway. The latter upon coordination of the enyne **2a** undergoes enantioselective oxidative cyclization to generate the cobaltacycle **Int-II** with the construction of the first stereogenic center. Then, migratory insertion of the dialdehyde **1a** takes place, which enables the formation of the cobaltacycle **Int-Ⅲ** leading to the formation of the second stereogenic center. Thereafter, *β*-hydride elimination followed by reductive elimination delivers the desired product **3a** as well as the regeneration of the catalyst. An alternative pathway contemplating oxidative addition is considered and depicted in Supplementary Fig. 6.

Thereafter, diverse synthetic transformations were performed to demonstrate the synthetic practicality of our methodology (Fig. 4g). Notably, a gram-scale electroreduction provided the product **3a** in good yield with excellent enantioselectivity ( > 99% e.e.) and diastereoselectivity ( > 20:1 d.r.). The reaction of **3a** with cyanoacetonitrile gave compound **13** in 96% yield. Condensation of the formyl group with benzohydrazide afforded the corresponding imine **14** in 79% yield. Oxidation of **3a** afforded chiral carboxylic acid **15** in 84% yield. To our delight, all these transformations proceeded with the retention of both enantioselectivity as well as diastereoselectivity. Furthermore, chiral carboxylic acid **15** was further examined as a chiral ligand in the ruthenium-catalyzed asymmetric C−H activation, furnishing the product **18** with both central and axial chirality in moderate enantioselectivity and excellent diastereoselectivity (Fig. 4h).

## Discussion

A uniquely robust and versatile strategy for remote dual stereocenters featuring diverse elements was viable through electroreductive cobalt-catalyzed enantioselective desymmetrization. Through the use of a single catalytic system and without any further optimization, our strategy enables the generation of various stereogenic elements, including central chirality, C–C/C–O axial chirality, [2.2]paracyclophane planar chirality, and ferrocene planar chirality. The mechanism demonstrates that the catalyst directly governs two stereoselectivities in different elementary steps in the catalytic cycle, thus enabling precise control over 1,5/1,6-double chiral inductions.

## Methods

### General procedure for electrochemical cobalt-catalyzed enantioselective desymmetrization

The electrocatalysis was carried out in an undivided cell, with a zinc electrode (10 mm × 25 mm × 0.25 mm) and a nickel foam electrode (10 mm × 25 mm × 1 mm). In the glovebox, [(*S*,*S*)-BDPP]CoBr_2_ (6.6 mg, 0.010 mmol), NaBArF (44.3 mg, 0.050 mmol), and dry DCM (2 mL) were placed in a 10 mL cell. Electrocatalysis was performed at 40 °C with a constant current of 1.0 mA maintained for 50 min. Then, the nickel foam cathode and the zinc anode were taken out, bialdehyde (0.11 mmol) and 1,6-enyne (0.10 mmol) were added, and the reaction mixture was stirred at 40 °C or room temperature under a nitrogen atmosphere for 24–72 h. The resulting mixture was purified by column chromatography on silica gel to afford the desired product.

## Supplementary information

Supplementary Information

Transparent Peer Review file

## Source data

Source Data

## Data Availability

The crystallographic data related to the structures reported herein have been stored at the Cambridge Crystallographic Data Centre (CCDC), with deposition numbers CCDC 2378811 (**3a**) and 2427462 (**9a**). These data can be obtained free of charge from The Cambridge Crystallographic Data Centre via www.ccdc.cam.ac.uk/data_request/cif. Source data are provided with this paper. All other data supporting the findings of this study, including detailed experimental procedures and compound characterization, are available within the paper and its Supplementary Information files or from the corresponding author on request. Source data are provided with this paper.
