# Supplementary material for: Construction of remote dual stereocenters by electrochemical cobalt-catalyzed enantioselective desymmetrization
Source: Nat Commun. 2026 Jan 15;17:743. doi: 10.1038/s41467-026-68437-w (PMC12820297; doi:10.1038/s41467-026-68437-w)
Supplement: Supplementary file 1 — Supplementary Information [file 41467_2026_68437_MOESM1_ESM.pdf]

Supplementary Information

Construction of remote dual stereocenters by electrochemical cobalt-catalyzed enantioselective desymmetrization

Yanjun Li[1,†], Siyu Liu[1,†], Binbin Yuan[1], Nico Graw[2], Lutz Ackermann[1*]

[1]Institut für Organische und Biomolekulare Chemie, Georg-August-Universität Göttingen, Tammannstraße 2, 37077 Göttingen, Germany.

[2]Institut für naturwissenschaftliche Bildung, Rheinland-Pfälzische Technische Universität Kaiserslautern-Landau, Fortstraße 7, 76829 Landau in der Pfalz, Germany.

[†]These authors contributed equally to this work.

*Corresponding author. E-mail: lutz.ackermann@chemie.uni-goettingen.de

Table of Contents

Supplementary Section 1. General Remarks 3

Supplementary Section 2. Optimization of the Reaction Conditions 4

Supplementary Section 3. Substrate Scope 6

Supplementary Section 4. X-Ray Analysis 65

Supplementary Section 5. Key Mechanistic Findings 93

Supplementary Section 6. Synthetic applications 99

Supplementary Section 7. DFT Calculations on the Rotation Barrier 106

Supplementary Section 8. NMR Spectra 108

Supplementary Section 9. Supplementary References 158

Supplementary Section 1. General Remarks

An atmosphere of N_2, pre-dried glassware, and glovebox techniques were employed for reactions involving air- or moisture-sensitive compounds. Ligands and catalysts were purchased from Sigma-Aldrich, Alfa Aesar, and TCI Deutschland. Substrates biaryl dialdehyde[1–4] and 1,6-enyne[5] were synthesized according to previously described methods. Solvents were redistilled under nitrogen and stored over molecular sieves. Zinc electrodes (10 mm × 25 mm × 0.25 mm, 99.9%) and nickel foam electrodes (10 mm × 25 mm × 1 mm, 99.9%) were obtained from ChemPur® Karlsruhe, Germany, and connected using stainless steel adapters. Electrocatalysis was conducted using a Metrohm MULTI AUTOLAB M204 potentiostat or ROHDE & SCHWARZ HMP4040 Potentiostat in two electrode constant current mode. If not otherwise noted, yields refer to isolated compounds, estimated to be >95% pure by NMR. TLC: Macherey-Nagel, TLC plates Alugram®Sil G/UV254. Detection under UV light at 254 nm. Chromatography: Separations were carried out on Merck Silica 60 (0.040–0.063 mm, 70–230 mesh ASTM). All IR spectra were recorded on a BRUKER ALPHA-P spectrometer. ESI-MS: Finnigan LCQ. High resolution mass spectrometry (HRMS): APEX IV 7T FTICR, Bruker Daltonic. HPLC chromatograms were recorded on an Agilent 1290 Infinity using CHIRALPAK® IA-3, AD-3, IC-3, ID-3, IE-3, and IF-3 columns (3.0 µm particle size; Ø: 4.6 mm and 250 mm length). Optical rotations were measured with Perkin Elmer 343 polarimeter at the stated temperature under a Na/Hg lamp, λ = 589 nm (c in g/100 ml). ^{1}H, ^{13}C, and ^{19}F-NMR-spectra were recorded at 300 (^{1}H), 400 (^{1}H), 75, 101 [^{13}C, APT (Attached Proton Test)], and 282 (^{19}F) MHz respectively, on Varian Bruker Avance III 400, Bruker Avance III HD 400. If not otherwise specified, chemical shifts (δ) are given in ppm.

Supplementary Section 2. Optimization of the Reaction Conditions

The synthesis of Co(II) complexes: in the glove box, anhydrous $CoBr_2$ (1.0 equiv., 0.020 mmol) was dissolved in dry THF. In another flask, the ligand (1.05 equiv., 0.021 mmol) was dissolved in dry THF and added slowly to the stirring $CoBr_2$ solution. After the addition, the resultant mixture was stirred for 18–24 h under a nitrogen atmosphere, after which the solvent was removed, and the solid was dried under high vacuum for 12–18 h to afford the desired complex L^*CoBr_2.

General procedure for catalytic reactions: The electrocatalysis was carried out in an undivided cell, with a zinc electrode (10 mm × 25 mm × 0.25 mm) and a nickel foam electrode (10 mm × 25 mm × 1 mm). In the glovebox, L^*CoBr_2 (0.010 mmol), NaBARF (44.3 mg, 0.050 mmol), and dry DCM (2 mL) were placed in a 10 mL cell. Electrocatalysis was performed at 40 °C with a constant current of 1.0 mA maintained for 50 min. Then, the nickel foam cathode and the zinc anode were taken out, **1a** (0.11 mmol) and **2a** (0.10 mmol) were added, and the reaction mixture was stirred at 40 °C under a nitrogen atmosphere for 24 h. The resulting mixture was purified by column chromatography on silica gel to afford the desired product **3a**. The d.r. values were determined by ^{1}H-NMR. The e.e. value was determined by chiral HPLC analysis.

Supplementary Table 1. Optimization of the reaction conditions.

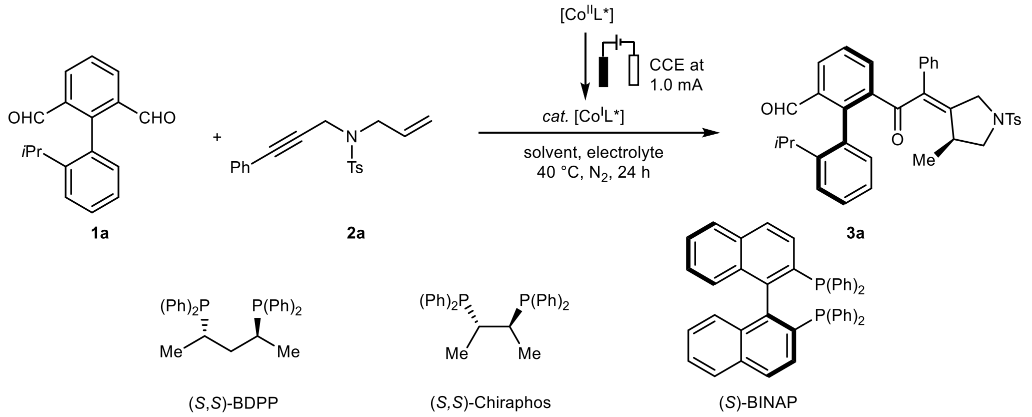

entry	ligand	electrolyte	solvent	yield (%)	d.r.	e.e.
1	(*S*,*S*)-BDPP	NaBArF	DCM	76	>20:1	>99
2	(*S*,*S*)-Chiraphos	NaBArF	DCM	0	---	---
3	(*S*)-BINAP	NaBArF	DCM	0	---	---
4	(*S*,*S*)-BDPP	*n*Bu_4NBr	DCM	0	---	---
5	(*S*,*S*)-BDPP	*n*Bu_4NPF_6	DCM	0	---	---
6	(*S*,*S*)-BDPP	NaBArF	DCE	56	>20:1	>99
7	(*S*,*S*)-BDPP	NaBArF	MeCN	0	---	---
8	(*S*,*S*)-BDPP	NaBArF	DMF	0	---	---
9	(*S*,*S*)-BDPP	NaBArF	MeOH	0	---	---
10[a]	(*S*,*S*)-BDPP	NaBArF	DCM	73	>20:1	>99
11[b]	(*S*,*S*)-BDPP	NaBArF	DCM	0	---	---

NaBArF = sodium tetrakis[3,5-bis(trifluoromethyl)phenyl]borate, DCM = dichloromethane, DCE = 1,2-dichloroethane, CCE = constant current electrolysis. [a]CCE at 2.0 mA. [b]Without current.

Reaction Setup

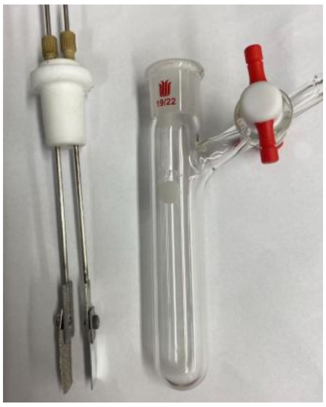

Supplementary Figure 1. Reaction setup.

Supplementary Section 3. Substrate Scope

General Procedure: The electrocatalysis was carried out in an undivided cell, with a zinc electrode (10 mm × 25 mm × 0.25 mm) and a nickel foam electrode (10 mm × 25 mm × 1 mm). In the glovebox, [(*S*,*S*)-BDPP]$CoBr_2$ (6.6 mg, 0.010 mmol), NaBArF (44.3 mg, 0.050 mmol), and dry DCM (2 mL) were placed in a 10 mL cell. Electrocatalysis was performed at 40 °C with a constant current of 1.0 mA maintained for 50 min. Then, the nickel foam cathode and the zinc anode were taken out, bialdehyde (0.11 mmol) and 1,6-enyne (0.10 mmol) were added, and the reaction mixture was stirred at 40 °C or room temperature under a nitrogen atmosphere for 24–72 h. The resulting mixture was purified by column chromatography on silica gel to afford the desired product.

The construction of 1,6-central/C–C axial chirality

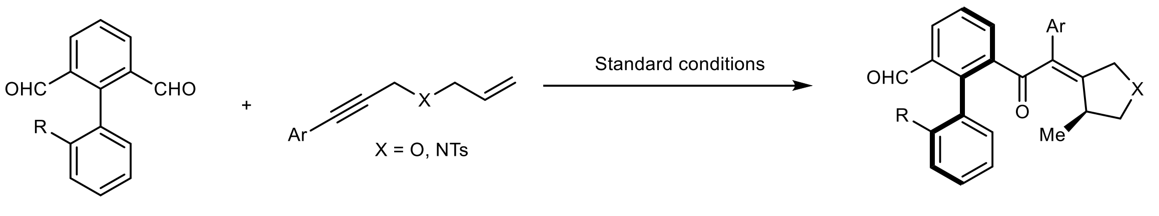

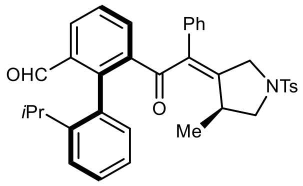

3a

(*S*)-2'-isopropyl-6-((*E*)-2-((*S*)-4-methyl-1-tosylpyrrolidin-3-ylidene)-2-phenylacetyl)-[1,1'-biphenyl]-2-carbaldehyde (3a)

The general procedure was followed using bialdehyde **1a** (0.11 mmol, 27.8 mg) and 1,6-enyne **2a** (0.10 mmol, 32.5 mg) at 40 °C for 24 h. Purification by column chromatography on silica gel (DCM) yielded **3a** (43.9 mg, 76%, >20:1 d.r., >99% e.e.) as a white solid.

^{1}H-NMR (300 MHz, $CDCl_3$) δ 9.46 (d, *J* = 0.7 Hz, 1H), 7.90 (dd, *J* = 7.8, 1.4 Hz, 1H), 7.51 (td, *J* = 7.4, 7.0, 1.6 Hz, 3H), 7.33 – 7.25 (m, 3H), 7.24 – 7.15 (m, 5H), 6.95 (td, *J* = 7.2, 1.9 Hz, 1H), 6.88 – 6.83 (m, 2H), 6.69 (dd, *J* = 7.6, 1.3 Hz, 1H), 3.97 (dd, *J* = 16.3, 1.5 Hz, 1H), 3.37 (d, *J* = 16.3 Hz, 1H), 3.06 (dd, *J* = 8.8, 1.8 Hz, 1H), 3.01 – 2.88 (m, 2H), 2.53 – 2.43 (m, 1H), 2.36 (s, 3H), 1.13 (d, *J* = 6.7 Hz, 3H), 1.03 (d, *J* = 6.7 Hz, 3H), 0.87 (d, *J* = 6.9 Hz, 3H).

^{13}C-NMR (75 MHz, CDCl$_3$) δ 195.6 (C_q), 191.6 (CH), 150.8 (C_q), 148.5 (C_q), 143.9 (C_q), 139.2 (C_q), 135.9 (C_q), 135.2 (C_q), 134.9 (C_q), 133.8 (CH), 133.5 (C_q), 132.2 (C_q), 129.7 (CH), 129.1 (CH), 129.0 (CH), 128.8 (CH), 128.6 (CH), 128.5 (CH), 128.2 (CH), 127.8 (CH), 127.4 (CH), 125.5 (CH), 124.8 (CH), 54.9 (CH$_2$), 51.0 (CH$_2$), 36.1 (CH), 30.8 (CH), 24.2 (CH$_3$), 22.9 (CH$_3$), 21.6 (CH$_3$), 19.7 (CH$_3$).

IR (ATR): 1693, 1676, 1570, 1347, 1238, 1160, 1090, 735, 664 cm^{-1}.

HR-MS (ESI): *m*/*z* calcd. for $[C_{36}H_{35}NO_4S + Na]^+$ 600.2179, found 600.2190.

$[\alpha_D^{20}]$ = +69.0 (c = 1.0, DCM).

HPLC separation (Chiralpak® IA-3, *n*-hexane/*i*-PrOH 95:5, 1.0 mL/min, detection at 273 nm): t_r (major) = 21.9 min, t_r (minor) = 23.7 min, >99% e.e.

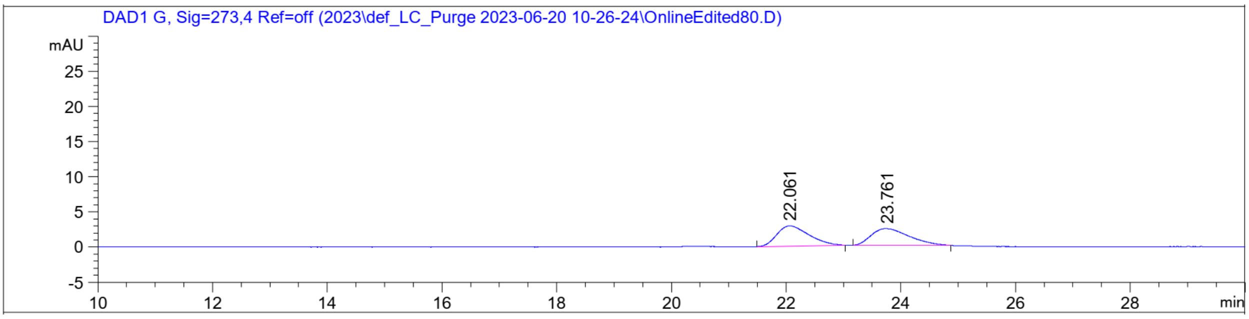

Peak #	RetTime [min]	Type	Width [min]	Area [mAU*s]	Height [mAU]	Area %
1	22.060	BB	0.4655	365.88458	9.22996	50.5692
2	23.742	BB	0.5474	357.64819	7.66510	49.4308

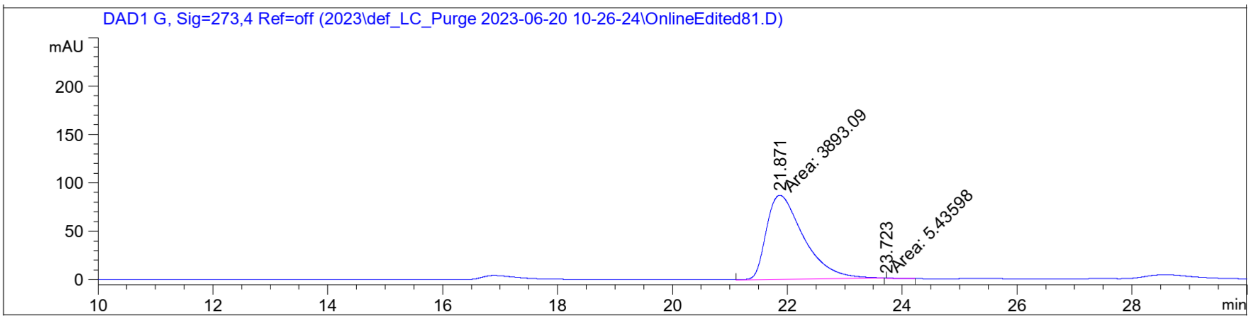

Peak #	RetTime [min]	Type	Width [min]	Area [mAU*s]	Height [mAU]	Area %
1	21.871	MP	0.7483	3893.08960	86.71185	99.8606
2	23.723	MM	0.1620	5.43598	3.97845e-1	0.1394

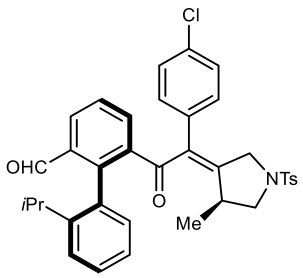

3b

(*S*)-6-((*E*)-2-(4-chlorophenyl)-2-((*S*)-4-methyl-1-tosylpyrrolidin-3-ylidene)acetyl)-2'-isopropyl-[1,1'-biphenyl]-2-carbaldehyde (3b)

The general procedure was followed using bialdehyde **1a** (0.11 mmol, 27.8 mg) and 1,6-enyne **2b** (0.10 mmol, 36.0 mg) at 40 °C for 24 h. Purification by column chromatography on silica gel (DCM) yielded **3b** (39.8 mg, 65%, >20:1 d.r., >99% e.e.) as a white solid.

^{1}H-NMR (400 MHz, CDCl$_3$) δ 9.55 (d, *J* = 0.8 Hz, 1H), 8.02 (dd, *J* = 7.8, 1.4 Hz, 1H), 7.64 – 7.56 (m, 3H), 7.43 (td, *J* = 7.7, 0.9 Hz, 1H), 7.40 – 7.30 (m, 4H), 7.27 (dd, *J* = 6.5, 2.0 Hz, 2H), 7.05 (ddd, *J* = 7.6, 6.7, 2.0 Hz, 1H), 6.91 – 6.85 (m, 2H), 6.80 – 6.75 (m, 1H), 4.02 (dd, *J* = 16.3, 1.5 Hz, 1H), 3.40 (d, *J* = 16.3 Hz, 1H), 3.16 (dd, *J* = 9.1, 2.0 Hz, 1H), 3.09 – 2.95 (m, 2H), 2.60 – 2.49 (m, 1H), 2.45 (s, 3H), 1.20 (d, *J* = 6.8 Hz, 3H), 1.10 (d, *J* = 6.9 Hz, 3H), 0.96 (d, *J* = 6.8 Hz, 3H).

^{13}C-NMR (101 MHz, CDCl$_3$) δ 195.3 (C$_q$), 191.4 (CH), 151.5 (C$_q$), 148.6 (C$_q$), 144.0 (C$_q$), 144.0 (C$_q$), 139.1 (C$_q$), 135.1 (C$_q$), 134.4 (C$_q$), 134.3 (C$_q$), 134.2 (C$_q$), 133.6 (CH), 133.4 (C$_q$), 132.2 (C$_q$), 129.9 (CH), 129.8 (CH), 129.4 (CH), 129.1 (CH), 129.1 (CH), 128.5 (CH), 127.8 (CH), 127.5 (CH), 125.6 (CH), 124.9 (CH), 54.8 (CH$_2$), 51.0 (CH$_2$), 36.2 (CH), 30.8 (CH), 24.2 (CH$_3$), 22.8 (CH$_3$), 21.6 (CH$_3$), 19.6 (CH$_3$).

IR (ATR): 1693, 1490, 1347, 1236, 1159, 1089, 1035, 814, 664 cm^{-1}.

HR-MS (ESI): *m*/*z* calcd. for [$C_{36}H_{34}ClNO_4S$ + Na]$^+$ 634.1789, found 634.1776.

[α_D^{20}] = +136.2 (c = 1, DCM).

HPLC separation (Chiralpak® IA-3, *n*-hexane/*i*-PrOH 90:10, 1.0 mL/min, detection at 250 nm): t_r (minor) = 13.7 min, t_r (major) = 17.6 min, >99% e.e.

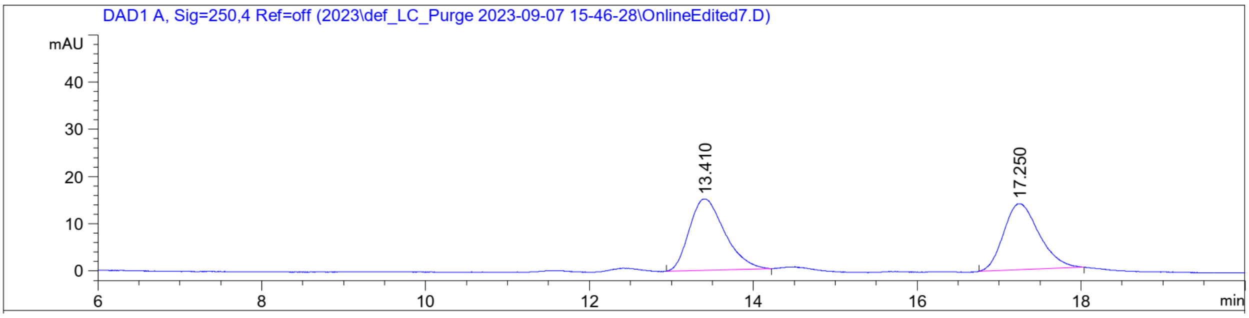

Peak #	RetTime [min]	Type	Width [min]	Area [mAU*s]	Height [mAU]	Area %
1	13.410	BB	0.3541	453.17630	15.09994	51.7697
2	17.250	BB	0.3544	422.19376	14.01607	48.2303

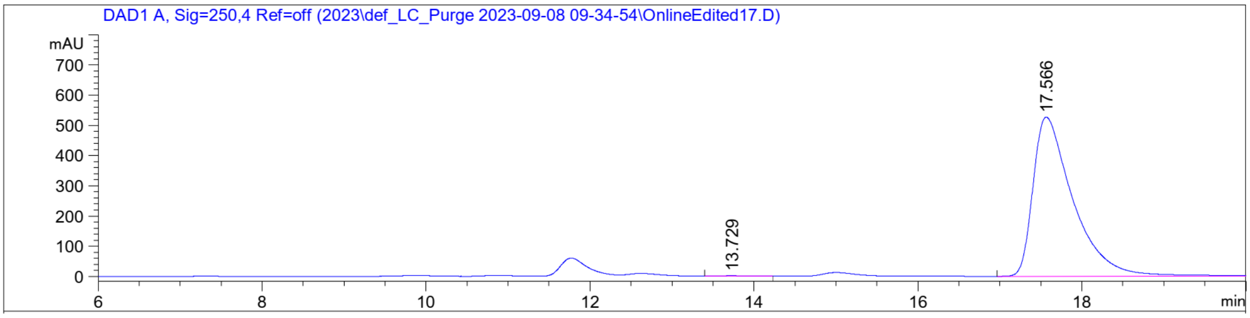

Peak #	RetTime [min]	Type	Width [min]	Area [mAU*s]	Height [mAU]	Area %
1	13.729	BB	0.2853	35.49738	1.46690	0.1981
2	17.566	BB	0.4883	1.78847e4	526.57245	99.8019

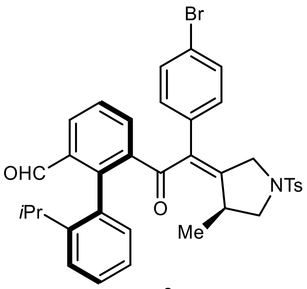

3c

(*S*)-6-((*E*)-2-(4-bromophenyl)-2-((*S*)-4-methyl-1-tosylpyrrolidin-3-ylidene)acetyl)-2'-isopropyl-[1,1'-biphenyl]-2-carbaldehyde (3c)

The general procedure was followed using bialdehyde **1a** (0.11 mmol, 27.8 mg) and 1,6-enyne **2c** (0.10 mmol, 40.4 mg) at 40 °C for 24 h. Purification by column chromatography on silica gel (DCM) yielded **3c** (24.2 mg, 57%, >20:1 d.r., >99% e.e.) as a colorless oil.

^{1}H-NMR (400 MHz, CDCl$_3$) δ 9.53 (d, *J* = 0.8 Hz, 1H), 8.01 (dd, *J* = 7.8, 1.4 Hz, 1H), 7.61 – 7.55 (m, 3H), 7.44 – 7.39 (m, 3H), 7.36 – 7.30 (m, 4H), 7.03 (ddd, *J* = 8.6, 6.8, 1.9 Hz, 1H), 6.82 – 6.73 (m, 3H), 4.00 (dd, *J* = 16.3, 1.5 Hz, 1H), 3.38 (d, *J* = 16.3 Hz, 1H), 3.14 (dd, *J* = 9.1, 2.0 Hz, 1H), 3.08 – 2.95 (m, 2H), 2.57 – 2.48 (m, 1H), 2.43 (s, 3H), 1.18 (d, *J* = 6.8 Hz, 3H), 1.08 (d, *J* = 6.9 Hz, 3H), 0.94 (d, *J* = 6.9 Hz, 3H).

^{13}C-NMR (101 MHz, CDCl$_3$) δ 195.2 (C$_q$), 191.4 (CH), 151.4 (C$_q$), 148.6 (C$_q$), 144.0 (C$_q$), 143.9 (C$_q$), 139.0 (C$_q$), 135.1 (C$_q$), 134.9 (C$_q$), 134.2 (C$_q$), 133.6 (CH), 133.4 (C$_q$), 132.2 (C$_q$), 132.1 (CH), 130.2 (CH), 129.8 (CH), 129.5 (CH), 129.1 (CH), 128.5 (CH), 127.8 (CH), 127.6 (CH), 125.6 (CH), 124.9 (CH), 122.5 (C$_q$), 54.8 (CH$_2$), 50.9 (CH$_2$), 36.2 (CH), 30.8 (CH), 24.2 (CH$_3$), 22.8 (CH$_3$), 21.6 (CH$_3$), 19.6 (CH$_3$).

IR (ATR): 1677, 1570, 1488, 1454, 1347, 1236, 1160, 1090, 814, 736 cm^{-1}.

HR-MS (ESI): *m*/*z* calcd. for [$C_{36}H_{34}BrNO_4S$+ Na]$^+$ 678.1284, found 678.1272.

[α_D^{20}] = +83.2 (c = 1.0, DCM).

HPLC separation (Chiralpak® IA-3, *n*-hexane/*i*-PrOH 90:10, 1.0 mL/min, detection at 250 nm): t_r (minor) = 13.6 min, t_r (major) = 20.5 min, >99% e.e.

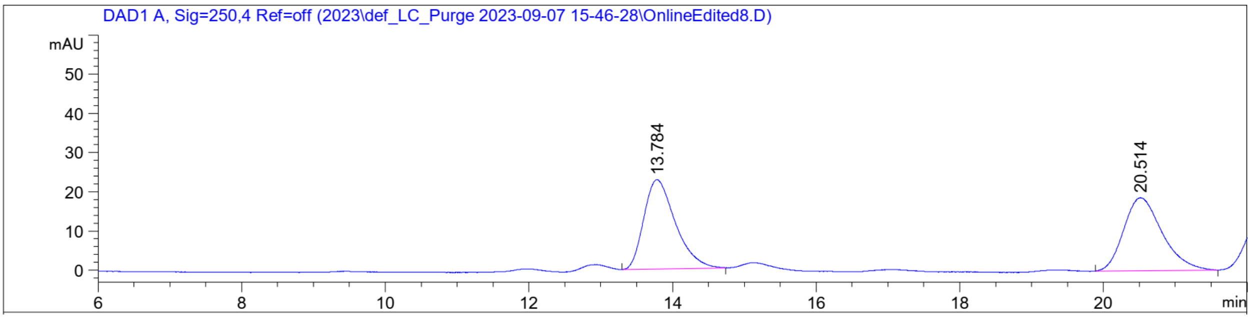

Peak #	RetTime [min]	Type	Width [min]	Area [mAU*s]	Height [mAU]	Area %
1	13.784	BB	0.3630	694.35730	22.75668	50.0717
2	20.514	BB	0.4371	692.36853	18.60636	49.9283

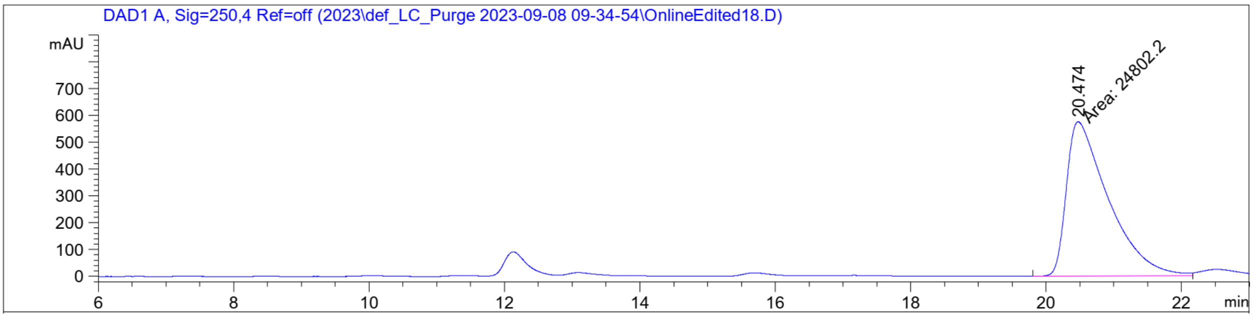

Peak #	RetTime [min]	Type	Width [min]	Area [mAU*s]	Height [mAU]	Area %
1	20.474	MF	0.7189	2.48022e4	575.03253	100.0000

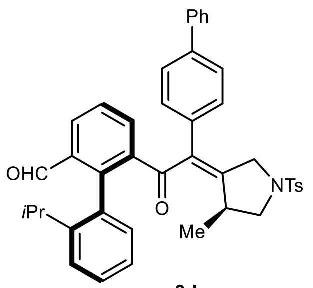

3d

(*S*)-6-((*E*)-2-([1,1'-biphenyl]-4-yl)-2-((*S*)-4-methyl-1-tosylpyrrolidin-3-ylidene)acetyl)-2'-isopropyl-[1,1'-biphenyl]-2-carbaldehyde (3d)

The general procedure was followed using bialdehyde **1a** (0.11 mmol, 27.8 mg) and 1,6-enyne **2d** (0.10 mmol, 40.2 mg) at 40 °C for 24 h. Purification by column chromatography on silica gel (DCM) yielded **3d** (43.1 mg, 66%, >20:1 d.r., >99% e.e.) as a colorless oil.

^{1}H-NMR (300 MHz, CDCl$_3$) δ 9.55 (d, *J* = 0.8 Hz, 1H), 8.00 (dd, *J* = 7.8, 1.4 Hz, 1H), 7.66 – 7.57 (m, 4H), 7.56 – 7.45 (m, 4H), 7.37 (dddd, *J* = 14.4, 13.0, 5.4, 4.2 Hz, 7H), 7.09 – 7.03 (m, 1H), 7.03 – 6.98 (m, 2H), 6.82 (dd, *J* = 7.4, 1.2 Hz, 1H), 4.11 (dd, *J* = 16.2, 1.5 Hz, 1H), 3.52 (d, *J* = 16.2 Hz, 1H), 3.21 – 2.96 (m, 3H), 2.63 – 2.50 (m, 1H), 2.43 (s, 3H), 1.21 (d, *J* = 6.7 Hz, 3H), 1.12 (d, *J* = 6.6 Hz, 3H), 0.96 (d, *J* = 6.9 Hz, 3H).

^{13}C-NMR (75 MHz, CDCl$_3$) δ 195.6 (C$_q$), 191.5 (CH), 150.6 (C$_q$), 148.6 (C$_q$), 144.1 (C$_q$), 143.9 (C$_q$), 140.9 (C$_q$), 140.0 (CH), 139.1 (C$_q$), 135.1 (C$_q$), 135.0 (C$_q$), 134.8 (C$_q$), 134.0 (C$_q$), 133.5 (C$_q$), 132.3 (C$_q$), 129.7 (CH), 129.3 (CH), 129.0 (CH), 129.0 (CH), 128.9 (CH), 128.5 (CH), 127.8 (CH), 127.7 (CH), 127.4 (CH), 127.4 (CH), 127.0 (CH), 125.5 (CH), 124.9 (CH), 54.8 (CH$_2$), 51.0 (CH$_2$), 36.1 (CH), 30.8 (CH), 24.2 (CH$_3$), 22.9 (CH$_3$), 21.6 (CH$_3$), 19.7 (CH$_3$).

IR (ATR): 1693, 1590, 1448, 1349, 1236, 1161, 1090, 764 cm^{-1}.

HR-MS (ESI): *m*/*z* calcd. for [$C_{42}H_{39}NO_4S$ + Na]$^+$ 676.2492, found 676.2493.

[α^{20}_{D}] = +78.1 (c = 1.0, DCM).

HPLC separation (Chiralpak® IA-3, *n*-hexane/*i*-PrOH 90:10, 1.0 mL/min, detection at 273 nm): t_r (major) = 33.0 min, >99% e.e.

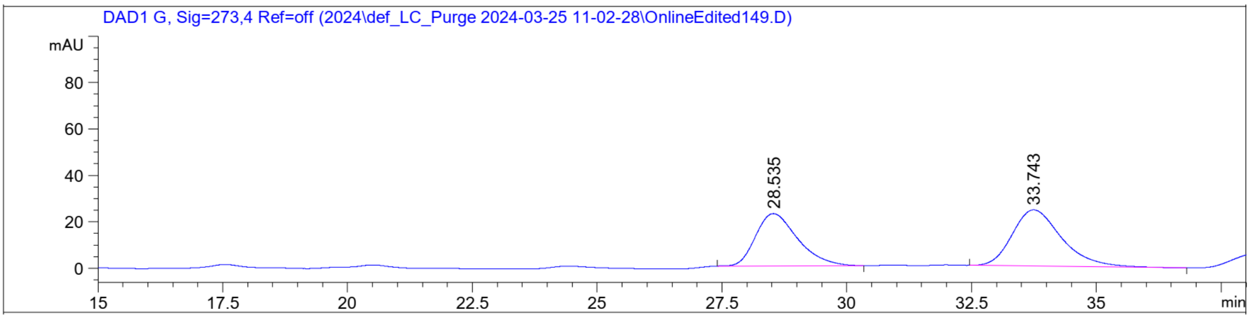

Peak #	RetTime [min]	Type	Width [min]	Area [mAU*s]	Height [mAU]	Area %
1	28.535	BB	0.7106	1318.21143	22.53172	43.9513
2	33.743	BB	0.8355	1681.04321	24.25712	56.0487

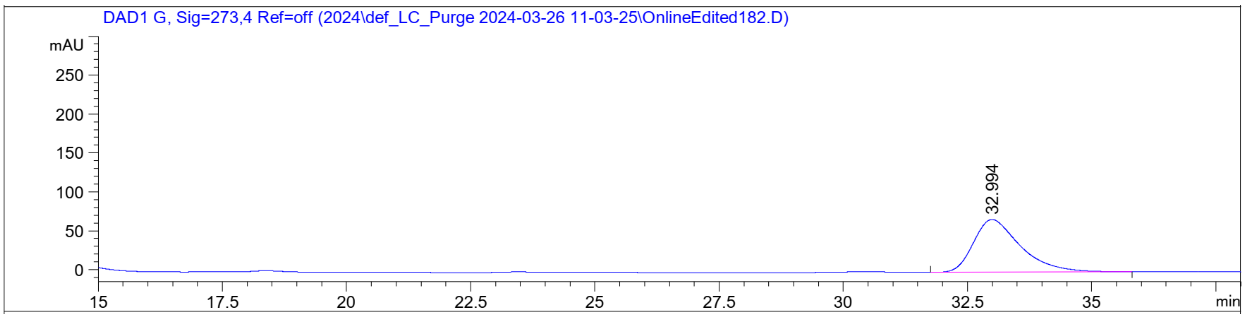

Peak #	RetTime [min]	Type	Width [min]	Area [mAU*s]	Height [mAU]	Area %
1	32.994	BB	0.9265	4451.98584	67.76096	100.0000

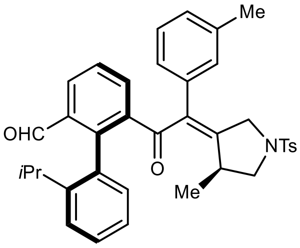

3e

(*S*)-2'-isopropyl-6-((*E*)-2-((*S*)-4-methyl-1-tosylpyrrolidin-3-ylidene)-2-(m-tolyl)acetyl)-[1,1'-biphenyl]-2-carbaldehyde (3e)

The general procedure was followed using bialdehyde **1a** (0.11 mmol, 27.8 mg) and 1,6-enyne **2e** (0.10 mmol, 33.9 mg) at 40 °C for 24 h. Purification by column chromatography on silica gel (DCM) yielded **3e** (36.7 mg, 62%, >20:1 d.r., >99% e.e.) as a colorless oil.

^{1}H-NMR (300 MHz, $CDCl_3$) δ 9.53 (d, J = 0.8 Hz, 1H), 7.98 (dd, J = 7.8, 1.4 Hz, 1H), 7.63 – 7.55 (m, 3H), 7.41 – 7.28 (m, 5H), 7.16 (t, J = 7.6 Hz, 1H), 7.03 (ddd, J = 7.5, 6.6, 1.9 Hz, 2H), 6.77 – 6.65 (m, 3H), 4.06 (dd, J = 16.2, 1.5 Hz, 1H), 3.45 (d, J = 16.3 Hz, 1H), 3.17 – 2.95 (m,

3H), 2.61 – 2.51 (m, 1H), 2.43 (s, 3H), 2.29 (s, 3H), 1.21 (d, J = 6.8 Hz, 3H), 1.10 (d, J = 6.7 Hz, 3H), 0.95 (d, J = 6.9 Hz, 3H).

^{13}C-NMR (75 MHz, CDCl$_3$) δ 195.7 (C_q), 191.6 (CH), 150.3 (C_q), 148.6 (C_q), 144.0 (C_q), 143.9 (C_q), 139.2 (C_q), 138.4 (C_q), 135.8 (C_q), 135.4 (C_q), 135.0 (C_q), 133.8 (CH), 133.5 (C_q), 132.3 (C_q), 129.7 (CH), 129.7 (CH), 129.2 (CH), 129.2 (CH), 128.9 (CH), 128.7 (CH), 128.6 (CH), 127.8 (CH), 127.3 (CH), 125.6 (CH), 125.5 (CH), 124.7 (CH), 54.9 (CH_2), 50.9 (CH_2), 36.0 (CH), 30.9 (CH), 24.2 (CH_3), 22.9 (CH_3), 21.6 (CH_3), 21.4 (CH_3), 19.7 (CH_3).

IR (ATR): 1693, 1677, 1598, 1454, 1348, 1232, 1160, 1093, 814, 762 cm^{-1}.

HR-MS (ESI): m/z calcd. for $[C_{37}H_{37}NO_4S + Na]^+$ 614.2336, found 614.2336.

$[\alpha^{20}_{D}]$ = +93.2 (c = 1.0, DCM).

HPLC separation (Chiralpak® IA-3, n-hexane/i-PrOH 90:10, 1.0 mL/min, detection at 250 nm): t_r (major) = 10.4 min, >99% e.e.

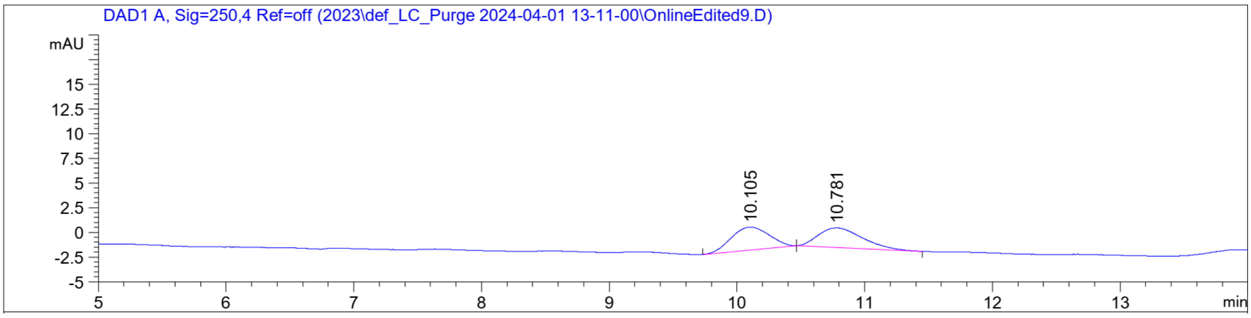

Peak #	RetTime [min]	Type	Width [min]	Area [mAU*s]	Height [mAU]	Area %
1	10.105	BB	0.2494	47.68170	2.31379	49.8402
2	10.781	BB	0.2871	47.98746	1.98482	50.1598

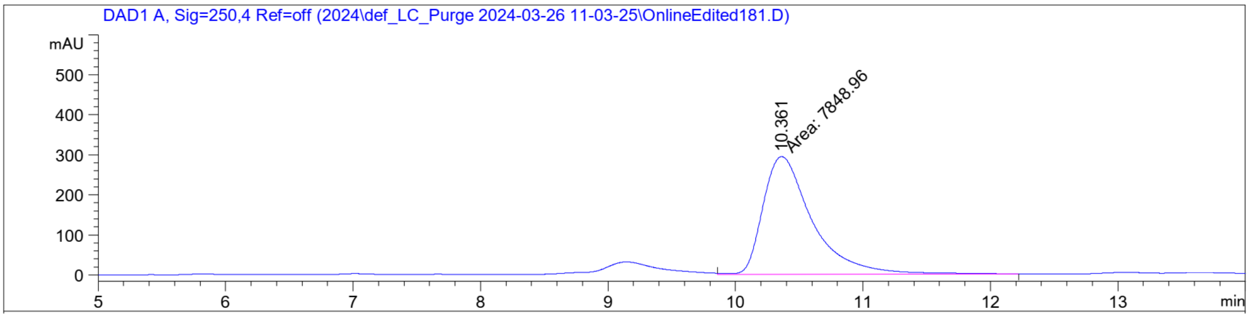

Peak #	RetTime [min]	Type	Width [min]	Area [mAU*s]	Height [mAU]	Area %
1	10.361	FM	0.4457	7848.95654	293.52161	100.0000

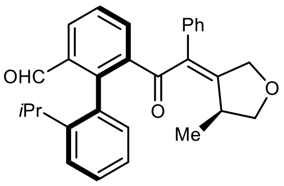

3f

(*S*)-2'-isopropyl-6-((*E*)-2-((*S*)-4-methyldihydrofuran-3(2H)-ylidene)-2-phenylacetyl)-[1,1'-biphenyl]-2-carbaldehyde (3f)

The general procedure was followed using bialdehyde **1a** (0.11 mmol, 27.8 mg) and 1,6-enyne **2f** (0.10 mmol, 17.2 mg) at 40 °C for 24 h. Purification by column chromatography on silica gel (DCM) yielded **3f** (30.6 mg, 72%, 14:1 d.r., >99% e.e.) as a colorless oil.

^{1}H-NMR (300 MHz, $CDCl_3$) δ 9.50 (d, *J* = 0.8 Hz, 1H), 7.92 (dd, *J* = 7.8, 1.4 Hz, 1H), 7.63 (dd, *J* = 7.7, 1.4 Hz, 1H), 7.37 – 7.29 (m, 3H), 7.21 – 7.08 (m, 4H), 6.97 – 6.91 (m, 2H), 6.79 (dt, *J* = 7.6, 1.0 Hz, 1H), 4.39 (dd, *J* = 15.6, 1.8 Hz, 1H), 3.98 (d, *J* = 15.5 Hz, 1H), 3.74 (dd, *J* = 8.5, 6.0 Hz, 1H), 3.57 – 3.49 (m, 1H), 3.07 (ddddd, *J* = 12.1, 7.0, 5.2, 3.3, 1.6 Hz, 1H), 2.53 (p, *J* = 6.9 Hz, 1H), 1.20 (d, *J* = 6.8 Hz, 3H), 1.05 (d, *J* = 7.0 Hz, 3H), 0.91 (d, *J* = 6.9 Hz, 3H).

^{13}C-NMR (75 MHz, $CDCl_3$) δ 195.8 (C_q), 191.7 (CH), 155.9 (C_q), 148.6 (C_q), 144.3 (C_q), 139.4 (C_q), 136.8 (C_q), 135.0 (C_q), 134.1 (CH), 133.7 (C_q), 132.8 (C_q), 129.0 (CH), 129.0 (CH), 128.6 (2C, CH), 128.5 (CH), 127.8 (CH), 127.3 (CH), 125.5 (CH), 125.0 (CH), 75.7 (CH_2), 70.6 (CH_2), 37.1 (CH), 30.9 (CH), 24.2 (CH_3), 23.0 (CH_3), 18.4 (CH_3).

IR (ATR): 1694, 1569, 1454, 1380, 1236, 1101, 927, 757 cm^{-1}.

HR-MS (ESI): *m*/*z* calcd. for $[C_{29}H_{28}O_3 + Na]^+$ 447.1931, found 447.1929.

$[\alpha_D^{20}]$ = +110.5 (c = 1.0, DCM).

HPLC separation (Chiralpak® IA-3, *n*-hexane/*i*-PrOH 95:5, 1.0 mL/min, detection at 250 nm): t_r (major) = 6.3 min, >99% e.e.

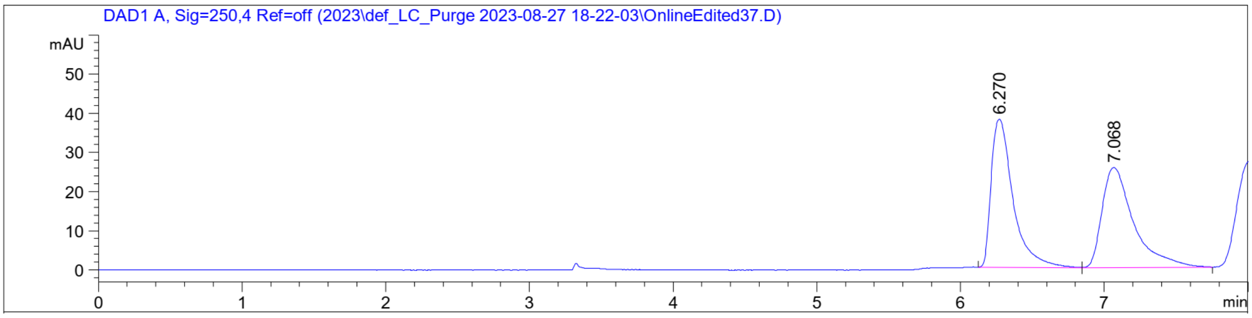

Peak #	RetTime [min]	Type	Width [min]	Area [mAU*s]	Height [mAU]	Area %
1	6.270	BB	0.1612	398.89508	37.77498	50.6101
2	7.068	BB	0.2250	389.27808	25.50848	49.3899

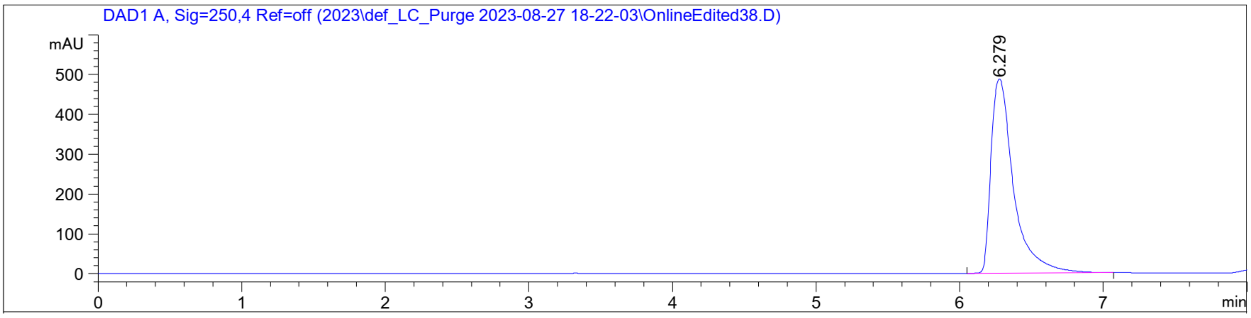

Peak #	RetTime [min]	Type	Width [min]	Area [mAU*s]	Height [mAU]	Area %
1	6.279	BB	0.1651	5310.02051	487.52545	100.0000

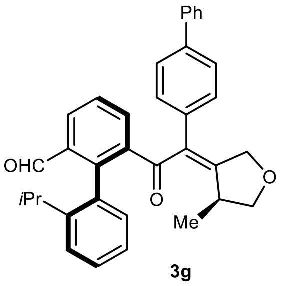

3g

(*S*)-6-((*E*)-2-([1,1'-biphenyl]-4-yl)-2-((*S*)-4-methyldihydrofuran-3(2H)-ylidene)acetyl)-2'-isopropyl-[1,1'-biphenyl]-2-carbaldehyde (3g)

The general procedure was followed using bialdehyde **1a** (0.11 mmol, 27.8 mg) and 1,6-enyne **2g** (0.10 mmol, 24.8 mg) at 40 °C for 24 h. Purification by column chromatography on silica gel (DCM) yielded **3g** (32.5 mg, 65%, >20:1 d.r., 98% e.e.) as a colorless oil.

^{1}H-NMR (300 MHz, CDCl$_3$) δ 9.58 (d, J = 0.8 Hz, 1H), 8.02 (dd, J = 7.8, 1.4 Hz, 1H), 7.77 (dd, J = 7.7, 1.4 Hz, 1H), 7.57 – 7.47 (m, 4H), 7.46 – 7.39 (m, 5H), 7.37 – 7.31 (m, 1H), 7.21 (dt, J = 7.6, 4.3 Hz, 1H), 7.13 – 7.06 (m, 2H), 6.91 (dt, J = 7.6, 1.0 Hz, 1H), 4.54 (dd, J = 15.5, 1.8 Hz, 1H), 4.14 (d, J = 15.5 Hz, 1H), 3.84 (dd, J = 8.5, 6.0 Hz, 1H), 3.63 (dd, J = 8.5, 3.5 Hz, 1H), 3.17 (s, 1H), 2.62 (p, J = 6.8 Hz, 1H), 1.28 (d, J = 6.8 Hz, 3H), 1.14 (d, J = 7.0 Hz, 3H), 1.00 (d, J = 6.9 Hz, 3H).

^{13}C-NMR (101 MHz, CDCl$_3$) δ 195.9 (C$_q$), 191.7 (CH), 155.6 (C$_q$), 148.6 (C$_q$), 144.4 (C$_q$), 140.5 (C$_q$), 140.1 (C$_q$), 139.4 (C$_q$), 135.7 (C$_q$), 135.1 (C$_q$), 134.3 (CH), 133.7 (C$_q$), 132.5 (C$_q$), 129.2 (CH), 129.0 (CH), 129.0 (CH), 128.8 (CH), 128.5 (CH), 127.6 (CH), 127.4 (CH), 127.3 (CH),

127.0 (CH), 125.5 (CH), 125.0 (CH), 75.7 (CH_2), 70.7 (CH_2), 37.2 (CH), 30.9 (CH), 24.2 (CH_3), 23.0 (CH_3), 18.4 (CH_3).

IR (ATR): 1681, 1487, 1454, 1278, 1199, 1093, 763, 681 cm^{-1}.

HR-MS (ESI): *m*/*z* calcd. for $[C_{35}H_{32}O_3 + Na]^+$ 523.2244, found 523.2235.

$[\alpha_D^{20}]$ = +130.6 (c = 1.0, DCM).

HPLC separation (Chiralpak® IA-3, *n*-hexane/*i*-PrOH 97:3, 1.0 mL/min, detection at 273 nm): t_r (minor) = 12.6 min, t_r (major) = 18.3 min, 98% e.e.

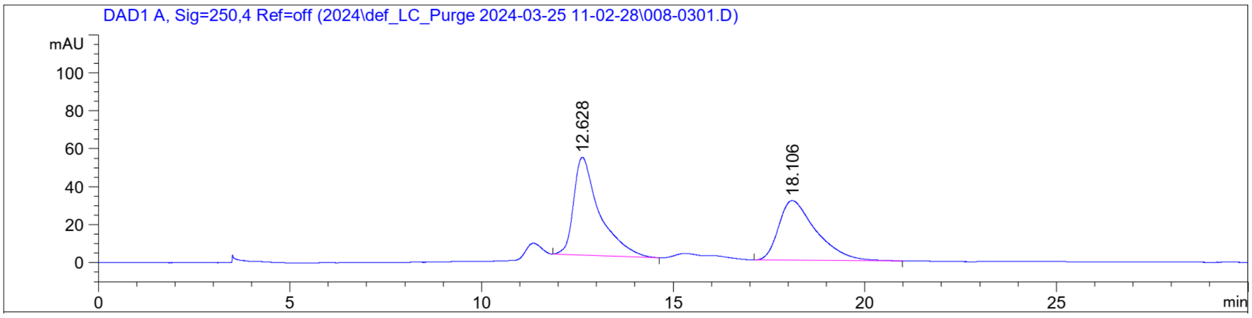

Peak #	RetTime [min]	Type	Width [min]	Area [mAU*s]	Height [mAU]	Area %
1	12.628	BB	0.6530	2401.63721	51.50329	53.0509
2	18.106	BB	0.8984	2125.40283	31.37317	46.9491

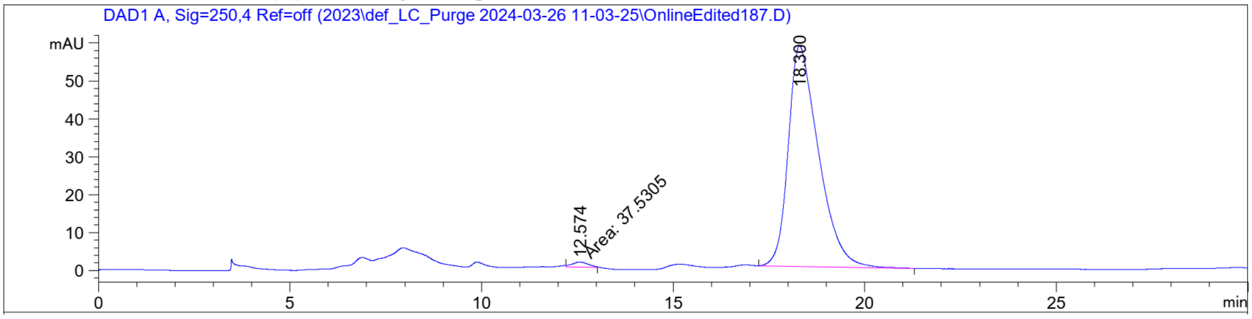

Peak #	RetTime [min]	Type	Width [min]	Area [mAU*s]	Height [mAU]	Area %
1	12.574	MM	0.4821	37.53045	1.29759	1.1593
2	18.300	BB	0.7777	3199.69312	58.17762	98.8407

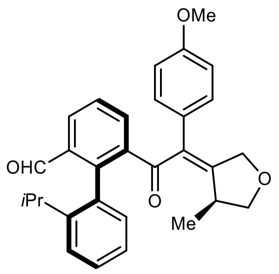

3h

(S)-2'-isopropyl-6-((E)-2-(4-methoxyphenyl)-2-((S)-4-methyldihydrofuran-3(2H)-ylidene)acetyl)-[1,1'-biphenyl]-2-carbaldehyde (3h)

The general procedure was followed using bialdehyde **1a** (0.11 mmol, 27.8 mg) and 1,6-enyne **2h** (0.10 mmol, 20.2 mg) at 40 °C for 24 h. Purification by column chromatography on silica gel (DCM) yielded **3h** (32.2 mg, 71%, 17:1 d.r., 96% e.e.) as a colorless oil.

^{1}H-NMR (400 MHz, $CDCl_3$) δ 9.57 (d, J = 0.8 Hz, 1H), 8.00 (dd, J = 7.8, 1.4 Hz, 1H), 7.70 (dd, J = 7.7, 1.4 Hz, 1H), 7.43 – 7.38 (m, 3H), 7.19 (ddd, J = 7.6, 5.1, 3.6 Hz, 1H), 6.96 – 6.91 (m, 2H), 6.87 (dt, J = 7.5, 1.0 Hz, 1H), 6.81 – 6.76 (m, 2H), 4.46 (dd, J = 15.5, 1.8 Hz, 1H), 4.07 (d, J = 15.4 Hz, 1H), 3.85 – 3.77 (m, 2H), 3.77 (s, 3H), 3.60 (ddd, J = 8.5, 3.4, 0.7 Hz, 1H), 3.17 – 3.07 (m, 1H), 2.61 (p, J = 6.9 Hz, 1H), 1.27 (d, J = 6.8 Hz, 3H), 1.12 (d, J = 7.0 Hz, 3H), 0.99 (d, J = 6.9 Hz, 3H).

^{13}C-NMR (101 MHz, $CDCl_3$) δ 196.1 (C_q), 191.8 (CH), 159.0 (C_q), 155.2 (C_q), 148.6 (C_q), 144.3 (C_q), 139.5 (C_q), 135.1 (C_q), 134.2 (CH), 133.7 (C_q), 132.4 (C_q), 129.9 (CH), 129.0 (C_q), 129.0 (CH), 128.9 (CH), 128.5 (CH), 127.3 (CH), 125.5 (CH), 125.0 (CH), 114.0 (CH), 75.7 (CH_2), 70.6 (CH_2), 55.2 (CH_3), 37.0 (CH), 30.9 (CH), 24.2 (CH_3), 23.0 (CH_3), 18.4 (CH_3).

IR (ATR): 1691, 1606, 1511, 1248, 1176, 1034, 762, 735 cm^{-1}.

HR-MS (ESI): m/z calcd. for $[C_{30}H_{30}O_4 + Na]^+$ 477.2036, found 477.2035.

$[\alpha_D^{20}]$ = +76.3 (c = 1.0, DCM).

HPLC separation (Chiralpak® IE-3, n-hexane/i-PrOH 90:10, 1.0 mL/min, detection at 273 nm): t_r (major) = 10.2 min, t_r (minor) = 11.5 min, 96% e.e.

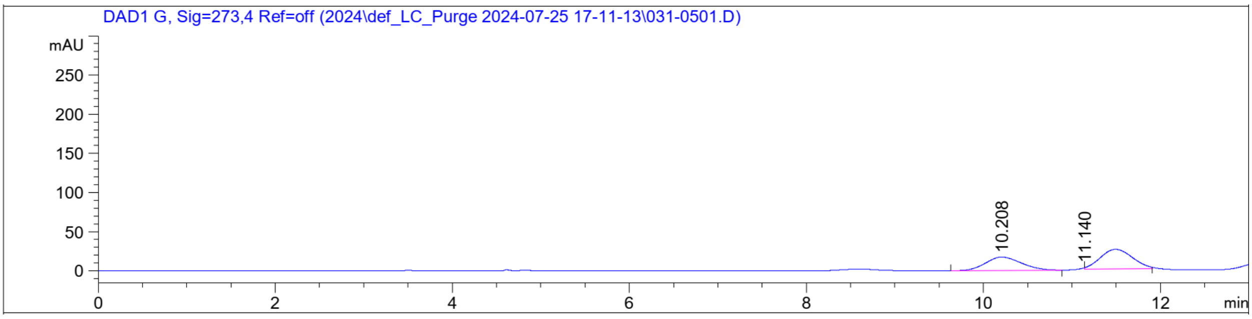

Peak #	RetTime [min]	Type	Width [min]	Area [mAU*s]	Height [mAU]	Area %
1	10.208	BB	0.3801	474.98407	17.04792	42.9416
2	11.140	MM R	0.4198	631.13281	1.90312	57.0584

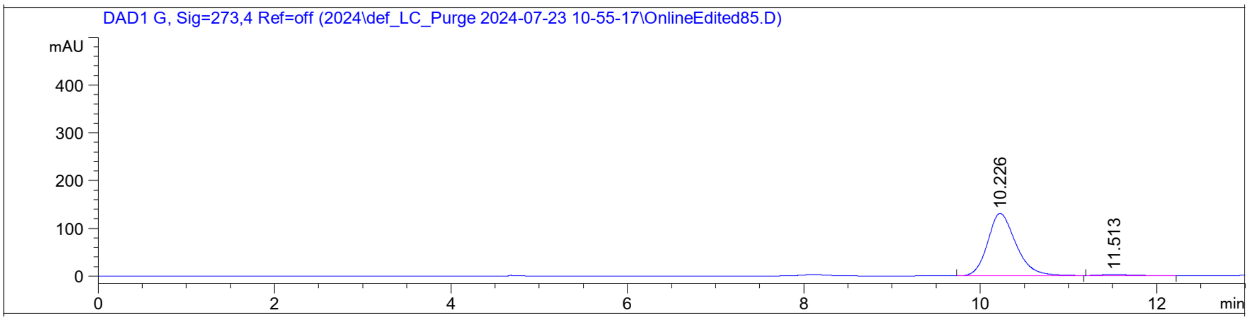

Peak #	RetTime [min]	Type	Width [min]	Area [mAU*s]	Height [mAU]	Area %
1	10.226	BB	0.3367	2891.23560	130.39615	98.1443
2	11.513	BB	0.3031	54.66719	2.14700	1.8557

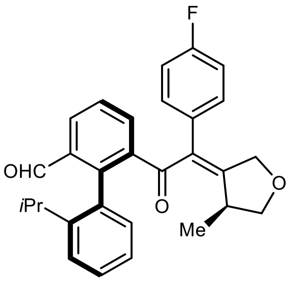

3i

(*S*)-2'-isopropyl-6-((*E*)-2-(4-methoxyphenyl)-2-((*S*)-4-methyldihydrofuran-3(2H)-ylidene)acetyl)-[1,1'-biphenyl]-2-carbaldehyde (3i)

The general procedure was followed using bialdehyde **1a** (0.11 mmol, 27.8 mg) and 1,6-enyne **2i** (0.10 mmol, 19.0 mg) at 40 °C for 24 h. Purification by column chromatography on silica gel (DCM) yielded **3i** (29.2 mg, 66%, 16:1 d.r., 99% e.e.) as a colorless oil.

^{1}H-NMR (300 MHz, CDCl$_3$) δ 9.57 (d, J = 0.9 Hz, 1H), 8.02 (dd, J = 7.8, 1.4 Hz, 1H), 7.68 (dd, J = 7.6, 1.4 Hz, 1H), 7.48 – 7.38 (m, 3H), 7.22 – 7.15 (m, 1H), 7.00 – 6.92 (m, 4H), 6.84 (dt, J = 7.6, 1.0 Hz, 1H), 4.42 (dd, J = 15.6, 1.7 Hz, 1H), 4.02 (d, J = 15.6 Hz, 1H), 3.80 (dd, J = 8.6, 5.9 Hz, 1H), 3.61 (ddd, J = 8.5, 3.3, 0.7 Hz, 1H), 3.19 – 3.04 (m, 1H), 2.59 (p, J = 6.8 Hz, 1H), 1.25 (d, J = 6.8 Hz, 3H), 1.11 (d, J = 7.0 Hz, 3H), 0.98 (d, J = 6.9 Hz, 3H).

^{13}C-NMR (101 MHz, CDCl$_3$) δ 195.8 (C$_q$), 191.6 (CH), 162.1 (d, J = 248.1 Hz, C$_q$), 156.3 (C$_q$), 148.7 (C$_q$), 144.1 (C$_q$), 139.5 (C$_q$), 135.1 (C$_q$), 133.8 (CH), 133.5 (C$_q$), 132.8 (d, J = 3.4 Hz, C$_q$), 131.9 (C$_q$), 130.4 (CH), 130.4 (CH), 129.1 (d, J = 11.6 Hz, CH), 128.6 (CH), 127.4 (CH), 125.6

(CH), 125.0 (CH), 115.7 (d, J = 21.5 Hz, CH), 75.7 (CH_2), 70.6 (CH_2), 37.2 (CH), 30.9 (CH), 24.3 (CH_3), 22.9 (CH_3), 18.4 (CH_3).

^{19}F-NMR (282 MHz, $CDCl_3$) δ -113.44.

IR (ATR): 1681, 1507, 1455, 1385, 1228, 1091, 1015, 840, 761, 737 cm^{-1}.

HR-MS (ESI): *m*/*z* calcd. for $[C_{29}H_{27}FO_3 + Na]^+$ 465.1836, found 465.1844.

$[\alpha^{20}_{D}]$ = +69.9 (c = 1.0, DCM).

HPLC separation (Chiralpak® IE-3, *n*-hexane/*i*-PrOH 97:3, 1.0 mL/min, detection at 273 nm): t_r (major) = 11.3 min, t_r (minor) = 12.8 min, 99% e.e.

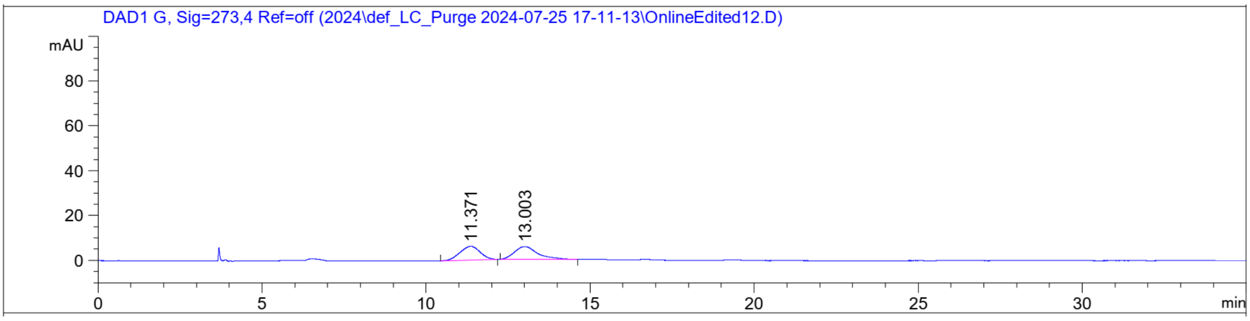

Peak #	RetTime [min]	Type	Width [min]	Area [mAU*s]	Height [mAU]	Area %
1	11.371	BB	0.5013	261.54376	6.13643	47.9897
2	13.003	BB	0.5831	283.45645	5.73076	52.0103

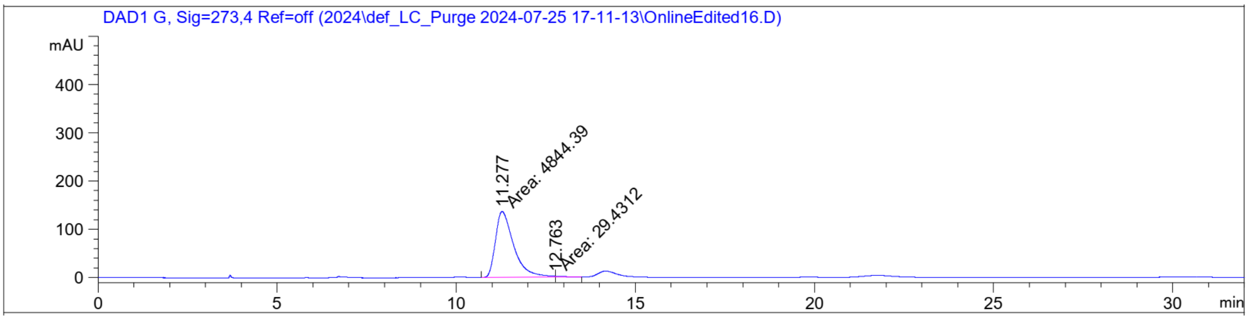

Peak #	RetTime [min]	Type	Width [min]	Area [mAU*s]	Height [mAU]	Area %
1	11.277	MF	0.5919	4844.39258	136.39786	99.3961
2	12.763	FM	0.2589	29.43119	1.89467	0.6039

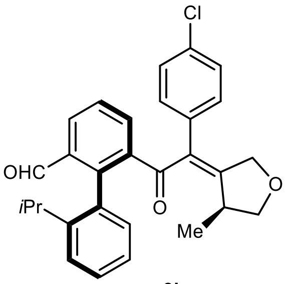

3j

(*S*)-6-((*E*)-2-(4-chlorophenyl)-2-((*S*)-4-methyldihydrofuran-3(2H)-ylidene)acetyl)-2'-isopropyl-[1,1'-biphenyl]-2-carbaldehyde (3j)

The general procedure was followed using bialdehyde **1a** (0.11 mmol, 27.8 mg) and 1,6-enyne **2j** (0.10 mmol, 20.7 mg) at 40 °C for 24 h. Purification by column chromatography on silica gel (DCM) yielded **3j** (28.9 mg, 63%, >20:1 d.r., 92% e.e.) as a colorless oil.

^{1}H-NMR (300 MHz, CDCl$_3$) δ 9.57 (d, *J* = 0.8 Hz, 1H), 8.03 (dd, *J* = 7.8, 1.4 Hz, 1H), 7.69 (dd, *J* = 7.6, 1.4 Hz, 1H), 7.47 – 7.37 (m, 3H), 7.26 – 7.16 (m, 3H), 7.00 – 6.88 (m, 2H), 6.85 (dt, *J* = 7.6, 1.0 Hz, 1H), 4.42 (dd, *J* = 15.6, 1.7 Hz, 1H), 4.02 (d, *J* = 15.6 Hz, 1H), 3.81 (dd, *J* = 8.6, 6.0 Hz, 1H), 3.61 (dd, *J* = 8.5, 3.3 Hz, 1H), 3.18 – 3.03 (m, 1H), 2.59 (p, *J* = 6.8 Hz, 1H), 1.25 (d, *J* = 6.8 Hz, 3H), 1.11 (d, *J* = 7.0 Hz, 3H), 0.98 (d, *J* = 6.9 Hz, 3H).

^{13}C-NMR (75 MHz, CDCl$_3$) δ 195.5 (C_q), 191.6 (CH), 156.4 (C_q), 148.7 (C_q), 144.2 (C_q), 139.4 (C_q), 135.2 (C_q), 135.1 (C_q), 133.9 (CH), 133.8 (C_q), 133.5 (C_q), 131.8 (C_q), 129.9 (CH), 129.3 (CH), 129.1 (CH), 128.9 (CH), 128.5 (CH), 127.5 (CH), 125.6 (CH), 125.0 (CH), 75.6 (CH_2), 70.5 (CH_2), 37.2 (CH), 30.9 (CH), 24.2 (CH_3), 22.9 (CH_3), 18.3 (CH_3).

IR (ATR): 1727, 1693, 1586, 1458, 1386, 1278, 1237, 1093, 762 cm^{-1}.

HR-MS (ESI): *m*/*z* calcd. for $[C_{29}H_{27}ClO_3 + Na]^+$ 481.1541, found 481.1548.

$[\alpha_D^{20}]$ = +83.3 (c = 1.0, DCM).

HPLC separation (Chiralpak® IE-3, *n*-hexane/*i*-PrOH 90:10, 1.0 mL/min, detection at 273 nm): t_r (major) = 7.1 min, 92% e.e.

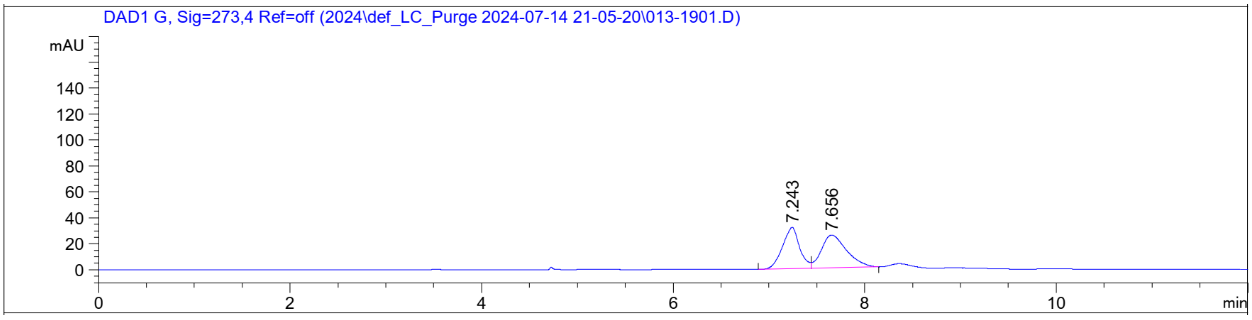

Peak #	RetTime [min]	Type	Width [min]	Area [mAU*s]	Height [mAU]	Area %
1	7.243	BV	0.1853	397.84610	31.93391	46.0992
2	7.656	VB	0.2697	465.17587	25.38995	53.9008

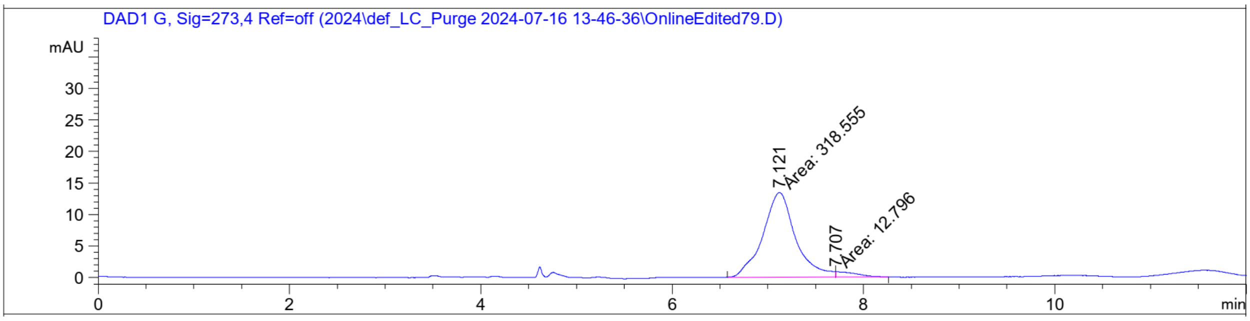

Peak #	RetTime [min]	Type	Width [min]	Area [mAU*s]	Height [mAU]	Area %
1	7.121	MF	0.3963	318.55466	13.39707	96.1382
2	7.707	FM	0.2504	12.79600	8.51816e-1	3.8618

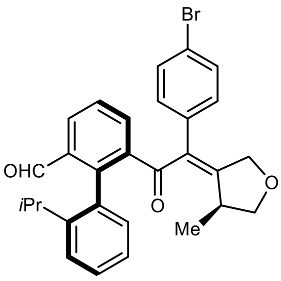

3k

(*S*)-6-((*E*)-2-(4-bromophenyl)-2-((*S*)-4-methyldihydrofuran-3(2H)-ylidene)acetyl)-2'-isopropyl-[1,1'-biphenyl]-2-carbaldehyde (3k)

The general procedure was followed using bialdehyde **1a** (0.11 mmol, 27.8 mg) and 1,6-enyne **2k** (0.10 mmol, 25.1 mg) at 40 °C for 24 h. Purification by column chromatography on silica gel (DCM) yielded **3k** (36.2 mg, 72%, >20:1 d.r., >99% e.e.) as a colorless oil.

^{1}H-NMR (400 MHz, $CDCl_3$) δ 9.57 (d, *J* = 0.9 Hz, 1H), 8.04 (dd, *J* = 7.8, 1.5 Hz, 1H), 7.72 (dd, *J* = 7.7, 1.4 Hz, 1H), 7.54 (d, *J* = 8.1 Hz, 2H), 7.49 – 7.41 (m, 3H), 7.20 (ddd, *J* = 7.5, 5.7, 2.9 Hz, 1H), 7.16 – 7.11 (m, 2H), 6.86 (dd, *J* = 7.3, 1.1 Hz, 1H), 4.43 (dd, *J* = 15.6, 1.7 Hz, 1H), 4.03 (d, *J* = 15.7 Hz, 1H), 3.82 (dd, *J* = 8.6, 6.0 Hz, 1H), 3.62 (dd, *J* = 8.5, 3.3 Hz, 1H), 3.17 – 3.06 (m, 1H), 2.59 (dq, *J* = 13.7, 6.8 Hz, 1H), 1.24 (d, *J* = 6.8 Hz, 3H), 1.11 (d, *J* = 7.0 Hz, 3H), 0.98 (d, *J* = 6.8 Hz, 3H).

^{13}C-NMR (75 MHz, $CDCl_3$) δ 195.4 (C_q), 191.6 (CH), 156.4 (C_q), 148.7 (C_q), 144.2 (C_q), 139.3 (C_q), 135.7 (C_q), 135.2 (C_q), 133.9 (CH), 133.5 (C_q), 131.9 (CH), 131.8 (C_q), 130.2 (CH), 129.3

(CH), 129.1 (CH), 128.5 (CH), 127.5 (CH), 125.6 (CH), 125.0 (CH), 122.0 (C_q), 75.6 (CH_2), 70.53 (CH_2), 37.3 (CH), 30.9 (CH), 24.2 (CH_3), 22.9 (CH_3), 18.3 (CH_3).

IR (ATR): 1683, 1585, 1487, 1456, 1362, 1235, 1092, 761 cm^{-1}.

HR-MS (ESI): *m*/*z* calcd. for $[C_{29}H_{27}BrO_3 + Na]^+$ 525.1036, found 525.1049.

$[\alpha_D^{20}]$ = +87.9 (c = 1.0, DCM).

HPLC separation (Chiralpak® IE-3, *n*-hexane/*i*-PrOH 90:10, 1.0 mL/min, detection at 273 nm): t_r (major) = 7.4 min, >99% e.e.

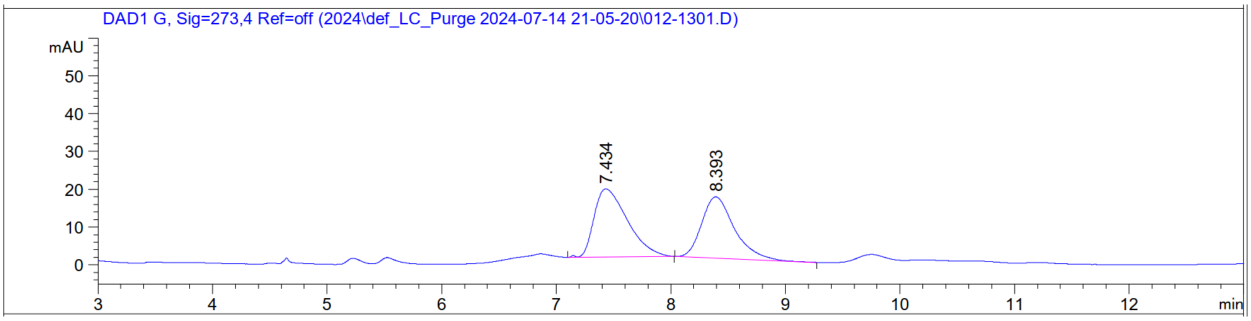

Peak #	RetTime [min]	Type	Width [min]	Area [mAU*s]	Height [mAU]	Area %
1	7.434	VB R	0.2922	364.94125	18.02743	53.0437
2	8.393	BB	0.2933	323.05927	16.22387	46.9563

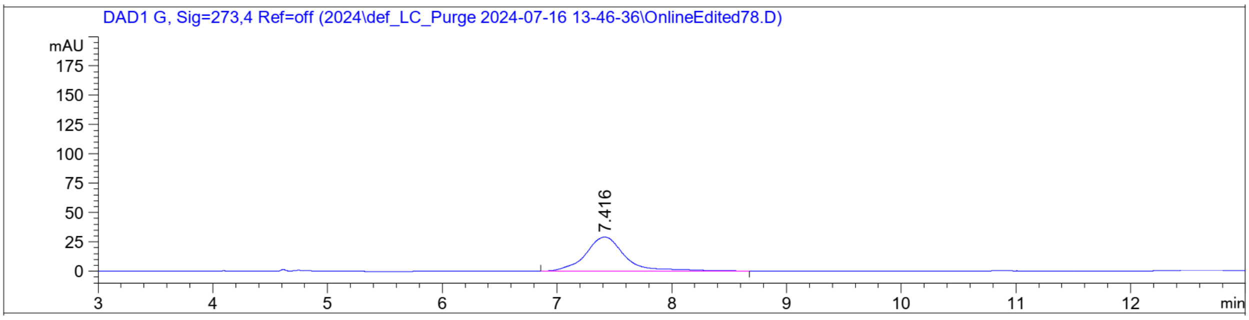

Peak #	RetTime [min]	Type	Width [min]	Area [mAU*s]	Height [mAU]	Area %
1	7.416	BB	0.3556	684.15527	28.95918	100.0000

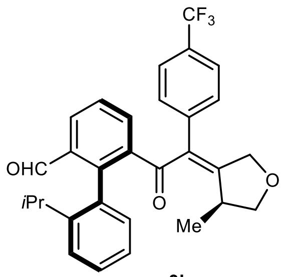

3l

(*S*)-2'-isopropyl-6-((*E*)-2-((*S*)-4-methyldihydrofuran-3(2H)-ylidene)-2-(4-(trifluoromethyl)phenyl)acetyl)-[1,1'-biphenyl]-2-carbaldehyde (3l)

The general procedure was followed using bialdehyde **1a** (0.11 mmol, 27.8 mg) and 1,6-enyne **2l** (0.10 mmol, 24.2 mg) at 40 °C for 24 h. Purification by column chromatography on silica gel (DCM) yielded **3l** (34.0 mg, 69%, 18:1 d.r., >99% e.e.) as a colorless oil.

^{1}H-NMR (400 MHz, CDCl$_3$) δ 9.57 (d, *J* = 0.9 Hz, 1H), 8.04 (dd, *J* = 7.8, 1.5 Hz, 1H), 7.72 (dd, *J* = 7.7, 1.4 Hz, 1H), 7.54 (d, *J* = 8.1 Hz, 2H), 7.49 – 7.41 (m, 3H), 7.20 (ddd, *J* = 7.5, 5.7, 2.9 Hz, 1H), 7.16 – 7.11 (m, 2H), 6.86 (dd, *J* = 7.3, 1.1 Hz, 1H), 4.43 (dd, *J* = 15.6, 1.7 Hz, 1H), 4.03 (d, *J* = 15.7 Hz, 1H), 3.82 (dd, *J* = 8.6, 6.0 Hz, 1H), 3.62 (dd, *J* = 8.5, 3.3 Hz, 1H), 3.17 – 3.06 (m, 1H), 2.59 (dq, *J* = 13.7, 6.8 Hz, 1H), 1.24 (d, *J* = 6.8 Hz, 3H), 1.11 (d, *J* = 7.0 Hz, 3H), 0.98 (d, *J* = 6.8 Hz, 3H).

^{13}C-NMR (126 MHz, CDCl$_3$) δ 195.3 (C_q), 191.5 (CH), 156.9 (C_q), 148.6 (C_q), 144.2 (C_q), 140.5 (C_q), 139.2 (C_q), 135.2 (C_q), 133.9 (CH), 133.4 (C_q), 131.8 (C_q), 129.9 (q, *J* = 32.7 Hz, C_q), 129.5 (CH), 129.1 (CH), 128.9 (CH), 128.5 (CH), 127.5 (CH), 125.6 (CH), 125.6 (q, *J* = 3.6 Hz, CH), 125.06 (CH), 123.8 (q, *J* = 272.3 Hz, C_q), 75.6 (CH_2), 70.4 (CH_2), 37.3 (CH), 30.9 (CH), 24.2 (CH_3), 22.9 (CH_3), 18.3 (CH_3).

^{19}F-NMR (282 MHz, CDCl$_3$) δ -62.72.

IR (ATR): 1691, 1487, 1454, 1385, 1278, 1235, 1093, 915, 763 cm^{-1}.

HR-MS (ESI): *m*/*z* calcd. for $[C_{30}H_{27}F_3O_3 + Na]^+$ 515.1805, found 515.1798.

$[\alpha_D^{20}]$ = +77.5 (c = 1.0, DCM).

HPLC separation (Chiralpak® AD-3, *n*-hexane/*i*-PrOH 95:5, 1.0 mL/min, detection at 250 nm): t_r (major) = 8.7 min, >99% e.e.

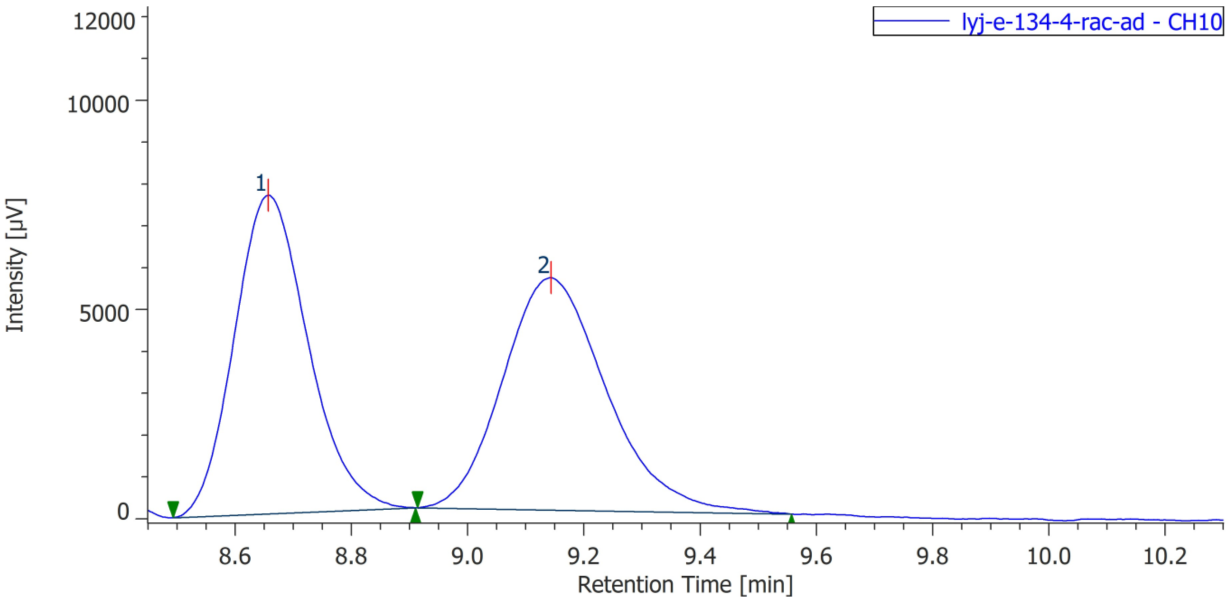

#	Peak Name	CH	tR [min]	Area [µV·sec]	Height [µV]	Area%	Height%	Quantity	NTP	Resolution	Symmetry Factor	Warning
1	Unknown	10	8.657	67199	7615	49.391	57.827	N/A	22414	1.767	1.184	
2	Unknown	10	9.143	68856	5553	50.609	42.173	N/A	12969	N/A	1.200	

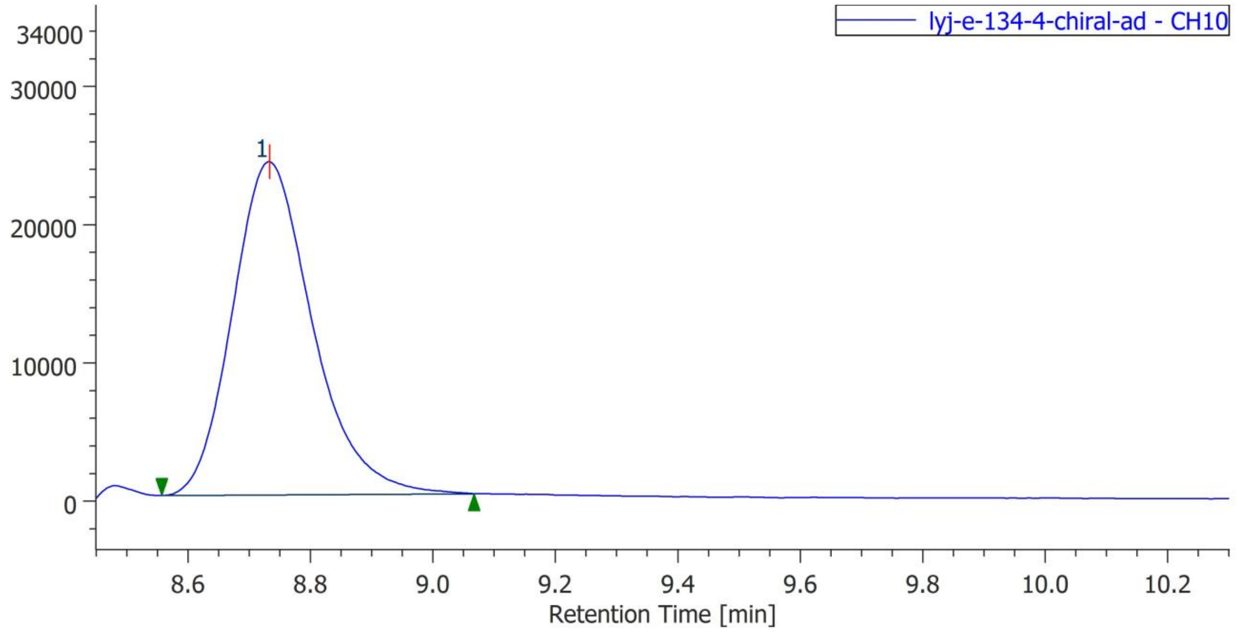

#	Peak Name	CH	tR [min]	Area [µV·sec]	Height [µV]	Area%	Height%	Quantity	NTP	Resolution	Symmetry Factor	Warning
1	Unknown	10	8.733	218628	24086	100.000	100.000	N/A	22452	N/A	1.223	

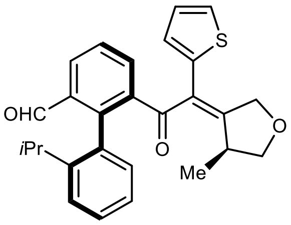

3m

(*S*)-2'-isopropyl-6-((*Z*)-2-((*S*)-4-methyldihydrofuran-3(2H)-ylidene)-2-(thiophen-2-yl)acetyl)-[1,1'-biphenyl]-2-carbaldehyde (3m)

The general procedure was followed using bialdehyde **1a** (0.11 mmol, 27.8 mg) and 1,6-enyne **2m** (0.10 mmol, 17.8 mg) at 40 °C for 24 h. Purification by column chromatography on silica gel (DCM) yielded **3m** (30.5 mg, 71%, >20:1 d.r., >99% e.e.) as a colorless oil.

^{1}H-NMR (400 MHz, CDCl$_3$) δ 9.61 (d, J = 0.8 Hz, 1H), 8.08 (dd, J = 7.8, 1.4 Hz, 1H), 7.82 (dd, J = 7.7, 1.4 Hz, 1H), 7.48 (td, J = 7.8, 0.8 Hz, 1H), 7.44 – 7.39 (m, 2H), 7.26 – 7.24 (m, 1H), 7.21 – 7.16 (m, 1H), 6.99 (dt, J = 7.4, 1.0 Hz, 1H), 6.93 (dd, J = 5.1, 3.6 Hz, 1H), 6.67 (dd, J = 3.7, 1.1 Hz, 1H), 4.62 (dd, J = 15.8, 1.8 Hz, 1H), 4.33 (d, J = 15.9 Hz, 1H), 3.74 (dd, J = 8.5, 5.7 Hz, 1H), 3.63 (dd, J = 8.5, 2.7 Hz, 1H), 3.05 – 2.93 (m, 1H), 2.58 (p, J = 6.8 Hz, 1H), 1.25 (d, J = 6.8 Hz, 3H), 1.08 (d, J = 7.0 Hz, 3H), 0.99 (d, J = 6.9 Hz, 3H).

^{13}C-NMR (101 MHz, CDCl$_3$) δ 195.0 (C$_q$), 191.7 (CH), 155.2 (C$_q$), 148.3 (C$_q$), 144.6 (C$_q$), 138.8 (C$_q$), 137.8 (C$_q$), 135.2 (C$_q$), 134.8 (CH), 133.6 (C$_q$), 129.7 (CH), 129.0 (CH), 128.6 (CH), 127.7 (CH), 127.3 (CH), 127.1 (CH), 126.5 (C$_q$), 126.4 (CH), 125.5 (CH), 125.1 (CH), 75.6 (CH$_2$), 71.1 (CH$_2$), 37.9 (CH), 30.9 (CH), 24.2 (CH$_3$), 23.0 (CH$_3$), 18.7 (CH$_3$).

IR (ATR): 1762, 1691, 1585, 1490, 1247, 1176, 1093, 1066, 1033, 762 cm^{-1}.

HR-MS (ESI): m/z calcd. for $[C_{27}H_{26}O_3S + Na]^+$ 453.1495, found 453.1489.

[α_D^{20}] = +101.5 (c = 1.0, DCM).

HPLC separation (Chiralpak® IA-3, *n*-hexane/*i*-PrOH 99:1, 1.0 mL/min, detection at 250 nm): t_r (major) = 12.7 min, >99% e.e.

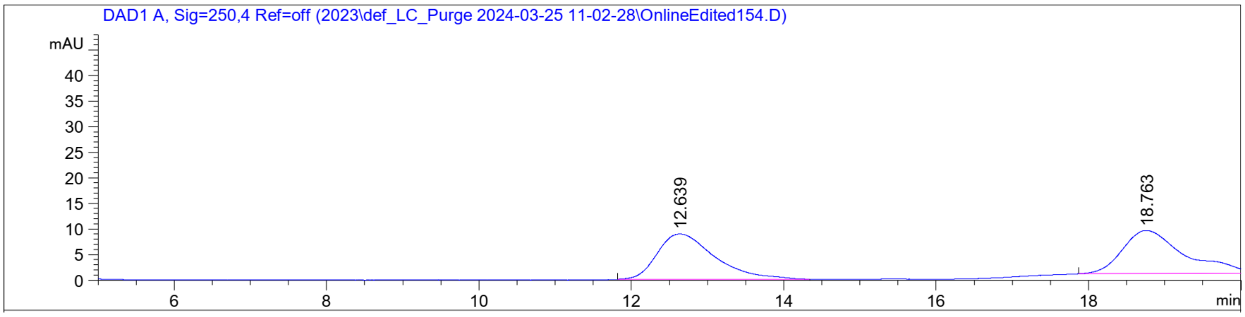

Peak #	RetTime [min]	Type	Width [min]	Area [mAU*s]	Height [mAU]	Area %
1	12.639	BB	0.6151	461.51724	8.83977	49.3417
2	18.763	BB	0.6617	473.83176	8.41760	50.6583

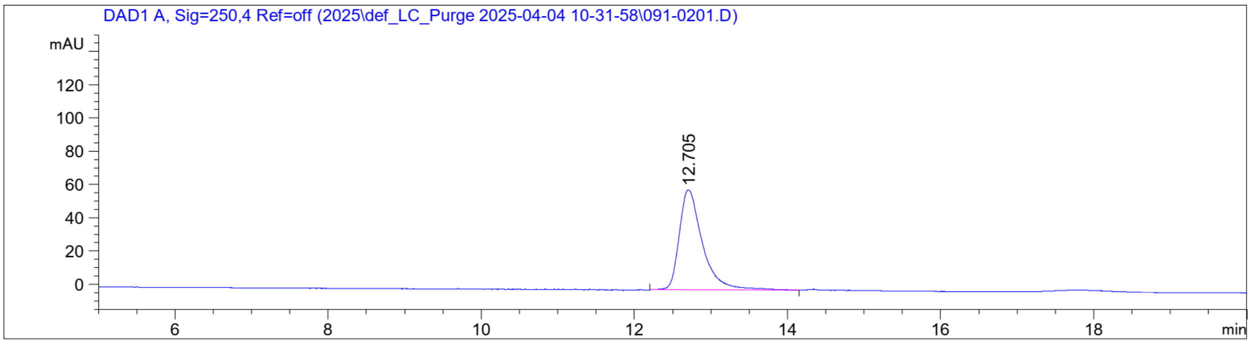

Peak #	RetTime [min]	Type	Width [min]	Area [mAU*s]	Height [mAU]	Area %
1	12.705	MM R	0.3524	1271.96985	60.15712	100.0000

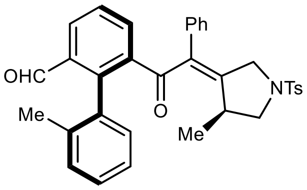

3n

(*S*)-2'-methyl-6-((*E*)-2-((*S*)-4-methyl-1-tosylpyrrolidin-3-ylidene)-2-phenylacetyl)-[1,1'-biphenyl]-2-carbaldehyde (3n)

The general procedure was followed using bialdehyde **1b** (0.11 mmol, 24.6 mg) and 1,6-enyne **2a** (0.10 mmol, 32.5 mg) at 40 °C for 24 h. Purification by column chromatography on silica gel (DCM) yielded **3n** (36.8 mg, 67%, 13:1 d.r., 99% e.e.) as a colorless oil.

^{1}H-NMR (300 MHz, $CDCl_3$) δ 9.49 (d, *J* = 0.8 Hz, 1H), 7.96 (dd, *J* = 7.8, 1.5 Hz, 1H), 7.58 (ddd, *J* = 8.8, 7.2, 1.6 Hz, 3H), 7.36 (dd, *J* = 7.7, 0.9 Hz, 1H), 7.34 – 7.29 (m, 2H), 7.24 (ddt, *J* = 6.7, 5.7, 1.2 Hz, 5H), 7.09 – 7.02 (m, 1H), 6.91 – 6.83 (m, 3H), 4.00 (dd, *J* = 16.5, 1.6 Hz, 1H), 3.42 (d, *J* = 16.5 Hz, 1H), 3.19 – 3.05 (m, 2H), 3.00 (dd, *J* = 9.0, 6.1 Hz, 1H), 2.44 (s, 3H), 1.95 (s, 3H), 1.11 (d, *J* = 6.8 Hz, 3H).

^{13}C-NMR (75 MHz, $CDCl_3$) δ 195.8 (C_q), 191.5 (CH), 152.1 (C_q), 143.9 (C_q), 143.5 (C_q), 139.8 (C_q), 137.9 (C_q), 136.0 (C_q), 134.9 (C_q), 134.7 (C_q), 134.4 (C_q), 133.6 (CH), 132.3 (C_q), 129.7 (CH), 129.1 (CH), 128.9 (CH), 128.8 (CH), 128.7 (CH), 128.6 (CH), 128.1 (CH), 127.8 (CH),

127.8 (CH), 127.4 (CH), 125.2 (CH), 54.9 (CH_2), 51.2 (CH_2), 36.2 (C_q), 21.6 (CH_3), 20.5 (CH_3), 19.8 (CH_3).

IR (ATR): 1691, 1452, 1343, 1305, 1237, 1161, 1092, 814, 765, 666 cm^{-1}.

HR-MS (ESI): *m*/*z* calcd. for $[C_{34}H_{31}NO_4S + Na]^+$ 572.1866, found 572.1867.

$[\alpha_D^{20}]$ = +123.8 (c = 1.0, DCM).

HPLC separation (Chiralpak® IA-3, *n*-hexane/*i*-PrOH 95:5, 1.0 mL/min, detection at 250 nm): t_r (minor) = 31.2 min, t_r (major) = 33.4 min, 99% e.e.

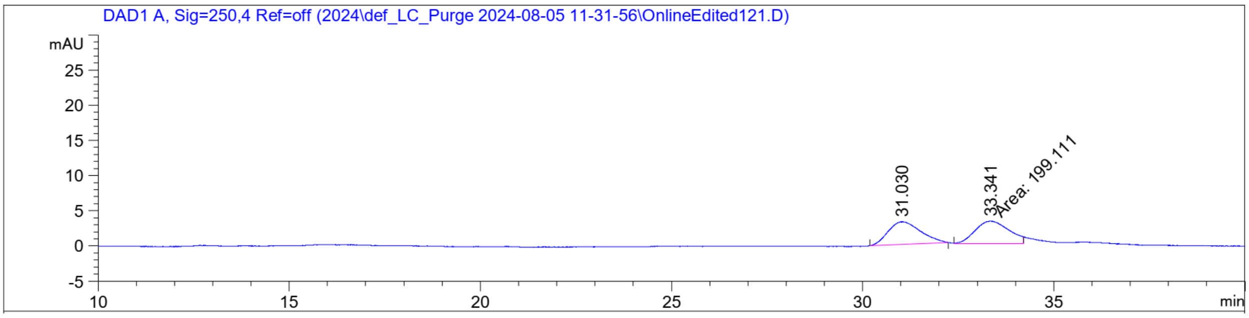

Peak #	RetTime [min]	Type	Width [min]	Area [mAU*s]	Height [mAU]	Area %
1	31.030	BB	0.6935	190.25352	3.22394	48.8626
2	33.341	MF	1.0331	199.11050	3.21226	51.1374

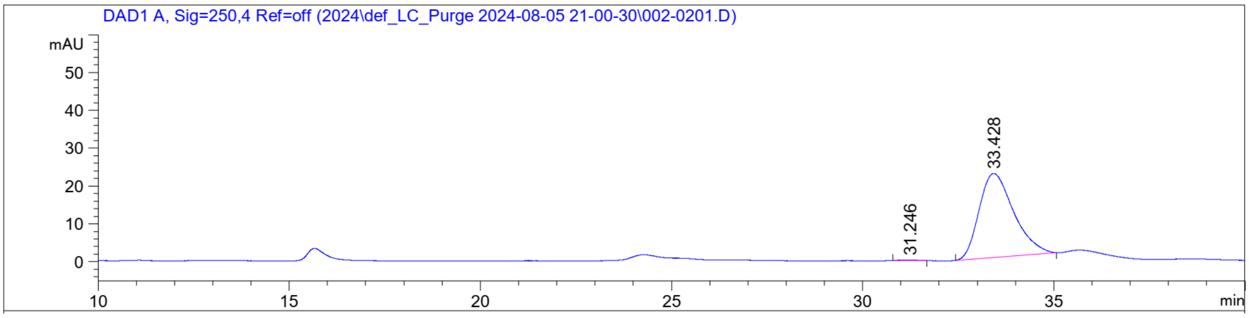

Peak #	RetTime [min]	Type	Width [min]	Area [mAU*s]	Height [mAU]	Area %
1	31.246	MM R	0.6008	6.49366	1.80143e-1	0.4637
2	33.428	BB	0.7390	1393.83813	22.18489	99.5363

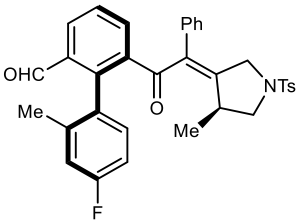

3o

(*S*)-4'-fluoro-2'-methyl-6-((*E*)-2-((*S*)-4-methyl-1-tosylpyrrolidin-3-ylidene)-2-phenylacetyl)-[1,1'-biphenyl]-2-carbaldehyde (3o)

The general procedure was followed using bialdehyde **1c** (0.11 mmol, 24.2 mg) and 1,6-enyne **2a** (0.10 mmol, 32.5 mg) at room temperature for 48 h. Purification by column chromatography on silica gel (DCM) yielded **3o** (35.2 mg, 62%, 17:1 d.r., >99% e.e.) as a colorless oil.

^{1}H-NMR (300 MHz, CDCl$_3$) δ 9.43 (s, 1H), 7.89 (dd, *J* = 7.8, 1.5 Hz, 1H), 7.57 – 7.48 (m, 3H), 7.33 (t, *J* = 7.7 Hz, 1H), 7.25 – 7.16 (m, 5H), 6.91 – 6.85 (m, 1H), 6.85 – 6.78 (m, 2H), 6.71 (dd, *J* = 7.5, 1.9 Hz, 2H), 3.96 (dd, *J* = 16.5, 1.6 Hz, 1H), 3.36 (d, *J* = 16.5 Hz, 1H), 3.21 – 3.07 (m, 2H), 2.99 (dd, *J* = 9.5, 6.5 Hz, 1H), 2.36 (s, 3H), 1.88 (s, 3H), 1.04 (d, *J* = 6.9 Hz, 3H).

^{13}C-NMR (101 MHz, CDCl$_3$) δ 195.6 (C_q), 191.2 (CH), 162.6 (d, *J* = 247.6 Hz, C_q), 152.3(C_q), 143.9 (C_q), 142.6 (C_q), 140.7 (C_q), 140.6 (C_q), 139.7 (C_q), 135.9 (C_q), 134.8 (d, *J* = 8.9 Hz, C_q), 133.7 (C_q), 132.2 (C_q), 130.5 (d, *J* = 3.4 Hz, CH), 130.1 (d, *J* = 8.1 Hz, CH), 129.7 (CH), 129.4 (CH), 128.8 (CH), 128.6 (CH), 128.2 (CH), 127.8 (CH), 127.6 (CH), 116.5 (d, *J* = 21.2 Hz, CH), 112.1 (d, *J* = 21.4 Hz, CH), 54.8 (CH_2), 51.2 (CH_2), 36.1 (CH), 21.5 (CH_3), 20.6 (CH_3), 19.7 (CH_3).

^{19}F-NMR (282 MHz, CDCl$_3$) δ -113.73.

IR (ATR): 1694, 1587, 1455, 1348, 1276, 1222, 162, 1092, 815, 732, 664 cm^{-1}.

HR-MS (ESI): *m*/*z* calcd. for $[C_{34}H_{30}FNO_4S + Na]^+$ 590.1772, found 590.1774.

$[\alpha_D^{20}]$ = +60.1 (c = 1.0, DCM).

HPLC separation (Chiralpak® IA-3, *n*-hexane/*i*-PrOH 98:2, 1.0 mL/min, detection at 250 nm): t_r (major) = 38.3 min, >99% e.e.

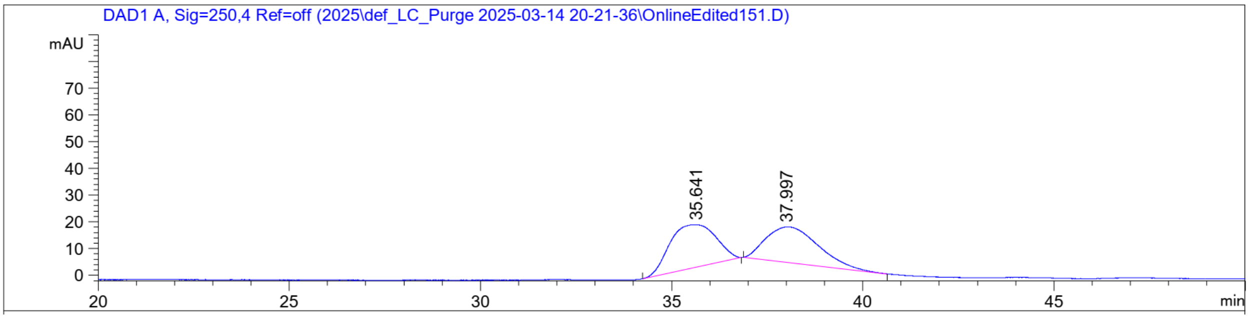

Peak #	RetTime [min]	Type	Width [min]	Area [mAU*s]	Height [mAU]	Area %
1	35.641	BB	0.9977	1357.02332	15.91656	51.4817
2	37.997	BB	1.1310	1278.91174	13.25126	48.5183

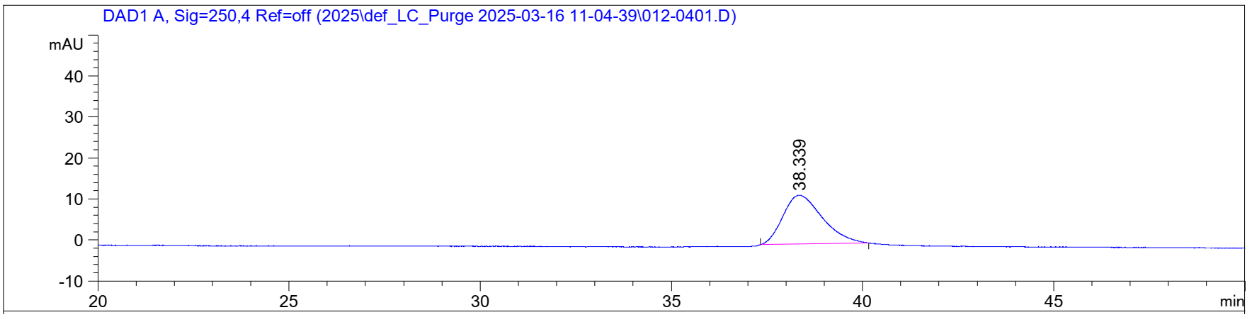

Peak #	RetTime [min]	Type	Width [min]	Area [mAU*s]	Height [mAU]	Area %
1	38.339	BB	0.8433	856.62744	11.90697	100.0000

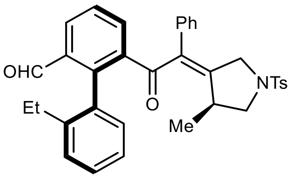

3p

(*S*)-2'-ethyl-6-((*E*)-2-((*S*)-4-methyl-1-tosylpyrrolidin-3-ylidene)-2-phenylacetyl)-[1,1'-biphenyl]-2-carbaldehyde (3p)

The general procedure was followed using bialdehyde **1d** (0.11 mmol, 26.3 mg) and 1,6-enyne **2a** (0.10 mmol, 32.5 mg) at 40 °C for 24 h. Purification by column chromatography on silica gel (DCM) yielded **3p** (37.1 mg, 66%, >20:1 d.r., 99% e.e.) as a colorless oil.

^{1}H-NMR (400 MHz, $CDCl_3$) δ 9.51 (d, J = 0.8 Hz, 1H), 7.96 (dd, J = 7.8, 1.4 Hz, 1H), 7.63 – 7.54 (m, 3H), 7.38 (td, J = 7.8, 0.9 Hz, 1H), 7.32 (d, J = 8.0 Hz, 2H), 7.29 – 7.26 (m, 3H), 7.25 – 7.20 (m, 2H), 7.04 (ddd, J = 7.5, 5.4, 3.3 Hz, 1H), 6.91 – 6.87 (m, 2H), 6.82 – 6.78 (m, 1H), 3.99 (dd, J = 16.3, 1.6 Hz, 1H), 3.42 (d, J = 16.4 Hz, 1H), 3.15 (dd, J = 9.0, 1.8 Hz, 1H), 3.08 – 2.92 (m, 2H), 2.44 (s, 3H), 2.30 (ddt, J = 41.0, 15.0, 7.5 Hz, 2H), 1.11 (d, J = 6.8 Hz, 3H), 1.00 (t, J = 7.6 Hz, 3H).

^{13}C-NMR (101 MHz, $CDCl_3$) δ 195.9 (C_q), 191.6 (CH), 151.3 (C_q), 143.9 (C_q), 143.5 (C_q), 143.5 (C_q), 139.6 (C_q), 135.9 (C_q), 135.1 (C_q), 134.7 (C_q), 134.1 (C_q), 133.7 (CH), 132.3 (C_q), 129.7 (CH), 129.1 (CH), 129.0 (CH), 128.8 (2C, CH), 128.6 (CH), 128.1 (CH), 127.8 (CH), 127.7 (CH),

127.4 (CH), 125.0 (CH), 54.9 (CH_2), 51.0 (CH_2), 36.3 (CH), 26.5 (CH_2), 21.6 (CH_3), 20.0 (CH_3), 14.0 (CH_3).

IR (ATR): 1693, 1455, 1347, 1236, 1162, 1092, 1051, 815, 762 cm^{-1}.

HR-MS (ESI): *m*/*z* calcd. for $[C_{35}H_{33}NO_4S + Na]^+$ 586.2023, found 586.2022.

$[\alpha^{20}_{D}]$ = +57.9 (c = 1.0, DCM).

HPLC separation (Chiralpak® IF-3, *n*-hexane/*i*-PrOH 90:10, 1.0 mL/min, detection at 250 nm): t_r (major) = 28.3 min, t_r (minor) = 30.8 min, 99% e.e.

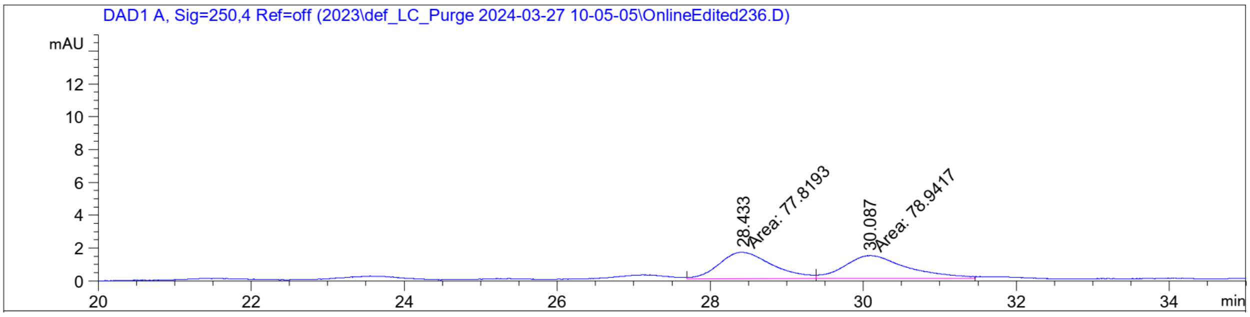

Peak #	RetTime [min]	Type	Width [min]	Area [mAU*s]	Height [mAU]	Area %
1	28.433	MF	0.8083	77.81926	1.60454	49.6420
2	30.087	FM	0.9461	78.94172	1.39071	50.3580

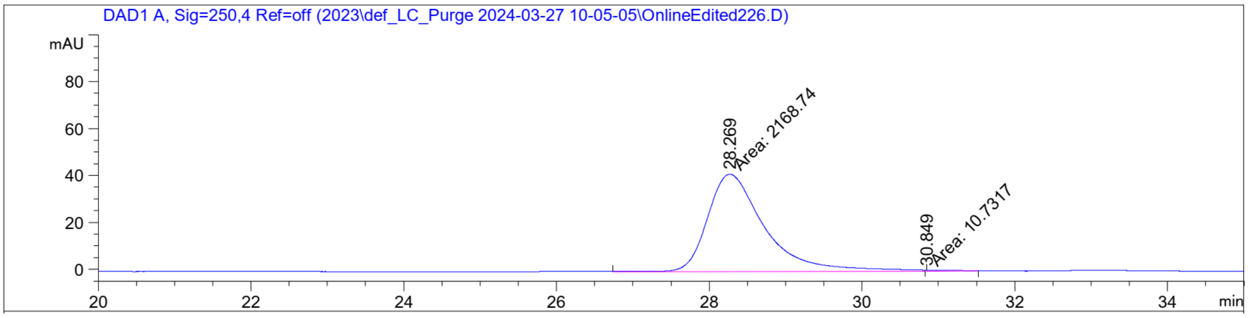

Peak #	RetTime [min]	Type	Width [min]	Area [mAU*s]	Height [mAU]	Area %
1	28.269	MF	0.8709	2168.74414	41.50396	99.5076
2	30.849	MM	0.2898	10.73172	4.39372e-1	0.4924

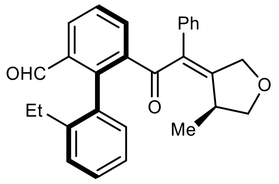

3q

(*S*)-2'-ethyl-6-((*E*)-2-((*S*)-4-methyldihydrofuran-3(2H)-ylidene)-2-phenylacetyl)-[1,1'-biphenyl]-2-carbaldehyde (3q)

The general procedure was followed using bialdehyde **1d** (0.11 mmol, 26.3 mg) and 1,6-enyne **2f** (0.10 mmol, 17.2 mg) at 40 °C for 24 h. Purification by column chromatography on silica gel (DCM) yielded **3q** (27.9 mg, 68%, >20:1 d.r., >99% e.e.) as a colorless oil.

^{1}H-NMR (400 MHz, $CDCl_3$) δ 9.59 (d, *J* = 0.8 Hz, 1H), 8.04 (dd, *J* = 7.8, 1.4 Hz, 1H), 7.75 (dd, *J* = 7.7, 1.4 Hz, 1H), 7.49 – 7.38 (m, 3H), 7.32 – 7.23 (m, 4H), 7.03 – 6.99 (m, 2H), 6.94 (dd, *J* = 7.5, 1.3 Hz, 1H), 4.46 (dd, *J* = 15.7, 1.7 Hz, 1H), 4.08 (d, *J* = 15.7 Hz, 1H), 3.84 (dd, *J* = 8.5, 5.9 Hz, 1H), 3.74 – 3.57 (m, 1H), 3.23 – 3.09 (m, 1H), 2.43 (ddq, *J* = 56.9, 15.0, 7.6 Hz, 2H), 1.19 (d, *J* = 7.0 Hz, 3H), 1.12 (t, *J* = 7.6 Hz, 3H).

^{13}C-NMR (101 MHz, $CDCl_3$) δ 196.1 (C_q), 191.8 (CH), 156.4 (C_q), 143.8 (C_q), 143.6 (C_q), 140.0 (C_q), 136.8 (C_q), 134.8 (C_q), 134.3 (C_q), 134.0 (CH), 132.7 (C_q), 129.1 (CH), 129.0 (CH), 128.8 (CH), 128.7 (CH), 128.6 (CH), 127.8 (CH), 127.7 (CH), 127.4 (CH), 125.2 (CH), 75.7 (CH_2), 70.7 (CH_2), 37.3 (CH), 26.5 (CH_2), 18.7 (CH_3), 14.1 (CH_3).

IR (ATR): 1691, 1572, 1455, 1386, 1236, 1045, 1072, 1045, 761, 702 cm^{-1}.

HR-MS (ESI): *m*/*z* calcd. for $[C_{28}H_{26}O_3 + Na]^+$ 433.1774, found 433.1764.

$[\alpha^{20}_{D}]$ = +76.9 (c = 1.0, DCM).

HPLC separation (Chiralpak® ID-3, *n*-hexane/*i*-PrOH 90:10, 1.0 mL/min, detection at 250 nm): t_r (major) = 12.4 min, >99% e.e.

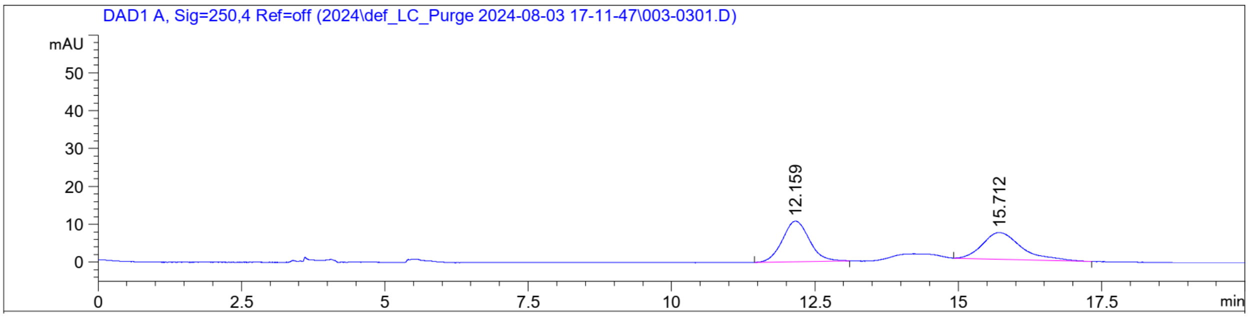

Peak #	RetTime [min]	Type	Width [min]	Area [mAU*s]	Height [mAU]	Area %
1	12.159	BB	0.3984	350.90060	10.70508	51.2907
2	15.712	BB	0.5540	333.24008	7.04322	48.7093

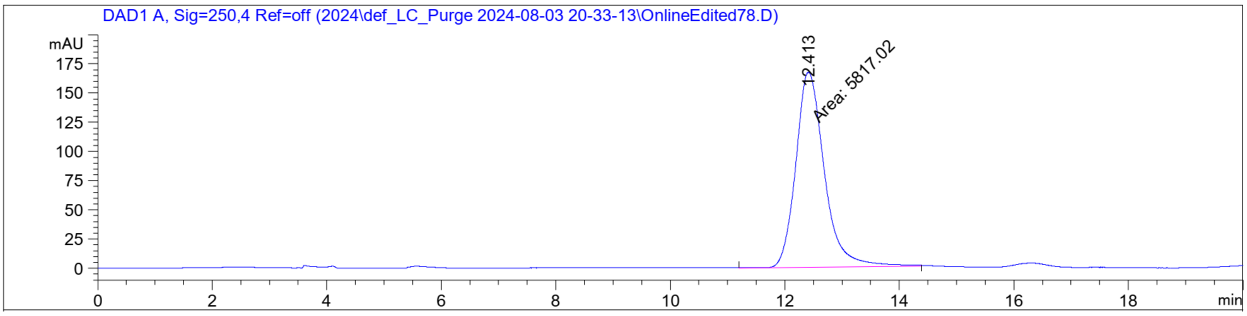

Peak #	RetTime [min]	Type	Width [min]	Area [mAU*s]	Height [mAU]	Area %
1	12.413	MM	0.5800	5817.02295	167.16225	100.0000

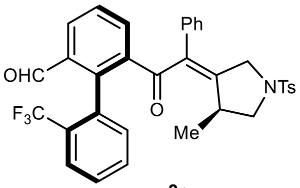

3r

(*S*)-6-((*E*)-2-((*S*)-4-methyl-1-tosylpyrrolidin-3-ylidene)-2-phenylacetyl)-2'-(trifluoromethyl)-[1,1'-biphenyl]-2-carbaldehyde (3r)

The general procedure was followed using bialdehyde **1e** (0.11 mmol, 30.6 mg) and 1,6-enyne **2a** (0.10 mmol, 32.5 mg) at 40 °C for 24 h. Purification by column chromatography on silica gel (DCM) yielded **3r** (45.3 mg, 75%, 18:1 d.r., >99% e.e.) as a white solid.

^{1}H-NMR (500 MHz, $CDCl_3$) δ 9.49 (s, 1H), 8.04 (dd, *J* = 7.8, 1.4 Hz, 1H), 7.86 (dd, *J* = 7.8, 1.4 Hz, 1H), 7.78 (dd, *J* = 5.5, 3.7 Hz, 1H), 7.61 – 7.57 (m, 2H), 7.55 – 7.51 (m, 2H), 7.45 (td, *J* = 7.8, 0.8 Hz, 1H), 7.31 – 7.28 (m, 2H), 7.28 – 7.24 (m, 3H), 7.03 – 6.98 (m, 3H), 4.18 (dd, *J* = 15.8, 1.4 Hz, 1H), 3.57 (d, *J* = 15.8 Hz, 1H), 3.15 (dddd, *J* = 7.9, 6.4, 4.3, 1.9 Hz, 2H), 3.12 – 3.05 (m, 1H), 2.39 (s, 3H), 1.00 (d, *J* = 7.0 Hz, 3H).

^{13}C-NMR (126 MHz, $CDCl_3$) δ 194.4 (C_q), 190.2 (CH), 147.9 (C_q), 143.9 (C_q), 142.3 (C_q), 136.3 (C_q), 135.9 (CH), 135.8 (C_q), 135.1 (C_q), 135.1 (C_q), 134.8 (q, *J* = 2.2 Hz, C_q), 132.4 (C_q), 131.4 (CH), 130.5 (CH), 129.9 (q, *J* = 30.1 Hz, C_q), 129.7 (CH), 129.4 (CH), 129.0 (CH), 128.4 (CH), 128.3 (CH), 128.3 (CH), 128.0 (CH), 127.7 (CH), 126.1 (q, *J* = 4.9 Hz, CH), 123.8 (q, *J* = 273.9 Hz, C_q), 54.5 (CH_2), 50.7 (CH_2), 35.8 (CH), 21.5 (CH_3), 19.2 (CH_3).

^{19}F-NMR (282 MHz, $CDCl_3$) δ -59.33.

IR (ATR): 1697, 1678, 1572, 1442, 1345, 1314, 1161, 1123, 1091, 769 cm^{-1}.

HR-MS (ESI): *m*/*z* calcd. for $[C_{34}H_{28}F_3NO_4S + Na]^+$ 626.1583, found 626.1579.

$[\alpha^{20}_{D}]$ = +93.2 (c = 1.0, DCM).

HPLC separation (Chiralpak® IF-3, *n*-hexane/*i*-PrOH 80:20, 1.0 mL/min, detection at 273 nm): t_r (major) = 17.3 min, t_r (minor) = 37.3 min, >99% e.e.

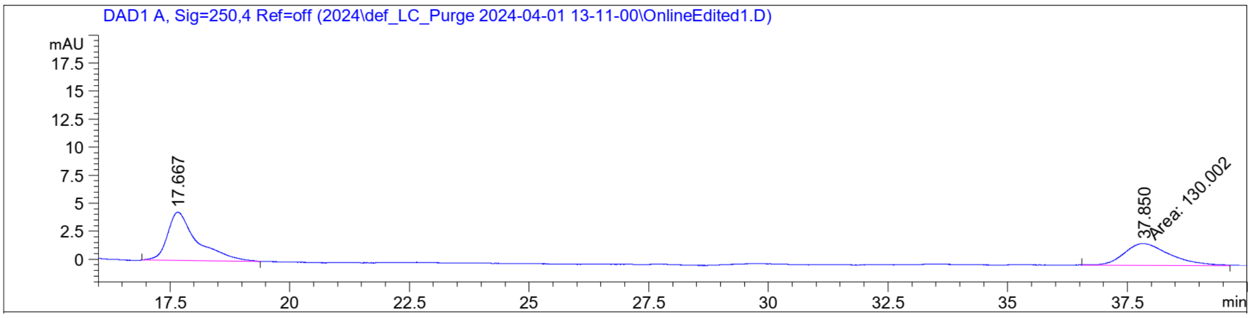

Peak #	RetTime [min]	Type	Width [min]	Area [mAU*s]	Height [mAU]	Area %
1	17.667	BB	0.5059	185.27078	4.31537	58.7652
2	37.850	MM	1.1178	130.00237	1.93842	41.2348

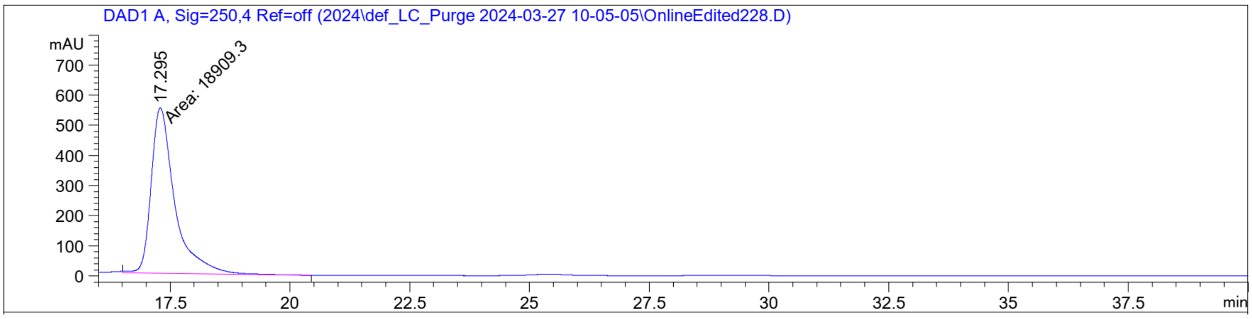

Peak #	RetTime [min]	Type	Width [min]	Area [mAU*s]	Height [mAU]	Area %
1	17.295	FM	0.5734	1.89093e4	549.62604	100.0000

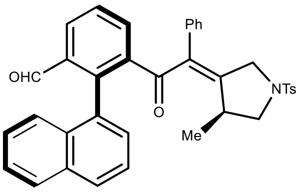

3s

3-((*E*)-2-((*S*)-4-methyl-1-tosylpyrrolidin-3-ylidene)-2-phenylacetyl)-2-(naphthalen-1-yl)benzaldehyde (3s)

The general procedure was followed using bialdehyde **1f** (0.11 mmol, 28.6 mg) and 1,6-enyne **2a** (0.10 mmol, 32.5 mg) at room temperature for 48 h. Purification by column chromatography on silica gel (DCM) yielded **3s** (46.3 mg, 79%, 12:1 d.r., >99% e.e.) as a colorless oil.

^{1}H-NMR (300 MHz, CDCl$_3$) δ 9.29 (s, 1H), 8.05 (dd, J = 7.8, 1.4 Hz, 1H), 7.82 (d, J = 8.2 Hz, 1H), 7.77 – 7.70 (m, 2H), 7.59 – 7.49 (m, 3H), 7.43 – 7.29 (m, 4H), 7.17 (ddt, J = 8.9, 5.8, 2.6 Hz, 5H), 7.09 (dd, J = 7.0, 1.2 Hz, 1H), 6.66 – 6.54 (m, 2H), 3.72 (dd, J = 16.4, 1.5 Hz, 1H), 3.15 – 3.02 (m, 2H), 2.67 (dd, J = 9.2, 6.1 Hz, 1H), 2.50 (s, 3H), 2.48 – 2.39 (m, 1H), 1.02 (d, J = 7.0 Hz, 3H).

^{13}C-NMR (75 MHz, CDCl$_3$) δ 196.4 (C$_q$), 191.2 (CH), 152.5 (C$_q$), 143.8 (C$_q$), 141.9 (C$_q$), 141.2 (C$_q$), 135.7 (C$_q$), 135.2 (C$_q$), 134.5 (C$_q$), 133.9 (CH), 133.0 (C$_q$), 133.0 (C$_q$), 132.5 (C$_q$), 132.2 (C$_q$), 129.7 (CH), 129.2 (CH), 129.1 (CH), 128.6 (CH), 128.5 (CH), 128.4 (CH), 128.3 (CH), 128.1 (CH), 127.91 (CH), 127.90 (CH), 127.0 (CH), 126.4 (CH), 125.9 (CH), 124.7 (CH), 54.9 (CH$_2$), 51.2 (CH$_2$), 36.5 (CH), 21.6 (CH$_3$), 20.4 (CH$_3$).

IR (ATR): 1691, 1597, 1454, 1347, 1237, 1159, 1090, 1042, 733, 633 cm^{-1}.

HR-MS (ESI): m/z calcd. for $[C_{37}H_{31}NO_4S + Na]^+$ 608.1866, found 608.1869.

$[\alpha_D^{20}]$ = +68.3 (c = 1.0, DCM).

HPLC separation (Chiralpak® IE-3, n-hexane/i-PrOH 60:40, 1.0 mL/min, detection at 273 nm): t_r (major) = 24.8 min, >99% e.e.

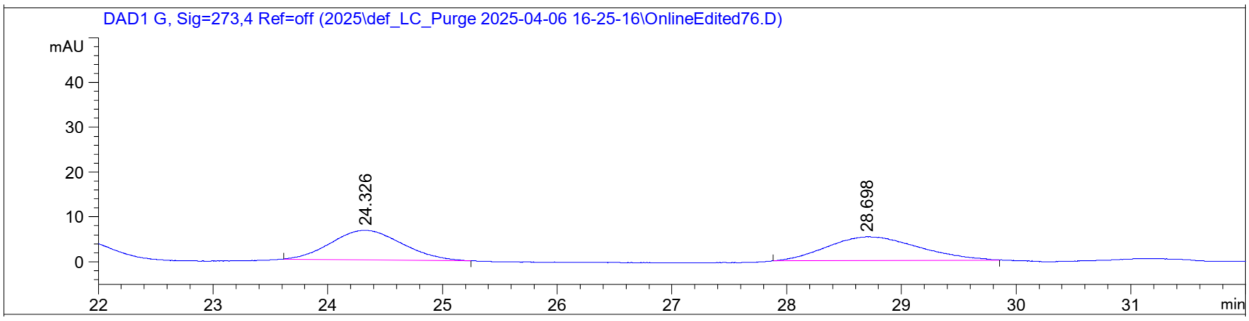

Peak #	RetTime [min]	Type	Width [min]	Area [mAU*s]	Height [mAU]	Area %
1	24.326	BB	0.5013	281.96918	6.60198	48.8831
2	28.698	BB	0.6520	294.85452	5.31662	51.1169

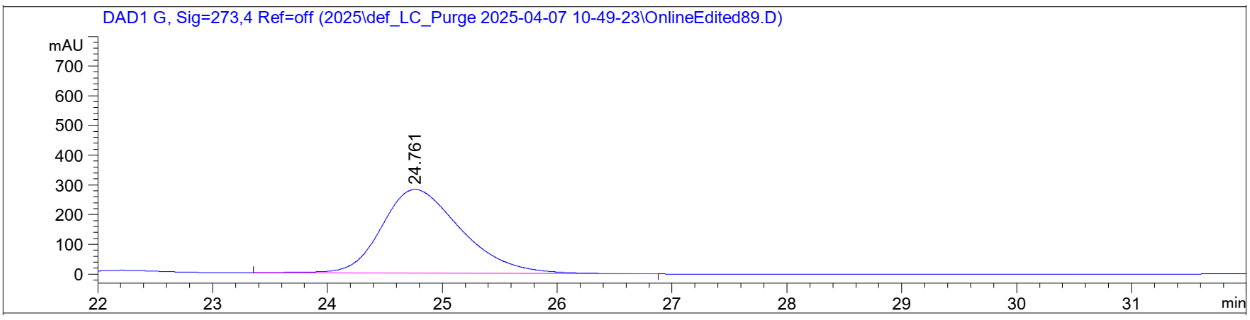

Peak #	RetTime [min]	Type	Width [min]	Area [mAU*s]	Height [mAU]	Area %
1	24.761	BB	0.7015	1.36152e4	281.86774	100.0000

The construction of 1,6-central/C–O axial chirality

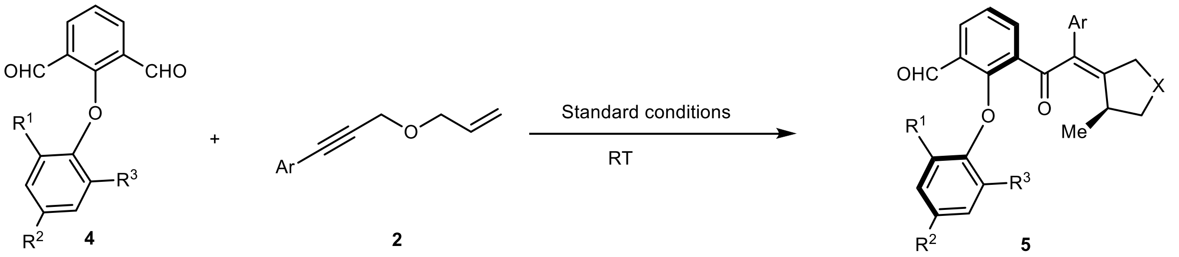

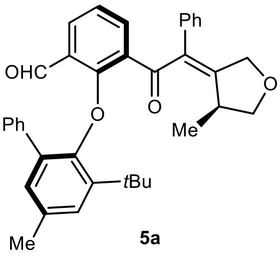

5a

(*S*,*E*)-2-((3-(*tert*-butyl)-5-methyl-[1,1'-biphenyl]-2-yl)oxy)-3-(2-(4-methyldihydrofuran-3(2H)-ylidene)-2-phenylacetyl)benzaldehyde (5a)

The general procedure was followed using bialdehyde **4a** (0.11 mmol, 40.1 mg) and 1,6-enyne **2f** (0.10 mmol, 17.2 mg) at room temperature for 72 h. Purification by column chromatography on silica gel (DCM) yielded **5a** (32.1 mg, 59%, 15:1 d.r., 99% e.e.) as a colorless oil.

^{1}H-NMR (400 MHz, $CDCl_3$) δ 9.32 (s, 1H), 7.40 – 7.34 (m, 4H), 7.26 – 7.24 (m, 2H), 7.21 – 7.18 (m, 1H), 7.17 – 7.14 (m, 2H), 7.01 – 6.91 (m, 5H), 6.64 – 6.60 (m, 1H), 4.64 (dd, *J* = 15.2, 1.8 Hz, 1H), 4.26 (d, *J* = 15.1 Hz, 1H), 4.05 (dd, *J* = 8.6, 6.5 Hz, 1H), 3.66 (dd, *J* = 8.6, 4.5 Hz, 1H), 3.50 – 3.41 (m, 1H), 2.37 (s, 3H), 1.53 (s, 9H), 1.26 (d, *J* = 7.0 Hz, 3H).

^{13}C-NMR (101 MHz, CDCl$_3$) δ 194.0 (C_q), 187.3 (CH), 157.6 (C_q), 153.4 (C_q), 153.3 (C_q), 140.2 (C_q), 137.9 (C_q), 137.3 (C_q), 136.4 (CH), 134.4 (C_q), 133.5 (C_q), 132.6 (CH), 131.9 (CH), 131.0 (C_q), 129.5 (CH), 128.8 (CH), 128.7 (C_q), 128.5 (CH), 128.4 (CH), 127.9 (CH), 127.7 (CH), 127.1 (CH), 126.8 (C_q), 121.2 (CH), 75.7 (CH_2), 70.8 (CH_2), 37.2 (CH), 35.5 (C_q), 30.5 (CH_3), 21.3 (CH_3), 18.2 (CH_3).

IR (ATR): 1680, 1586, 1494, 1396, 1278, 1223, 1127, 1099, 701 cm^{-1}.

HR-MS (ESI): *m*/*z* calcd. for $[C_{37}H_{36}O_4 + Na]^+$ 567.2506, found 567.2501.

[α_D^{20}] = +113.0 (c = 1.0, DCM).

HPLC separation (Chiralpak® IE-3, *n*-hexane/*i*-PrOH 97:3, 1.0 mL/min, detection at 273 nm): t_r (major) = 11.3 min, t_r (minor) = 12.8 min, 99% e.e.

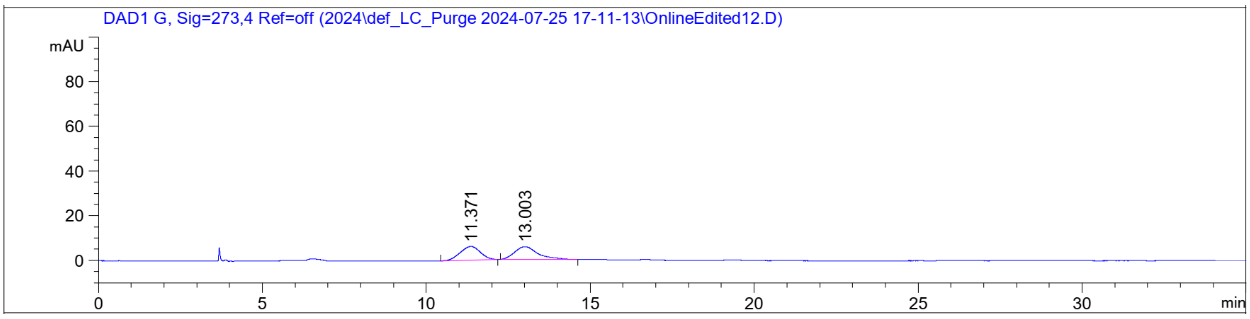

Peak #	RetTime [min]	Type	Width [min]	Area [mAU*s]	Height [mAU]	Area %
1	11.371	BB	0.5013	261.54376	6.13643	47.9897
2	13.003	BB	0.5831	283.45645	5.73076	52.0103

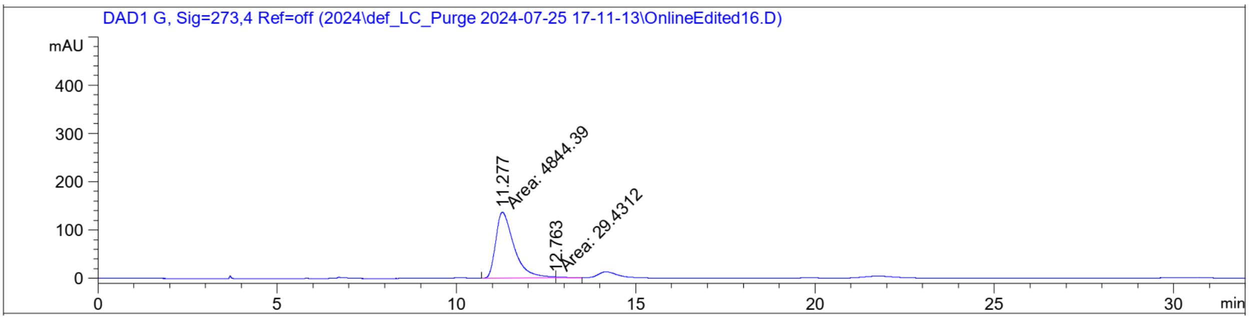

Peak #	RetTime [min]	Type	Width [min]	Area [mAU*s]	Height [mAU]	Area %
1	11.277	MF	0.5919	4844.39258	136.39786	99.3961
2	12.763	FM	0.2589	29.43119	1.89467	0.6039

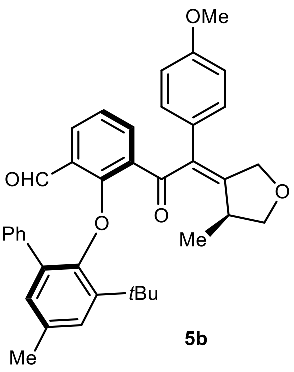

5b

(*S,E*)-2-((3-(*tert*-butyl)-5-methyl-[1,1'-biphenyl]-2-yl)oxy)-3-(2-(4-methoxyphenyl)-2-(4-methyldihydrofuran-3(2H)-ylidene)acetyl)benzaldehyde (5b)

The general procedure was followed using bialdehyde **4a** (0.11 mmol, 40.1 mg) and 1,6-enyne **2h** (0.10 mmol, 20.2 mg) at room temperature for 72 h. Purification by column chromatography on silica gel (DCM) yielded **5b** (30.0 mg, 52%, 10:1 d.r., 99% e.e.) as a colorless oil.

^{1}H-NMR (300 MHz, $CDCl_3$) δ 9.25 (s, 1H), 7.35 – 7.25 (m, 4H), 7.03 – 6.96 (m, 3H), 6.92 – 6.85 (m, 4H), 6.73 – 6.68 (m, 2H), 6.55 (td, *J* = 7.7, 0.8 Hz, 1H), 4.57 (dd, *J* = 15.0, 1.9 Hz, 1H), 4.20 (d, *J* = 15.0 Hz, 1H), 3.97 (dd, *J* = 8.5, 6.5 Hz, 1H), 3.68 (s, 3H), 3.58 (dd, *J* = 8.6, 4.5 Hz, 1H), 3.36 (q, *J* = 6.5 Hz, 1H), 2.30 (s, 3H), 1.46 (s, 9H), 1.17 (d, *J* = 7.0 Hz, 3H).

^{13}C-NMR (101 MHz, $CDCl_3$) δ 194.3 (C_q), 187.3 (CH), 159.0 (C_q), 157.7 (C_q), 153.4 (C_q), 152.2 (C_q), 140.2 (C_q), 137.9 (C_q), 136.5 (CH), 134.4 (C_q), 133.1 (C_q), 132.6 (CH), 131.9 (CH), 131.0 (C_q), 129.7 (CH), 129.6 (C_q), 129.5 (CH), 128.7 (C_q), 128.4 (CH), 127.7 (CH), 127.1 (CH), 126.8 (C_q), 121.3 (CH), 114.2 (CH), 75.7 (CH_2), 70.8 (CH_2), 55.3 (CH_3), 37.1 (CH), 35.5 (C_q), 30.5 (CH_3), 21.3 (CH_3), 18.2 (CH_3).

IR (ATR): 1681, 1606, 1511, ,1418, 1397, 1248, 1176, 1093, 702 cm^{-1}.

HR-MS (ESI): *m*/*z* calcd. for $[C_{38}H_{38}O_5 + Na]^+$ 597.2611, found 597.2612.

$[\alpha^{20}_{D}]$ = +55.9 (c = 1.0, DCM).

HPLC separation (Chiralpak® IF-3, *n*-hexane/*i*-PrOH 98:2, 1.0 mL/min, detection at 273 nm): t_r (minor) = 14.1 min, t_r (major) = 22.0 min, 99% e.e.

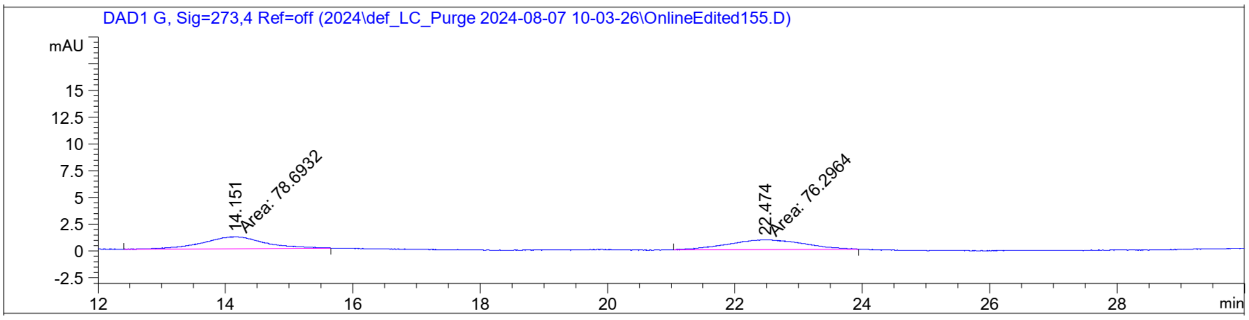

Peak #	RetTime [min]	Type	Width [min]	Area [mAU*s]	Height [mAU]	Area %
1	14.151	MM	1.1667	78.69322	1.12419	50.7732
2	22.474	MM	1.3944	76.29639	9.11954e-1	49.2268

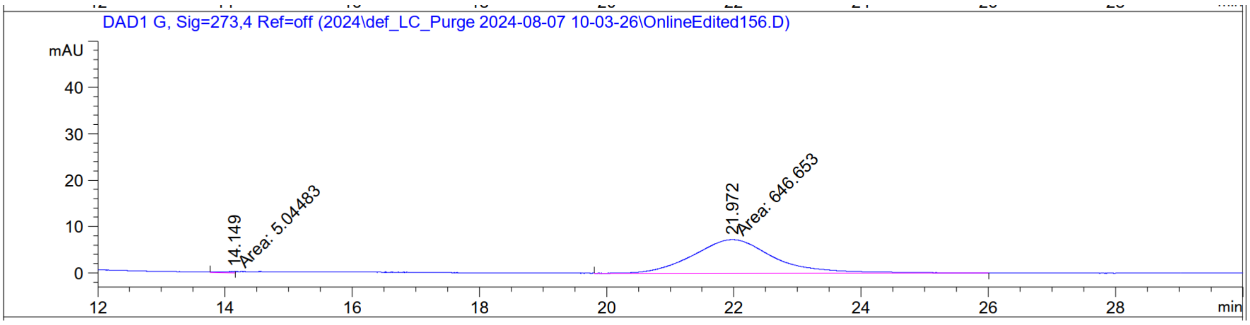

Peak #	RetTime [min]	Type	Width [min]	Area [mAU*s]	Height [mAU]	Area %
1	14.149	FM	0.3329	5.04483	2.52554e-1	0.7741
2	21.972	MM	1.4843	646.65302	7.26084	99.2259

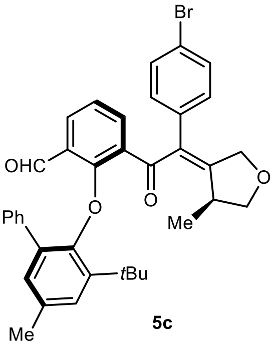

5c

(*S,E*)-3-(2-(4-bromophenyl)-2-(4-methyldihydrofuran-3(2H)-ylidene)acetyl)-2-((3-(*tert*-butyl)-5-methyl-[1,1'-biphenyl]-2-yl)oxy)benzaldehyde (5c)

The general procedure was followed using bialdehyde **4a** (0.11 mmol, 40.1 mg) and 1,6-enyne **2k** (0.10 mmol, 25.1 mg) at room temperature for 72 h. Purification by column chromatography on silica gel (DCM) yielded **5c** (33.1 mg, 53%, 10:1 d.r., >99% e.e.) as a colorless oil.

^{1}H-NMR (400 MHz, CDCl$_3$) δ 9.32 (d, J = 0.9 Hz, 1H), 7.42 – 7.33 (m, 6H), 7.26 – 7.24 (m, 1H), 7.05 – 7.01 (m, 2H), 7.00 – 6.94 (m, 4H), 6.66 – 6.62 (m, 1H), 4.60 (dd, J = 15.2, 1.9 Hz, 1H), 4.23 (d, J = 15.2 Hz, 1H), 4.04 (dd, J = 8.6, 6.5 Hz, 1H), 3.65 (dd, J = 8.6, 4.6 Hz, 1H), 3.48 – 3.39 (m, 1H), 2.37 (s, 3H), 1.52 (s, 9H), 1.24 (d, J = 7.0 Hz, 3H).

^{13}C-NMR (101 MHz, CDCl$_3$) δ 193.5 (C$_q$), 187.2 (CH), 157.7 (C$_q$), 154.1 (C$_q$), 153.4 (C$_q$), 140.2 (C$_q$), 137.8 (C$_q$), 136.3 (C$_q$), 136.2 (CH), 134.5 (C$_q$), 132.9 (CH), 132.4 (C$_q$), 132.0 (CH), 131.9 (CH), 131.0 (C$_q$), 130.1 (CH), 129.4 (CH), 128.4 (CH), 127.7 (CH), 127.1 (CH), 126.9 (C$_q$), 122.0 (C$_q$), 121.3 (CH), 75.7 (CH$_2$), 70.8 (CH$_2$), 37.3 (CH), 35.5 (C$_q$), 30.5 (CH$_3$), 21.3 (CH$_3$), 18.1

(CH_3).

IR (ATR): 1679, 1585, 1436, 1396, 1354, 1277, 1217, 1164, 1126, 1072, 701 cm^{-1}.

HR-MS (ESI): *m*/*z* calcd. for $[C_{37}H_{35}BrO_4 + Na]^+$ 645.1611, found 645.1612.

[α_D^{20}] = +73.8 (c = 1.0, DCM).

HPLC separation (Chiralpak® ID-3, *n*-hexane/*i*-PrOH 90:10, 1.0 mL/min, detection at 273 nm): t_r (major) = 14.8 min, >99% e.e.

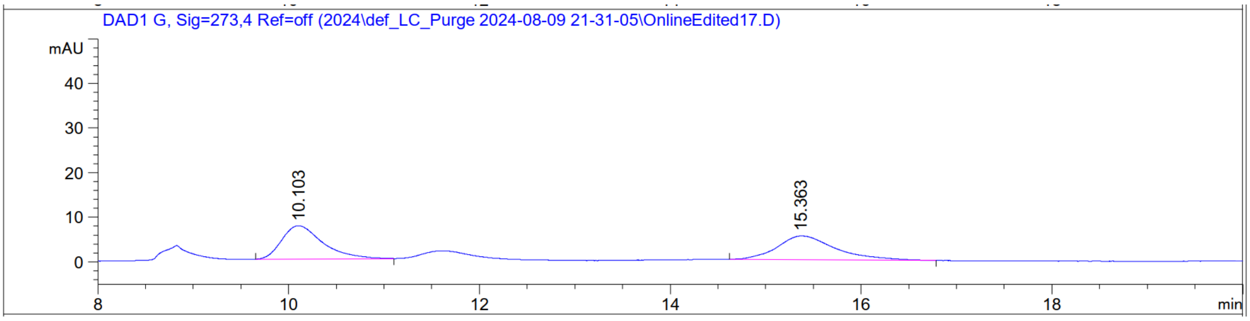

Peak #	RetTime [min]	Type	Width [min]	Area [mAU*s]	Height [mAU]	Area %
1	10.103	BB	0.3541	222.20811	7.44668	49.4804
2	15.363	BB	0.5027	226.87454	5.31792	50.5196

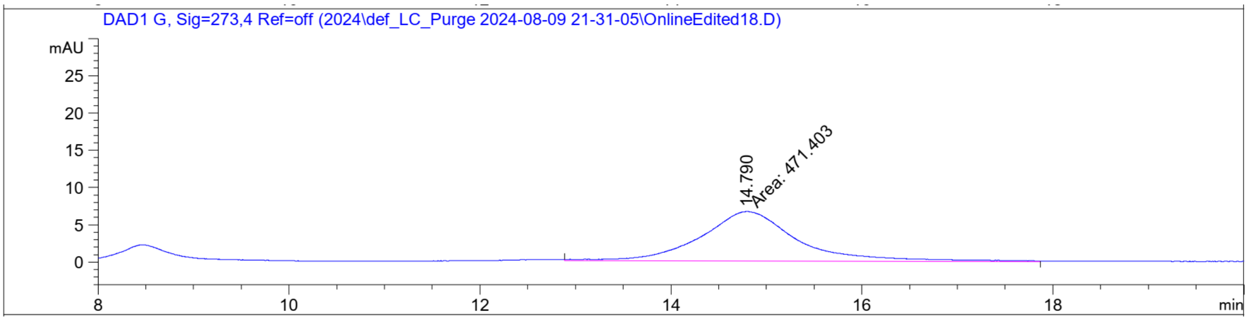

Peak #	RetTime [min]	Type	Width [min]	Area [mAU*s]	Height [mAU]	Area %
1	14.790	MM	1.1824	471.40259	6.64471	100.0000

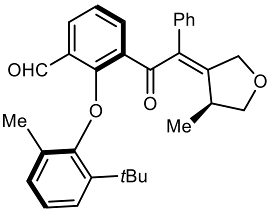

5d

(*S*,*E*)-2-(2-(tert-butyl)-6-methylphenoxy)-3-(2-(4-methyldihydrofuran-3(2H)-ylidene)-2-phenylacetyl)benzaldehyde (5d)

The general procedure was followed using bialdehyde **4b** (0.11 mmol, 32.6 mg) and 1,6-enyne **2f** (0.10 mmol, 17.2 mg) at room temperature for 72 h. Purification by column chromatography on silica gel (DCM) yielded **5d** (30.9 mg, 66%, 12:1 d.r., >99% e.e.) as a colorless oil.

^{1}H-NMR (300 MHz, CDCl$_3$) δ 9.19 (s, 1H), 7.70 (dd, J = 7.9, 1.8 Hz, 1H), 7.47 (dd, J = 7.5, 1.8 Hz, 1H), 7.29 – 7.18 (m, 6H), 7.08 – 7.03 (m, 1H), 7.01 – 6.95 (m, 2H), 4.50 (dd, J = 16.0, 1.8 Hz, 1H), 4.15 (d, J = 16.0 Hz, 1H), 3.95 (dd, J = 8.6, 6.0 Hz, 1H), 3.73 (dd, J = 8.5, 3.2 Hz, 1H), 3.64 – 3.51 (m, 1H), 1.84 (s, 3H), 1.43 (s, 9H), 1.31 (d, J = 7.0 Hz, 3H).

^{13}C-NMR (75 MHz, CDCl$_3$) δ 194.3 (C$_q$), 187.8 (CH), 159.0 (C$_q$), 156.9 (C$_q$), 156.6 (C$_q$), 140.1 (C$_q$), 137.4 (C$_q$), 134.9 (CH), 132.8 (C$_q$), 132.0 (C$_q$), 131.6 (CH), 131.6 (CH), 128.9 (CH), 128.8 (CH), 127.9 (CH), 127.7 (C$_q$), 126.9 (C$_q$), 126.0 (CH), 125.3 (CH), 121.9 (CH), 76.0 (CH$_2$), 71.3 (CH$_2$), 37.9 (CH), 35.4 (C$_q$), 30.3 (CH$_3$), 18.8 (CH$_3$), 17.6 (CH$_3$).

HR-MS (ESI): m/z calcd. for $[C_{31}H_{32}O_4 + Na]^+$ 491.2193, found 491.2183.

IR (ATR): 1694, 1652, 1466, 1389, 1295, 1158, 1093, 754 cm^{-1}.

[α$^{20}_{D}$] = +136.5 (c = 1.0, DCM).

HPLC separation (Chiralpak® ID-3, n-hexane/i-PrOH 98:2, 1.0 mL/min, detection at 250 nm): t_r (minor) = 15.4 min, >99% e.e.

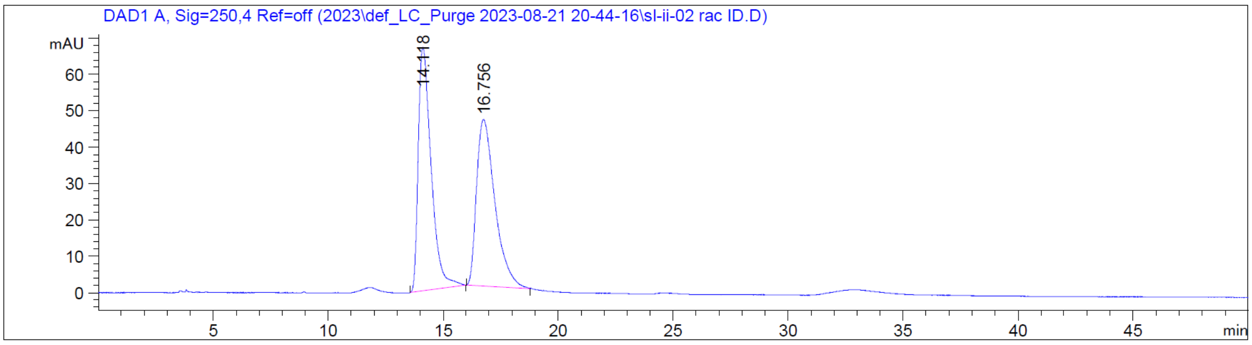

Peak #	RetTime [min]	Type	Width [min]	Area [mAU*s]	Height [mAU]	Area %
1	14.118	BB	0.5174	2610.41626	67.02258	50.6631
2	16.756	BB	0.6531	2542.07910	45.83506	49.3369

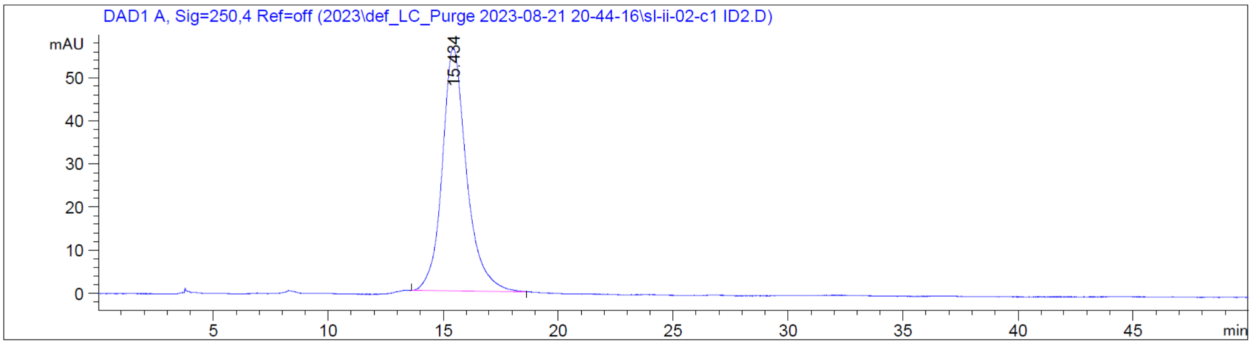


```
Peak RetTime Type  Width     Area      Height     Area
  #   [min]        [min]   [mAU*s]     [mAU]        %
----|-------|----|-------|----------|----------|--------|
   1  15.434 MM R  1.2292 4162.69238   56.44265 100.0000
```

The construction of 1,5-central/[2.2]paracyclophane planar chirality.

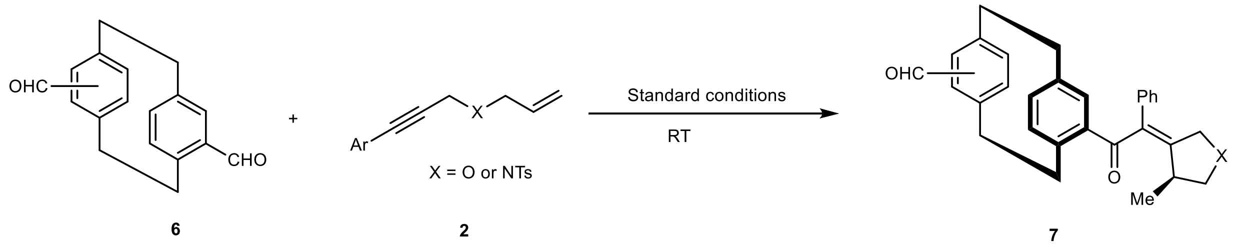

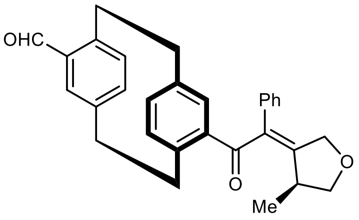

7a

(*S*,*E*)-4^3-(2-(4-methyldihydrofuran-3(2H)-ylidene)-2-phenylacetyl)-1,4(1,4)-dibenzenacyclohexaphane-12-carbaldehyde (7a)

The general procedure was followed using bialdehyde **6a** (0.11 mmol, 29.0 mg) and 1,6-enyne **2f** (0.10 mmol, 17.2 mg) at room temperature for 72 h. Purification by column chromatography on silica gel (DCM) yielded **7a** (31.9 mg, 73%, 7:1 d.r., >99% e.e.) as a colorless oil.

^{1}H-NMR (400 MHz, $CDCl_3$) δ 9.90 (s, 1H), 7.37 – 7.34 (m, 2H), 7.30 – 7.27 (m, 3H), 7.00 (d, *J* = 2.0 Hz, 1H), 6.92 (d, *J* = 1.9 Hz, 1H), 6.56 (dd, *J* = 7.8, 2.0 Hz, 1H), 6.44 (dd, *J* = 7.8, 1.9 Hz, 1H), 6.35 (d, *J* = 7.8 Hz, 1H), 5.88 (d, *J* = 7.7 Hz, 1H), 4.59 (dd, *J* = 14.8, 1.6 Hz, 1H), 4.16 (d, *J*

= 14.9 Hz, 1H), 4.04 – 3.97 (m, 1H), 3.92 – 3.85 (m, 2H), 3.61 (dd, J = 8.4, 2.9 Hz, 1H), 3.29 – 3.22 (m, 1H), 3.18 – 3.00 (m, 5H), 2.81 – 2.73 (m, 1H), 0.88 (d, J = 6.9 Hz, 3H).

^{13}C-NMR (101 MHz, CDCl$_3$) δ 197.4 (C$_q$), 192.2 (CH), 150.8 (C$_q$), 142.8 (C$_q$), 141.5 (C$_q$), 140.7 (C$_q$), 139.4 (C$_q$), 138.6 (C$_q$), 137.3 (C$_q$), 136.7 (CH), 136.5 (CH), 136.4 (C$_q$), 135.8 (CH), 135.4 (CH), 135.1 (CH), 134.8 (CH), 134.1 (C$_q$), 128.7 (CH), 128.3 (CH), 127.9 (CH), 75.6 (CH$_2$), 70.2 (CH$_2$), 37.7 (CH), 34.7 (CH$_2$), 34.7 (CH$_2$), 34.5 (CH$_2$), 32.9 (CH$_2$), 18.5 (CH$_3$).

IR (ATR): 1683, 1654, 1451, 1389, 1231, 1158, 1093, 702 cm^{-1}.

HRMS (ESI): *m/z* [M+H]$^+$ calculated for $C_{30}H_{29}O_3^+$: 437.2111, found 437.2110.

$[\alpha^{20}_{D}]$ = +39.4 (c = 0.5, DCM).

HPLC separation (IA column, *n*-hexane/i-PrOH 98/2, 1.0 mL/min, 250.0 nm): t_r(major) = 21.4 min, t_r(minor) = 33.6 min, >99% e.e.

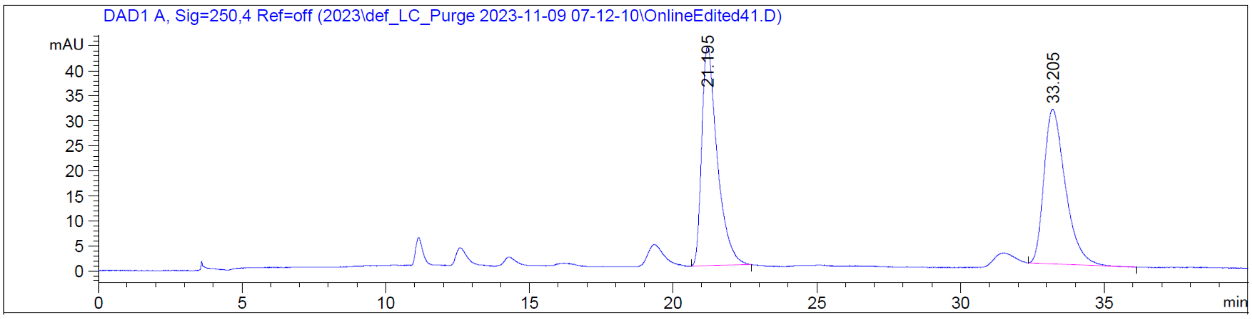

Peak #	RetTime [min]	Type	Width [min]	Area [mAU*s]	Height [mAU]	Area %
1	21.195	BB	0.5154	1641.02991	43.87353	50.4099
2	33.205	MM R	0.8679	1614.34497	31.00229	49.5901

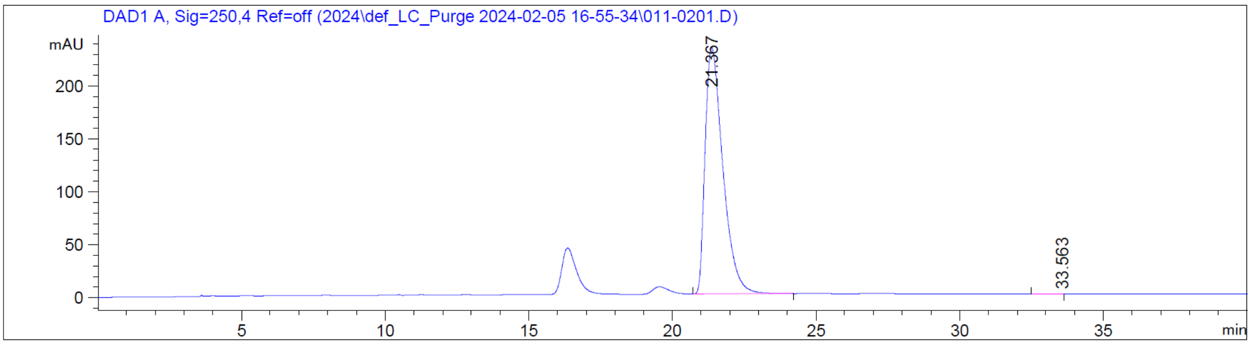

Peak #	RetTime [min]	Type	Width [min]	Area [mAU*s]	Height [mAU]	Area %
1	21.367	BB	0.6590	1.00345e4	232.73192	99.8115
2	33.563	MM R	0.9064	18.95542	3.48552e-1	0.1885

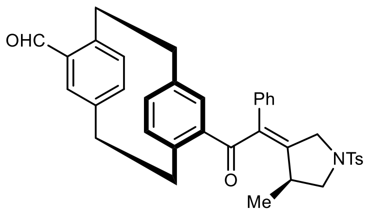

7b

(*S,E*)-4^3-(2-(4-methyl-1-tosylpyrrolidin-3-ylidene)-2-phenylacetyl)-1,4(1,4)-dibenzenacyclohexaphane-12-carbaldehyde (7b)

The general procedure was followed using bialdehyde **6a** (0.11 mmol, 29.0 mg) and 1,6-enyne **2a** (0.10 mmol, 32.5 mg) at room temperature for 72 h. Purification by column chromatography on silica gel (DCM) yielded **7b** (32.4 mg, 55%, 4:1 d.r., >99% e.e.) as a colorless oil.

^{1}H-NMR (400 MHz, $CDCl_3$) δ 9.87 (s, 1H), 7.62 – 7.60 (m, 2H), 7.44 – 7.39 (m, 2H), 7.32 – 7.27 (m, 5H), 6.97 (d, *J* = 2.0 Hz, 1H), 6.86 (d, *J* = 1.9 Hz, 1H), 6.45 (dd, *J* = 7.8, 2.0 Hz, 1H), 6.40 (dd, *J* = 7.9, 1.9 Hz, 1H), 6.37 (d, *J* = 7.8 Hz, 1H), 5.63 (d, *J* = 7.8 Hz, 1H), 4.22 (dd, *J* = 15.3, 1.4 Hz, 1H), 4.01 – 3.88 (m, 2H), 3.57 (d, *J* = 15.3 Hz, 1H), 3.28 – 3.20 (m, 1H), 3.14 (d, *J* = 4.0 Hz, 2H), 3.13 – 2.93 (m, 5H), 2.73 – 2.66 (m, 1H), 2.43 (s, 3H), 0.71 (d, *J* = 6.9 Hz, 3H).

^{13}C-NMR (101 MHz, $CDCl_3$) δ 197.1 (C_q), 192.2 (CH), 144.3 (C_q), 143.9 (C_q), 142.7 (C_q), 142.2 (C_q), 140.7 (C_q), 139.5 (C_q), 137.8 (C_q), 136.6 (CH), 136.5 (C_q), 136.5 (C_q), 136.4 (CH), 136.4 (C_q), 136.2 (CH), 135.4 (CH), 135.4 (CH), 135.3 (CH), 133.0 (C_q), 129.9 (CH), 129.0 (CH), 128.4 (CH), 128.2 (CH), 127.8 (CH), 54.8 (CH_2), 50.1 (CH_2), 36.8 (CH), 34.6 (CH_2), 34.5 (CH_2), 34.5 (CH_2), 32.9 (CH_2), 21.7 (CH_3), 19.1 (CH_3).

HRMS (ESI): *m/z* $[M+Na]^+$ calculated for $C_{37}H_{35}NNaO_4S^+$: 612.2179, found 612.2188.

IR (ATR): 1682, 1560, 1453, 1229, 1161, 923, 735 cm^{-1}.

$[\alpha_D^{20}]$ = +58.6 (c = 1.0, DCM).

HPLC separation (IA-3 column, *n*-hexane/*i*-PrOH 70/30, 1.0 mL/min, 250.0 nm): t_r(major) = 11.8 min, t_r(minor) = 18.2 min, >99% e.e.

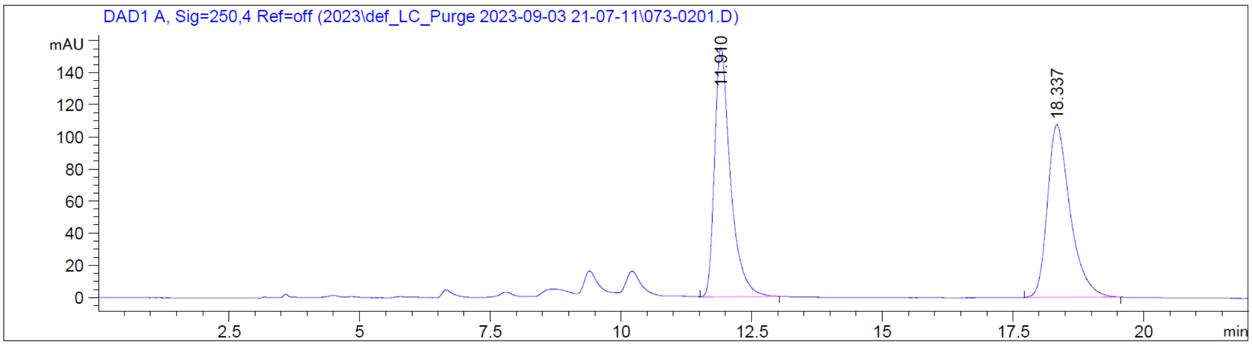

Peak #	RetTime [min]	Type	Width [min]	Area [mAU*s]	Height [mAU]	Area %
1	11.910	BB	0.3133	3269.65991	154.90086	49.9999
2	18.337	BB	0.4466	3269.66772	107.56158	50.0001

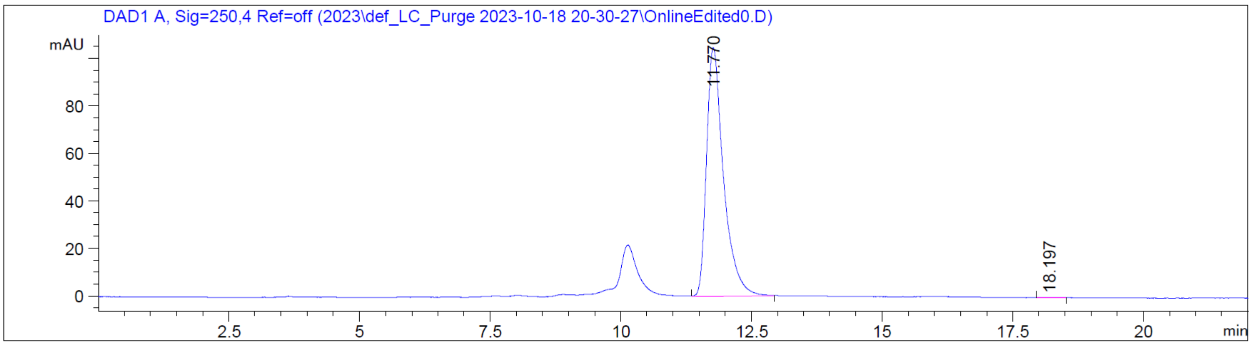

Peak #	RetTime [min]	Type	Width [min]	Area [mAU*s]	Height [mAU]	Area %
1	11.770	MM R	0.3753	2348.07861	104.27399	99.7753
2	18.197	MM R	0.3893	5.28804	2.26365e-1	0.2247

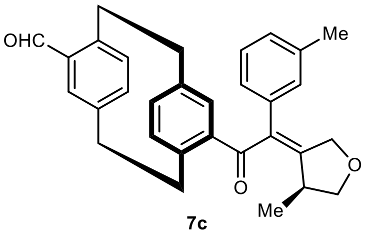

7c

(*S,E*)-4^3-(2-(4-methyldihydrofuran-3(2H)-ylidene)-2-(m-tolyl)acetyl)-1,4(1,4)-dibenzenacyclohexaphane-12-carbaldehyde (7c)

The general procedure was followed using bialdehyde **6a** (0.11 mmol, 29.0 mg) and 1,6-enyne **2e** (0.10 mmol, 19.0 mg) at room temperature for 72 h. Purification by column chromatography on silica gel (DCM) yielded **7c** (30.2 mg, 67%, 7:1 d.r., >99% e.e.) as a colorless oil. The diastereomeric products can be separated by silica gel chromatography.

The major diastereomer:

^{1}H-NMR (300 MHz, $CDCl_3$) δ 9.91 (s, 1H), 7.29 – 7.24 (m, 1H), 7.14 – 7.07 (m, 3H), 7.01 (d, *J* = 2.0 Hz, 1H), 6.94 (d, *J* = 1.9 Hz, 1H), 6.53 (dd, *J* = 7.8, 2.0 Hz, 1H), 6.45 (dd, *J* = 7.8, 1.9 Hz, 1H), 6.37 (d, *J* = 7.8 Hz, 1H), 5.81 (d, *J* = 7.8 Hz, 1H), 4.60 (dd, *J* = 14.8, 1.6 Hz, 1H), 4.17 (d, *J* = 14.8 Hz, 1H), 4.05 – 3.96 (m, 1H), 3.96 – 3.80 (m, 2H), 3.59 (dd, *J* = 8.4, 2.9 Hz, 1H), 3.32 – 3.22 (m, 1H), 3.17 – 3.04 (m, 3H), 3.03 – 2.93 (m, 2H), 2.81 – 2.70 (m, 1H), 2.36 (s, 3H), 0.84 (d, *J* = 7.0 Hz, 3H).

^{13}C-NMR (75 MHz, $CDCl_3$) δ 197.5 (C_q), 192.2 (CH), 150.0 (C_q), 142.8 (C_q), 141.7 (C_q), 140.8 (C_q), 139.4 (C_q), 138.6 (C_q), 138.4 (C_q), 137.2 (C_q), 136.7 (CH), 136.5 (CH), 136.5 (C_q), 135.8 (CH), 135.4 (CH), 135.2 (CH), 135.0 (CH), 134.3 (C_q), 128.9 (CH), 128.7 (CH), 128.7 (CH), 125.4 (CH), 75.6 (CH_2), 70.2 (CH_2), 37.6 (CH), 34.7 (CH_2), 34.7 (CH_2), 34.5 (CH_2), 33.0 (CH_2), 21.6 (CH_3), 18.5 (CH_3).

IR (ATR): 1683, 1655, 1551, 1452, 1238, 1162, 921, 736 cm^{-1}.

HRMS (ESI): *m/z* $[M+H]^+$ calculated for $C_{31}H_{31}O_3^+$: 451.2268, found 451.2262.

$[\alpha_D^{20}]$ = +34.8 (c = 0.5, DCM).

HPLC separation (Chiralpak® IA-3 column, *n*-hexane/*i*-PrOH 98/2, 1.0 mL/min, 250.0 nm): t_r(major) = 16.8 min, t_r(minor) = 20.5 min, >99% e.e.

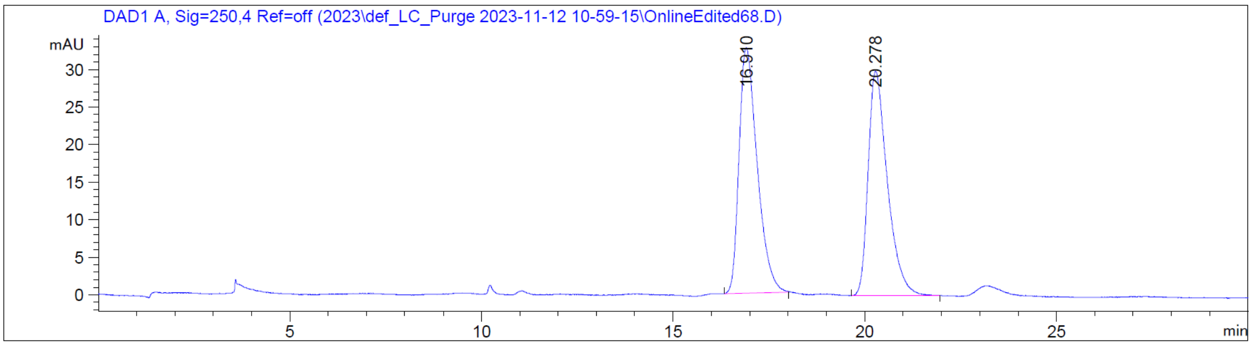

Peak #	RetTime [min]	Type	Width [min]	Area [mAU*s]	Height [mAU]	Area %
1	16.910	BB	0.4571	1075.09741	32.65594	50.6167
2	20.278	MM R	0.5852	1048.89905	29.87377	49.3833

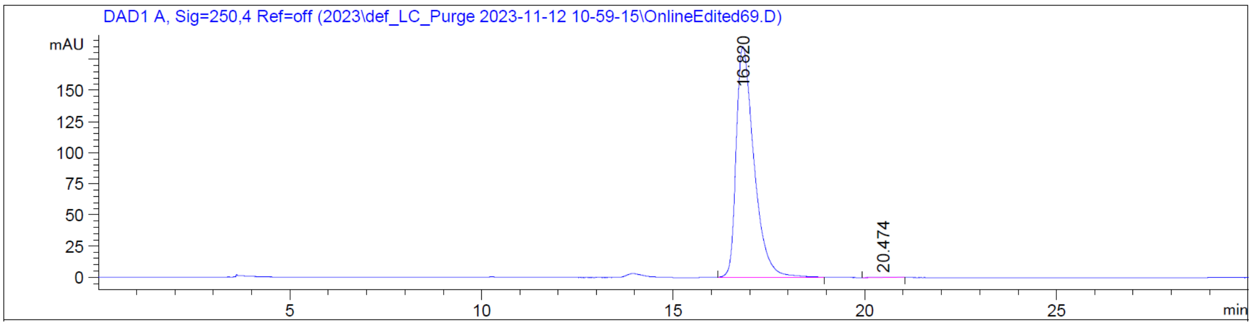

Peak #	RetTime [min]	Type	Width [min]	Area [mAU*s]	Height [mAU]	Area %
1	16.820	BB	0.4868	5977.92285	185.07388	99.7758
2	20.474	MM R	0.5569	13.43370	4.02053e-1	0.2242

The minor diastereomer:

^{1}H-NMR (400 MHz, CDCl$_3$) δ 9.94 (s, 1H), 7.26 – 7.23 (m, 1H), 7.11 – 7.01 (m, 4H), 6.93 (d, *J* = 2.0 Hz, 1H), 6.74 (dd, *J* = 7.8, 2.0 Hz, 1H), 6.54 (dd, *J* = 7.8, 1.9 Hz, 1H), 6.43 (d, *J* = 7.8 Hz, 1H), 6.20 (d, *J* = 7.8 Hz, 1H), 4.44 (dd, *J* = 15.0, 1.8 Hz, 1H), 4.25 (d, *J* = 15.0 Hz, 1H), 4.12 – 4.06 (m, 1H), 3.88 – 3.78 (m, 2H), 3.46 (dd, *J* = 8.7, 4.3 Hz, 1H), 3.44 – 3.36 (m, 1H), 3.21 – 3.13 (m, 3H), 2.96 – 2.87 (m, 2H), 2.58 – 2.50 (m, 1H), 2.36 (s, 3H), 1.00 (d, *J* = 7.0 Hz, 3H).

^{13}C-NMR (101 MHz, CDCl$_3$) δ 198.5 (C$_q$), 192.4 (CH), 152.2 (C$_q$), 142.8 (C$_q$), 142.2 (C$_q$), 141.3 (C$_q$), 139.7 (C$_q$), 138.4 (C$_q$), 138.3 (C$_q$), 137.3 (C$_q$), 136.8 (CH), 136.6 (C$_q$), 136.0 (CH), 135.7 (CH), 135.3 (CH), 133.8 (CH), 133.7 (C$_q$), 129.1 (CH), 128.7 (CH), 128.6 (CH), 125.4 (CH), 75.7 (CH$_2$), 71.0 (CH$_2$), 37.0 (CH), 34.7 (CH$_2$), 34.6 (CH$_2$), 34.6 (CH$_2$), 32.7 (CH$_2$), 21.7 (CH$_3$), 19.1 (CH$_3$).

HRMS (ESI): *m/z* [M+H]$^+$ calculated for $C_{31}H_{31}O_3^+$: 451.2268, found 451.2270.

HPLC separation (Chiralpak® ID-3 column, *n*-hexane/*i*-PrOH 95/5, 1.0 mL/min, 250.0 nm): t_r(minor) = 16.7 min, t_r(major) = 20.8 min, 90% e.e.

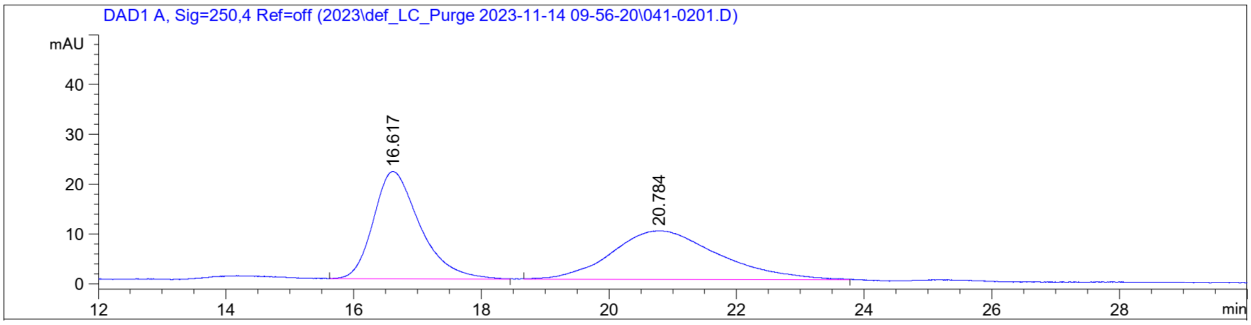

Peak #	RetTime [min]	Type	Width [min]	Area [mAU*s]	Height [mAU]	Area %
1	16.617	MM R	0.8450	1091.03699	21.51906	49.2626
2	20.784	MM R	1.9262	1123.69812	9.72315	50.7374

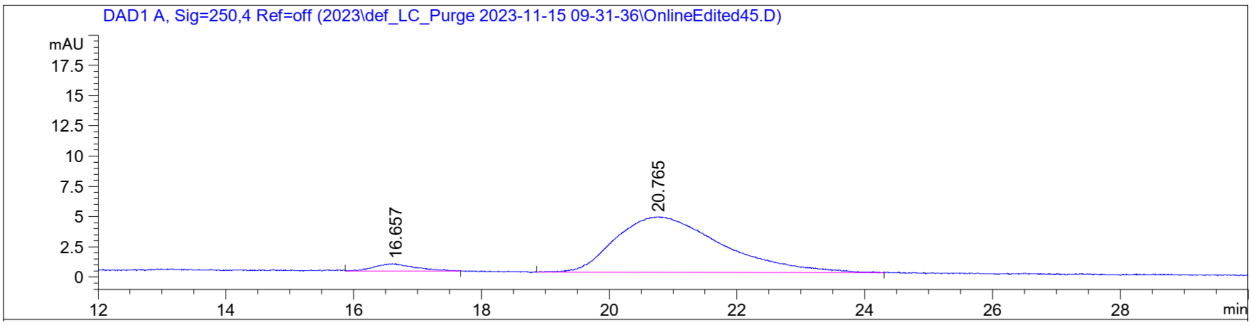

Peak #	RetTime [min]	Type	Width [min]	Area [mAU*s]	Height [mAU]	Area %
1	16.657	MM R	0.7516	27.01454	5.99023e-1	4.8179
2	20.765	MM R	1.9392	533.69714	4.58680	95.1821

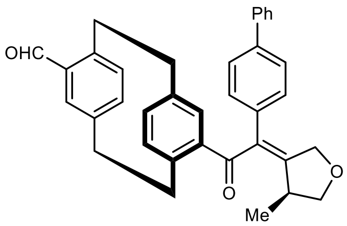

7d

(*S,E*)-4[3]-(2-([1,1'-biphenyl]-4-yl)-2-(4-methyldihydrofuran-3(2H)-ylidene)acetyl)-1,4(1,4)-dibenzenacyclohexaphane-12-carbaldehyde (7d)

The general procedure was followed using bialdehyde **6a** (0.11 mmol, 29.0 mg) and 1,6-enyne **2g** (0.10 mmol, 24.8 mg) at room temperature for 72 h. Purification by column chromatography on silica gel (DCM) yielded **7d** (30.8 mg, 60%, 6:1 d.r., >99% e.e.) as a colorless oil.

^{1}H-NMR (400 MHz, CDCl$_3$) δ 9.91 (s, 1H), 7.63 – 7.57 (m, 4H), 7.45 – 7.41 (m, 2H), 7.38 – 7.31 (m, 3H), 7.02 (d, *J* = 2.0 Hz, 1H), 6.95 (d, *J* = 2.0 Hz, 1H), 6.62 (dd, *J* = 7.8, 2.0 Hz, 1H), 6.46 (dd, *J* = 7.8, 2.0 Hz, 1H), 6.37 (d, *J* = 7.8 Hz, 1H), 5.95 (d, *J* = 7.8 Hz, 1H), 4.65 (dd, *J* = 14.8, 1.6 Hz, 1H), 4.24 (d, *J* = 14.9 Hz, 1H), 4.05 – 3.98 (m, 1H), 3.95 – 3.86 (m, 2H), 3.63 (dd, *J* = 8.4, 2.8 Hz, 1H), 3.33 – 3.25 (m, 1H), 3.17 – 2.95 (m, 5H), 2.84 – 2.75 (m, 1H), 0.89 (d, *J* = 7.0 Hz, 3H).

^{13}C-NMR (101 MHz, CDCl$_3$) δ 197.5 (C$_q$), 192.2 (CH), 150.8 (C$_q$), 142.9 (C$_q$), 141.5 (C$_q$), 140.7 (C$_q$), 140.6 (C$_q$), 140.4 (C$_q$), 139.5 (C$_q$), 138.7 (C$_q$), 136.6 (CH), 136.5 (CH), 136.5 (C$_q$), 136.2 (C$_q$), 135.9 (CH), 135.4 (CH), 135.2 (CH), 134.8 (CH), 133.8 (C$_q$), 129.0 (CH), 128.7 (CH), 127.7 (CH), 127.3 (CH), 127.1 (CH), 75.6 (CH$_2$), 70.2 (CH$_2$), 37.8 (CH), 34.7 (CH$_2$), 34.7 (CH$_2$), 34.5 (CH$_2$), 32.9 (CH$_2$), 18.6 (CH$_3$).

IR (ATR): 1684, 1656, 1590, 1551, 1231, 1085, 922, 702 cm^{-1}.

HRMS (ESI): *m/z* [M+Na]$^+$ calculated for $C_{36}H_{32}NaO_3^+$: 535.2244, found 535.2236.

[α^{20}_{D}] = +4.8 (c = 1.0, DCM).

HPLC Separation (IA-3 column, *n*-hexane/*i*-PrOH 90/10, 1.0 mL/min, 250.0 nm): t_r(major) = 15.4 min, >99% e.e.

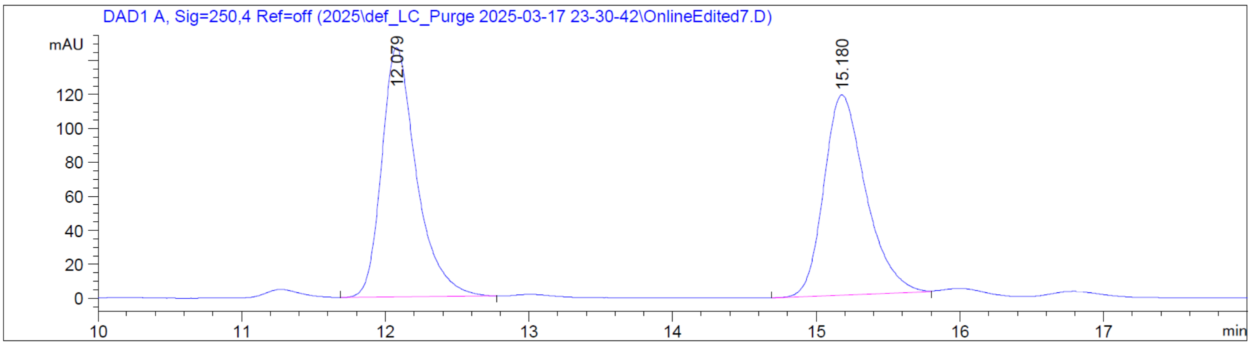

Peak #	RetTime [min]	Type	Width [min]	Area [mAU*s]	Height [mAU]	Area %
1	12.079	MM R	0.2807	2472.98584	146.83577	50.8435
2	15.180	MM R	0.3376	2390.93018	118.03157	49.1565

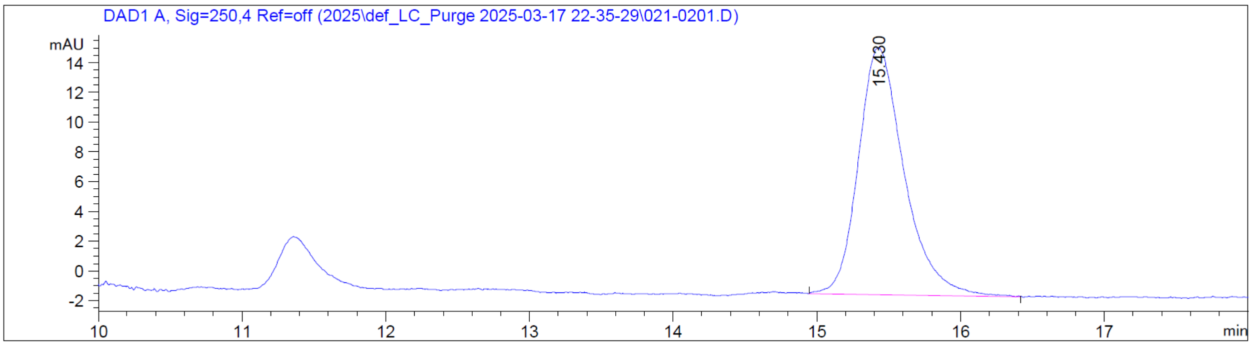

Peak #	RetTime [min]	Type	Width [min]	Area [mAU*s]	Height [mAU]	Area %
1	15.430	MM R	0.3708	369.75067	16.62037	100.0000

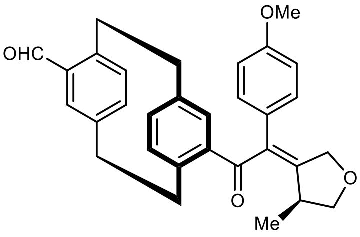

7e

(*S*,*E*)-4³-(2-(4-methoxyphenyl)-2-(4-methyldihydrofuran-3(2H)-ylidene)acetyl)-1,4(1,4)-dibenzenacyclohexaphane-12-carbaldehyde (7e)

The general procedure was followed using bialdehyde **6a** (0.11 mmol, 29.0 mg) and 1,6-enyne **2h** (0.10 mmol, 20.2 mg) at room temperature for 72 h. Purification by column chromatography on silica gel (DCM) yielded **7e** (24.3 mg, 52%, 7:1 d.r., >99% e.e.) as a colorless oil. The diastereomeric products can be separated by silica gel chromatography.

The major diastereomer:

^{1}H-NMR (300 MHz, $CDCl_3$) δ 9.91 (s, 1H), 7.24 – 7.15 (m, 2H), 7.01 (d, *J* = 2.0 Hz, 1H), 6.95 – 6.83 (m, 3H), 6.59 (dd, *J* = 7.8, 2.0 Hz, 1H), 6.45 (dd, *J* = 7.8, 2.0 Hz, 1H), 6.36 (d, *J* = 7.8 Hz, 1H), 5.95 (d, *J* = 7.8 Hz, 1H), 4.58 (dd, *J* = 14.7, 1.6 Hz, 1H), 4.17 (d, *J* = 14.7 Hz, 1H), 4.06 – 3.97 (m, 1H), 3.92 – 3.83 (m, 2H), 3.79 (s, 3H), 3.60 (dd, *J* = 8.4, 2.8 Hz, 1H), 3.31 – 3.21 (m, 1H), 3.17 – 2.90 (m, 5H), 2.85 – 2.74 (m, 1H), 0.87 (d, *J* = 7.0 Hz, 3H).

^{13}C-NMR (101 MHz, $CDCl_3$) δ 197.8 (C_q), 192.2 (CH), 159.2 (C_q), 149.8 (C_q), 142.9 (C_q), 141.5

(C_q), 140.8 (C_q), 139.4 (C_q), 138.7 (C_q), 136.7 (CH), 136.5 (CH), 136.5 (C_q), 135.7 (CH), 135.5 (CH), 135.1 (CH), 134.8 (CH), 133.7 (C_q), 129.6 (C_q), 129.5 (CH), 114.1 (CH), 75.6 (CH_2), 70.2 (CH_2), 55.4 (CH_3), 37.7 (CH), 34.7 (CH_2), 34.7 (CH_2), 34.5 (CH_2), 32.9 (CH_2), 18.6 (CH_3).

IR (ATR): 1683, 1655, 1551, 1248, 1161, 1033, 735 cm^{-1}.

HR-MS (ESI): *m*/*z* calcd. for $[C_{31}H_{30}O_4 + Na]^+$ 489.2036, found 489.2029.

$[\alpha_D^{20}]$ = +118.1 (c = 1.0, DCM).

HPLC separation (Chiralpak® IF-3, *n*-hexane/*i*-PrOH 95:5, 1.0 mL/min, detection at 250 nm): t_r (major) = 32.7 min, >99% e.e.

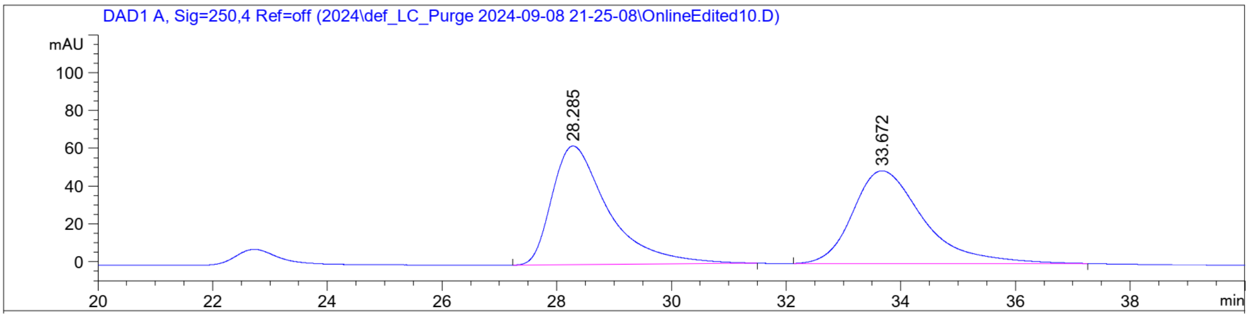

Peak #	RetTime [min]	Type	Width [min]	Area [mAU*s]	Height [mAU]	Area %
1	28.285	BB	0.8586	4284.92969	62.75389	50.5176
2	33.672	BB	1.0023	4197.13184	49.00156	49.4824

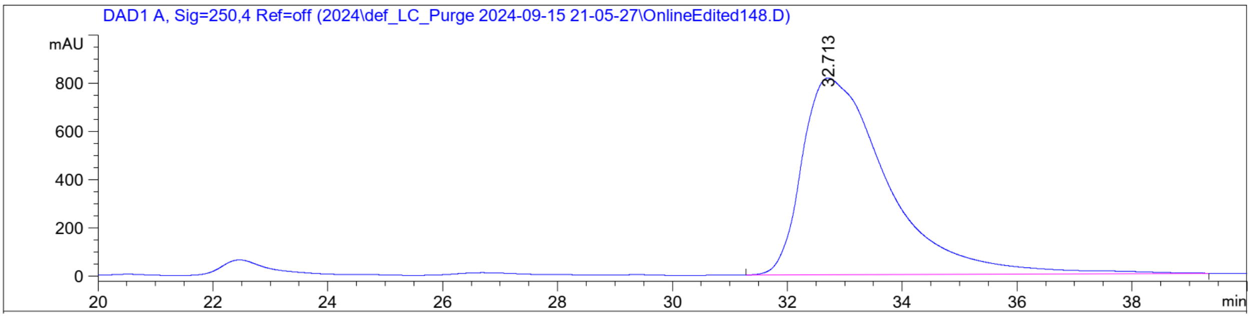

Peak #	RetTime [min]	Type	Width [min]	Area [mAU*s]	Height [mAU]	Area %
1	32.713	BB	1.3822	8.43799e4	814.80048	100.0000

The minor diastereomer:

^{1}H-NMR (300 MHz, $CDCl_3$) δ 9.91 (s, 1H), 7.22 – 7.17 (m, 2H), 7.00 (d, *J* = 2.0 Hz, 1H), 6.91 – 6.85 (m, 3H), 6.58 (dd, *J* = 7.8, 2.0 Hz, 1H), 6.45 (dd, *J* = 7.8, 2.0 Hz, 1H), 6.35 (d, *J* = 7.8 Hz, 1H), 5.95 (d, *J* = 7.8 Hz, 1H), 4.58 (dd, *J* = 14.7, 1.6 Hz, 1H), 4.17 (d, *J* = 14.7 Hz, 1H), 4.06 –

3.96 (m, 1H), 3.91 – 3.83 (m, 2H), 3.79 (s, 3H), 3.60 (dd, J = 8.4, 2.7 Hz, 1H), 3.31 – 3.21 (m, 1H), 3.13 – 2.93 (m, 5H), 2.84 – 2.74 (m, 1H), 0.86 (d, J = 7.0 Hz, 3H).

^{13}C-NMR (101 MHz, CDCl$_3$) δ 197.8 (C$_q$), 192.2 (CH), 159.2 (C$_q$), 149.7 (C$_q$), 142.9 (C$_q$), 141.5 (C$_q$), 140.7 (C$_q$), 139.4 (C$_q$), 138.7 (C$_q$), 136.6 (CH), 136.5 (CH), 136.4 (C$_q$), 135.7 (CH), 135.5 (CH), 135.1 (CH), 134.8 (CH), 133.7 (C$_q$), 129.6 (C$_q$), 129.5 (CH), 114.1 (CH), 75.6 (CH$_2$), 70.2 (CH$_2$), 55.4 (CH$_3$), 37.6 (CH), 34.7 (CH$_2$), 34.7 (CH$_2$), 34.5 (CH$_2$), 32.9 (CH$_2$), 18.5 (CH$_3$).

HR-MS (ESI): m/z calcd. for $[C_{31}H_{30}O_4 + Na]^+$ 489.2036, found 489.2040.

HPLC separation (Chiralpak® IF-3, n-hexane/i-PrOH 95:5, 1.0 mL/min, detection at 250 nm): t_r (minor) = 26.6 min, t_r (major) = 33.2 min, 95% e.e.

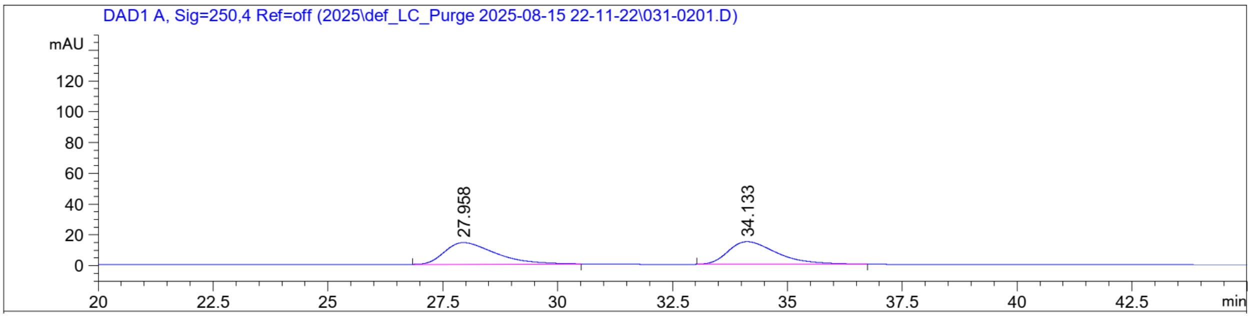

Peak #	RetTime [min]	Type	Width [min]	Area [mAU*s]	Height [mAU]	Area %
1	27.958	BB	0.8961	1069.01050	14.02745	49.8508
2	34.133	BB	0.8616	1075.40979	14.66309	50.1492

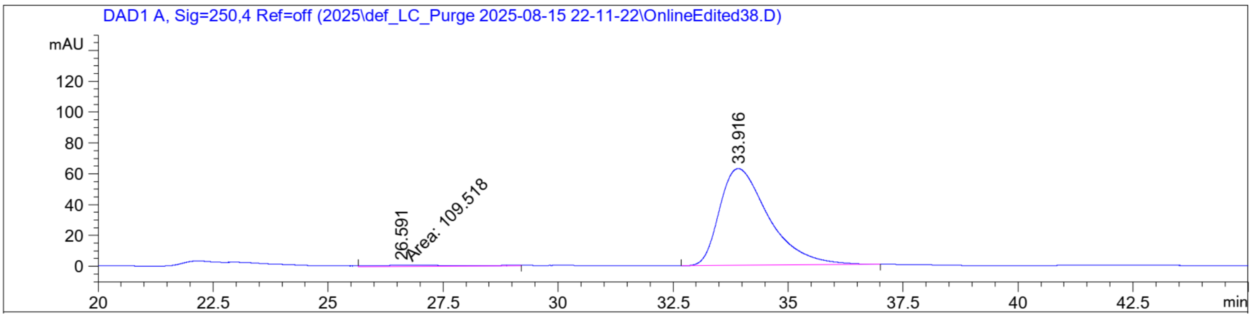

Peak #	RetTime [min]	Type	Width [min]	Area [mAU*s]	Height [mAU]	Area %
1	26.591	MM	2.3750	109.51785	7.68554e-1	2.2898
2	33.916	BB	0.9943	4673.41992	62.79661	97.7102

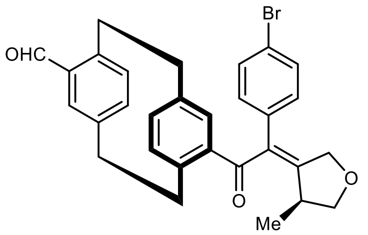

7f

(*S,E*)-4^3-(2-(4-bromophenyl)-2-(4-methyldihydrofuran-3(2H)-ylidene)acetyl)-1,4(1,4)-dibenzenacyclohexaphane-12-carbaldehyde (7f)

The general procedure was followed using bialdehyde **6a** (0.11 mmol, 29.0 mg) and 1,6-enyne **2k** (0.10 mmol, 25.1 mg) at room temperature for 72 h. Purification by column chromatography on silica gel (DCM) yielded **7f** (33.5 mg, 65%, 8:1 d.r., 99% e.e.) as a colorless oil. The diastereomeric products can be separated by silica gel chromatography.

The major diastereomer:

^{1}H-NMR (300 MHz, CDCl$_3$) δ 9.91 (s, 1H), 7.55 – 7.40 (m, 2H), 7.15 – 7.03 (m, 2H), 7.00 (d, *J* = 2.0 Hz, 1H), 6.82 (d, *J* = 1.9 Hz, 1H), 6.62 (dd, *J* = 7.8, 2.0 Hz, 1H), 6.46 (dd, *J* = 7.8, 1.9 Hz, 1H), 6.34 (d, *J* = 7.8 Hz, 1H), 6.08 (d, *J* = 7.8 Hz, 1H), 4.51 (dd, *J* = 15.1, 1.6 Hz, 1H), 4.12 (d, *J* = 15.1 Hz, 1H), 4.03 (ddd, *J* = 12.8, 9.7, 2.9 Hz, 1H), 3.88 (dd, *J* = 8.5, 5.7 Hz, 1H), 3.74 (ddd, *J* = 13.1, 10.2, 3.2 Hz, 1H), 3.62 (dd, *J* = 8.5, 2.7 Hz, 1H), 3.24 (ddd, *J* = 12.7, 10.2, 4.7 Hz, 1H), 3.17 – 2.99 (m, 4H), 2.95 – 2.87 (m, 1H), 2.86 – 2.76 (m, 1H), 0.90 (d, *J* = 7.0 Hz, 3H).

^{13}C-NMR (101 MHz, CDCl$_3$) δ 197.0 (C_q), 192.1 (CH), 152.6 (C_q), 142.9 (C_q), 141.0 (C_q), 140.6 (C_q), 139.6 (C_q), 138.6 (C_q), 136.5 (CH), 136.5 (C_q), 136.4 (CH), 136.3 (C_q), 135.8 (CH), 135.4 (CH), 135.0 (CH), 134.2 (CH), 133.0 (C_q), 131.8 (CH), 129.9 (CH), 122.0 (C_q), 75.6 (CH_2), 70.2 (CH_2), 37.9 (CH), 34.7 (CH_2), 34.6 (CH_2), 34.4 (CH_2), 32.8 (CH_2), 18.6 (CH_3).

IR (ATR): 1683, 1354, 1277, 1160, 1124, 1011, 739, 712 cm^{-1}.

HR-MS (ESI): *m*/*z* calcd. for $[C_{30}H_{27}BrO_3 + Na]^+$ 537.1036, found 537.1020.

$[\alpha^{20}_{D}]$ = +56.4 (c = 1.0, DCM).

HPLC separation (Chiralpak® IF-3, *n*-hexane/*i*-PrOH 95:5, 1.0 mL/min, detection at 250 nm): t_r (major) = 20.9 min, t_r (minor) = 24.7 min, 99% e.e.

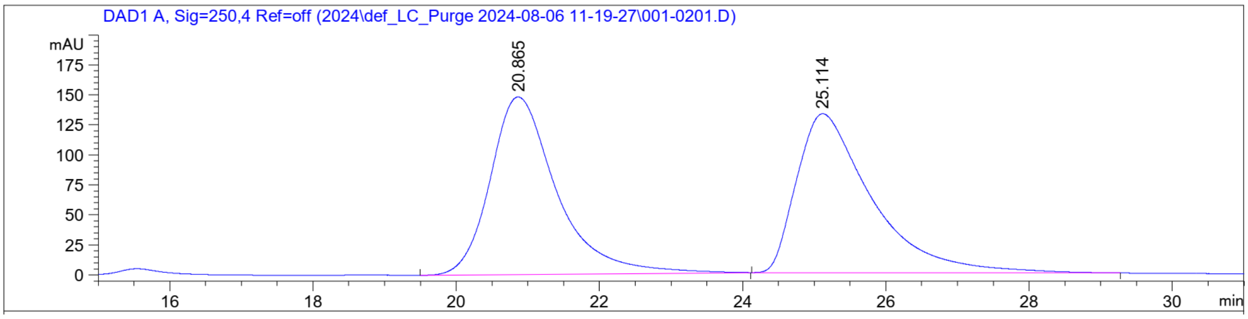

Peak #	RetTime [min]	Type	Width [min]	Area [mAU*s]	Height [mAU]	Area %
1	20.865	BB	0.9037	9541.93652	147.90125	49.7304
2	25.114	BB	0.9980	9645.40234	132.38477	50.2696

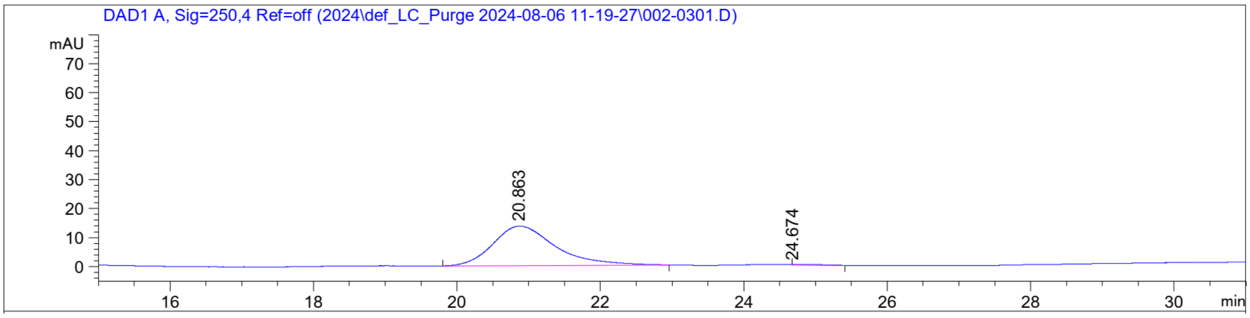

Peak #	RetTime [min]	Type	Width [min]	Area [mAU*s]	Height [mAU]	Area %
1	20.863	BB	0.7127	826.87598	13.65025	99.3680
2	24.674	MM R	0.4882	5.25913	6.29081e-2	0.6320

The minor diastereomer:

^{1}H-NMR (400 MHz, CDCl$_3$) δ 9.94 (s, 1H), 7.48 – 7.45 (m, 2H), 7.10 – 7.06 (m, 2H), 7.04 (d, *J* = 2.0 Hz, 1H), 6.81 (d, *J* = 2.0 Hz, 1H), 6.76 (dd, *J* = 7.8, 2.0 Hz, 1H), 6.54 (dd, *J* = 7.8, 1.9 Hz, 1H), 6.42 (d, *J* = 7.8 Hz, 1H), 6.21 (d, *J* = 7.8 Hz, 1H), 4.40 (dd, *J* = 15.1, 1.9 Hz, 1H), 4.20 (d, *J* = 15.1 Hz, 1H), 4.12 – 4.05 (m, 1H), 3.84 (dd, *J* = 8.7, 6.4 Hz, 1H), 3.80 – 3.73 (m, 1H), 3.50 (dd, *J* = 8.7, 4.0 Hz, 1H), 3.47 – 3.38 (m, 1H), 3.21 – 3.12 (m, 3H), 2.95 – 2.87 (m, 2H), 2.73 – 2.64 (m, 1H), 1.03 (d, *J* = 6.9 Hz, 3H).

^{13}C-NMR (101 MHz, CDCl$_3$) δ 197.9 (C$_q$), 192.3 (CH), 154.2 (C$_q$), 142.8 (C$_q$), 142.1 (C$_q$), 141.3 (C$_q$), 139.7 (C$_q$), 137.2 (C$_q$), 137.0 (C$_q$), 136.8 (CH), 136.6 (C$_q$), 135.8 (CH), 135.8 (CH), 135.3 (CH), 135.3 (CH), 133.3 (CH), 132.5 (C$_q$), 132.0 (CH), 130.1 (CH), 121.9 (C$_q$), 75.7 (CH$_2$), 71.0 (CH$_2$), 37.1 (CH), 34.7 (CH$_2$), 34.6 (CH$_2$), 34.5 (CH$_2$), 32.7 (CH$_2$), 19.0 (CH$_3$).

HR-MS (ESI): *m*/*z* calcd. for $[C_{30}H_{27}BrO_3 + Na]^+$ 537.1036, found 537.1039.

HPLC separation (Chiralpak® IF-3, *n*-hexane/*i*-PrOH 95:5, 1.0 mL/min, detection at 250 nm): t_r (minor) = 11.2 min, t_r (major) = 15.5 min, 89% e.e.

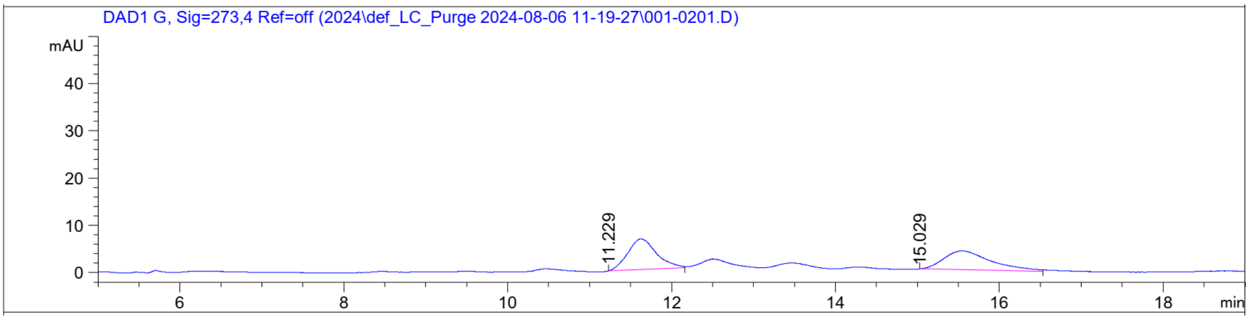

Peak #	RetTime [min]	Type	Width [min]	Area [mAU*s]	Height [mAU]	Area %
1	11.229	PM R	0.4044	156.93057	0.00000	49.5090
2	15.029	PM R	0.6744	160.04327	0.00000	50.4910

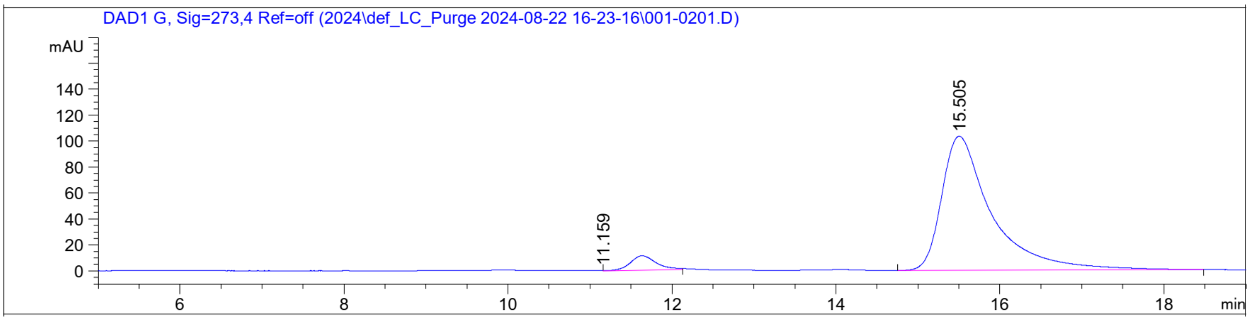

Peak #	RetTime [min]	Type	Width [min]	Area [mAU*s]	Height [mAU]	Area %
1	11.159	MM R	0.3761	253.51451	4.52640e-1	5.4748
2	15.505	BB	0.6033	4377.06250	103.29902	94.5252

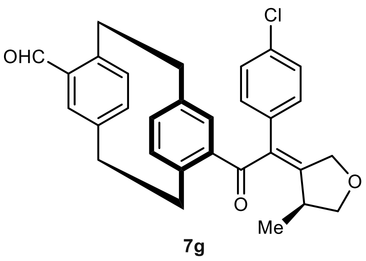

7g

(*S*,*E*)-4^3-(2-(4-chlorophenyl)-2-(4-methyldihydrofuran-3(2H)-ylidene)acetyl)-1,4(1,4)-dibenzenacyclohexaphane-12-carbaldehyde (7g)

The general procedure was followed using bialdehyde **6a** (0.11 mmol, 29.0 mg) and 1,6-enyne **2j** (0.10 mmol, 20.7 mg) at room temperature for 72 h. Purification by column chromatography on silica gel (DCM) yielded **7g** (35.8 mg, 76%, 7:1 d.r., 94% e.e.) as a colorless oil.

^{1}H-NMR (400 MHz, CDCl$_3$) δ 9.92 (s, 1H), 7.32 – 7.29 (m, 2H), 7.21 – 7.15 (m, 2H), 7.01 (d, J = 2.0 Hz, 1H), 6.83 (d, J = 1.9 Hz, 1H), 6.63 (dd, J = 7.8, 2.0 Hz, 1H), 6.46 (dd, J = 7.8, 1.9 Hz, 1H), 6.35 (d, J = 7.8 Hz, 1H), 6.08 (d, J = 7.8 Hz, 1H), 4.52 (dd, J = 15.1, 1.6 Hz, 1H), 4.13 (d, J = 15.1 Hz, 1H), 4.08 – 4.01 (m, 1H), 3.89 (dd, J = 8.5, 5.7 Hz, 1H), 3.79 – 3.72 (m, 1H), 3.63 (dd, J = 8.3, 2.7 Hz, 1H), 3.29 – 3.21 (m, 1H), 3.20 – 2.96 (m, 6H), 0.92 (d, J = 7.0 Hz, 3H).

^{13}C-NMR (101 MHz, CDCl$_3$) δ 197.2 (C$_q$), 192.2 (CH), 152.6 (C$_q$), 142.9 (C$_q$), 141.0 (C$_q$), 140.6 (C$_q$), 139.6 (C$_q$), 138.7 (C$_q$), 136.6 (CH), 136.5 (C$_q$), 136.5 (CH), 135.9 (C$_q$), 135.8 (CH), 135.5 (CH), 135.0 (CH), 134.3 (CH), 133.8 (C$_q$), 133.0 (C$_q$), 129.7 (CH), 128.9 (CH), 75.7 (CH$_2$), 70.3 (CH$_2$), 37.9 (CH), 34.7 (CH$_2$), 34.7 (CH$_2$), 34.5 (CH$_2$), 32.9 (CH$_2$), 18.6 (CH$_3$).

IR (ATR): 1683, 1488, 1266, 1243, 1160, 1092, 736 cm^{-1}.

HR-MS (ESI): m/z calcd. for [$C_{30}H_{27}ClO_3$ + Na]$^+$ 493.1541, found 493.1532.

[α_D^{20}] = +48.5 (c = 1.0, DCM).

HPLC separation (Chiralpak® IF-3, n-hexane/i-PrOH 90:10, 1.0 mL/min, detection at 250 nm): t_r (major) = 13.6 min, t_r (minor) = 16.2 min, 94% e.e.

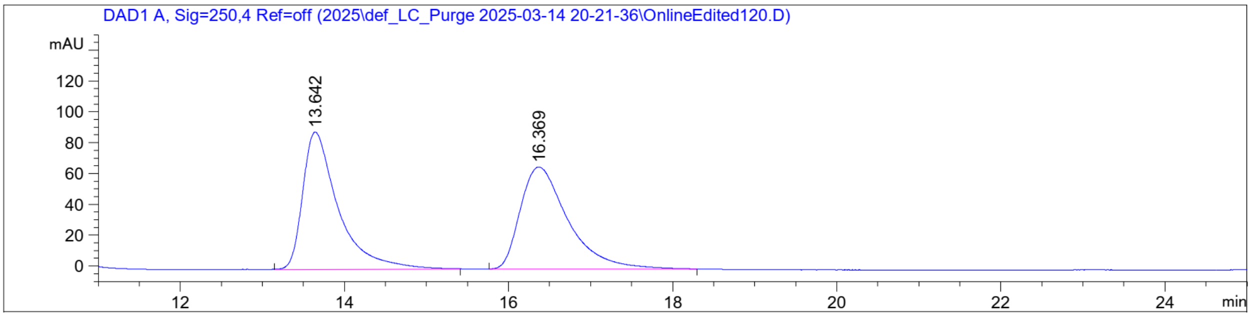

Peak #	RetTime [min]	Type	Width [min]	Area [mAU*s]	Height [mAU]	Area %
1	13.642	BB	0.4278	2669.92896	89.04737	49.9610
2	16.369	BB	0.5496	2674.09375	66.15205	50.0390

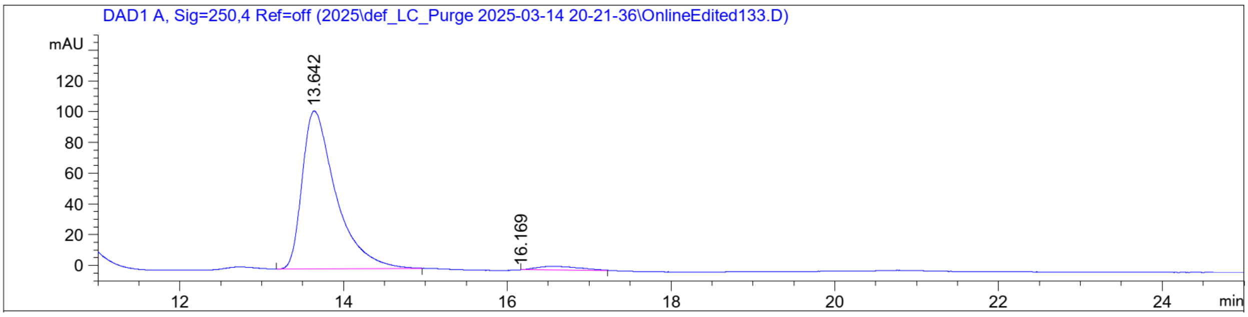

Peak #	RetTime [min]	Type	Width [min]	Area [mAU*s]	Height [mAU]	Area %
1	13.642	BB	0.4181	2934.30444	102.78309	97.1609
2	16.169	PM R	0.6104	85.74126	0.00000	2.8391

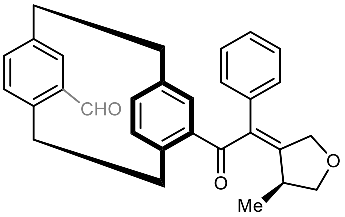

7h

(*S*,*E*)-4[3]-(2-(4-chlorophenyl)-2-(4-methyldihydrofuran-3(2H)-ylidene)acetyl)-1,4(1,4)-dibenzenacyclohexaphane-12-carbaldehyde (7h)

The general procedure was followed using bialdehyde **6b** (0.11 mmol, 29.0 mg) and 1,6-enyne **2f** (0.10 mmol, 17.2 mg) at room temperature for 72 h. Purification by column chromatography on silica gel (DCM) yielded **7h** (29.2 mg, 67%, 4:1 d.r., >99% e.e.) as a colorless oil.

^{1}H-NMR (400 MHz, $CDCl_3$) δ 9.93 (s, 1H), 7.19 – 7.14 (m, 2H), 6.98 – 6.95 (m, 2H), 6.91 (d, *J* = 2.1 Hz, 1H), 6.74 – 6.64 (m, 2H), 6.56 (d, *J* = 7.8 Hz, 3H), 6.40 (d, *J* = 7.5 Hz, 1H), 4.41 (dd, *J* = 15.7, 1.6 Hz, 1H), 4.22 – 4.16 (m, 1H), 4.08 (d, *J* = 15.7 Hz, 1H), 3.88 (dd, *J* = 8.4, 5.4 Hz, 1H), 3.71 (dd, *J* = 8.4, 2.0 Hz, 1H), 3.52 – 3.45 (m, 1H), 3.28 – 3.21 (m, 1H), 3.14 – 3.02 (m, 6H), 1.13 (d, *J* = 6.9 Hz, 3H).

^{13}C-NMR (101 MHz, $CDCl_3$) δ 197.2 (C_q), 191.2 (CH), 156.7 (C_q), 143.0 (C_q), 140.1 (C_q), 139.6 (C_q), 139.3 (C_q), 138.6 (C_q), 137.9 (C_q), 137.5 (CH), 136.3 (C_q), 135.9 (CH), 135.7 (CH), 135.3 (CH), 134.6 (CH), 133.3 (C_q), 132.0 (CH), 128.9 (CH), 128.2 (CH), 127.3 (CH), 76.1 (CH_2), 70.9 (CH_2), 38.1 (CH), 34.9 (CH_2), 34.7 (CH_2), 34.4 (CH_2), 33.0 (CH_2), 19.2 (CH_3).

IR (ATR): 1683, 1655, 1486, 1232, 1161, 735, 646 cm^{-1}.

HRMS (ESI): *m/z* $[M+Na]^+$ calculated for $C_{30}H_{28}NaO_3^+$: 459.1931, found 459.1926.

[α]^{D}$_{20}$ = +20.6 (c = 1.0, DCM).

HPLC Separation (IA-3 column, *n*-hexane/*i*-PrOH 90/10, 1.0 mL/min, 250.0 nm): t_r(major) = 8.9 min, >99% e.e.

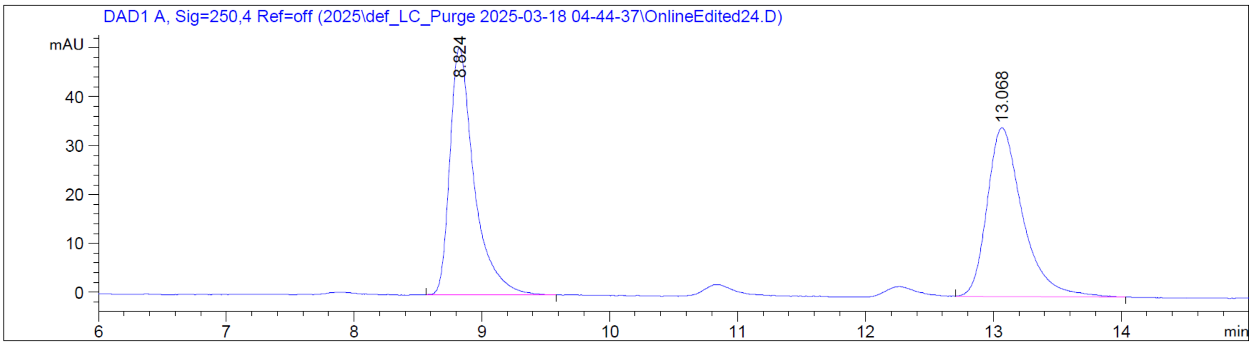

Peak #	RetTime [min]	Type	Width [min]	Area [mAU*s]	Height [mAU]	Area %
1	8.824	BB	0.1941	666.71906	50.48392	50.0259
2	13.068	BB	0.2794	666.02753	34.47280	49.9741

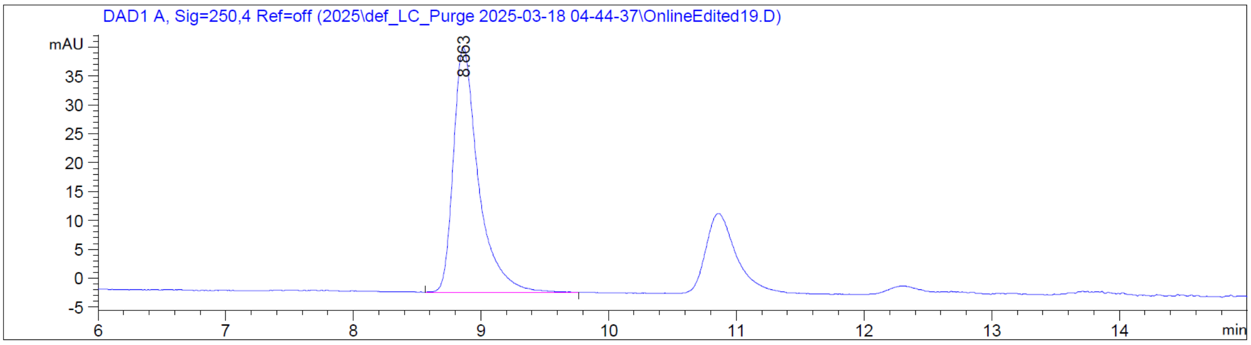

Peak #	RetTime [min]	Type	Width [min]	Area [mAU*s]	Height [mAU]	Area %
1	8.863	MM R	0.2278	579.60022	42.40483	100.0000

The construction of 1,5-central/ferrocene planar chirality

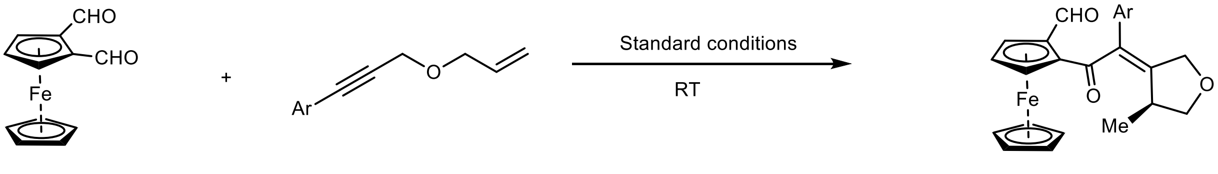

8a **2** **9**

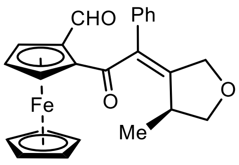

9a

The general procedure was followed using bialdehyde **8a** (0.11 mmol, 26.6 mg) and 1,6-enyne **2f** (0.10 mmol, 17.2 mg) at room temperature for 72 h. Purification by column chromatography on silica gel (DCM/acetone: 20/1) yielded **9a** (23.2 mg, 56%, >20:1 d.r., >99% e.e.) as a red solid.

^{1}H-NMR (300 MHz, CDCl$_3$) δ 10.78 (s, 1H), 7.48 – 7.40 (m, 4H), 7.36 – 7.30 (m, 1H), 5.29 (dd, J = 2.8, 1.5 Hz, 1H), 4.86 (dd, J = 2.8, 1.5 Hz, 1H), 4.78 (t, J = 2.8 Hz, 1H), 4.52 (d, J = 14.7 Hz, 1H), 4.33 (dd, J = 14.8, 1.7 Hz, 1H), 4.00 – 3.90 (m, 6H), 3.61 (dd, J = 8.6, 3.8 Hz, 1H), 3.08 – 2.97 (m, 1H), 1.12 (d, J = 7.0 Hz, 3H).

^{13}C-NMR (75 MHz, CDCl$_3$) δ 201.2 (C$_q$), 195.6 (CH), 147.9 (C$_q$), 137.8 (C$_q$), 133.5 (C$_q$), 128.8 (CH), 128.2 (CH), 127.9 (CH), 81.3 (C$_q$), 80.2 (C$_q$), 77.0 (CH), 76.0 (CH$_2$), 74.7 (CH), 73.0 (CH), 72.1 (CH), 70.6 (CH$_2$), 37.5 (CH), 19.0 (CH$_3$).

IR (ATR): 1667, 1640, 1440, 1417, 1334, 1262, 1203, 1089, 821, 702 cm^{-1}.

HR-MS (ESI): m/z calcd. for $[C_{24}H_{22}FeO_3 + Na]^+$ 437.0811, found 437.0820.

$[\alpha_D^{20}]$ = +73.3 (c = 0.03, DCM).

HPLC separation (Chiralpak® IE-3, MeOH/i-PrOH 90:10, 0.5 mL/min, detection at 273 nm): t_r (major) = 45.7 min, >99% e.e.

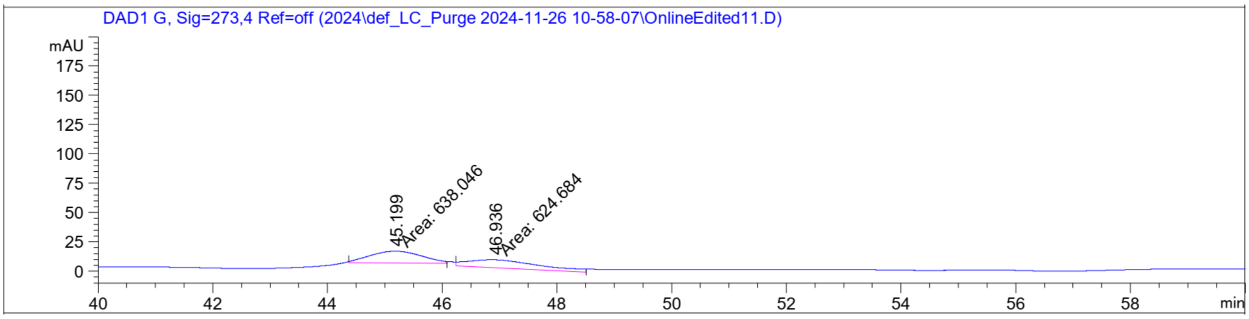

Peak #	RetTime [min]	Type	Width [min]	Area [mAU*s]	Height [mAU]	Area %
1	45.199	MM	1.0547	638.04553	10.08226	50.5291
2	46.936	MM	1.4935	624.68365	6.97094	49.4709

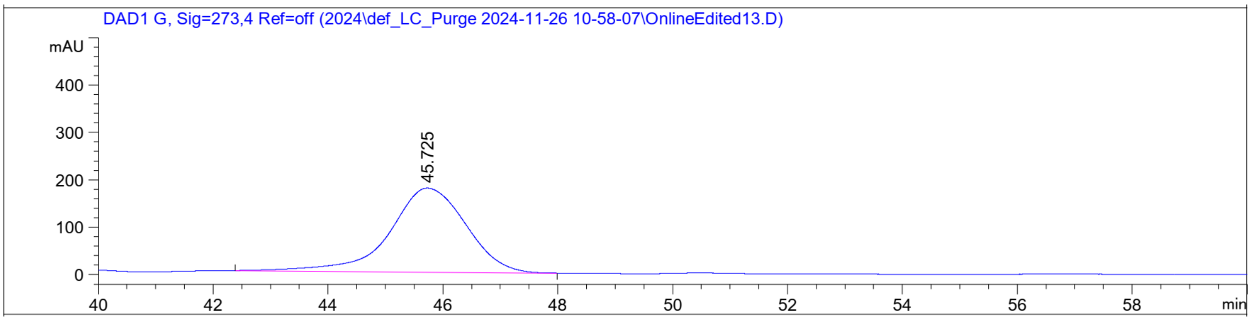

Peak #	RetTime [min]	Type	Width [min]	Area [mAU*s]	Height [mAU]	Area %
1	45.725	BB	1.0963	1.63320e4	177.19987	100.0000

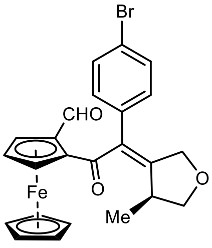

9b

The general procedure was followed using bialdehyde **8a** (0.11 mmol, 26.6 mg) and 1,6-enyne **2k** (0.10 mmol, 25.1 mg) at room temperature for 72 h. Purification by column chromatography on silica gel (DCM/acetone: 20/1) yielded **9b** (24.7 mg, 50%, >20:1 d.r., >99% e.e.) as a red solid.

^{1}H-NMR (300 MHz, $CDCl_3$) δ 10.76 (s, 1H), 7.57 (d, *J* = 8.1 Hz, 2H), 7.33 (d, *J* = 8.1 Hz, 2H), 5.31 (s, 1H), 4.81 (d, *J* = 12.1 Hz, 2H), 4.46 (d, *J* = 14.9 Hz, 1H), 4.30 (d, *J* = 14.9 Hz, 1H), 4.11 – 3.87 (m, 6H), 3.60 (dd, *J* = 8.6, 3.9 Hz, 1H), 3.14 – 2.93 (m, 1H), 1.10 (d, *J* = 7.0 Hz, 3H).

^{13}C-NMR (101 MHz, $CDCl_3$) δ 200.7 (C_q), 195.5 (CH), 149.0 (C_q), 136.7 (C_q), 132.4 (C_q), 132.0 (CH), 129.6 (CH), 122.3 (C_q), 81.5 (C_q), 80.7 (C_q), 76.8 (CH), 76.0 (CH_2), 74.8 (CH), 73.2 (CH), 72.1 (CH), 70.6 (CH_2), 37.6 (CH), 18.9 (CH_3).

IR (ATR): 1668, 1643, 1417, 1332, 1263, 1073, 828, 733 cm^{-1}.

HR-MS (ESI): *m*/*z* calcd. for $[C_{24}H_{21}BrFeO_3 + Na]^+$ 514.9916, found 514.9924.

[α^{20}_{D}] = +68.5 (c = 0.03, DCM).

HPLC separation (Chiralpak® IE-3, MeOH/*i*-PrOH 90:10, 0.5 mL/min, detection at 273 nm): t_r (major) = 23.9 min, >99% e.e.

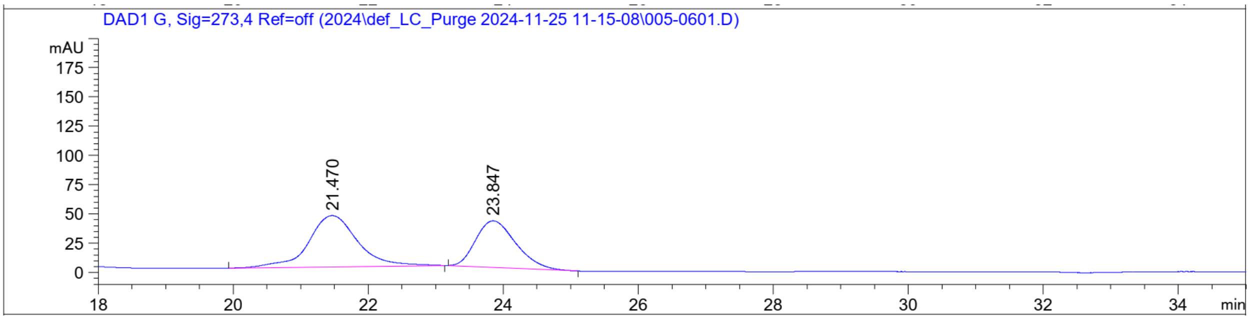


```
Peak RetTime Type  Width      Area       Height      Area
  #    [min]         [min]    [mAU*s]      [mAU]        %
----|-------|----|-------|----------|----------|--------|
   1  21.470 BB     0.6031 2217.36353   43.83839   57.4207
   2  23.847 BB     0.5611 1644.24487   39.71043   42.5793
```

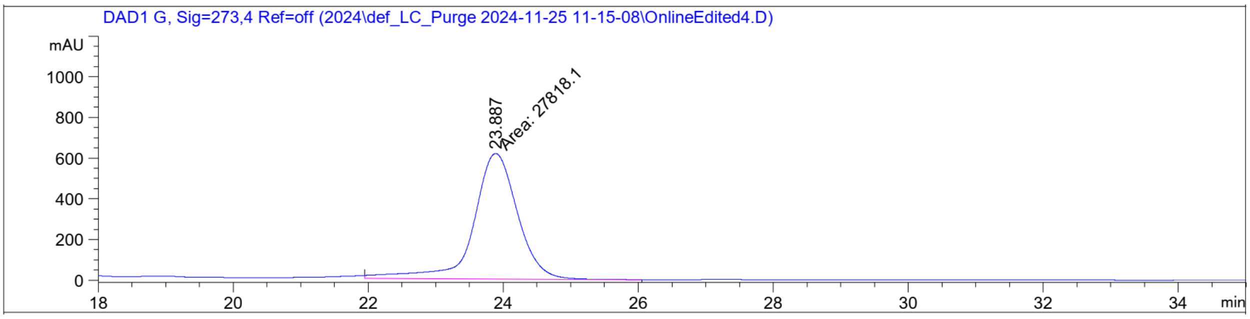


```
Peak RetTime Type  Width      Area       Height      Area
  #    [min]         [min]    [mAU*s]      [mAU]        %
----|-------|----|-------|----------|----------|--------|
   1  23.887 FM     0.7526 2.78181e4   616.01080 100.0000
```

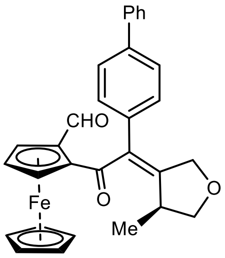

9c

The general procedure was followed using bialdehyde **8a** (0.11 mmol, 26.6 mg) and 1,6-enyne **2g** (0.10 mmol, 24.8 mg) at room temperature for 72 h. Purification by column chromatography on silica gel (DCM/acetone: 20/1) yielded **9c** (28.9 mg, 61%, >20:1 d.r., >99% e.e.) as a red solid.

^{1}H-NMR (300 MHz, CDCl$_3$) δ 10.80 (s, 1H), 7.73 – 7.29 (m, 9H), 5.30 (d, J = 3.4 Hz, 1H), 4.85 (d, J = 31.4 Hz, 2H), 4.58 (d, J = 14.8 Hz, 1H), 4.40 (d, J = 14.7 Hz, 1H), 4.18 – 3.80 (m, 6H), 3.63 (dd, J = 8.6, 3.7 Hz, 1H), 3.14 – 2.93 (m, 1H), 1.14 (d, J = 6.9 Hz, 3H).

^{13}C-NMR (75 MHz, CDCl$_3$) δ 201.3 (C_q), 195.6 (CH), 148.0 (C_q), 140.9 (C_q), 140.3 (C_q), 136.7

(C_q), 133.2 (C_q), 129.0 (CH), 128.3 (CH), 127.8 (CH), 127.4 (CH), 127.1 (CH), 81.4 (C_q), 80.3 (C_q), 77.1 (CH), 76.0 (CH_2), 74.7 (CH), 73.0 (CH), 72.1 (CH), 70.7 (CH_2), 37.6 (CH), 19.1 (CH_3).

IR (ATR): 1668, 1639, 1486, 1439, 1417, 1334, 1262, 1203, 1088, 1006, 819, 734 cm^{-1}.

HR-MS (ESI): *m*/*z* calcd. for $[C_{30}H_{26}FeO_3 + Na]^+$ 513.1124, found 513.1119.

$[\alpha^{20}_{D}]$ = +82.0 (c = 0.03, DCM).

HPLC separation (Chiralpak® IE-3, MeOH/*i*-PrOH 90:10, 0.5 mL/min, detection at 273 nm): t_r (major) = 30.9 min, >99% e.e.

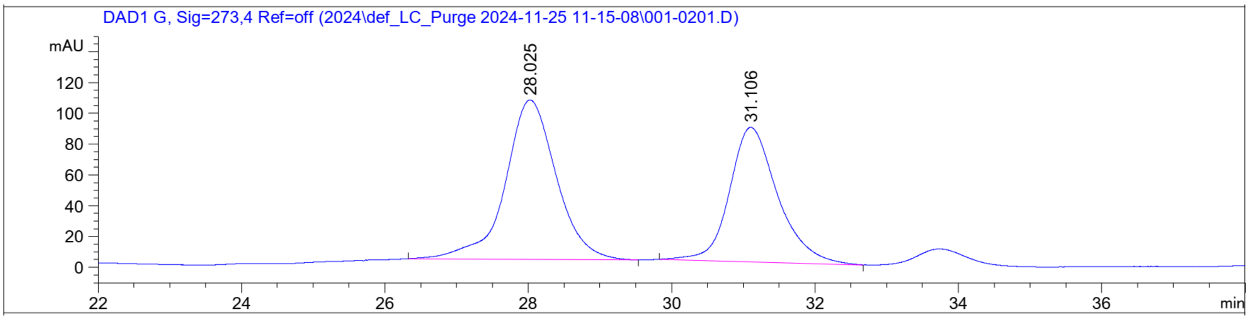

Peak #	RetTime [min]	Type	Width [min]	Area [mAU*s]	Height [mAU]	Area %
1	28.025	BB	0.6882	5121.50781	103.52248	55.6141
2	31.106	BB	0.6707	4087.49414	87.38250	44.3859

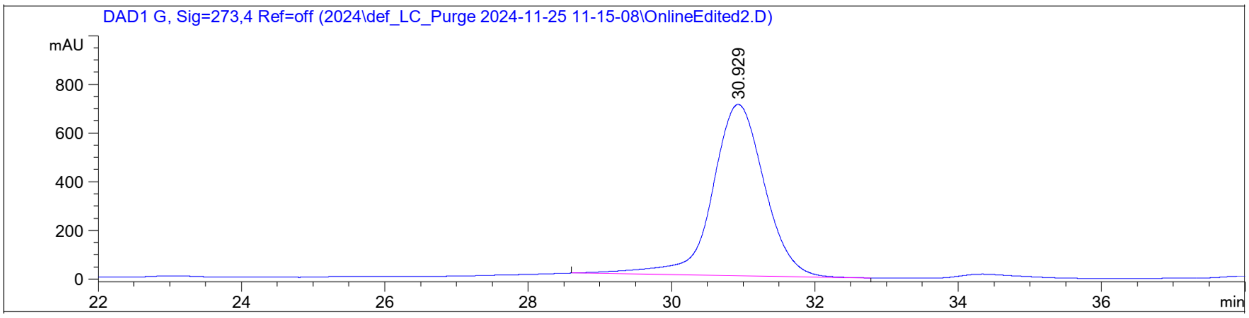

Peak #	RetTime [min]	Type	Width [min]	Area [mAU*s]	Height [mAU]	Area %
1	30.929	BB	0.7630	3.56047e4	703.99670	100.0000

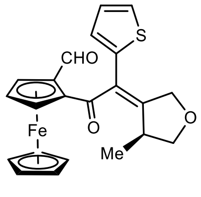

9d

The general procedure was followed using bialdehyde **8a** (0.11 mmol, 26.6 mg) and 1,6-enyne **2m** (0.10 mmol, 17.8 mg) at room temperature for 72 h. Purification by column chromatography on silica gel (DCM/acetone: 20/1) yielded **9d** (22.3 mg, 53%, 19:1 d.r., >99% e.e.) as a red solid.

^{1}H-NMR (300 MHz, $CDCl_3$) δ 10.80 (s, 1H), 7.34 (dd, J = 5.0, 1.3 Hz, 1H), 7.15 – 7.05 (m, 2H), 5.34 (dd, J = 2.8, 1.5 Hz, 1H), 4.92 (dd, J = 2.8, 1.5 Hz, 1H), 4.85 – 4.80 (m, 1H), 4.71 (d, J = 15.2 Hz, 1H), 4.59 (dd, J = 15.1, 1.8 Hz, 1H), 4.15 – 4.06 (m, 5H), 3.92 (dd, J = 8.6, 6.0 Hz, 1H), 3.62 (dd, J = 8.6, 3.4 Hz, 1H), 3.01 – 2.91 (m, 1H), 1.06 (d, J = 7.0 Hz, 3H).

^{13}C-NMR (75 MHz, $CDCl_3$) δ 201.1 (C_q), 195.4 (CH), 148.0 (C_q), 138.8 (C_q), 127.2 (CH), 127.1 (C_q), 126.4 (CH), 125.9 (CH), 81.4 (C_q), 79.8 (C_q), 77.0 (CH), 75.9 (CH_2), 74.9 (CH), 73.2 (CH), 72.3 (CH), 71.4 (CH_2), 38.2 (CH), 19.1 (CH_3).

IR (ATR): 1667, 1439, 1417, 1332, 1261, 1204, 1108, 834, 703 cm^{-1}.

HR-MS (ESI): m/z calcd. for $[C_{22}H_{20}FeO_3S + Na]^+$ 443.0375, found 443.0364.

$[\alpha^{20}_{D}]$ = +59.6 (c = 0.03, DCM).

HPLC separation (Chiralpak® IE-3, MeOH/i-PrOH 90:10, 0.2 mL/min, detection at 273 nm): t_r (major) = 46.9 min, >99% e.e.

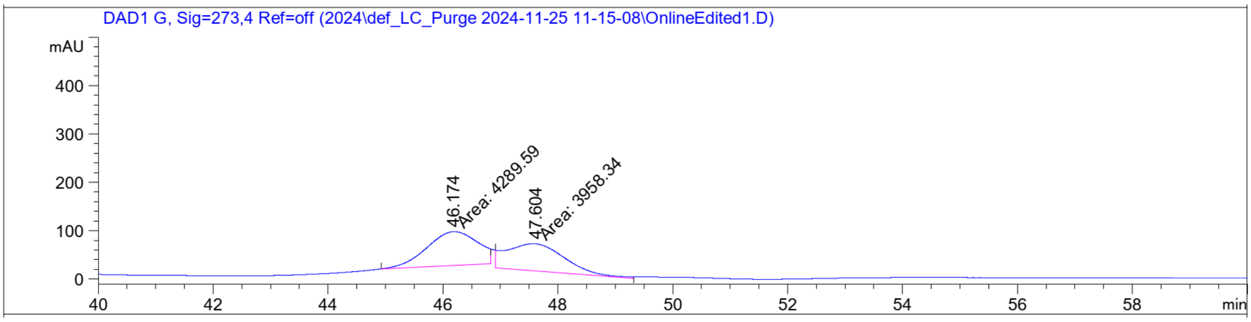

Peak #	RetTime [min]	Type	Width [min]	Area [mAU*s]	Height [mAU]	Area %
1	46.174	MM	1.0252	4289.59277	69.73347	52.0081
2	47.604	MM	1.1724	3958.34448	56.26943	47.9919

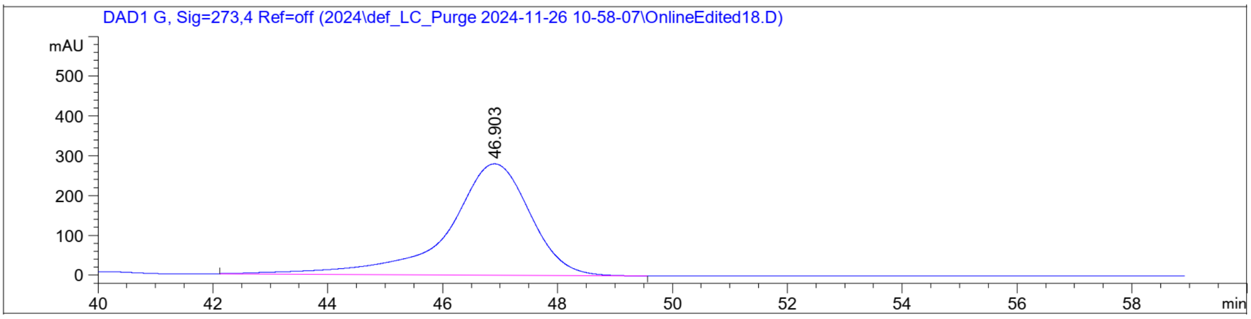

Peak #	RetTime [min]	Type	Width [min]	Area [mAU*s]	Height [mAU]	Area %
1	46.903	BB	1.2029	3.31815e4	335.19977	100.0000

Scope of monosubstituted aldehydes

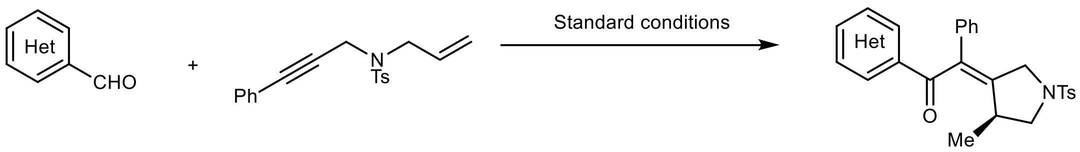

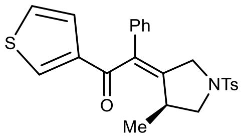

10a

The general procedure was followed using indole-3-carbaldehyde (0.11 mmol, 16.0 mg) and 1,6-enyne **2a** (0.10 mmol, 32.5 mg) at 40 °C for 24 h. Purification by column chromatography on silica gel (DCM) yielded **10a** (35.9 mg, 82%, 99% e.e.) as a colorless oil.

^{1}H-NMR (300 MHz, $CDCl_3$) δ 7.74 (dd, *J* = 2.9, 1.2 Hz, 1H), 7.68 – 7.61 (m, 2H), 7.41 (dd, *J* = 5.1, 1.2 Hz, 1H), 7.32 (dq, *J* = 6.8, 5.7, 4.9 Hz, 5H), 7.23 – 7.14 (m, 3H), 4.24 (dd, *J* = 15.4, 1.5 Hz, 1H), 3.80 (d, *J* = 15.4 Hz, 1H), 3.34 – 3.24 (m, 1H), 3.14 (ddt, *J* = 9.1, 7.7, 2.9 Hz, 2H), 2.43 (s, 3H), 1.00 (d, *J* = 6.9 Hz, 3H).

^{13}C-NMR (75 MHz, $CDCl_3$) δ 189.7 (C_q), 144.3 (C_q), 143.9 (C_q), 141.9 (C_q), 136.2 (C_q), 135.5 (C_q), 134.9 (CH), 132.7 (C_q), 129.8 (CH), 129.0 (CH), 128.2 (CH), 128.0 (CH), 127.8 (CH), 127.5 (CH), 126.5 (CH), 54.6 (CH_2), 50.4 (CH_2), 36.5 (CH), 21.6 (CH_3), 19.5 (CH_3).

HR-MS (ESI): *m*/*z* calcd. for $[C_{24}H_{23}NO_3S_2 + Na]^+$ 460.1012, found 460.1008.

$[\alpha_D^{20}]$ = +172.9 (c = 1, DCM).

HPLC separation (Chiralpak® IC-3, *n*-hexane/*i*-PrOH 60:40, 1.0 mL/min, detection at 273 nm): t_r (minor) = 18.3 min, t_r (major) = 20.2 min, 99% e.e.

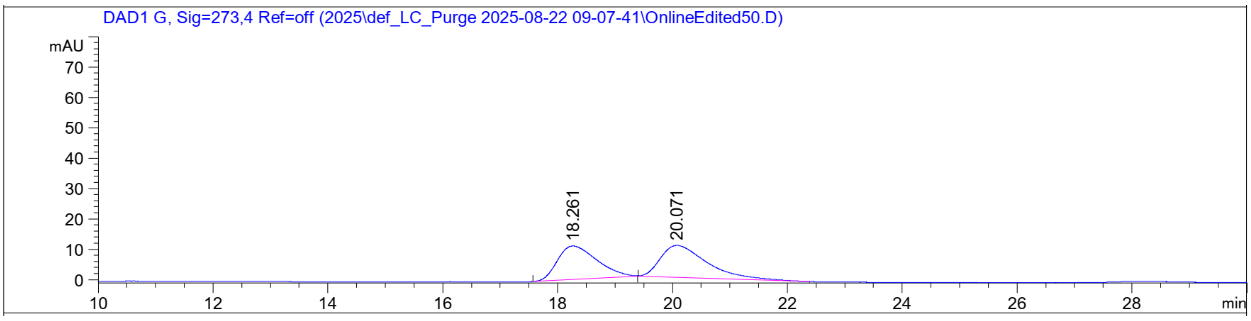

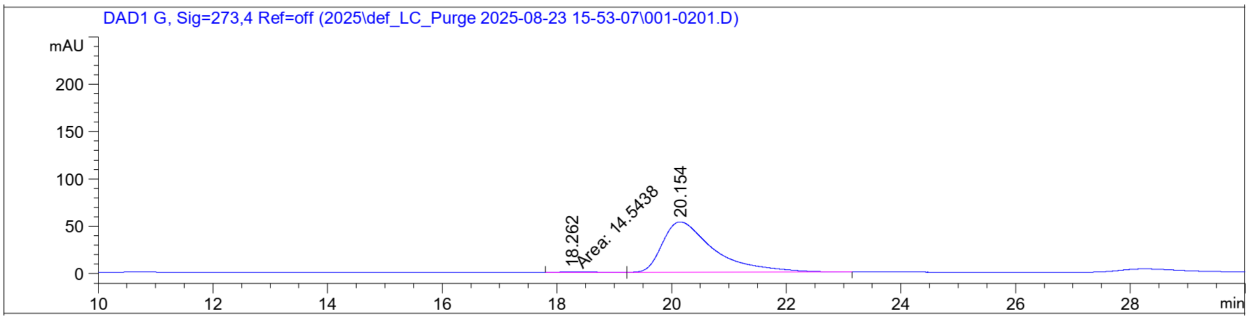

Peak #	RetTime [min]	Type	Width [min]	Area [mAU*s]	Height [mAU]	Area %
1	18.262	MM	0.8091	14.54382	2.99592e-1	0.4386
2	20.154	BB	0.8660	3301.17578	53.12124	99.5614

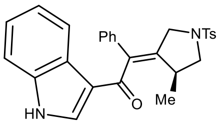

10b

The general procedure was followed using thiophene-3-carbaldehyde (0.11 mmol, 12.3 mg) and 1,6-enyne **2a** (0.10 mmol, 32.5 mg) at 40 °C for 24 h. Purification by column chromatography on silica gel (DCM) yielded **10b** (37.2 mg, 79%, >99% e.e.) as a white solid.

^{1}H-NMR (300 MHz, $CDCl_3$) δ 8.84 (s, 1H), 8.47 – 8.35 (m, 1H), 7.68 (d, *J* = 8.2 Hz, 2H), 7.47 (d, *J* = 2.9 Hz, 1H), 7.43 – 7.24 (m, 10H), 4.25 (dd, *J* = 15.0, 1.4 Hz, 1H), 3.84 (d, *J* = 15.0 Hz, 1H), 3.34 – 3.17 (m, 2H), 3.13 – 3.03 (m, 1H), 2.46 (s, 3H), 1.00 (d, *J* = 6.6 Hz, 3H).

^{13}C-NMR (75 MHz, $CDCl_3$) δ 191.1 (C_q), 143.9 (C_q), 142.1 (C_q), 137.1 (C_q), 136.5 (C_q), 136.3 (C_q), 134.2 (CH), 132.6 (C_q), 129.8 (CH), 128.8 (CH), 127.9 (CH), 127.9 (CH), 127.7 (CH),

125.6 (C_q), 124.1 (CH), 123.0 (CH), 122.3 (CH), 117.9 (C_q), 111.6 (CH), 54.5 (CH_2), 50.3 (CH_2), 36.6 (CH), 21.6 (CH_3), 19.5 (CH_3).

HR-MS (ESI): *m*/*z* calcd. for $[C_{28}H_{26}N_2O_3S + Na]^+$ 493.1556, found 493.1553.

$[\alpha^{20}_{D}]$ = +279.0 (c = 0.5, DCM).

HPLC separation (Chiralpak® IA-3, *n*-hexane/*i*-PrOH 80:20, 1.0 mL/min, detection at 273 nm): t_r (major) = 21.5 min, >99% e.e.

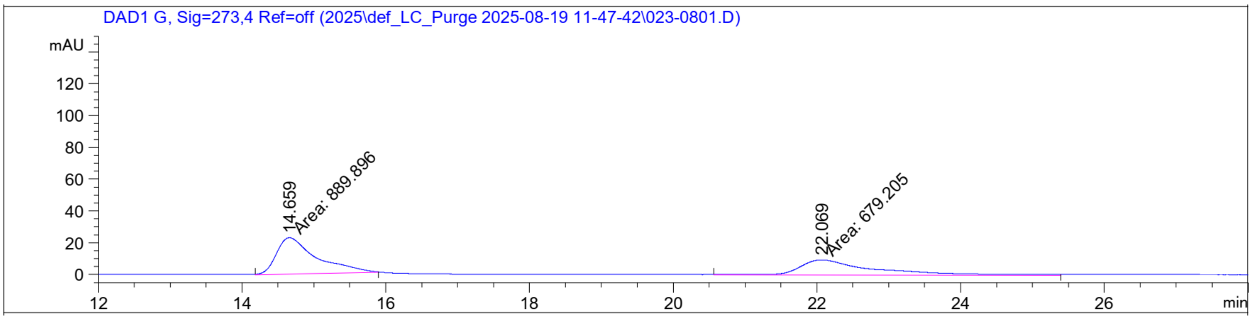

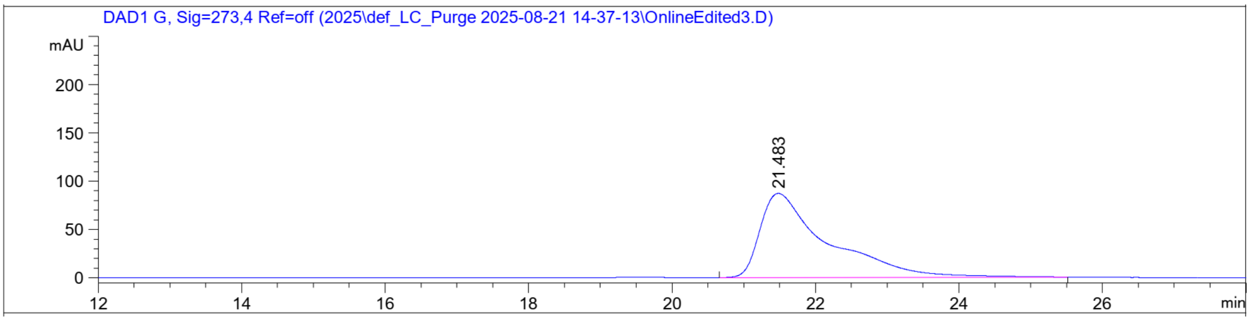

Peak #	RetTime [min]	Type	Width [min]	Area [mAU*s]	Height [mAU]	Area %
1	21.483	BB	0.8994	5671.56738	87.01391	100.0000

Supplementary Section 4. X-Ray Analysis

General Data Acquisition and Processing

The data were integrated with SAINT[6]. A multi-scan absorption correction was applied using SADABS[7]. The structure was solved by SHELXT[8] and refined on F^2 using SHELXL[9] in the graphical user interface ShelXle[10].

X-Ray Analysis of 3a

A solution of compound **3a** (20 mg) was dissolved in DCM (0.3 mL) in a glass tube, then layed with *n*-hexane (2.5 mL). The solution was kept standing. Single crystals of **3a** were obtained by slow diffusion at room temperature for X-Ray crystallographic analysis. A colourless, plank-shaped crystal of **3a** was mounted on a MiTeGen micromount with perfluoroether oil. Data were collected from a shock-cooled single crystal at 100.00 K on a Bruker D8 VENTURE dual wavelength Mo/Ag four-circle diffractometer with a microfocus sealed X-ray tube using a mirror optics as monochromator and a Bruker PHOTON III detector. The diffractometer was equipped with an Oxford Cryostream 800 low temperature device and used MoKα radiation (λ = 0.71073 Å).

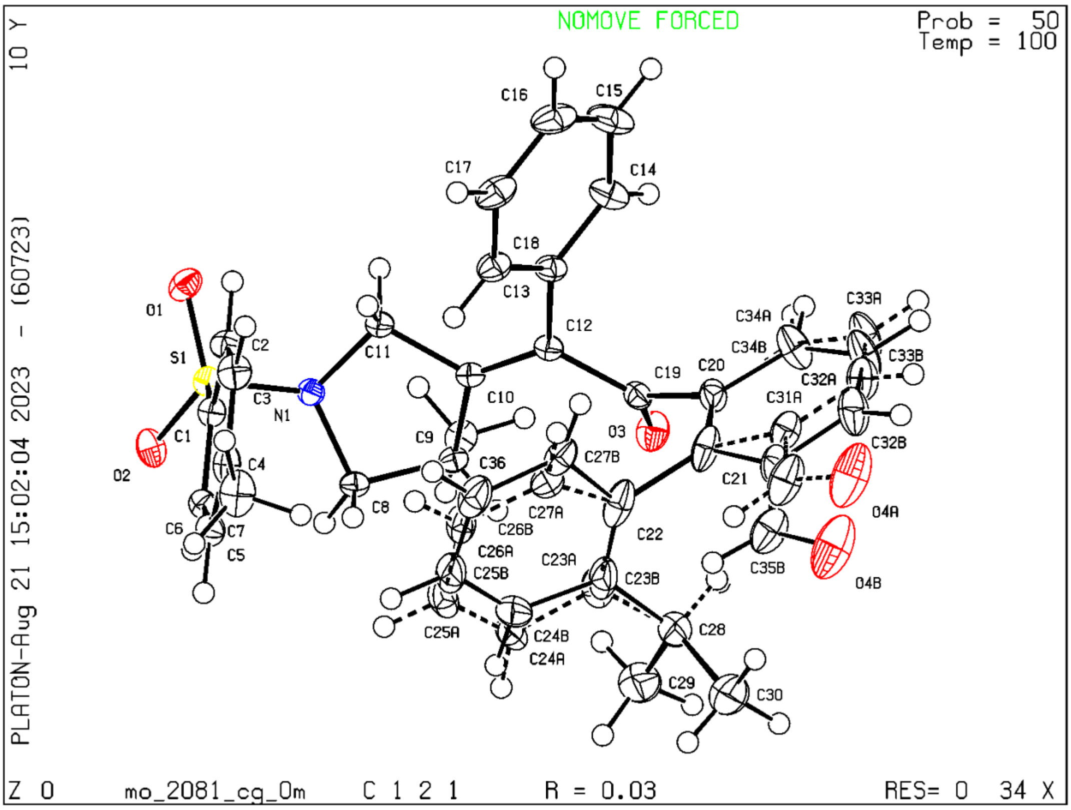

Supplementary Figure 2. Structure of compound **3a** (CCDC 2378811).

CheckCIF analysis: No A- or B-level alerts were generated for this structure.

Supplementary Table 2. Crystal data and structure refinement for **3a**.

CCDC number	**2378811**
Empirical formula	$C_{36}H_{35}NO_4S$
Formula weight	577.71
Temperature [K]	100.00
Crystal system	monoclinic
Space group (number)	$C2$ (5)
a [Å]	39.257(5)
b [Å]	8.2784(11)
c [Å]	9.2737(12)
α [°]	90
β [°]	99.738(5)
γ [°]	90
Volume [Å^3]	2970.4(7)
Z	4
ρ_{calc} [gcm^{-3}]	1.292
μ [mm^{-1}]	0.150
F(000)	1224
Crystal size [mm^3]	0.925×0.613×0.014
Crystal colour	colourless
Crystal shape	plank
Radiation	MoK_α (λ=0.71073 Å)
2θ range [°]	4.21 to 61.09 (0.70 Å)
Index ranges	$-56 \le h \le 56$<br>$-11 \le k \le 11$<br>$-13 \le l \le 13$
Reflections collected	133622
Independent reflections	9072<br>R_{int} = 0.0336<br>R_{sigma} = 0.0128
Completeness to θ = 25.242°	100.0 %
Data / Restraints / Parameters	9072/224/483
Goodness-of-fit on F^2	1.049
Final R indexes [$I \geq 2\sigma(I)$]	R_1 = 0.0271<br>wR_2 = 0.0707

Final *R* indexes [all data]	R_1 = 0.0284<br>wR_2 = 0.0719
Largest peak/hole [eÅ^{-3}]	0.29/-0.20
Flack X parameter	-0.002(9)

Supplementary Table 3. Atomic coordinates and Ueq [Å2] for **3a**.

Atom	*x*	*y*	*z*	U_{eq}
S1	0.25651(2)	0.61646(4)	0.66224(3)	0.01633(7)
O1	0.25134(3)	0.75777(13)	0.74591(12)	0.0234(2)
O2	0.22900(3)	0.55621(14)	0.55332(11)	0.0229(2)
O3	0.40575(3)	0.68950(15)	0.43239(11)	0.0263(2)
O4A	0.52293(8)	0.2449(6)	0.9039(5)	0.0511(14)
O4B	0.51359(9)	0.1444(6)	0.8355(4)	0.0528(14)
N1	0.28856(3)	0.65851(13)	0.57771(11)	0.01577(19)
C1	0.27043(3)	0.45873(15)	0.78601(14)	0.0164(2)
C2	0.28567(4)	0.49310(17)	0.92976(14)	0.0205(2)
H2	0.287773	0.601508	0.963691	0.025
C3	0.29776(4)	0.3659(2)	1.02266(15)	0.0235(3)
H3	0.308211	0.388555	1.120602	0.028
C4	0.29485(3)	0.20599(18)	0.97503(15)	0.0216(3)
C5	0.27904(3)	0.17480(17)	0.83159(15)	0.0204(2)
H5	0.276477	0.066284	0.798196	0.025
C6	0.26696(3)	0.29993(16)	0.73654(14)	0.0180(2)
H6	0.256440	0.277210	0.638700	0.022
C7	0.30885(4)	0.0694(2)	1.07492(18)	0.0306(3)
H7A	0.293315	-0.023709	1.056764	0.046
H7B	0.310525	0.103945	1.176966	0.046
H7C	0.331833	0.039211	1.056071	0.046
C8	0.30026(3)	0.53892(16)	0.47733(13)	0.0168(2)
H8A	0.282844	0.524285	0.387750	0.020
H8B	0.305310	0.433036	0.525863	0.020
C9	0.33325(3)	0.61876(17)	0.44280(12)	0.01575(19)
H9	0.348989	0.536248	0.410606	0.019
C10	0.34869(3)	0.68735(15)	0.59071(13)	0.0154(2)
C11	0.31896(3)	0.73715(17)	0.66536(14)	0.0201(2)
H11A	0.322542	0.698794	0.767929	0.024

H11B	0.316246	0.856055	0.664417	0.024
C12	0.38220(3)	0.69959(15)	0.65257(13)	0.0161(2)
C13	0.39298(3)	0.75104(16)	0.80792(14)	0.0187(2)
C14	0.41456(4)	0.88377(19)	0.84215(17)	0.0264(3)
H14	0.422651	0.941638	0.766184	0.032
C15	0.42443(5)	0.9327(2)	0.98765(19)	0.0340(4)
H15	0.439193	1.023467	1.009999	0.041
C16	0.41279(5)	0.8495(2)	1.09882(17)	0.0315(3)
H16	0.419371	0.883629	1.197396	0.038
C17	0.39155(4)	0.7164(2)	1.06685(15)	0.0280(3)
H17	0.383747	0.658532	1.143489	0.034
C18	0.38162(4)	0.66751(18)	0.92234(14)	0.0222(3)
H18	0.366948	0.576298	0.900962	0.027
C19	0.40999(3)	0.66597(16)	0.56427(14)	0.0177(2)
C20	0.44492(3)	0.6098(2)	0.64049(14)	0.0221(2)
C21	0.44938(4)	0.4540(2)	0.69827(15)	0.0271(3)
C22	0.41842(4)	0.3457(2)	0.6861(2)	0.0316(4)
C23A	0.4018(4)	0.274(2)	0.5445(16)	0.0284(17)
C24A	0.36972(16)	0.1901(9)	0.5230(6)	0.0235(10)
H24A	0.360194	0.146385	0.430224	0.028
C25A	0.35293(14)	0.1743(8)	0.6412(6)	0.0267(9)
H25A	0.330860	0.124158	0.629069	0.032
C26A	0.36806(14)	0.2310(8)	0.7776(7)	0.0250(10)
H26A	0.356904	0.216539	0.859966	0.030
C27A	0.39987(16)	0.3096(8)	0.7925(7)	0.0223(10)
H27A	0.409740	0.341717	0.888877	0.027
C28	0.42194(4)	0.2783(2)	0.4215(2)	0.0336(3)
H28A	0.436741	0.377031	0.424641	0.040
H28	0.436177	0.379151	0.429863	0.040
C29	0.39815(5)	0.2774(3)	0.2724(2)	0.0436(4)
H29A	0.385493	0.174957	0.259554	0.065
H29B	0.412035	0.289613	0.194652	0.065
H29C	0.381697	0.367073	0.267669	0.065
C30	0.44627(5)	0.1317(3)	0.4343(3)	0.0472(5)
H30A	0.462456	0.137158	0.527007	0.071
H30B	0.459204	0.132161	0.352704	0.071
H30C	0.432651	0.032129	0.431329	0.071

C31A	0.48530(12)	0.4363(6)	0.7740(5)	0.0238(8)
C31B	0.48085(11)	0.3796(7)	0.7540(5)	0.0245(8)
C32A	0.51157(12)	0.5483(8)	0.7714(6)	0.0288(9)
H32A	0.534422	0.524274	0.818455	0.035
C32B	0.50967(12)	0.4796(9)	0.7716(6)	0.0343(11)
H32B	0.531461	0.438573	0.817041	0.041
C33A	0.50466(14)	0.6922(7)	0.7016(8)	0.0357(11)
H33A	0.522644	0.768681	0.700258	0.043
C33B	0.50711(13)	0.6339(10)	0.7250(7)	0.0395(12)
H33B	0.527203	0.699745	0.735780	0.047
C34A	0.47107(16)	0.7272(10)	0.6318(11)	0.0287(12)
H34A	0.465842	0.825858	0.580489	0.034
C34B	0.47505(19)	0.6985(11)	0.6607(11)	0.0350(14)
H34B	0.474087	0.808095	0.629843	0.042
C35A	0.49367(11)	0.2786(7)	0.8447(5)	0.0361(11)
H35A	0.475794	0.201082	0.844114	0.043
C35B	0.48552(10)	0.2064(7)	0.7967(4)	0.0328(9)
H35B	0.465489	0.141177	0.793297	0.039
C36	0.32507(4)	0.75294(18)	0.32782(14)	0.0208(2)
H36A	0.309779	0.832861	0.361663	0.031
H36B	0.313625	0.706055	0.235156	0.031
H36C	0.346596	0.805547	0.313253	0.031
C25B	0.35937(17)	0.1887(7)	0.6961(9)	0.0355(13)
H25B	0.337779	0.135742	0.692710	0.043
C26B	0.37633(17)	0.2529(8)	0.8260(8)	0.0345(12)
H26B	0.366882	0.239882	0.913085	0.041
C24B	0.37393(18)	0.2016(9)	0.5712(8)	0.0336(13)
H24B	0.361907	0.152543	0.484725	0.040
C27B	0.40695(17)	0.3360(8)	0.8298(7)	0.0243(10)
H27B	0.419472	0.382559	0.916508	0.029
C23B	0.4049(4)	0.281(2)	0.5610(16)	0.034(3)

U_{eq} is defined as 1/3 of the trace of the orthogonalized U_{ij} tensor.

Supplementary Table 4. Anisotropic displacement parameters [Å^2] for **3a**.
The anisotropic displacement factor exponent takes the form:
$-2\pi^2[h^2(a^*)^2U_{11} + k^2(b^*)^2U_{22} + \ldots + 2hka^*b^*U_{12}]$

**Atom**	U_{11}	U_{22}	U_{33}	U_{23}	U_{13}	U_{12}

S1	0.01680(12)	0.01568(12)	0.01667(12)	0.00334(11)	0.00327(9)	0.00167(11)
O1	0.0274(5)	0.0186(4)	0.0264(5)	0.0023(4)	0.0115(4)	0.0046(4)
O2	0.0176(4)	0.0275(5)	0.0221(4)	0.0073(4)	−0.0008(3)	−0.0015(4)
O3	0.0257(5)	0.0360(6)	0.0182(4)	0.0049(4)	0.0065(4)	0.0015(4)
O4A	0.0354(15)	0.070(3)	0.050(2)	0.029(2)	0.0119(14)	0.0310(17)
O4B	0.0371(14)	0.077(3)	0.0452(18)	0.024(2)	0.0086(13)	0.0330(18)
N1	0.0165(4)	0.0158(5)	0.0152(4)	−0.0002(3)	0.0030(3)	−0.0009(3)
C1	0.0177(5)	0.0168(5)	0.0147(5)	0.0036(4)	0.0032(4)	0.0005(4)
C2	0.0259(6)	0.0209(6)	0.0147(5)	−0.0007(4)	0.0039(5)	−0.0012(5)
C3	0.0251(6)	0.0305(7)	0.0144(5)	0.0038(5)	0.0023(5)	−0.0002(5)
C4	0.0179(5)	0.0268(7)	0.0210(6)	0.0100(5)	0.0056(4)	0.0022(5)
C5	0.0203(5)	0.0172(5)	0.0245(6)	0.0040(5)	0.0059(5)	−0.0002(5)
C6	0.0187(5)	0.0185(6)	0.0167(5)	0.0013(4)	0.0031(4)	−0.0017(4)
C7	0.0279(7)	0.0319(8)	0.0323(7)	0.0177(6)	0.0060(6)	0.0061(6)
C8	0.0187(5)	0.0163(5)	0.0148(5)	−0.0019(4)	0.0017(4)	−0.0001(4)
C9	0.0181(5)	0.0159(5)	0.0127(4)	−0.0001(5)	0.0013(4)	0.0015(5)
C10	0.0189(5)	0.0135(5)	0.0137(5)	−0.0003(4)	0.0028(4)	−0.0002(4)
C11	0.0177(5)	0.0224(6)	0.0202(6)	−0.0076(5)	0.0030(4)	−0.0017(5)
C12	0.0190(5)	0.0146(5)	0.0144(5)	−0.0001(4)	0.0020(4)	−0.0009(4)
C13	0.0209(5)	0.0185(6)	0.0155(5)	−0.0024(4)	−0.0004(4)	0.0006(5)
C14	0.0310(7)	0.0213(7)	0.0240(7)	−0.0017(5)	−0.0033(5)	−0.0039(5)
C15	0.0391(8)	0.0259(7)	0.0313(8)	−0.0094(6)	−0.0108(6)	−0.0031(6)
C16	0.0369(8)	0.0338(8)	0.0196(6)	−0.0097(6)	−0.0074(6)	0.0120(7)
C17	0.0348(7)	0.0331(8)	0.0154(6)	−0.0021(5)	0.0027(5)	0.0110(6)
C18	0.0258(6)	0.0244(6)	0.0165(5)	−0.0025(5)	0.0035(5)	0.0021(5)
C19	0.0181(5)	0.0171(5)	0.0180(5)	−0.0004(4)	0.0031(4)	−0.0037(4)
C20	0.0155(5)	0.0320(7)	0.0186(5)	−0.0039(6)	0.0017(4)	−0.0031(5)
C21	0.0185(6)	0.0459(9)	0.0183(6)	0.0106(6)	0.0070(5)	0.0104(6)
C22	0.0283(7)	0.0279(7)	0.0434(9)	0.0193(7)	0.0201(6)	0.0135(6)
C23A	0.020(2)	0.018(4)	0.047(3)	0.010(2)	0.0038(17)	0.005(2)
C24A	0.0200(16)	0.0183(19)	0.031(2)	−0.0005(19)	−0.0004(17)	−0.0038(13)
C25A	0.0193(17)	0.0181(17)	0.042(2)	0.0054(19)	0.0027(17)	−0.0017(13)
C26A	0.022(2)	0.021(2)	0.034(3)	0.008(2)	0.0104(18)	0.0007(15)
C27A	0.024(2)	0.020(2)	0.022(2)	0.0011(15)	0.0038(15)	0.0037(14)
C28	0.0276(7)	0.0263(8)	0.0490(10)	−0.0073(7)	0.0125(7)	−0.0041(6)
C29	0.0378(9)	0.0363(10)	0.0565(12)	−0.0086(9)	0.0069(8)	0.0013(8)
C30	0.0306(8)	0.0428(11)	0.0676(13)	−0.0160(10)	0.0069(8)	0.0064(8)

C31A	0.0194(14)	0.0323(19)	0.0194(17)	0.0051(16)	0.0028(11)	0.0078(14)
C31B	0.0141(13)	0.041(2)	0.0174(15)	0.0007(16)	0.0001(10)	0.0035(14)
C32A	0.0166(14)	0.038(2)	0.0300(18)	0.006(2)	−0.0022(12)	0.0048(17)
C32B	0.0159(15)	0.052(3)	0.0322(18)	0.007(2)	−0.0031(12)	−0.0005(18)
C33A	0.0156(14)	0.036(3)	0.051(3)	0.010(2)	−0.0058(14)	−0.0044(16)
C33B	0.0179(16)	0.050(3)	0.047(3)	0.007(2)	−0.0067(15)	−0.010(2)
C34A	0.0121(15)	0.033(3)	0.037(4)	0.0029(18)	−0.0070(15)	−0.0039(14)
C34B	0.023(2)	0.039(3)	0.040(4)	0.004(2)	−0.0033(19)	−0.0091(19)
C35A	0.0305(16)	0.043(2)	0.037(2)	0.0178(19)	0.0122(15)	0.0180(16)
C35B	0.0285(15)	0.044(2)	0.0254(15)	0.0072(15)	0.0039(12)	0.0151(15)
C36	0.0232(6)	0.0231(6)	0.0161(5)	0.0050(5)	0.0033(4)	0.0032(5)
C25B	0.029(3)	0.0193(19)	0.064(4)	0.002(3)	0.024(3)	−0.0031(19)
C26B	0.037(3)	0.023(2)	0.050(3)	0.008(2)	0.027(2)	0.003(2)
C24B	0.031(2)	0.0185(17)	0.053(3)	−0.009(3)	0.011(3)	−0.0037(16)
C27B	0.031(3)	0.020(2)	0.027(3)	0.0094(18)	0.0167(19)	0.0060(17)
C23B	0.026(4)	0.021(3)	0.061(5)	0.006(4)	0.023(4)	0.000(3)

Supplementary Table 5. Bond lengths and angles for **3a**.

**Atom–Atom**	**Length [Å]**
S1–O1	1.4371(11)
S1–O2	1.4374(10)
S1–N1	1.6286(11)
S1–C1	1.7632(13)
O3–C19	1.2218(16)
O4A–C35A	1.219(5)
O4B–C35B	1.214(4)
N1–C8	1.4840(16)
N1–C11	1.4768(16)
C1–C2	1.3951(17)
C1–C6	1.3914(18)
C2–H2	0.9500
C2–C3	1.392(2)
C3–H3	0.9500
C3–C4	1.394(2)
C4–C5	1.3936(19)
C4–C7	1.5052(19)
C5–H5	0.9500

C5–C6	1.3905(18)
C6–H6	0.9500
C7–H7A	0.9800
C7–H7B	0.9800
C7–H7C	0.9800
C8–H8A	0.9900
C8–H8B	0.9900
C8–C9	1.5351(17)
C9–H9	1.0000
C9–C10	1.5133(16)
C9–C36	1.5357(18)
C10–C11	1.5114(17)
C10–C12	1.3470(17)
C11–H11A	0.9900
C11–H11B	0.9900
C12–C13	1.4933(17)
C12–C19	1.4967(17)
C13–C14	1.391(2)
C13–C18	1.4011(19)
C14–H14	0.9500
C14–C15	1.399(2)
C15–H15	0.9500
C15–C16	1.381(3)
C16–H16	0.9500
C16–C17	1.383(3)
C17–H17	0.9500
C17–C18	1.3912(19)
C18–H18	0.9500
C19–C20	1.5064(18)
C20–C21	1.396(2)
C20–C34A	1.426(7)
C20–C34B	1.378(7)
C21–C22	1.500(2)
C21–C31A	1.472(5)
C21–C31B	1.399(4)
C22–C23A	1.488(16)
C22–C27A	1.355(7)

C22–C27B	1.479(6)
C22–C23B	1.305(16)
C23A–C24A	1.422(12)
C23A–C28	1.495(12)
C24A–H24A	0.9500
C24A–C25A	1.377(7)
C25A–H25A	0.9500
C25A–C26A	1.386(6)
C26A–H26A	0.9500
C26A–C27A	1.394(7)
C27A–H27A	0.9500
C28–H28A	1.0000
C28–H28	1.0000
C28–C29	1.532(3)
C28–C30	1.537(3)
C28–C23B	1.553(11)
C29–H29A	0.9800
C29–H29B	0.9800
C29–H29C	0.9800
C30–H30A	0.9800
C30–H30B	0.9800
C30–H30C	0.9800
C31A–C32A	1.390(7)
C31A–C35A	1.473(5)
C31B–C32B	1.389(6)
C31B–C35B	1.490(6)
C32A–H32A	0.9500
C32A–C33A	1.361(7)
C32B–H32B	0.9500
C32B–C33B	1.347(8)
C33A–H33A	0.9500
C33A–C34A	1.397(6)
C33B–H33B	0.9500
C33B–C34B	1.404(7)
C34A–H34A	0.9500
C34B–H34B	0.9500
C35A–H35A	0.9500

C35B–H35B	0.9500
C36–H36A	0.9800
C36–H36B	0.9800
C36–H36C	0.9800
C25B–H25B	0.9500
C25B–C26B	1.381(8)
C25B–C24B	1.380(8)
C26B–H26B	0.9500
C26B–C27B	1.380(7)
C24B–H24B	0.9500
C24B–C23B	1.401(11)
C27B–H27B	0.9500
**Atom–Atom–Atom**	**Angle [°]**
O1–S1–O2	120.26(7)
O1–S1–N1	106.09(6)
O1–S1–C1	107.93(6)
O2–S1–N1	106.60(6)
O2–S1–C1	108.29(6)
N1–S1–C1	106.98(6)
C8–N1–S1	120.37(9)
C11–N1–S1	116.59(8)
C11–N1–C8	109.40(10)
C2–C1–S1	120.45(10)
C6–C1–S1	118.86(10)
C6–C1–C2	120.66(12)
C1–C2–H2	120.5
C3–C2–C1	118.90(13)
C3–C2–H2	120.5
C2–C3–H3	119.3
C2–C3–C4	121.41(13)
C4–C3–H3	119.3
C3–C4–C7	121.08(13)
C5–C4–C3	118.53(12)
C5–C4–C7	120.38(14)
C4–C5–H5	119.5
C6–C5–C4	121.07(13)

C6–C5–H5	119.5
C1–C6–H6	120.3
C5–C6–C1	119.40(12)
C5–C6–H6	120.3
C4–C7–H7A	109.5
C4–C7–H7B	109.5
C4–C7–H7C	109.5
H7A–C7–H7B	109.5
H7A–C7–H7C	109.5
H7B–C7–H7C	109.5
N1–C8–H8A	111.4
N1–C8–H8B	111.4
N1–C8–C9	101.84(10)
H8A–C8–H8B	109.3
C9–C8–H8A	111.4
C9–C8–H8B	111.4
C8–C9–H9	110.7
C8–C9–C36	111.82(10)
C10–C9–C8	101.26(9)
C10–C9–H9	110.7
C10–C9–C36	111.26(11)
C36–C9–H9	110.7
C11–C10–C9	107.19(10)
C12–C10–C9	128.82(11)
C12–C10–C11	123.95(11)
N1–C11–C10	104.03(10)
N1–C11–H11A	111.0
N1–C11–H11B	111.0
C10–C11–H11A	111.0
C10–C11–H11B	111.0
H11A–C11–H11B	109.0
C10–C12–C13	121.96(11)
C10–C12–C19	120.18(11)
C13–C12–C19	117.84(11)
C14–C13–C12	120.52(12)
C14–C13–C18	118.47(13)
C18–C13–C12	121.02(12)

C13–C14–H14	119.8
C13–C14–C15	120.50(15)
C15–C14–H14	119.8
C14–C15–H15	119.9
C16–C15–C14	120.18(15)
C16–C15–H15	119.9
C15–C16–H16	120.0
C15–C16–C17	120.08(14)
C17–C16–H16	120.0
C16–C17–H17	120.0
C16–C17–C18	119.94(15)
C18–C17–H17	120.0
C13–C18–H18	119.6
C17–C18–C13	120.84(14)
C17–C18–H18	119.6
O3–C19–C12	121.98(12)
O3–C19–C20	118.54(12)
C12–C19–C20	119.39(11)
C21–C20–C19	120.69(12)
C21–C20–C34A	127.4(3)
C34A–C20–C19	111.7(3)
C34B–C20–C19	126.3(3)
C34B–C20–C21	113.0(3)
C20–C21–C22	118.45(12)
C20–C21–C31A	108.8(2)
C20–C21–C31B	126.5(3)
C31A–C21–C22	132.5(2)
C31B–C21–C22	114.7(3)
C23A–C22–C21	122.3(5)
C27A–C22–C21	127.3(3)
C27A–C22–C23A	110.2(5)
C27B–C22–C21	109.3(3)
C23B–C22–C21	120.9(5)
C23B–C22–C27B	129.7(5)
C22–C23A–C28	117.0(8)
C24A–C23A–C22	124.4(9)
C24A–C23A–C28	118.4(11)

C23A–C24A–H24A	121.1
C25A–C24A–C23A	117.8(7)
C25A–C24A–H24A	121.1
C24A–C25A–H25A	119.8
C24A–C25A–C26A	120.4(5)
C26A–C25A–H25A	119.8
C25A–C26A–H26A	120.4
C25A–C26A–C27A	119.1(5)
C27A–C26A–H26A	120.4
C22–C27A–C26A	127.8(5)
C22–C27A–H27A	116.1
C26A–C27A–H27A	116.1
C23A–C28–H28	108.8
C23A–C28–C29	111.6(6)
C23A–C28–C30	108.9(8)
C29–C28–H28A	107.0
C29–C28–H28	108.8
C29–C28–C30	109.99(16)
C29–C28–C23B	118.0(6)
C30–C28–H28A	107.0
C30–C28–H28	108.8
C30–C28–C23B	107.2(8)
C23B–C28–H28A	107.0
C28–C29–H29A	109.5
C28–C29–H29B	109.5
C28–C29–H29C	109.5
H29A–C29–H29B	109.5
H29A–C29–H29C	109.5
H29B–C29–H29C	109.5
C28–C30–H30A	109.5
C28–C30–H30B	109.5
C28–C30–H30C	109.5
H30A–C30–H30B	109.5
H30A–C30–H30C	109.5
H30B–C30–H30C	109.5
C21–C31A–C35A	115.1(4)
C32A–C31A–C21	125.4(4)

C32A–C31A–C35A	119.3(4)
C21–C31B–C35B	125.7(4)
C32B–C31B–C21	115.6(4)
C32B–C31B–C35B	118.7(4)
C31A–C32A–H32A	119.9
C33A–C32A–C31A	120.2(4)
C33A–C32A–H32A	119.9
C31B–C32B–H32B	119.6
C33B–C32B–C31B	120.8(4)
C33B–C32B–H32B	119.6
C32A–C33A–H33A	120.1
C32A–C33A–C34A	119.9(5)
C34A–C33A–H33A	120.1
C32B–C33B–H33B	119.7
C32B–C33B–C34B	120.7(5)
C34B–C33B–H33B	119.7
C20–C34A–H34A	121.2
C33A–C34A–C20	117.7(5)
C33A–C34A–H34A	121.2
C20–C34B–C33B	122.8(6)
C20–C34B–H34B	118.6
C33B–C34B–H34B	118.6
O4A–C35A–C31A	121.7(5)
O4A–C35A–H35A	119.2
C31A–C35A–H35A	119.2
O4B–C35B–C31B	123.4(4)
O4B–C35B–H35B	118.3
C31B–C35B–H35B	118.3
C9–C36–H36A	109.5
C9–C36–H36B	109.5
C9–C36–H36C	109.5
H36A–C36–H36B	109.5
H36A–C36–H36C	109.5
H36B–C36–H36C	109.5
C26B–C25B–H25B	120.1
C24B–C25B–H25B	120.1
C24B–C25B–C26B	119.7(5)

C25B–C26B–H26B	119.9
C27B–C26B–C25B	120.2(5)
C27B–C26B–H26B	119.9
C25B–C24B–H24B	117.2
C25B–C24B–C23B	125.5(8)
C23B–C24B–H24B	117.2
C22–C27B–H27B	123.2
C26B–C27B–C22	113.6(5)
C26B–C27B–H27B	123.2
C22–C23B–C28	125.4(8)
C22–C23B–C24B	111.0(8)
C24B–C23B–C28	123.4(11)

Supplementary Table 6. Torsion angles for **3a**.

**Atom–Atom–Atom–Atom**	**Torsion Angle [°]**
S1–N1–C8–C9	−172.07(8)
S1–N1–C11–C10	152.98(9)
S1–C1–C2–C3	−177.16(11)
S1–C1–C6–C5	177.57(10)
O1–S1–N1–C8	−178.40(9)
O1–S1–N1–C11	45.22(11)
O1–S1–C1–C2	−21.91(12)
O1–S1–C1–C6	160.14(10)
O2–S1–N1–C8	−49.11(10)
O2–S1–N1–C11	174.51(9)
O2–S1–C1–C2	−153.57(11)
O2–S1–C1–C6	28.48(12)
O3–C19–C20–C21	−111.33(15)
O3–C19–C20–C34A	63.5(5)
O3–C19–C20–C34B	68.2(7)
N1–S1–C1–C2	91.87(11)
N1–S1–C1–C6	−86.08(11)
N1–C8–C9–C10	39.57(12)
N1–C8–C9–C36	−78.98(12)
C1–S1–N1–C8	66.58(10)
C1–S1–N1–C11	−69.81(10)

C1–C2–C3–C4	−0.1(2)
C2–C1–C6–C5	−0.37(19)
C2–C3–C4–C5	−0.8(2)
C2–C3–C4–C7	178.32(14)
C3–C4–C5–C6	1.2(2)
C4–C5–C6–C1	−0.63(19)
C6–C1–C2–C3	0.8(2)
C7–C4–C5–C6	−177.94(13)
C8–N1–C11–C10	12.11(13)
C8–C9–C10–C11	−33.67(13)
C8–C9–C10–C12	144.06(13)
C9–C10–C11–N1	14.20(13)
C9–C10–C12–C13	−171.88(12)
C9–C10–C12–C19	9.9(2)
C10–C12–C13–C14	−123.27(15)
C10–C12–C13–C18	56.59(18)
C10–C12–C19–O3	29.5(2)
C10–C12–C19–C20	−153.82(13)
C11–N1–C8–C9	−32.92(12)
C11–C10–C12–C13	5.5(2)
C11–C10–C12–C19	−172.68(12)
C12–C10–C11–N1	−163.67(12)
C12–C13–C14–C15	179.49(14)
C12–C13–C18–C17	−179.60(13)
C12–C19–C20–C21	71.87(17)
C12–C19–C20–C34A	−113.3(5)
C12–C19–C20–C34B	−108.6(7)
C13–C12–C19–O3	−148.77(13)
C13–C12–C19–C20	27.92(17)
C13–C14–C15–C16	0.0(3)
C14–C13–C18–C17	0.3(2)
C14–C15–C16–C17	0.6(3)
C15–C16–C17–C18	−0.7(2)
C16–C17–C18–C13	0.3(2)
C18–C13–C14–C15	−0.4(2)
C19–C12–C13–C14	54.95(17)
C19–C12–C13–C18	−125.19(14)

C19–C20–C21–C22	−1.6(2)
C19–C20–C21–C31A	−176.4(2)
C19–C20–C21–C31B	171.0(3)
C19–C20–C34A–C33A	178.4(7)
C19–C20–C34B–C33B	−175.9(6)
C20–C21–C22–C23A	71.7(9)
C20–C21–C22–C27A	−102.1(4)
C20–C21–C22–C27B	−104.0(3)
C20–C21–C22–C23B	73.6(11)
C20–C21–C31A–C32A	−7.9(5)
C20–C21–C31A–C35A	178.3(3)
C20–C21–C31B–C32B	9.7(5)
C20–C21–C31B–C35B	−171.3(3)
C21–C20–C34A–C33A	−7.3(12)
C21–C20–C34B–C33B	3.7(12)
C21–C22–C23A–C24A	−170.6(11)
C21–C22–C23A–C28	15.3(17)
C21–C22–C27A–C26A	168.7(5)
C21–C22–C27B–C26B	173.4(4)
C21–C22–C23B–C28	12(2)
C21–C22–C23B–C24B	−172.6(8)
C21–C31A–C32A–C33A	3.9(7)
C21–C31A–C35A–O4A	175.8(4)
C21–C31B–C32B–C33B	−5.6(7)
C21–C31B–C35B–O4B	175.0(4)
C22–C21–C31A–C32A	178.2(3)
C22–C21–C31A–C35A	4.5(5)
C22–C21–C31B–C32B	−177.5(3)
C22–C21–C31B–C35B	1.5(5)
C22–C23A–C24A–C25A	0(2)
C22–C23A–C28–C29	−153.5(10)
C22–C23A–C28–C30	84.9(13)
C23A–C22–C27A–C26A	−5.8(11)
C23A–C24A–C25A–C26A	−3.5(13)
C24A–C23A–C28–C29	32.0(16)
C24A–C23A–C28–C30	−89.6(14)
C24A–C25A–C26A–C27A	2.4(9)

C25A–C26A–C27A–C22	2.9(9)
C27A–C22–C23A–C24A	4.2(18)
C27A–C22–C23A–C28	−169.9(10)
C28–C23A–C24A–C25A	174.2(10)
C29–C28–C23B–C22	−150.4(14)
C29–C28–C23B–C24B	35(2)
C30–C28–C23B–C22	84.9(18)
C30–C28–C23B–C24B	−89.8(16)
C31A–C21–C22–C23A	−114.9(9)
C31A–C21–C22–C27A	71.2(5)
C31A–C32A–C33A–C34A	−0.2(9)
C31B–C21–C22–C27B	82.6(4)
C31B–C21–C22–C23B	−99.8(11)
C31B–C32B–C33B–C34B	1.6(10)
C32A–C31A–C35A–O4A	1.6(7)
C32A–C33A–C34A–C20	1.6(12)
C32B–C31B–C35B–O4B	−6.0(7)
C32B–C33B–C34B–C20	−0.6(13)
C34A–C20–C21–C22	−175.5(6)
C34A–C20–C21–C31A	9.7(6)
C34B–C20–C21–C22	178.9(6)
C34B–C20–C21–C31B	−8.6(6)
C35A–C31A–C32A–C33A	177.4(5)
C35B–C31B–C32B–C33B	175.3(5)
C36–C9–C10–C11	85.28(12)
C36–C9–C10–C12	−96.99(15)
C25B–C26B–C27B–C22	−0.2(8)
C25B–C24B–C23B–C22	−1(2)
C25B–C24B–C23B–C28	174.0(10)
C26B–C25B–C24B–C23B	−2.2(15)
C24B–C25B–C26B–C27B	2.8(9)
C27B–C22–C23B–C28	−170.7(9)
C27B–C22–C23B–C24B	5(2)
C23B–C22–C27B–C26B	−4.0(14)

X-Ray Analysis of 9a

A solution of compound **9a** (20 mg) was dissolved in acetone (0.3 mL) in a glass tube, then layed with *n*-pentane (2.0 mL). The solution was kept standing. Single crystals of **9a** were obtained by slow diffusion at room temperature for X-Ray crystallographic analysis. The diffraction data were collected using Mo Kα radiation and a Bruker Photon III C7 Detector.

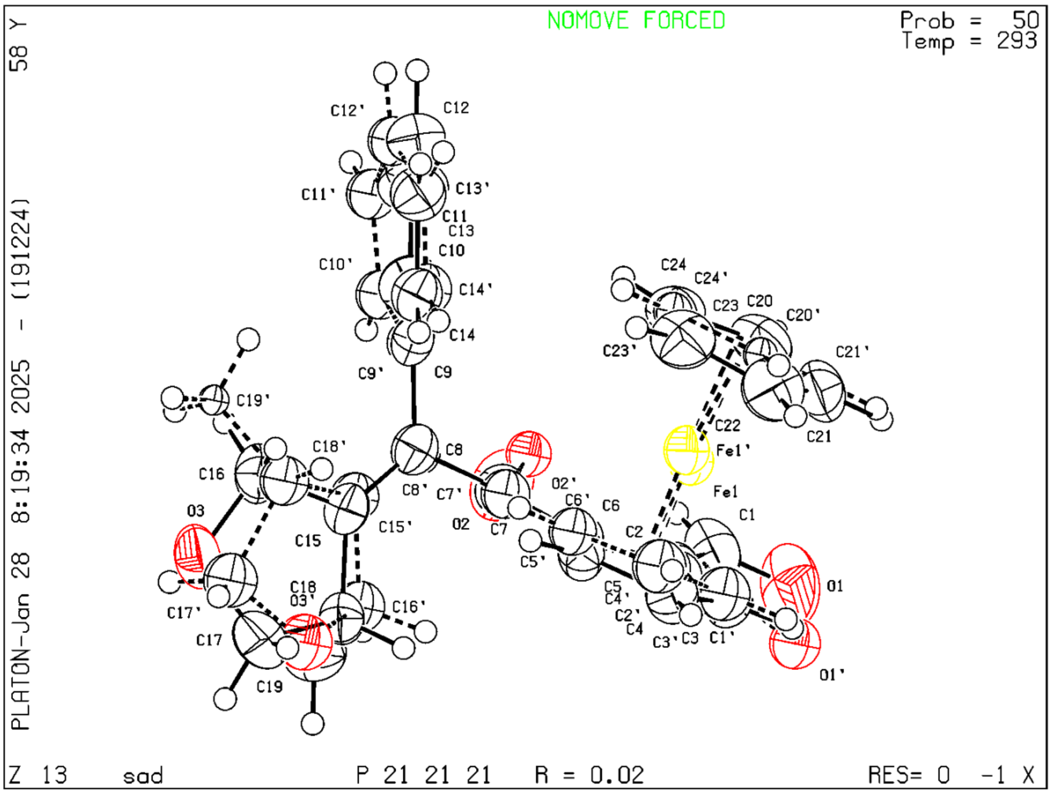

Supplementary Figure 3. Structure of compound **9a** (CCDC 2427462).

CheckCIF analysis: No A- or B-level alerts were generated for this structure.

Supplementary Table 7. Crystal data and structure refinement for compound **9a**.

Compound	**9a**
CCDC	**2427462**
Empirical Formula	$C_{24}H_{22}FeO_3$
Formula weight	414.26
T [K]	293(2)
λ [Å]	0.71073
Crystal system	Orthorhombic
Space group	$P2_12_12_1$
a [Å]	8.216(2)
b [Å]	14.453(2)
c [Å]	16.822(3)
V [Å^3]	1997.5(7)

Z	4
μ [mm^{-1}]	0.776
F(000)	864
Crystal size [mm]	0.406 x 0.311 x 0.134
Θ max [°]	1.858 to 25.053
Reflections collected	40880
Independent reflections	3533
R_{int}	0.0319
Data/restraints/parameters	3533 / 503 / 287
GooF	1.090
R1 [I >2σ(I)]	0.0237
wR2 [all data]	0.0613
Absolute structure parameter[6]	0.012(4)
ρ_{max}/ρ_{min} [e A^{-3}]	0.216/-0.118
Shape and color	red blocks

$^{a}R1 = \Sigma||F_o| - |F_c||/\Sigma|F_o|$. $^{b}wR_2 = [\Sigma w(F_o^2 - F_c^2)^2/\Sigma(F_o^2)^2]^{1/2}$

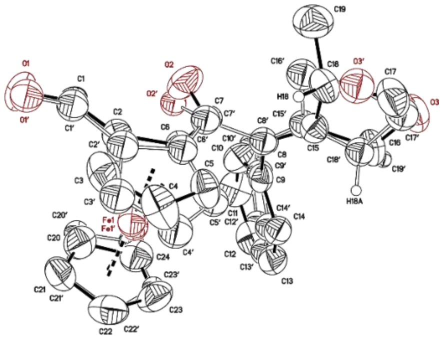

Supplementary Figure 4. Asymmetric unit of **9a.**

The structure crystallizes in space group $P2_12_12_1$ with one molecule in the asymmetric unit. All hydrogen atoms were placed according to geometrical criteria and refined with a riding model. The structure contained a small amount of a diastereomer of **9a** which was treated as disorder. Because of the low occupation of the minor position, it was refined as a rigid group with isotropic displacement parameters for all non-iron atoms. The anisotropic displacement parameters of both iron atoms were constrained to be identical. For all other atoms, restraints for the anisotropic displacement parameters were used. The occupancy of the minor position refined to 0.054(4). The absolute structure was confirmed by the Flack parameter of 0.012(4).[11] Bond lengths and angles are listed in Supplementary Table 8.

Supplementary Table 8. Bond lengths [Å] and angles [°] for **9a.**

Fe(1)-C(2)	2.024(3)
Fe(1)-C(22)	2.025(3)
Fe(1)-C(21)	2.027(3)
Fe(1)-C(6)	2.030(3)
Fe(1)-C(5)	2.036(3)
Fe(1)-C(3)	2.038(3)
Fe(1)-C(23)	2.041(3)
Fe(1)-C(20)	2.049(3)
Fe(1)-C(4)	2.052(3)
Fe(1)-C(24)	2.065(3)
C(20)-C(21)	1.408(6)
C(20)-C(24)	1.409(5)
C(21)-C(22)	1.410(5)
C(22)-C(23)	1.419(5)
C(23)-C(24)	1.390(6)
O(1)-C(1)	1.216(4)
C(1)-C(2)	1.461(5)
O(2)-C(7)	1.221(4)
C(2)-C(3)	1.430(5)
C(2)-C(6)	1.460(4)
C(3)-C(4)	1.408(5)
C(4)-C(5)	1.412(4)
C(5)-C(6)	1.419(4)
C(6)-C(7)	1.459(4)
C(7)-C(8)	1.511(4)
C(8)-C(15)	1.352(4)
C(8)-C(9)	1.497(4)
C(9)-C(14)	1.369(4)
C(9)-C(10)	1.385(4)
C(10)-C(11)	1.386(5)
C(11)-C(12)	1.377(6)
C(12)-C(13)	1.369(5)
C(13)-C(14)	1.383(4)
C(15)-C(18)	1.505(4)
C(15)-C(16)	1.511(5)
C(16)-O(3)	1.407(4)
O(3)-C(17)	1.432(4)
C(17)-C(18)	1.545(5)

C(18)-C(19)	1.520(5)
Fe(1')-C(2')	2.0154
Fe(1')-C(21')	2.0211
Fe(1')-C(5')	2.0342
Fe(1')-C(3')	2.0399
Fe(1')-C(20')	2.0400
Fe(1')-C(22')	2.0434
Fe(1')-C(6')	2.0449
Fe(1')-C(23')	2.0488
Fe(1')-C(4')	2.0616
Fe(1')-C(24')	2.0743
C(20')-C(21')	1.4027
C(20')-C(24')	1.4063
C(21')-C(22')	1.4294
C(22')-C(23')	1.4118
C(23')-C(24')	1.3865
O(1')-C(1')	1.2183
C(1')-C(2')	1.4544
O(2')-C(7')	1.2451
C(2')-C(3')	1.4307
C(2')-C(6')	1.4561
C(3')-C(4')	1.4172
C(4')-C(5')	1.4040
C(5')-C(6')	1.4483
C(6')-C(7')	1.4792
C(7')-C(8')	1.5302
C(8')-C(15')	1.0794
C(8')-C(9')	1.4899
C(9')-C(14')	1.3746
C(9')-C(10')	1.3936
C(10')-C(11')	1.3817
C(11')-C(12')	1.3662
C(12')-C(13')	1.3643
C(13')-C(14')	1.3841
C(15')-C(18')	1.4675
C(15')-C(16')	1.5512
C(16')-O(3')	1.3617
O(3')-C(17')	1.4485

C(17')-C(18')	1.5579
C(18')-C(19')	1.4751

C(2)-Fe(1)-C(22)	151.29(17)
C(2)-Fe(1)-C(21)	118.61(14)
C(22)-Fe(1)-C(21)	40.72(15)
C(2)-Fe(1)-C(6)	42.23(11)
C(22)-Fe(1)-C(6)	161.83(14)
C(21)-Fe(1)-C(6)	157.43(14)
C(2)-Fe(1)-C(5)	69.39(15)
C(22)-Fe(1)-C(5)	122.54(17)
C(21)-Fe(1)-C(5)	157.98(16)
C(6)-Fe(1)-C(5)	40.86(13)
C(2)-Fe(1)-C(3)	41.22(15)
C(22)-Fe(1)-C(3)	114.75(19)
C(21)-Fe(1)-C(3)	103.39(17)
C(6)-Fe(1)-C(3)	69.67(13)
C(5)-Fe(1)-C(3)	68.32(14)
C(2)-Fe(1)-C(23)	166.99(19)
C(22)-Fe(1)-C(23)	40.85(16)
C(21)-Fe(1)-C(23)	68.12(15)
C(6)-Fe(1)-C(23)	128.09(15)
C(5)-Fe(1)-C(23)	108.78(17)
C(3)-Fe(1)-C(23)	151.1(2)
C(2)-Fe(1)-C(20)	110.38(17)
C(22)-Fe(1)-C(20)	67.67(18)
C(21)-Fe(1)-C(20)	40.40(16)
C(6)-Fe(1)-C(20)	125.54(16)
C(5)-Fe(1)-C(20)	160.25(15)
C(3)-Fe(1)-C(20)	125.36(16)
C(23)-Fe(1)-C(20)	66.73(17)
C(2)-Fe(1)-C(4)	68.84(16)
C(22)-Fe(1)-C(4)	102.58(17)
C(21)-Fe(1)-C(4)	120.40(17)
C(6)-Fe(1)-C(4)	68.83(13)
C(5)-Fe(1)-C(4)	40.40(12)
C(3)-Fe(1)-C(4)	40.26(15)
C(23)-Fe(1)-C(4)	118.68(19)

C(20)-Fe(1)-C(4)	159.17(14)
C(2)-Fe(1)-C(24)	130.09(19)
C(22)-Fe(1)-C(24)	68.10(18)
C(21)-Fe(1)-C(24)	68.26(17)
C(6)-Fe(1)-C(24)	112.75(15)
C(5)-Fe(1)-C(24)	124.19(15)
C(3)-Fe(1)-C(24)	164.46(17)
C(23)-Fe(1)-C(24)	39.57(16)
C(20)-Fe(1)-C(24)	40.04(14)
C(4)-Fe(1)-C(24)	155.26(18)
C(21)-C(20)-C(24)	109.2(4)
C(21)-C(20)-Fe(1)	69.0(2)
C(24)-C(20)-Fe(1)	70.6(2)
C(20)-C(21)-C(22)	107.3(4)
C(20)-C(21)-Fe(1)	70.7(2)
C(22)-C(21)-Fe(1)	69.57(19)
C(21)-C(22)-C(23)	107.3(3)
C(21)-C(22)-Fe(1)	69.71(19)
C(23)-C(22)-Fe(1)	70.2(2)
C(24)-C(23)-C(22)	109.2(3)
C(24)-C(23)-Fe(1)	71.2(2)
C(22)-C(23)-Fe(1)	68.98(19)
C(23)-C(24)-C(20)	107.0(4)
C(23)-C(24)-Fe(1)	69.3(2)
C(20)-C(24)-Fe(1)	69.4(2)
O(1)-C(1)-C(2)	121.7(4)
C(3)-C(2)-C(6)	107.0(3)
C(3)-C(2)-C(1)	124.7(3)
C(6)-C(2)-C(1)	127.8(3)
C(3)-C(2)-Fe(1)	69.9(2)
C(6)-C(2)-Fe(1)	69.09(17)
C(1)-C(2)-Fe(1)	119.9(2)
C(4)-C(3)-C(2)	108.6(3)
C(4)-C(3)-Fe(1)	70.41(19)
C(2)-C(3)-Fe(1)	68.85(17)
C(3)-C(4)-C(5)	108.5(3)
C(3)-C(4)-Fe(1)	69.34(18)
C(5)-C(4)-Fe(1)	69.17(17)

C(4)-C(5)-C(6)	109.2(3)
C(4)-C(5)-Fe(1)	70.43(17)
C(6)-C(5)-Fe(1)	69.33(16)
C(5)-C(6)-C(7)	127.0(3)
C(5)-C(6)-C(2)	106.7(3)
C(7)-C(6)-C(2)	126.2(3)
C(5)-C(6)-Fe(1)	69.80(17)
C(7)-C(6)-Fe(1)	125.0(2)
C(2)-C(6)-Fe(1)	68.68(17)
O(2)-C(7)-C(6)	122.1(3)
O(2)-C(7)-C(8)	120.3(3)
C(6)-C(7)-C(8)	117.6(3)
C(15)-C(8)-C(9)	124.0(3)
C(15)-C(8)-C(7)	121.7(3)
C(9)-C(8)-C(7)	114.3(2)
C(14)-C(9)-C(10)	119.0(3)
C(14)-C(9)-C(8)	121.0(3)
C(10)-C(9)-C(8)	119.9(3)
C(9)-C(10)-C(11)	119.9(3)
C(12)-C(11)-C(10)	120.3(3)
C(13)-C(12)-C(11)	119.9(3)
C(12)-C(13)-C(14)	119.7(4)
C(9)-C(14)-C(13)	121.2(3)
C(8)-C(15)-C(18)	127.0(3)
C(8)-C(15)-C(16)	124.4(3)
C(18)-C(15)-C(16)	108.5(3)
O(3)-C(16)-C(15)	105.0(3)
C(16)-O(3)-C(17)	107.0(2)
O(3)-C(17)-C(18)	105.0(3)
C(15)-C(18)-C(19)	112.8(3)
C(15)-C(18)-C(17)	100.0(3)
C(19)-C(18)-C(17)	111.9(3)
C(2')-Fe(1')-C(21')	119.0
C(2')-Fe(1')-C(5')	69.7
C(21')-Fe(1')-C(5')	158.6
C(2')-Fe(1')-C(3')	41.3
C(21')-Fe(1')-C(3')	104.6
C(5')-Fe(1')-C(3')	68.2

C(2')-Fe(1')-C(20')	110.7
C(21')-Fe(1')-C(20')	40.4
C(5')-Fe(1')-C(20')	159.1
C(3')-Fe(1')-C(20')	126.7
C(2')-Fe(1')-C(22')	152.2
C(21')-Fe(1')-C(22')	41.2
C(5')-Fe(1')-C(22')	122.2
C(3')-Fe(1')-C(22')	115.7
C(20')-Fe(1')-C(22')	67.8
C(2')-Fe(1')-C(6')	42.0
C(21')-Fe(1')-C(6')	156.9
C(5')-Fe(1')-C(6')	41.6
C(3')-Fe(1')-C(6')	69.6
C(20')-Fe(1')-C(6')	124.5
C(22')-Fe(1')-C(6')	161.8
C(2')-Fe(1')-C(23')	166.6
C(21')-Fe(1')-C(23')	68.0
C(5')-Fe(1')-C(23')	108.1
C(3')-Fe(1')-C(23')	151.2
C(20')-Fe(1')-C(23')	66.4
C(22')-Fe(1')-C(23')	40.4
C(6')-Fe(1')-C(23')	127.7
C(2')-Fe(1')-C(4')	69.2
C(21')-Fe(1')-C(4')	121.6
C(5')-Fe(1')-C(4')	40.1
C(3')-Fe(1')-C(4')	40.4
C(20')-Fe(1')-C(4')	160.7
C(22')-Fe(1')-C(4')	102.9
C(6')-Fe(1')-C(4')	69.2
C(23')-Fe(1')-C(4')	118.2
C(2')-Fe(1')-C(24')	130.1
C(21')-Fe(1')-C(24')	68.2
C(5')-Fe(1')-C(24')	123.0
C(3')-Fe(1')-C(24')	165.6
C(20')-Fe(1')-C(24')	40.0
C(22')-Fe(1')-C(24')	67.8
C(6')-Fe(1')-C(24')	111.7
C(23')-Fe(1')-C(24')	39.3

C(4')-Fe(1')-C(24')	154.0
C(21')-C(20')-C(24')	109.7
C(21')-C(20')-Fe(1')	69.1
C(24')-C(20')-Fe(1')	71.3
C(20')-C(21')-C(22')	107.0
C(20')-C(21')-Fe(1')	70.5
C(22')-C(21')-Fe(1')	70.2
C(23')-C(22')-C(21')	106.4
C(23')-C(22')-Fe(1')	70.0
C(21')-C(22')-Fe(1')	68.6
C(24')-C(23')-C(22')	110.3
C(24')-C(23')-Fe(1')	71.3
C(22')-C(23')-Fe(1')	69.6
C(23')-C(24')-C(20')	106.6
C(23')-C(24')-Fe(1')	69.4
C(20')-C(24')-Fe(1')	68.7
O(1')-C(1')-C(2')	121.1
C(3')-C(2')-C(1')	123.4
C(3')-C(2')-C(6')	107.8
C(1')-C(2')-C(6')	128.7
C(3')-C(2')-Fe(1')	70.3
C(1')-C(2')-Fe(1')	122.7
C(6')-C(2')-Fe(1')	70.1
C(4')-C(3')-C(2')	108.8
C(4')-C(3')-Fe(1')	70.6
C(2')-C(3')-Fe(1')	68.4
C(5')-C(4')-C(3')	108.1
C(5')-C(4')-Fe(1')	68.9
C(3')-C(4')-Fe(1')	69.0
C(4')-C(5')-C(6')	109.7
C(4')-C(5')-Fe(1')	71.0
C(6')-C(5')-Fe(1')	69.6
C(5')-C(6')-C(2')	105.6
C(5')-C(6')-C(7')	122.3
C(2')-C(6')-C(7')	131.0
C(5')-C(6')-Fe(1')	68.8
C(2')-C(6')-Fe(1')	67.9
C(7')-C(6')-Fe(1')	118.9

O(2')-C(7')-C(6')	121.5
O(2')-C(7')-C(8')	116.6
C(6')-C(7')-C(8')	114.2
C(15')-C(8')-C(9')	121.5
C(15')-C(8')-C(7')	121.8
C(9')-C(8')-C(7')	114.9
C(14')-C(9')-C(10')	118.6
C(14')-C(9')-C(8')	121.6
C(10')-C(9')-C(8')	119.5
C(11')-C(10')-C(9')	119.7
C(12')-C(11')-C(10')	121.0
C(13')-C(12')-C(11')	119.3
C(12')-C(13')-C(14')	120.4
C(9')-C(14')-C(13')	120.3
C(8')-C(15')-C(18')	128.1
C(8')-C(15')-C(16')	117.4
C(18')-C(15')-C(16')	110.2
O(3')-C(16')-C(15')	98.3
C(16')-O(3')-C(17')	119.1
O(3')-C(17')-C(18')	97.8
C(15')-C(18')-C(19')	117.8
C(15')-C(18')-C(17')	105.1
C(19')-C(18')-C(17')	110.6

Supplementary Section 5. Key Mechanistic Findings

Deuterium labelling experiments

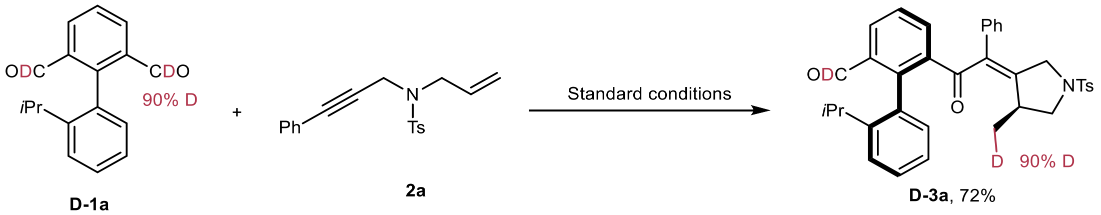

The electrocatalysis was carried out in an undivided cell, with a zinc electrode (10 mm × 25 mm × 0.25 mm) and a nickel foam electrode (10 mm × 25 mm × 1 mm). In the glovebox, [(*S*,*S*)-BDPP)]$CoBr_2$ (6.6 mg, 0.010 mmol), NaBARF (44.3 mg, 0.050 mmol), and dry DCM (2.0 mL) were placed in a 10 mL cell. Electrocatalysis was performed at 40 °C with a constant current of 1.0 mA maintained for 50 min. Then, the nickel foam cathode and the zinc anode were taken out, bialdehyde **1a-D** (0.11 mmol, 27.9 mg) and 1,6-enyne **2a** (0.10 mmol, 32.5 mg) were added, and the reaction mixture was stirred at 40 °C under a nitrogen atmosphere for 24 h. The resulting mixture was purified by column chromatography on silica gel (DCM) yielded **D-3a** (41.7 mg, 72%, >20:1 d.r., 90% D) as a white solid.

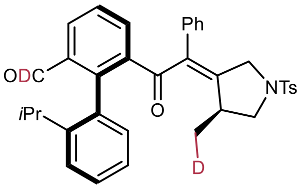

D-3a

^{1}H-NMR (300 MHz, $CDCl_3$) δ 9.53 (s, 0.1H), 7.98 (dd, *J* = 7.8, 1.5 Hz, 1H), 7.63 – 7.54 (m, 3H), 7.40 – 7.25 (m, 8H), 7.05 – 6.99 (m, 1H), 6.97 – 6.88 (m, 2H), 6.76 (dd, *J* = 7.6, 1.5 Hz, 1H), 4.03 (dd, *J* = 16.3, 1.5 Hz, 1H), 3.44 (d, *J* = 16.3 Hz, 1H), 3.14 (dd, *J* = 8.7, 1.7 Hz, 1H), 3.08 – 2.95 (m, 2H), 2.54 (p, *J* = 6.8 Hz, 1H), 2.43 (s, 3H), 1.20 (d, *J* = 6.8 Hz, 3H), 1.08 (d, *J* = 5.3 Hz, 2H), 0.95 (d, *J* = 6.8 Hz, 3H).

^{13}C-NMR (101 MHz, $CDCl_3$) δ 195.63 (C_q), 191.6 (CH), 150.9 (C_q), 148.6 (C_q), 144.0 (C_q), 143.9 (C_q), 139.2 (C_q), 136.0 (C_q), 135.3 (C_q), 134.9 (C_q), 133.8 (CH), 133.5 (C_q), 132.3 (C_q), 129.7 (CH), 129.2 (CH), 129.0 (CH), 128.9 (CH), 128.6 (CH), 128.6 (CH), 128.2 (CH), 127.8 (CH), 127.4 (CH), 125.5 (CH), 124.8 (CH), 54.9 (CH_2), 51.0 (CH_2), 36.0 (CH), 30.8 (CH), 24.2 (CH_3), 22.9 (CH_3), 21.6 (CH_3), 19.7 (CH_2D), 19.4 (t, *J* = 19.6 Hz) (CH_2D).

HR-MS (ESI): *m*/*z* calcd. for $[C_{36}H_{33}D_2NO_4S + Na]^+$ 602.2305, found 602.2288.

Deuterium crossover experiments

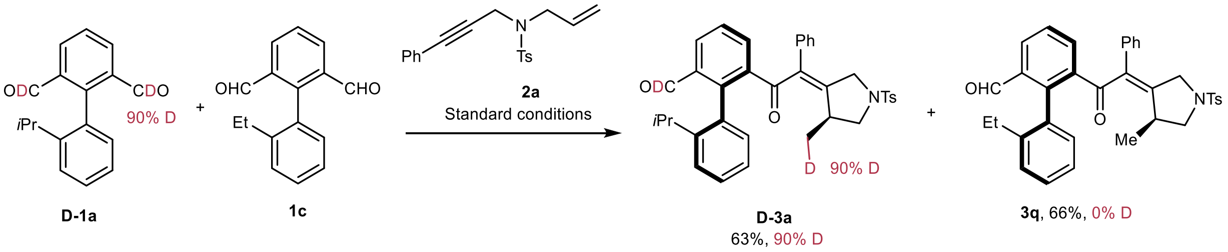

The electrocatalysis was carried out in an undivided cell, with a zinc electrode (10 mm × 25 mm × 0.25 mm) and a nickel foam electrode (10 mm × 25 mm × 1 mm). In the glovebox, [(*S*,*S*)-BDPP)]CoBr$_2$ (6.6 mg, 0.010 mmol), NaBARF (44.3 mg, 0.050 mmol), and dry DCM (2.0 mL) were placed in a 10 mL cell. Electrocatalysis was performed at 40 °C with a constant current of 1.0 mA maintained for 50 min. Then, the nickel foam cathode and the zinc anode were taken out, bialdehyde **D-1a** (0.055 mmol, 14.0 mg), **1c** (0.055 mmol, 13.1 mg) and 1,6-enyne **2a** (0.10 mmol, 32.5 mg) were added, and the reaction mixture was stirred at 40 °C under a nitrogen atmosphere for 24 h. The resulting mixture was purified by column chromatography on silica gel (DCM) yielded **D-3a** (18.3 mg, 63%, >20:1 d.r., 90% D) and **3q** (18.6 mg, 66%, >20:1 d.r.).

KIE experiments

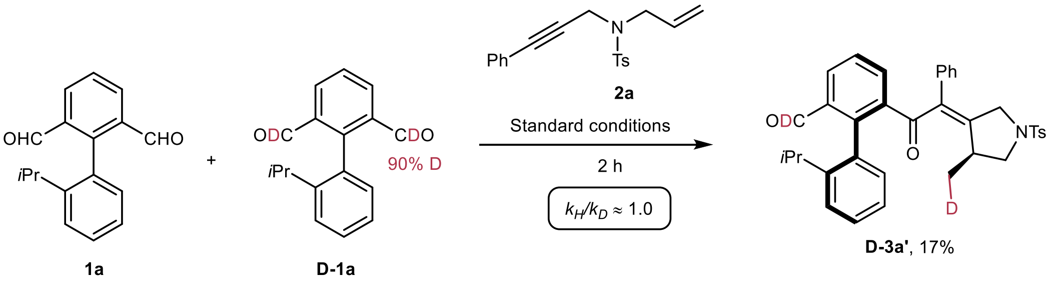

The electrocatalysis was carried out in an undivided cell, with a zinc electrode (10 mm × 25 mm × 0.25 mm) and a nickel foam electrode (10 mm × 25 mm × 1 mm). In the glovebox, [(*S*,*S*)-BDPP)]CoBr$_2$ (6.6 mg, 0.010 mmol), NaBARF (44.3 mg, 0.050 mmol), and dry DCM (2.0 mL) were placed in a 10 mL cell. Electrocatalysis was performed at 40 °C with a constant current of 1.0 mA maintained for 50 min. Then, the nickel foam cathode and the zinc anode were taken out, bialdehyde **1a** (0.055 mmol, 13.9 mg), **D-1a** (0.055 mmol, 14.0 mg) and 1,6-enyne **2a** (0.10 mmol, 32.5 mg) were added, and the reaction mixture was stirred at 40 °C under a nitrogen atmosphere for 2 h. The resulting mixture was purified by column chromatography on silica gel (DCM) yielded **D-3a'** (9.9 mg, 17%, >20:1 d.r.)

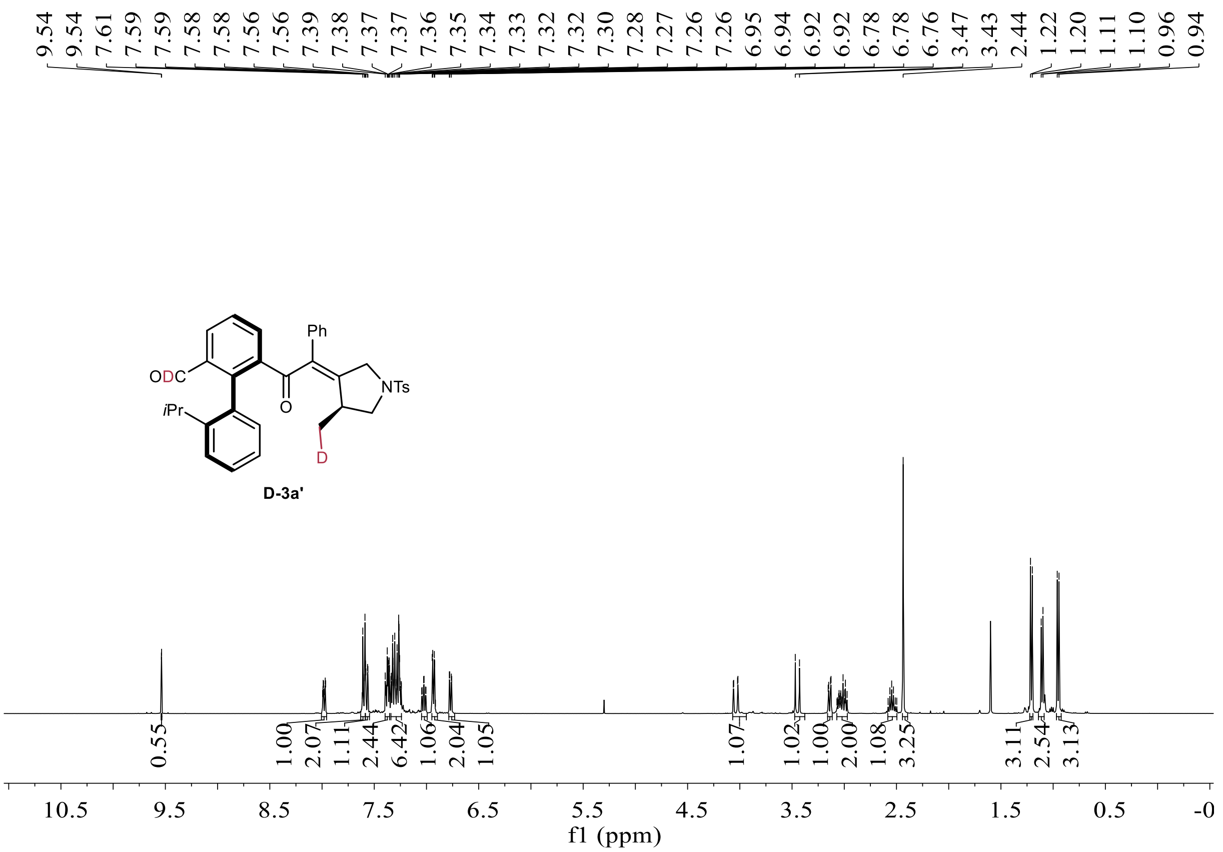

Control experiments for the reaction of enyne with ethanol

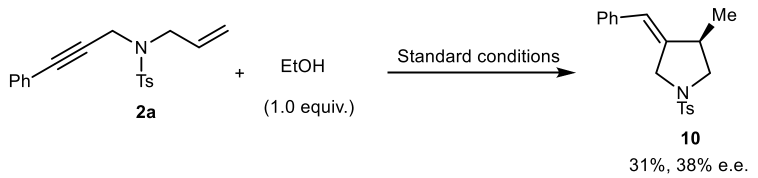

The electrocatalysis was carried out in an undivided cell, with a zinc electrode (10 mm × 25 mm × 0.25 mm) and a nickel foam electrode (10 mm × 25 mm × 1 mm). In the glovebox, [(*S*,*S*)-BDPP)]$CoBr_2$ (6.6 mg, 0.010 mmol), NaBARF (44.3 mg, 0.050 mmol), and dry DCM (2.0 mL) were placed in a 10 mL cell. Electrocatalysis was performed at 40 °C with a constant current of 1.0 mA maintained for 50 min. Then, the nickel foam cathode and the zinc anode were taken out, 1,6-enyne **2a** (0.10 mmol, 32.5 mg) and ethanol (0.10 mmol, 4.6 mg) were added, and the reaction mixture was stirred at 40 °C under a nitrogen atmosphere for 24 h. The resulting mixture was purified by column chromatography on silica gel (*n*-hexane/ethyl acetate: 10/1) yielded **10** (10.1 mg, 31%, 38% e.e.) as a colorless oil.

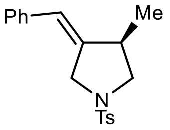

10

^{1}H-NMR (400 MHz, CDCl$_3$) δ 7.72 (d, J = 8.2 Hz, 2H), 7.37 – 7.29 (m, 5H), 7.14 (d, J = 7.6 Hz, 2H), 6.29 – 6.16 (m, 1H), 4.24 (dd, J = 14.9, 2.6 Hz, 1H), 4.10 – 4.02 (m, 1H), 3.55 (dd, J = 9.1, 7.2 Hz, 1H), 2.91 – 2.84 (m, 1H), 2.73 (t, J = 8.5 Hz, 1H), 2.41 (s, 3H), 1.17 (d, J = 6.6 Hz, 3H).

^{13}C-NMR (101 MHz, CDCl$_3$) δ 143.8 (C$_q$), 142.0 (C$_q$), 136.7 (C$_q$), 133.1 (C$_q$), 129.9 (CH), 128.7 (CH), 128.2 (CH), 127.9 (CH), 127.1 (CH), 122.2 (CH), 54.0 (CH$_2$), 51.0 (CH$_2$), 39.2 (CH), 21.7 (CH$_3$), 17.1 (CH$_3$).

The analytic data of **10** are consistent with the literature.[12]

HPLC separation (Chiralpak® IF-3, *n*-hexane/*i*-PrOH 97:3, 1.0 mL/min, detection at 250 nm): t_r (minor) = 28.8 min, t_r (major) = 32.2 min, 38% e.e.

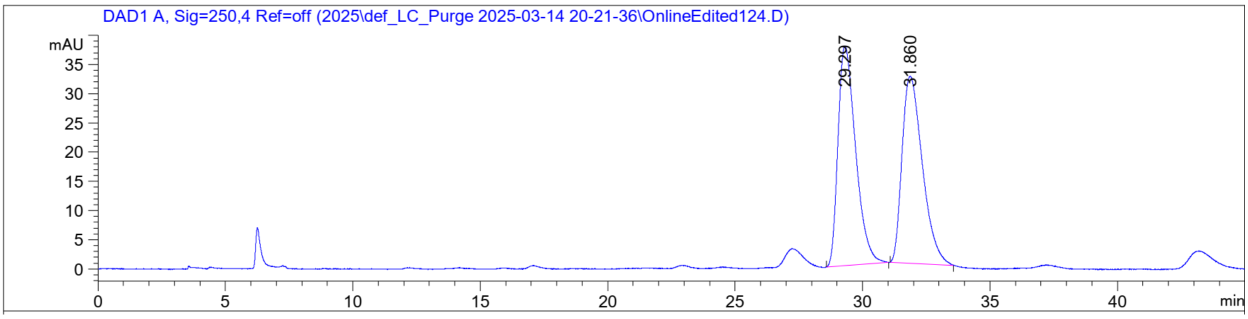

Peak #	RetTime [min]	Type	Width [min]	Area [mAU*s]	Height [mAU]	Area %
1	29.297	BB	0.5684	1804.65576	37.57036	50.4202
2	31.860	BB	0.6499	1774.57813	32.00407	49.5798

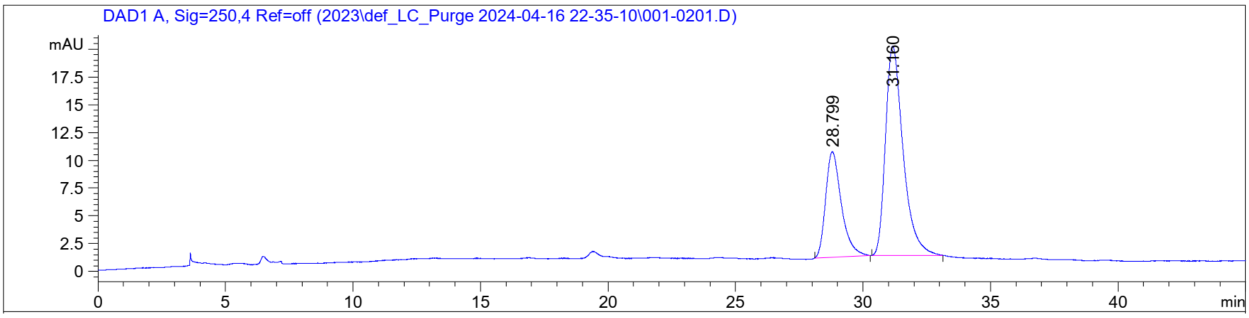

Peak #	RetTime [min]	Type	Width [min]	Area [mAU*s]	Height [mAU]	Area %
1	28.799	BB	0.5134	407.70816	9.50548	31.0242
2	31.160	BB	0.5675	906.45227	18.83547	68.9758

Control experiments with alkyne or alkene

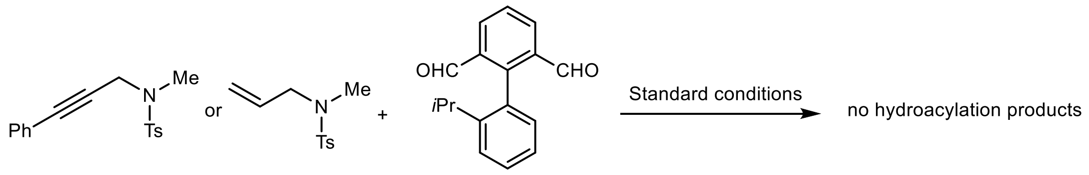

11 **12** **1a**

The electrocatalysis was carried out in an undivided cell, with a zinc electrode (10 mm × 25 mm × 0.25 mm) and a nickel foam electrode (10 mm × 25 mm × 1 mm). In the glovebox, [(*S*,*S*)-BDPP]$CoBr_2$ (6.6 mg, 0.010 mmol), NaBARF (44.3 mg, 0.050 mmol), and dry DCM (2.0 mL) were placed in a 10 mL cell. Electrocatalysis was performed at 40 °C with a constant current of 1.0 mA maintained for 50 min. Then, the nickel foam cathode and the zinc anode were taken out, **11** (0.10 mmol, 29.9 mg) or **12** (0.10 mmol, 22.5 mg), and **1a** (0.11 mmol, 27.7 mg) were added, and the reaction mixture was stirred at 40 °C under a nitrogen atmosphere for 24 h. No hydroacylation products were found.

Cyclic voltammetry experiments

CV measurements were conducted with a Metrohm Autolab PGSTAT204 potentiostat and Nova 2.1 software. A glassy carbon (disk, diameter: 3 mm), a coiled platinum wire counter electrode and a saturated calomel (SCE) reference electrode were employed. The voltammograms were recorded at room temperature in CH_3CN (3 mL) with 0.1 M *n*Bu_4NPF_6 as supporting electrolyte under N_2 atomsphere. The scan rate is 100 mV/s.

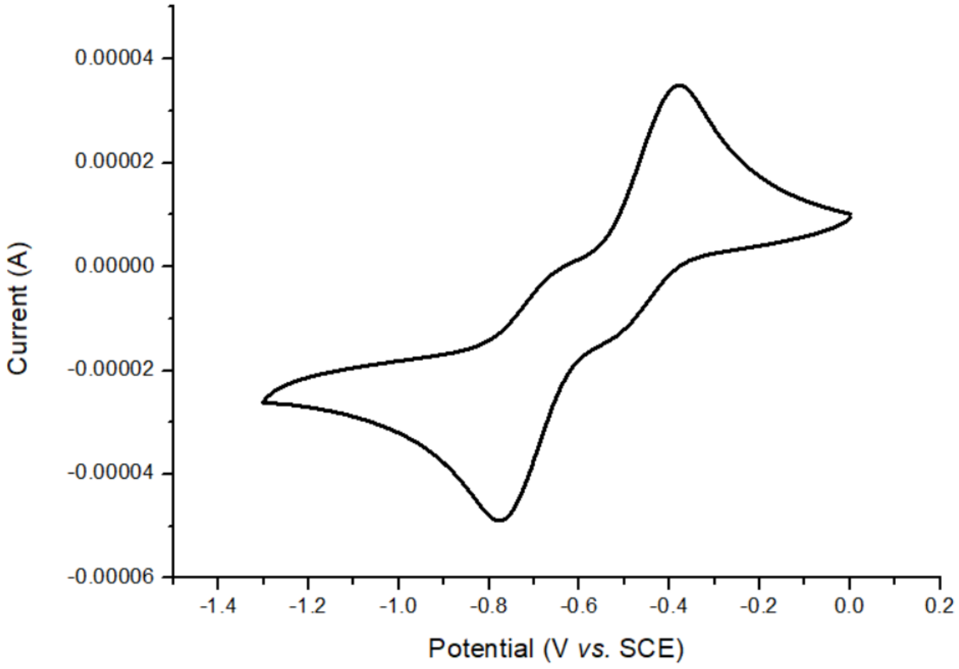

Supplementary Figure 5. CV studies of [(*S*,*S*)-BDPP]$CoBr_2$ (10 mM).

An alternative pathway contemplating oxidative addition

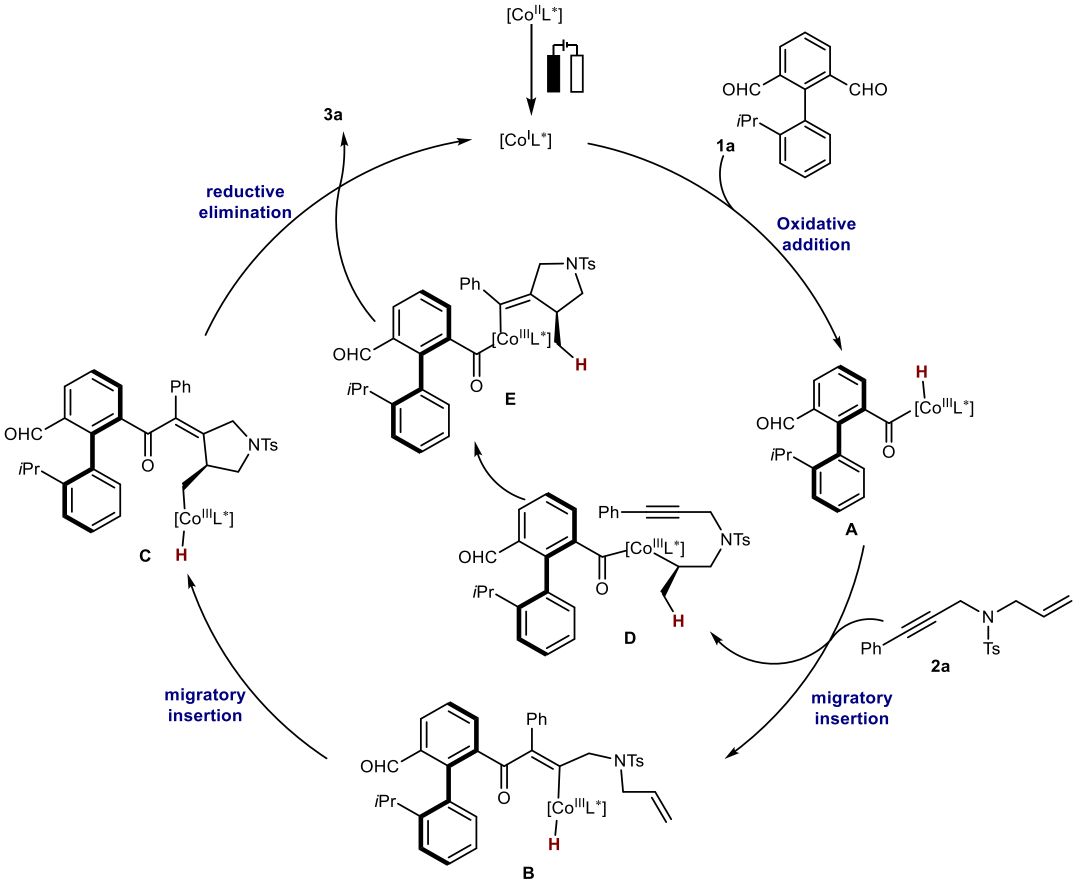

Supplementary Figure 6. An alternative pathway.

Supplementary Section 6. Synthetic applications

Gram-scale synthesis and follow-up transformations of product 3a

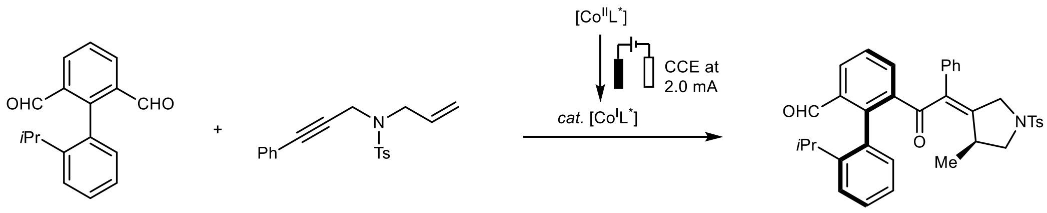

1a (2.2 mmol) **2a** (2.0 mmol) **3a**, 64%, 739 mg >20:1 d.r., >99% e.e.

The electrocatalysis was carried out in an undivided cell, with a zinc electrode (10 mm × 25 mm × 0.25 mm) and a nickel foam electrode (10 mm × 25 mm × 1 mm). In the glovebox, [(*S*,*S*)-BDPP)]$CoBr_2$ (132.0 mg, 0.20 mmol), NaBARF (886.2 mg, 1.0 mmol), and dry DCM (10.0 mL) were placed in a 20 mL cell. Electrocatalysis was performed at 40 °C with a constant current of 2.0 mA maintained for 8 h. Then, the nickel foam cathode and the zinc anode were taken out, bialdehyde **1a** (2.2 mmol, 556 mg) and 1,6-enyne **2a** (2.0 mmol, 650.0 mg) were added, and the reaction mixture was stirred at 40 °C under a nitrogen atmosphere for 24 h. The resulting mixture was purified by column chromatography on silica gel (DCM) yielded **3a** (739 mg, 64%, >20:1 d.r., >99% e.e.) as a white solid.

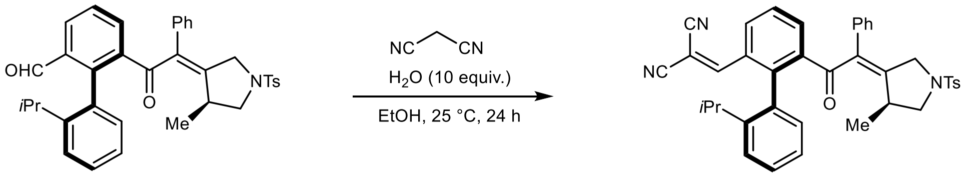

3a **13**, 96% >20:1 d.r., >99% e.e.

To a 10 mL vial was added **3a** (57.8 mg, 0.10 mmol), malononitrile (13.2 mg, 0.20 mmol), H_2O (18.0 mg, 1.0 mmol), and 1.0 mL EtOH. The mixture was stirred for 24 h at 25 °C. The resulting mixture was extracted with DCM for three times and the organic phase was collected. The organic phase was dried with anhydrous sodium sulfate and concentrated in vacuo to give a residue, which was purified by column chromatography on silica gel (DCM) yielded **13** (60.1 mg, 96%, >20:1 d.r., >99% e.e.) as a white solid.

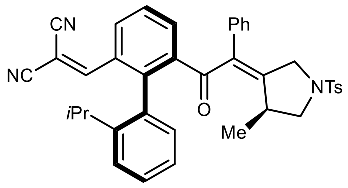

13

^{1}H-NMR (400 MHz, $CDCl_3$) δ 8.20 (dd, *J* = 8.1, 1.2 Hz, 1H), 7.59 (d, *J* = 8.2 Hz, 2H), 7.54 (dd, *J* = 7.8, 1.2 Hz, 1H), 7.43 – 7.34 (m, 4H), 7.33 – 7.27 (m, 5H), 7.05 – 7.01 (m, 1H), 6.94 – 6.88

(m, 2H), 6.61 (d, *J* = 7.1 Hz, 1H), 4.01 (dd, *J* = 16.5, 1.5 Hz, 1H), 3.43 (d, *J* = 16.4 Hz, 1H), 3.16 – 3.10 (m, 1H), 3.04 – 2.94 (m, 2H), 2.48 – 2.37 (m, 4H), 1.18 (d, *J* = 6.7 Hz, 3H), 1.10 (d, *J* = 6.7 Hz, 3H), 0.99 (d, *J* = 6.9 Hz, 3H).

^{13}C-NMR (101 MHz, $CDCl_3$) δ 195.1 (C_q), 158.3 (CH), 152.2 (C_q), 148.6 (C_q), 144.1 (C_q), 143.3 (C_q), 139.9 (C_q), 135.9 (C_q), 135.0 (C_q), 134.1 (C_q), 133.6 (CH), 132.4 (C_q), 131.4 (C_q), 129.9 (CH), 129.9 (CH), 129.1 (CH), 128.7 (CH), 128.5 (CH), 128.0 (CH), 127.9 (CH), 127.9 (CH), 126.3 (CH), 125.5 (CH), 113.3 (C_q), 112.3 (C_q), 84.7 (C_q), 55.0 (CH_2), 51.2 (CH_2), 36.2 (CH), 30.9 (CH), 24.3 (CH_3), 23.2 (CH_3), 21.7 (CH_3), 19.8 (CH_3).

IR (ATR): 1678, 1578, 1429, 1345, 1160, 1092, 1046, 736, 705 cm^{-1}.

HR-MS (ESI): *m*/*z* calcd. for $[C_{39}H_{35}N_3O_3S + Na]^+$ 648.2291, found 648.2295.

[α^{20}_{D}] = +130.8 (c = 1.0, DCM).

HPLC separation (Chiralpak® IE-3, *n*-hexane/*i*-PrOH 60:40, 1.0 mL/min, detection at 273 nm): t_r (major) = 13.9 min, >99% e.e.

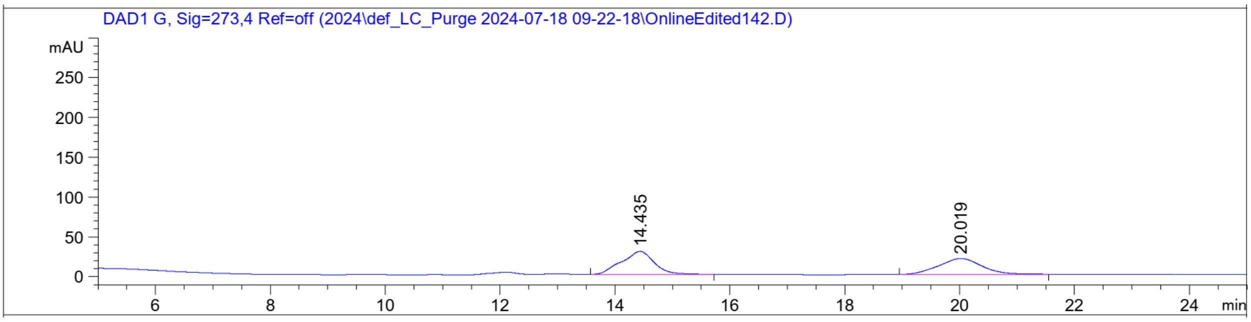

Peak #	RetTime [min]	Type	Width [min]	Area [mAU*s]	Height [mAU]	Area %
1	14.435	BB	0.5318	1144.68103	28.85770	52.6863
2	20.019	BB	0.6277	1027.95496	19.79982	47.3137

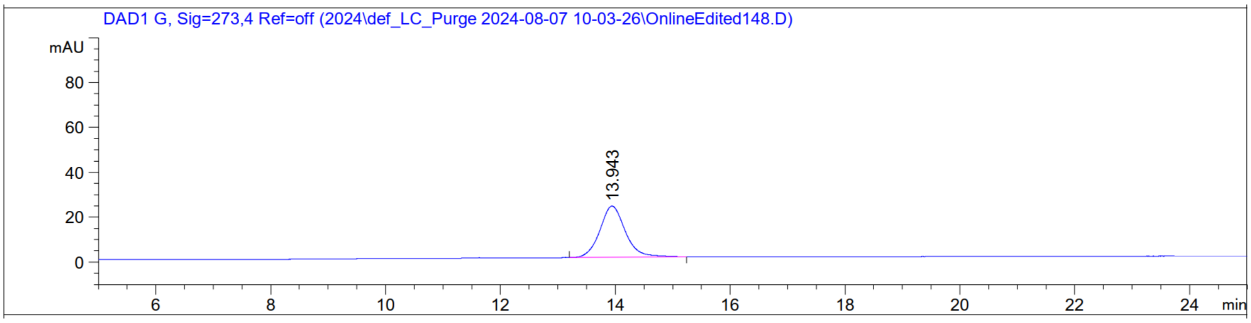

Peak #	RetTime [min]	Type	Width [min]	Area [mAU*s]	Height [mAU]	Area %
1	13.943	BB	0.4456	708.06836	22.84545	100.0000

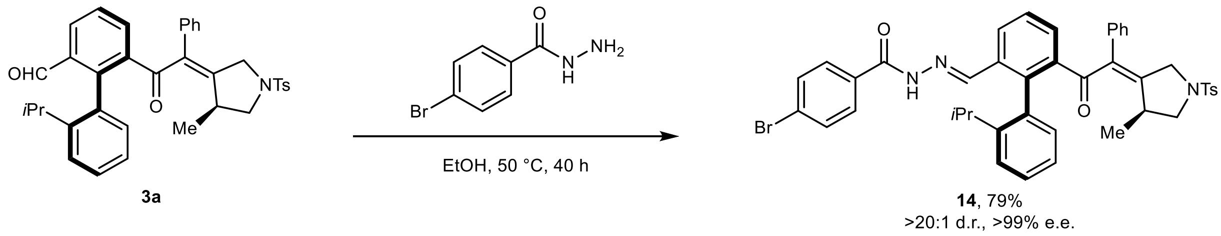

To a 10 mL vial was added **3a** (57.8 mg, 0.10 mmol), hydrazide (21.5 mg, 0.10 mmol), and 1.0 mL EtOH. The mixture was stirred for 40 h at 50 °C. The resulting mixture was extracted with DCM for three times and the organic phase was collected. The organic phase was dried with anhydrous sodium sulfate and concentrated in vacuo to give a residue, which was purified by column chromatography on silica gel (*n*-hexane/ethyl acetate: 1/1) yielded **14** (61.2 mg, 79%, >20:1 d.r., >99% e.e.) as a white solid.

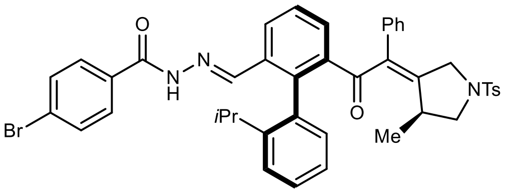

14

^{1}H-NMR (300 MHz, $CDCl_3$) δ 9.10 (s, 1H), 8.26 (s, 1H), 7.64 – 7.44 (m, 7H), 7.37 – 7.24 (m, 9H), 7.07 – 7.00 (m, 1H), 6.97 – 6.88 (m, 2H), 6.69 (d, J = 7.5 Hz, 1H), 4.04 (d, J = 16.1 Hz, 1H), 3.46 (d, J = 16.1 Hz, 1H), 3.17 – 2.96 (m, 3H), 2.57 – 2.48 (m, 1H), 2.43 (s, 3H), 1.15 (d, J = 6.7 Hz, 3H), 1.07 (d, J = 6.3 Hz, 3H), 0.93 (d, J = 6.8 Hz, 3H).

^{13}C-NMR (101 MHz, $CDCl_3$) δ 196.0 (C_q), 163.1 (C_q), 149.4 (C_q), 148.6 (C_q), 145.9 (CH), 144.0 (C_q), 140.8 (C_q), 138.3 (C_q), 136.2 (C_q), 135.7 (C_q), 135.5 (C_q), 133.3 (C_q), 132.5 (C_q), 131.9 (CH), 131.9 (C_q), 131.2 (CH), 129.8 (CH), 129.1 (CH), 128.9 (CH), 128.6 (CH), 128.5 (CH), 128.2 (CH), 127.9 (CH), 127.4 (CH), 127.0 (C_q), 125.6 (CH), 125.1 (CH), 54.9 (CH_2), 50.9 (CH_2), 36.0 (CH), 30.7 (CH), 24.4 (CH_3), 23.1 (CH_3), 21.7 (CH_3), 19.7 (CH_3).

IR (ATR): 1653, 1591, 1565, 1486, 1348, 1286, 1271, 1092, 1011, 759 cm^{-1}.

HR-MS (ESI): *m*/*z* calcd. for $[C_{43}H_{40}BrN_3O_4S + Na]^+$ 796.1815, found 796.1804.

$[\alpha_D^{20}]$ = +88.2 (c = 0.5, DCM).

HPLC separation (Chiralpak® IB-3, *n*-hexane/*i*-PrOH 80:20, 1.0 mL/min, detection at 220 nm): t_r (major) = 30.7 min, >99% e.e.

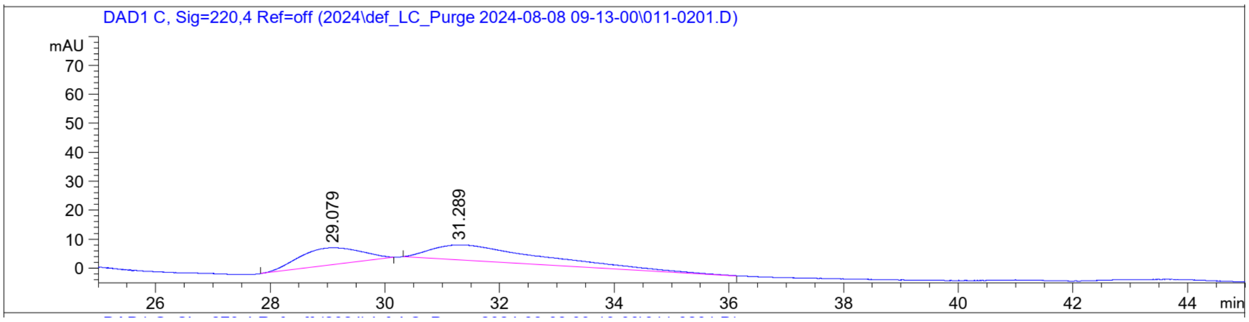

Peak #	RetTime [min]	Type	Width [min]	Area [mAU*s]	Height [mAU]	Area %
1	29.079	BB	0.8920	446.40228	5.87125	37.5674
2	31.289	BB	1.6765	741.86877	5.17190	62.4326

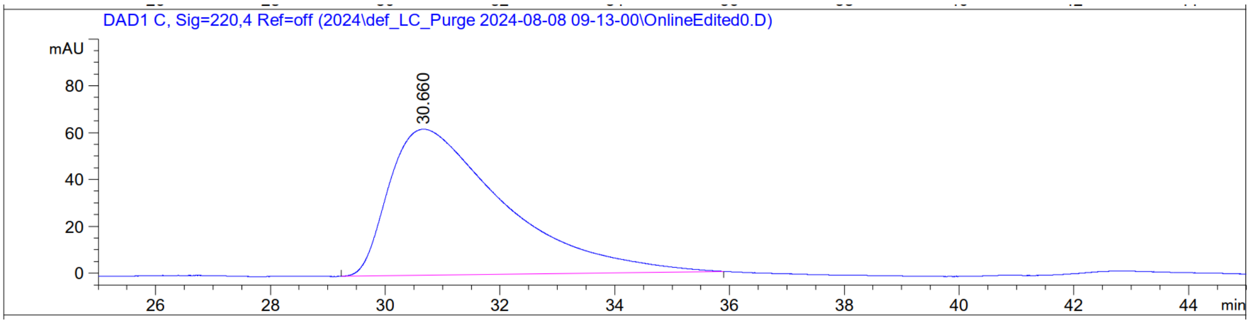

Peak #	RetTime [min]	Type	Width [min]	Area [mAU*s]	Height [mAU]	Area %
1	30.660	BB	1.6296	8690.98242	62.37225	100.0000

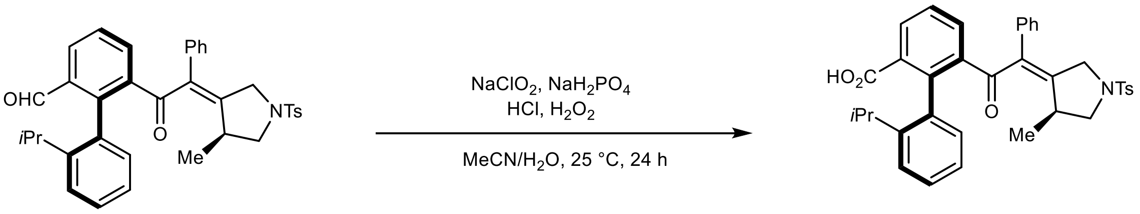

3a

15, 84%
>20:1 d.r., >99% e.e.

To a 10 mL vial was added **3a** (57.8 mg, 0.10 mmol), $NaClO_2$ (27.3 mg, 0.3 mmol), NaH_2PO_4 (36.0 mg, 0.3 mmol) was dissolved in MeCN/H_2O = 1:1 (1 mL). 100 µL solution of hydrochloric acid (2.0 M) was added, then 0.5 mL solution of hydrogen peroxide (30%) was added. The mixture was stirred at 50 °C for 24 h. The resulting mixture was extracted with DCM for three times and the organic phase was collected. The organic phase was dried with anhydrous sodium sulfate and concentrated in vacuo to give a residue, which was purified by column chromatography on silica gel (*n*-hexane/ethyl acetate: 3/1) yielded **15** (49.9 mg, 84%, >20:1 d.r., >99% e.e.) as a white solid.

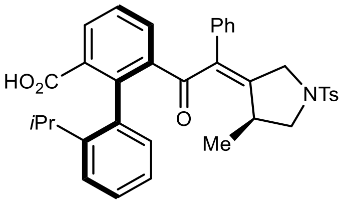

15

^{1}H-NMR (400 MHz, CDCl$_3$) δ 7.89 (dd, *J* = 7.8, 1.4 Hz, 1H), 7.56 (d, *J* = 8.3 Hz, 2H), 7.33 (dd, *J* = 7.7, 1.5 Hz, 1H), 7.30 (d, *J* = 8.0 Hz, 2H), 7.24 – 7.20 (m, 5H), 7.19 – 7.14 (m, 1H), 6.87 – 6.82 (m, 2H), 6.82 – 6.75 (m, 1H), 6.67 (dd, *J* = 7.6, 1.4 Hz, 1H), 3.89 (dd, *J* = 16.5, 1.5 Hz, 1H), 3.34 (d, *J* = 16.5 Hz, 1H), 3.08 (dd, *J* = 9.2, 1.7 Hz, 1H), 2.82 (dd, *J* = 9.2, 6.2 Hz, 1H), 2.78 – 2.72 (m, 1H), 2.46 – 2.37 (m, 4H), 1.11 (d, *J* = 6.8 Hz, 3H), 1.07 (d, *J* = 6.9 Hz, 3H), 0.92 (d, *J* = 6.8 Hz, 3H).

^{13}C-NMR (101 MHz, CDCl$_3$) δ 196.3 (C_q), 169.6 (C_q), 151.8 (C_q), 147.7 (C_q), 144.0 (C_q), 140.7 (C_q), 140.5 (C_q), 136.2 (C_q), 136.1 (C_q), 135.4 (C_q), 132.4 (C_q), 132.3 (CH), 132.1 (CH), 130.8 (C_q), 129.8 (CH), 128.9 (CH), 128.8 (CH), 128.4 (CH), 128.2 (CH), 128.1 (CH), 128.0 (CH), 126.9 (CH), 125.4 (CH), 124.4 (CH), 55.2 (CH_2), 51.1 (CH_2), 36.4 (CH), 30.8 (CH), 24.4 (CH_3), 23.2 (CH_3), 21.7 (CH_3), 20.1 (CH_3).

IR (ATR): 1701, 1680, 1454, 1347, 1289, 1161, 1092, 814, 761, 704 cm^{-1}.

HR-MS (ESI): *m*/*z* calcd. for $[C_{36}H_{35}NO_5S + Na]^+$ 616.2128, found 616.2109.

$[\alpha^{20}_{D}]$ = +88.5 (c = 0.2, DCM).

HPLC separation (Chiralpak® IC-3, *n*-hexane/*i*-PrOH 50:50, 1.0 mL/min, detection at 273 nm): t_r (major) = 14.8 min, >99% e.e.

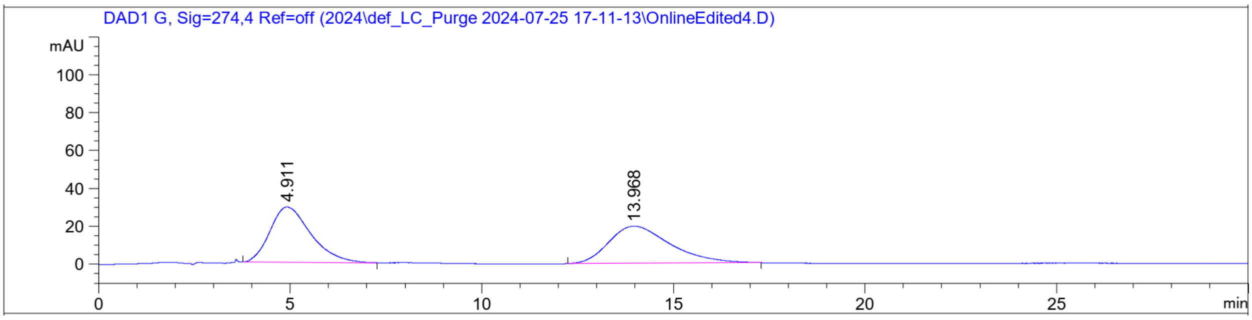

Peak #	RetTime [min]	Type	Width [min]	Area [mAU*s]	Height [mAU]	Area %
1	4.911	BB	0.8693	2160.80542	29.20274	49.7081
2	13.968	BB	1.3039	2186.17920	19.60567	50.2919

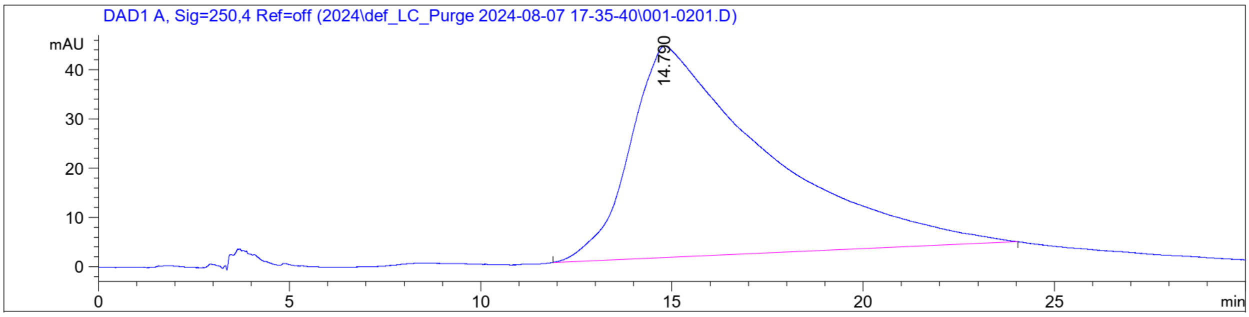

Peak #	RetTime [min]	Type	Width [min]	Area [mAU*s]	Height [mAU]	Area %
1	14.790	BB	2.9102	1.06868e4	42.94522	100.0000

Chiral carboxylic acid 15 was evaluated as a chiral ligand in the ruthenium-catalyzed asymmetric C−H activation reaction

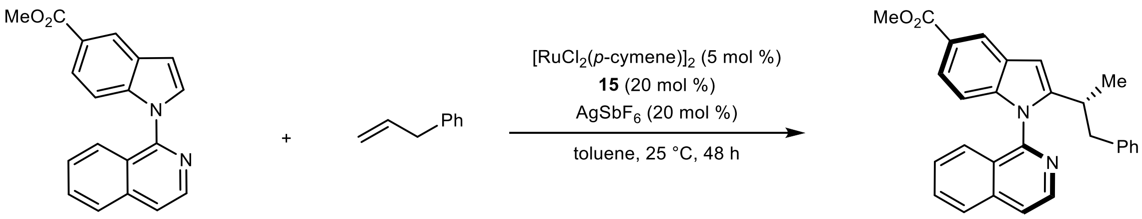

16 **17** **18**, 91%
>20:1 d.r., 50% e.e.

To a 10 mL vial was added **16** (0.10 mmol, 30.2 mg), **17** (0.30 mmol, 35.4 mg), $[RuCl_2(p\text{-}cymene)]_2$ (0.0050 mmol, 3.1 mg), **15** (0.020 mmol, 11.9 mg), $AgPF_6$ (0.020 mmol, 5.1 mg), and PhMe (1.0 mL). The mixture was stirred for 48 h at room temperature. The resulting mixture was purified by column chromatography on silica gel (*n*-hexane/ethyl acetate: 10/1) yielded **18** (38.3 mg, 91%, >20:1 d.r., 50% e.e.) as a white solid.

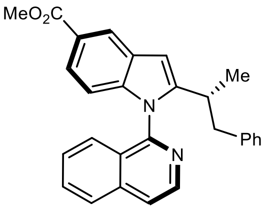

18

^{1}H-NMR (300 MHz, $CDCl_3$) δ 8.63 (d, *J* = 5.7 Hz, 1H), 8.42 (dd, *J* = 1.7, 0.7 Hz, 1H), 7.98 (d, *J* = 8.3 Hz, 1H), 7.87 (dd, *J* = 5.7, 0.9 Hz, 1H), 7.77 – 7.70 (m, 2H), 7.45 – 7.39 (m, 1H), 7.25 – 7.19 (m, 1H), 7.09 – 6.99 (m, 3H), 6.73 – 6.63 (m, 4H), 3.92 (s, 3H), 3.24 – 3.12 (m, 1H), 2.76 (dd, *J* = 13.5, 6.0 Hz, 1H), 2.52 (dd, *J* = 13.5, 8.3 Hz, 1H), 1.30 (d, *J* = 6.8 Hz, 3H).

^{13}C-NMR (101 MHz, $CDCl_3$) δ 168.3 (C_q), 150.0 (C_q), 148.9 (C_q), 142.1 (CH), 141.1 (C_q), 139.9

(C_q), 138.4 (C_q), 131.3 (CH), 129.0 (CH), 128.8 (CH), 128.2 (CH), 128.0 (C_q), 127.2 (CH), 126.1 (CH), 125.9 (C_q), 125.4 (CH), 123.2 (CH), 123.2 (CH), 122.6 (C_q), 122.1 (CH), 110.3 (CH), 101.4 (CH), 52.0 (CH_3), 43.4 (CH_2), 33.7 (CH), 19.9 (CH_3).

The analytic data of **18** are consistent with the literature.[13]

$[\alpha_D^{20}]$ = +54.6 (c = 1.0, DCM).

HPLC separation (Chiralpak® IF-3, *n*-hexane/*i*-PrOH 90:10, 1.0 mL/min, detection at 250 nm): t_r (major) = 17.8 min, t_r (minor) = 18.4 min, 50% e.e. (The racemic HPLC chromatogram of this compound refers to literature)[13].

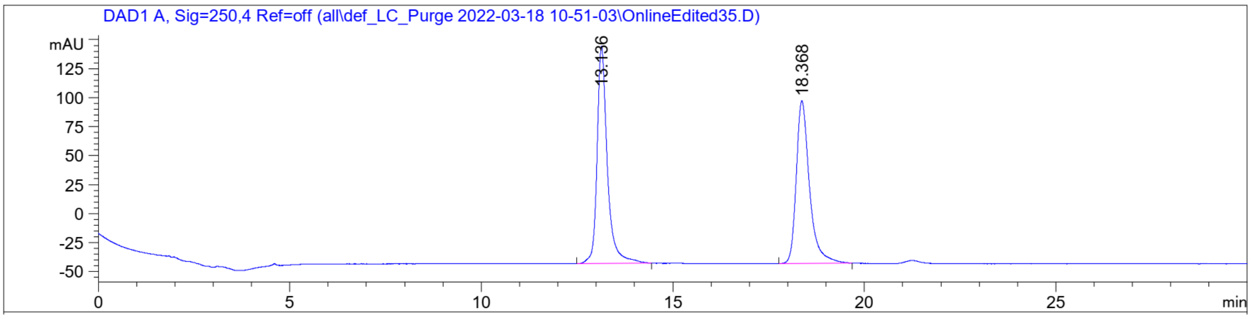

Peak #	RetTime [min]	Type	Width [min]	Area [mAU*s]	Height [mAU]	Area %
1	13.136	BV R	0.2752	3491.43896	187.49884	50.8940
2	18.368	VB R	0.3619	3368.77661	140.39731	49.1060

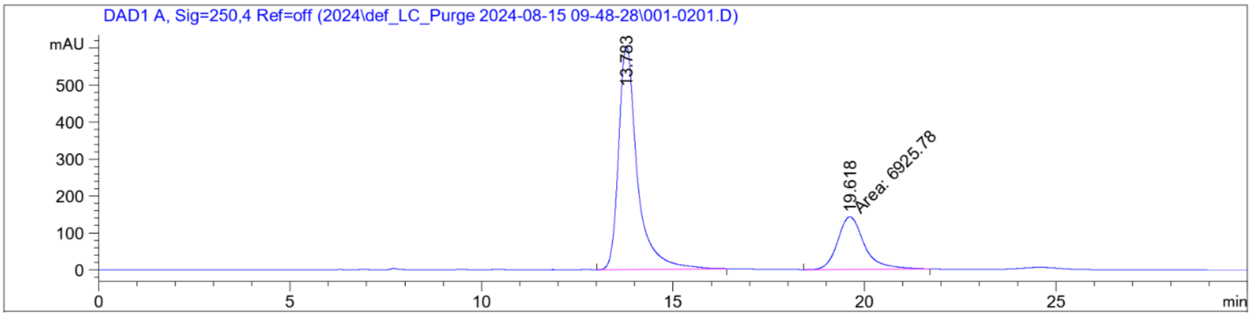

Peak #	RetTime [min]	Type	Width [min]	Area [mAU*s]	Height [mAU]	Area %
1	13.783	BB	0.5055	2.05259e4	605.50690	74.7710
2	19.618	MP	0.8115	6925.78027	142.23831	25.2290

Supplementary Section 7. DFT Calculations on the Rotation Barrier

In order to explore the possibility of racemization of the C−C axial chirality of product **3a**, the energetic barrier of the racemization process was calculated with Gaussian 16, Revision A.03 package.[14] The geometry optimizations were conducted at the B3LYP[15,16] level of theory in combination with Grimme's D3 dispersion corrections with a Becke-Johnson damping scheme (D3BJ)[17,18] in the gas phase. All atoms were described with a def2-SVP basis set[19,21]. Analytical frequency calculations were also carried out at the same level of theory to confirm each optimized stationary point is an energy minimum (zero imaginary frequencies) or a transition state (one imaginary frequency) and further to provide thermal and nonthermal corrections to the Gibbs free energy at 313.15 K and 1 atm. The single-point energies were further evaluated with M06-2X[23, 24] with a def2-TZVPP basis set[19,21]. Solvent effects were considered using the implicit solvation model SMD[25] with a dielectric constant of ε = 8.93, which corresponds to dichloromethane. All reported energies are based on gas-phase Gibbs free energies with def2-SVP basis set for which the electronic energies were corrected by M06-2X with a def2-TZVPP basis set and solvent effects. The rate constants for enantiomerization (k_{ent}) and racemization (k_{rac}), and half-life for racemization ($t_{1/2}$) were calculated based on the following equations:

$$k_{ent} = \kappa \frac{k_B T}{h} exp \frac{-\Delta G^{\ddagger}}{RT}$$

$$k_{\text{rac}} = 2k_{\text{ent}}$$

$$t_{1/2} = \frac{\ln 2}{k_{\text{rac}}}$$

The transmission coefficient is set as 1, Boltzmann constant k_B = 1.3806503×10^{-23} J·K^{-1}, Planck constant h = 6.62606876×10^{-34} J·s, idea gas constant R = 8.314472 J·mol^{-1}·K^{-1}, T = 313.15 K.

The DFT calculation results are shown below:

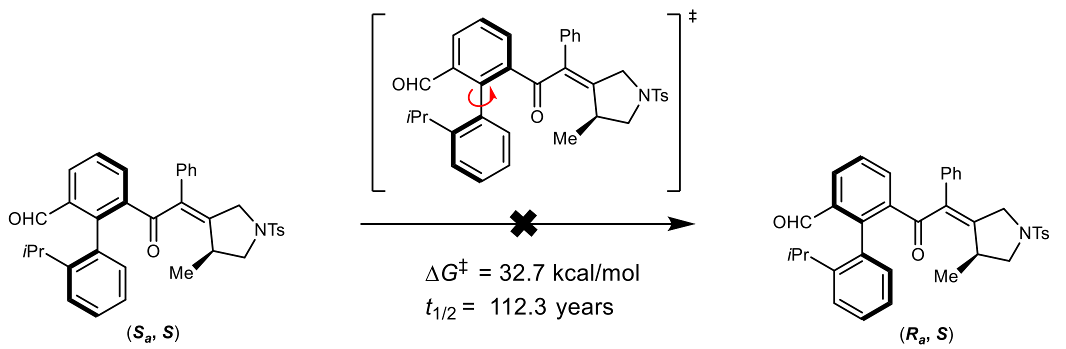

Supplementary Figure 7. Computed rotational barriers ($\Delta G_{313.15}$) in kcal mol^{-1} for the racemization of product **3a** and the corresponding half-life at the M06-2X-D3/def2-TZVPP-SMD(DCM)//B3LYP-D3(BJ)/def2-SVP level of theory.

Supplementary Table 9. Calculated electronic energies at the M06-2X-D3/def2-TZVPP-SMD(DCM) level of theory and Gibbs free energies with dispersion corrections for all structures (all in Hartree).

Structure	Electronic Energy	Total Gibbs Free Energy
**3a** (S_a,S)	-2146.162305	-2145.612289
TS	-2146.113574	-2145.560175
**3a** (R_a,S)	-2146.161170	-2145.612661

Supplementary Section 8. NMR Spectra

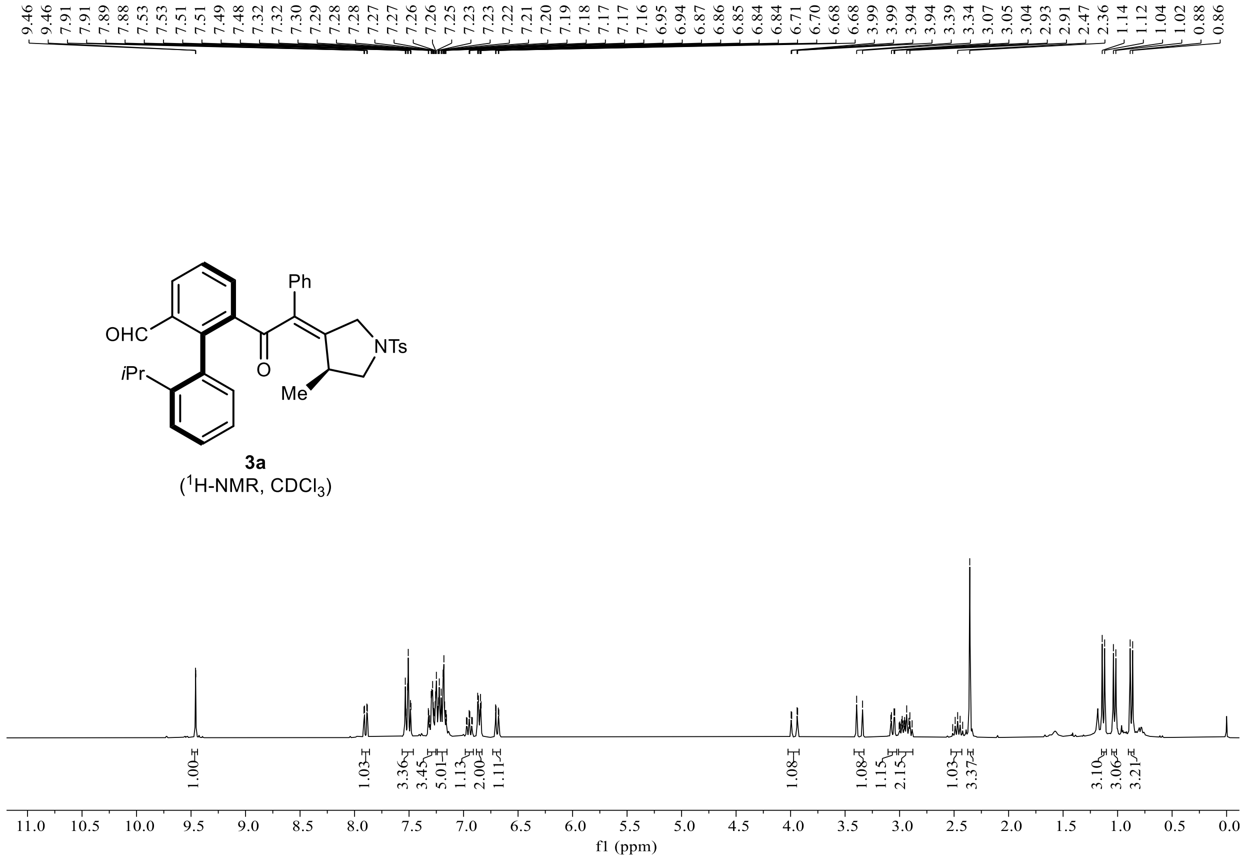

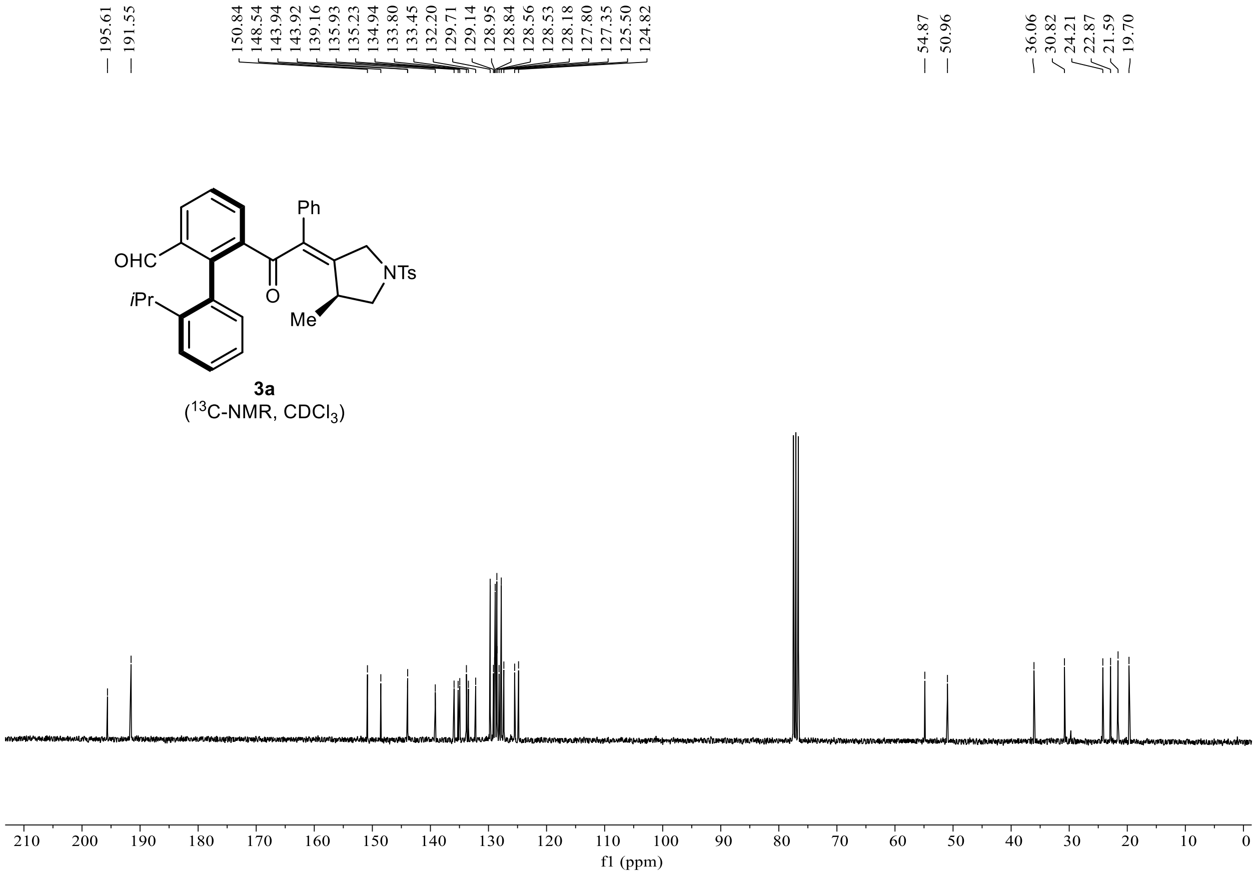

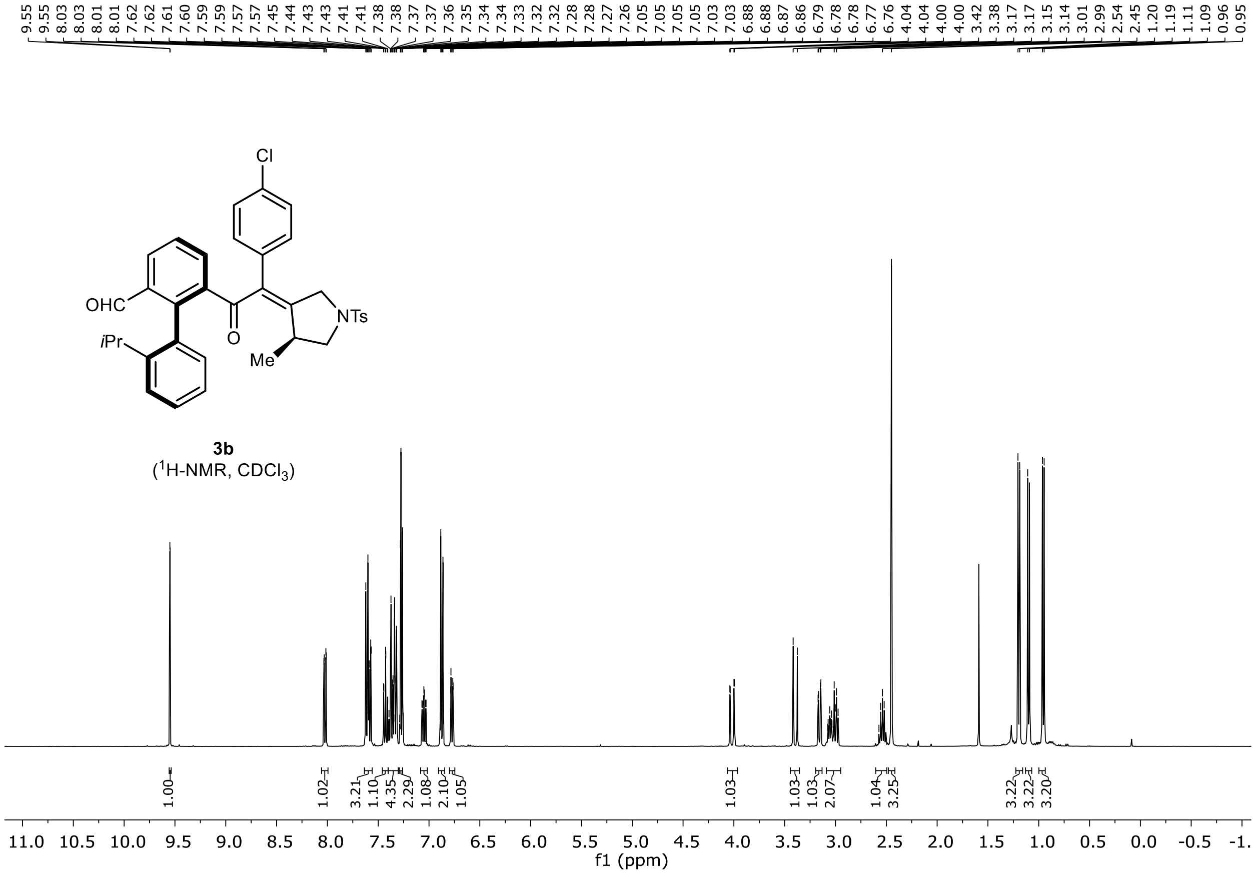

3b
(^{1}H-NMR, $CDCl_3$)

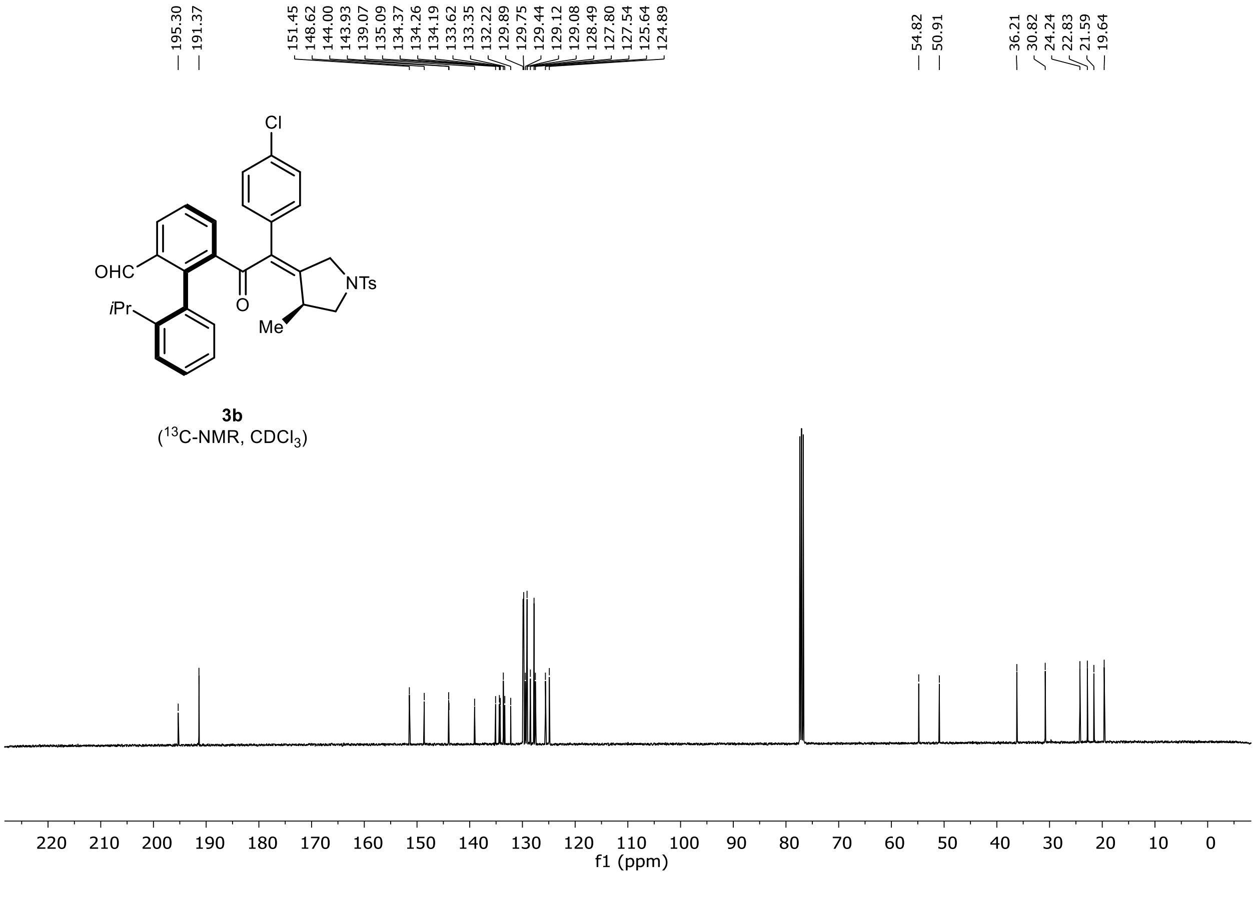

3b
(^{13}C-NMR, $CDCl_3$)

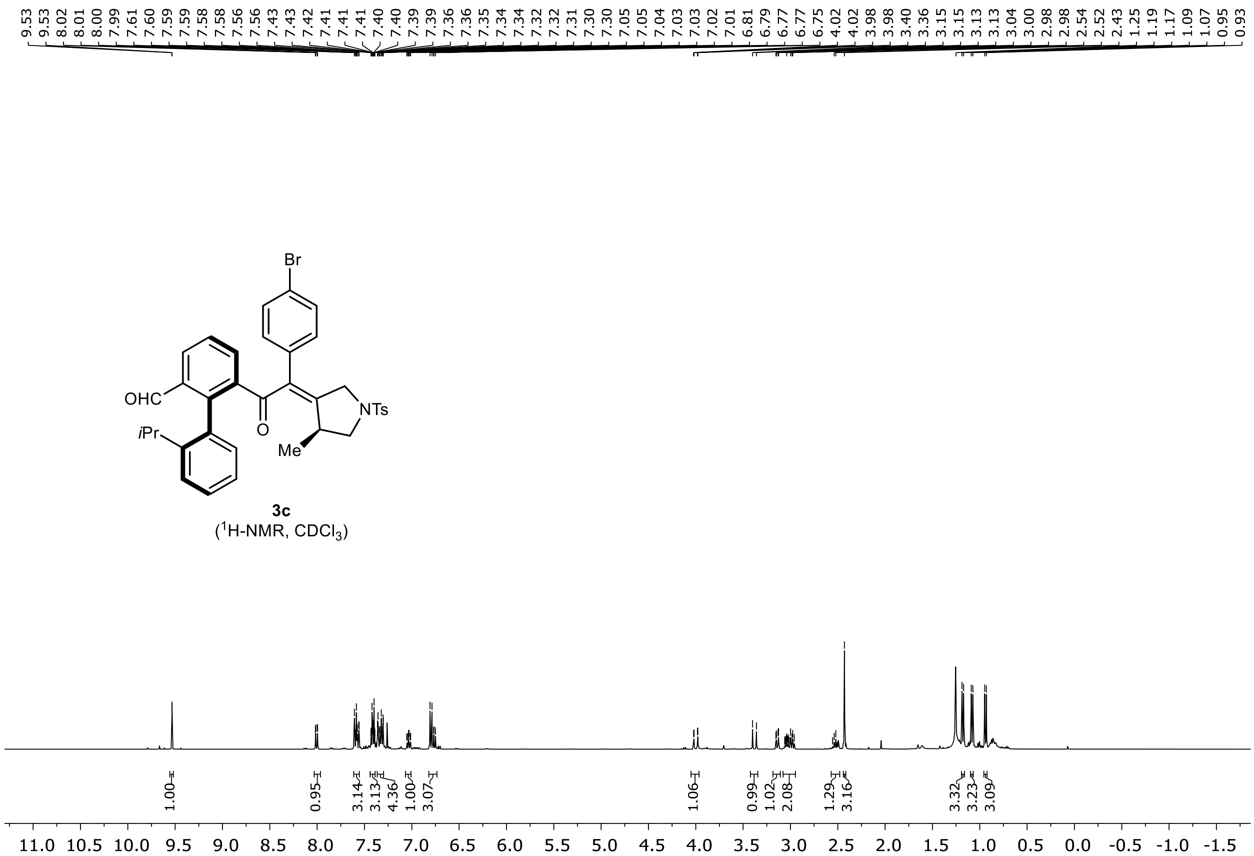
9.53 9.53 8.02 8.01 8.00 7.99 7.61 7.60 7.59 7.59 7.58 7.58 7.56 7.56 7.43 7.43 7.42 7.41 7.41 7.41 7.40 7.40 7.39 7.39 7.36 7.36 7.35 7.34 7.34 7.32 7.32 7.31 7.30 7.30 7.05 7.05 7.04 7.03 7.03 7.02 7.01 6.81 6.79 6.77 6.77 6.75 4.02 4.02 3.98 3.98 3.40 3.36 3.15 3.15 3.13 3.13 3.04 3.00 2.98 2.98 2.54 2.52 2.43 1.25 1.19 1.17 1.09 1.07 0.95 0.93
Br
OHC
O
NTs
iPr
Me
3c
(1H-NMR, CDCl3)
1.00 0.95 3.14 3.13 4.36 1.00 3.07 1.06 0.99 1.02 2.08 1.29 3.16 3.32 3.23 3.09
11.0 10.5 10.0 9.5 9.0 8.5 8.0 7.5 7.0 6.5 6.0 5.5 5.0 4.5 4.0 3.5 3.0 2.5 2.0 1.5 1.0 0.5 0.0 -0.5 -1.0 -1.5
f1 (ppm)

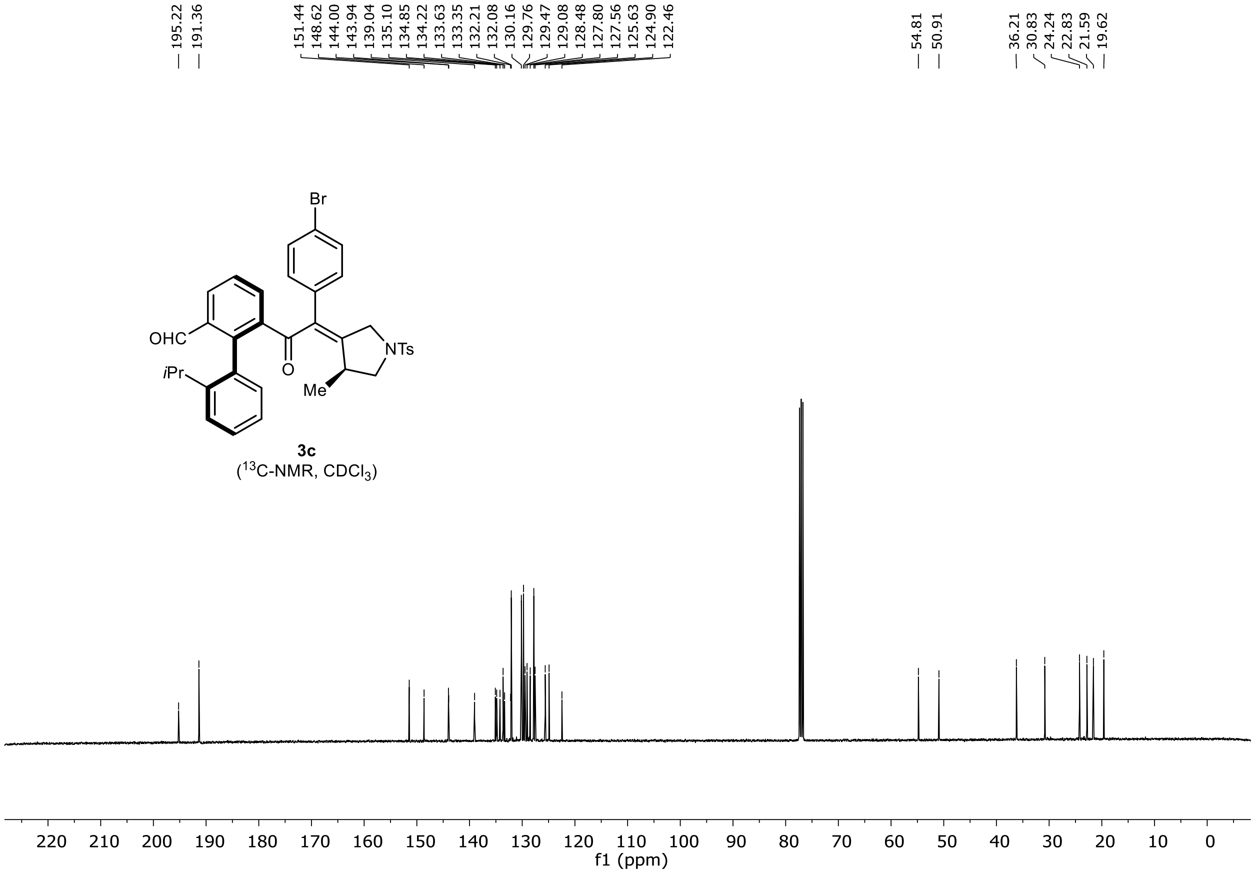
195.22 191.36 151.44 148.62 144.00 143.94 139.04 135.10 134.85 134.22 133.63 133.35 132.21 132.08 130.16 129.76 129.47 129.08 128.48 127.80 127.56 125.63 124.90 122.46 54.81 50.91 36.21 30.83 24.24 22.83 21.59 19.62
Br
OHC
O
NTs
iPr
Me
3c
(13C-NMR, CDCl3)
220 210 200 190 180 170 160 150 140 130 120 110 100 90 80 70 60 50 40 30 20 10 0
f1 (ppm)

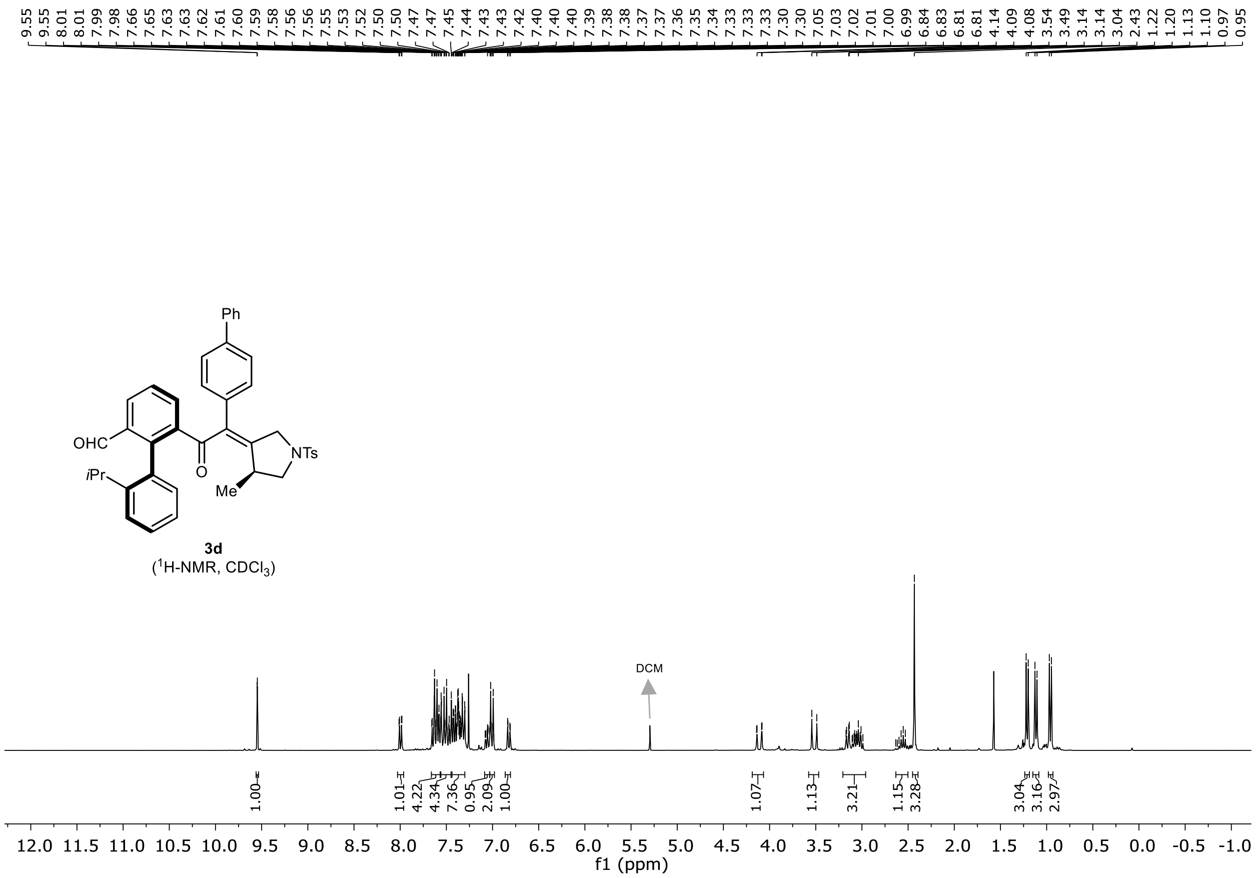
9.55 9.55 8.01 8.01 7.99 7.98 7.66 7.65 7.63 7.63 7.62 7.61 7.60 7.59 7.58 7.56 7.56 7.55 7.53 7.52 7.50 7.47 7.45 7.44 7.43 7.43 7.42 7.40 7.40 7.39 7.38 7.38 7.37 7.37 7.36 7.35 7.34 7.33 7.33 7.33 7.30 7.30 7.05 7.03 7.02 7.01 7.00 6.99 6.84 6.83 6.81 6.81 4.14 4.09 4.08 3.54 3.49 3.14 3.14 3.04 2.43 1.22 1.20 1.13 1.10 0.97 0.95
Ph
OHC
O
NTs
iPr
Me
3d
(^{1}H-NMR, CDCl$_3$)
DCM
1.00 1.01 4.22 4.34 7.36 0.95 2.09 1.00 1.07 1.13 3.21 1.15 3.28 3.04 3.16 2.97
12.0 11.5 11.0 10.5 10.0 9.5 9.0 8.5 8.0 7.5 7.0 6.5 6.0 5.5 5.0 4.5 4.0 3.5 3.0 2.5 2.0 1.5 1.0 0.5 0.0 -0.5 -1.0
f1 (ppm)

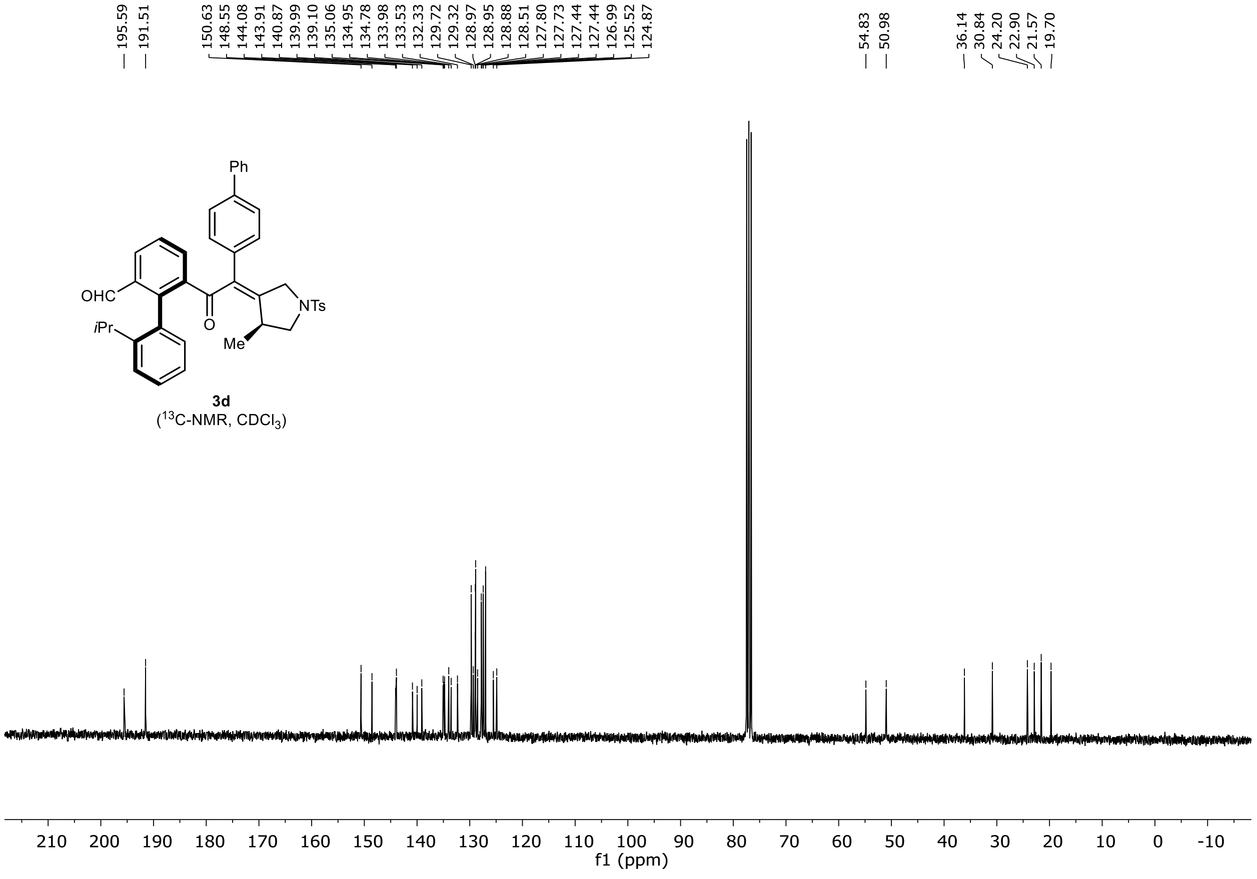
195.59 191.51 150.63 148.55 144.08 143.91 140.87 139.99 139.10 135.06 134.95 134.78 133.98 133.53 132.33 129.72 129.32 128.97 128.95 128.88 128.51 127.80 127.73 127.44 126.99 125.52 124.87 54.83 50.98 36.14 30.84 24.20 22.90 21.57 19.70
Ph
OHC
O
NTs
iPr
Me
3d
(^{13}C-NMR, CDCl$_3$)
210 200 190 180 170 160 150 140 130 120 110 100 90 80 70 60 50 40 30 20 10 0 -10
f1 (ppm)

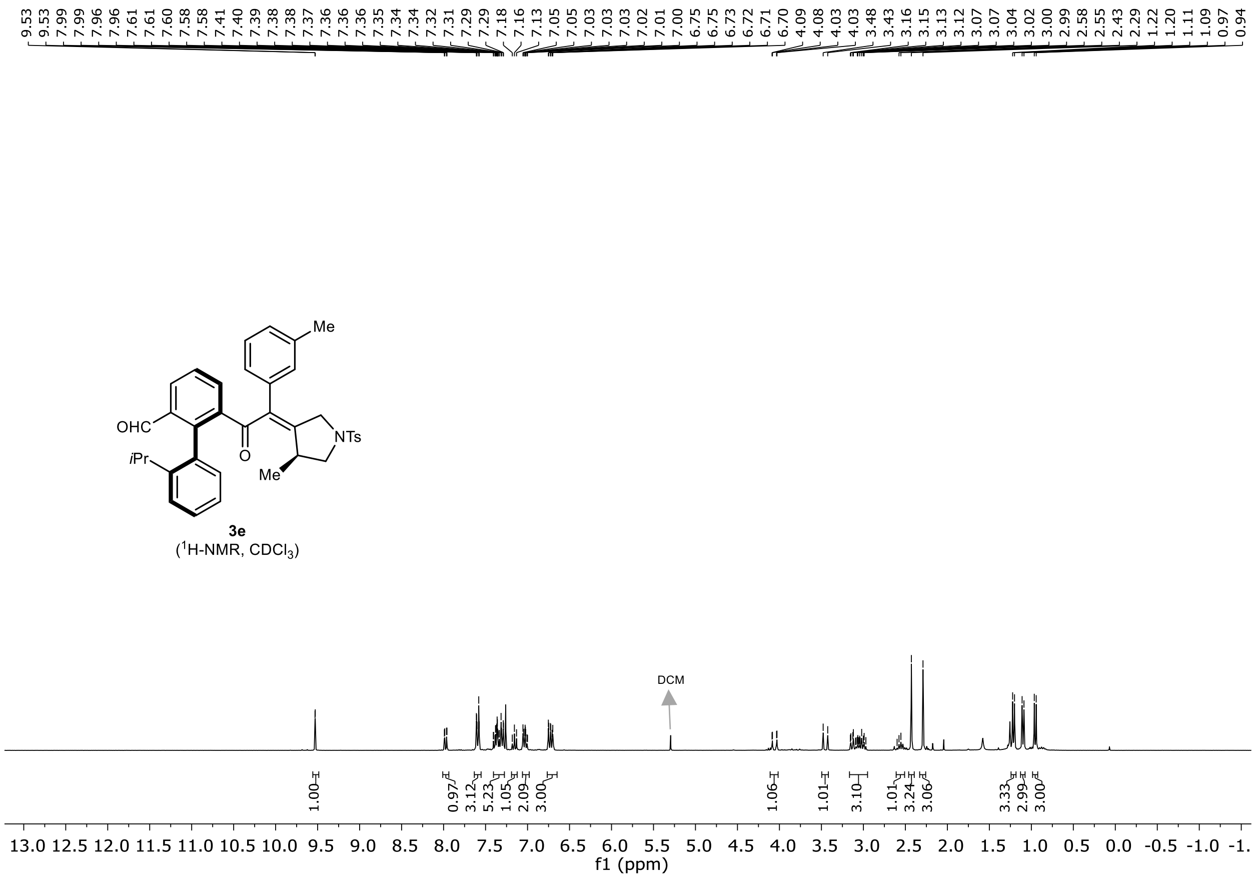
9.53 9.53 7.99 7.99 7.96 7.96 7.61 7.61 7.60 7.58 7.58 7.41 7.40 7.39 7.38 7.38 7.37 7.36 7.36 7.36 7.35 7.34 7.34 7.32 7.32 7.31 7.29 7.29 7.18 7.16 7.13 7.05 7.05 7.03 7.03 7.02 7.01 7.00 6.75 6.75 6.73 6.72 6.71 6.70 4.09 4.08 4.03 4.03 3.48 3.43 3.16 3.15 3.13 3.12 3.07 3.07 3.04 3.02 3.00 2.99 2.58 2.55 2.43 2.29 1.22 1.20 1.11 1.09 0.97 0.94
OHC
iPr
O
NTs
Me
Me
3e
(1H-NMR, CDCl3)
DCM
1.00
0.97
3.12
5.23
1.05
2.09
3.00
1.06
1.01
3.10
1.01
3.24
3.06
3.33
2.99
3.00
13.0 12.5 12.0 11.5 11.0 10.5 10.0 9.5 9.0 8.5 8.0 7.5 7.0 6.5 6.0 5.5 5.0 4.5 4.0 3.5 3.0 2.5 2.0 1.5 1.0 0.5 0.0 -0.5 -1.0 -1.
f1 (ppm)

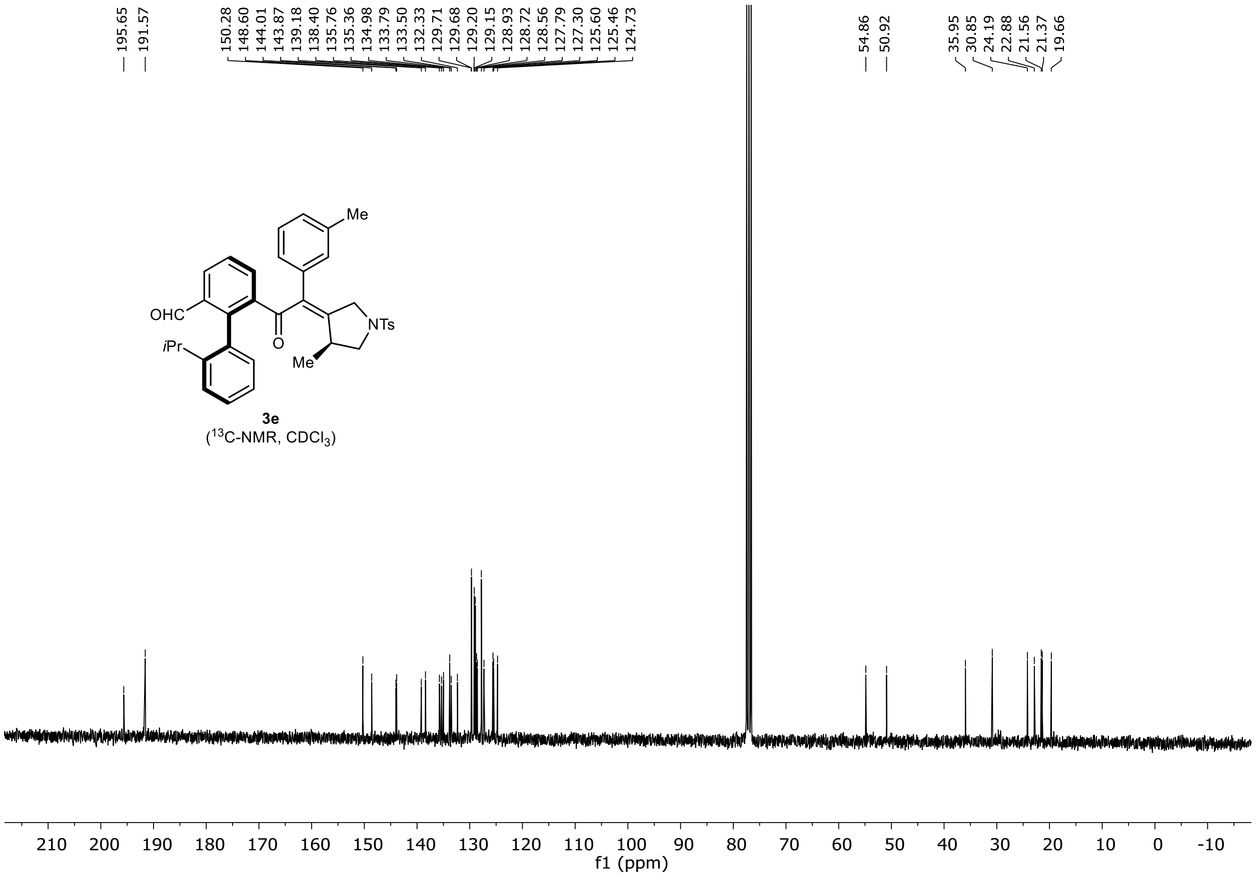
195.65 191.57
150.28 148.60 144.01 143.87 139.18 138.40 135.76 135.36 134.98 133.79 133.50 132.33 129.71 129.68 129.20 129.15 128.93 128.72 128.56 127.79 127.30 125.60 125.46 124.73
54.86 50.92
35.95 30.85 24.19 22.88 21.56 21.37 19.66
OHC
iPr
O
NTs
Me
Me
3e
(13C-NMR, CDCl3)
210 200 190 180 170 160 150 140 130 120 110 100 90 80 70 60 50 40 30 20 10 0 -10
f1 (ppm)

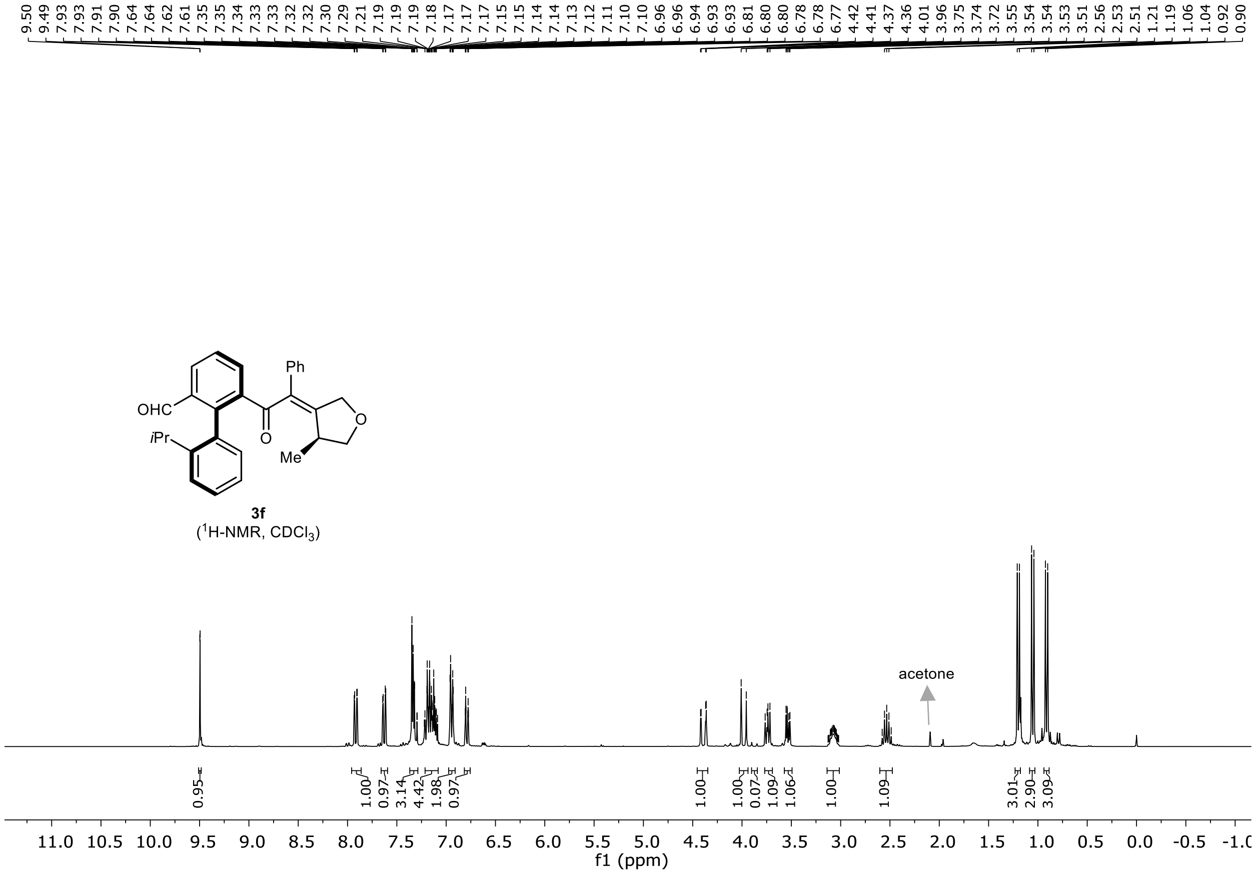
9.50 9.49 7.93 7.93 7.91 7.90 7.64 7.62 7.61 7.35 7.35 7.34 7.33 7.33 7.32 7.32 7.30 7.29 7.21 7.19 7.19 7.19 7.18 7.17 7.17 7.17 7.15 7.15 7.14 7.14 7.13 7.12 7.11 7.10 7.10 6.96 6.96 6.94 6.93 6.93 6.81 6.80 6.80 6.78 6.78 6.77 4.42 4.41 4.37 4.36 4.01 3.96 3.75 3.74 3.72 3.55 3.54 3.54 3.53 3.51 2.56 2.53 2.51 1.21 1.19 1.06 1.04 0.92 0.90
Ph
OHC
O
O
iPr
Me
3f
(^{1}H-NMR, $CDCl_3$)
acetone
0.95 1.00 0.97 3.14 4.42 1.98 0.97 1.00 1.00 0.07 1.09 1.06 1.00 1.09 3.01 2.90 3.09
11.0 10.5 10.0 9.5 9.0 8.5 8.0 7.5 7.0 6.5 6.0 5.5 5.0 4.5 4.0 3.5 3.0 2.5 2.0 1.5 1.0 0.5 0.0 -0.5 -1.0
f1 (ppm)

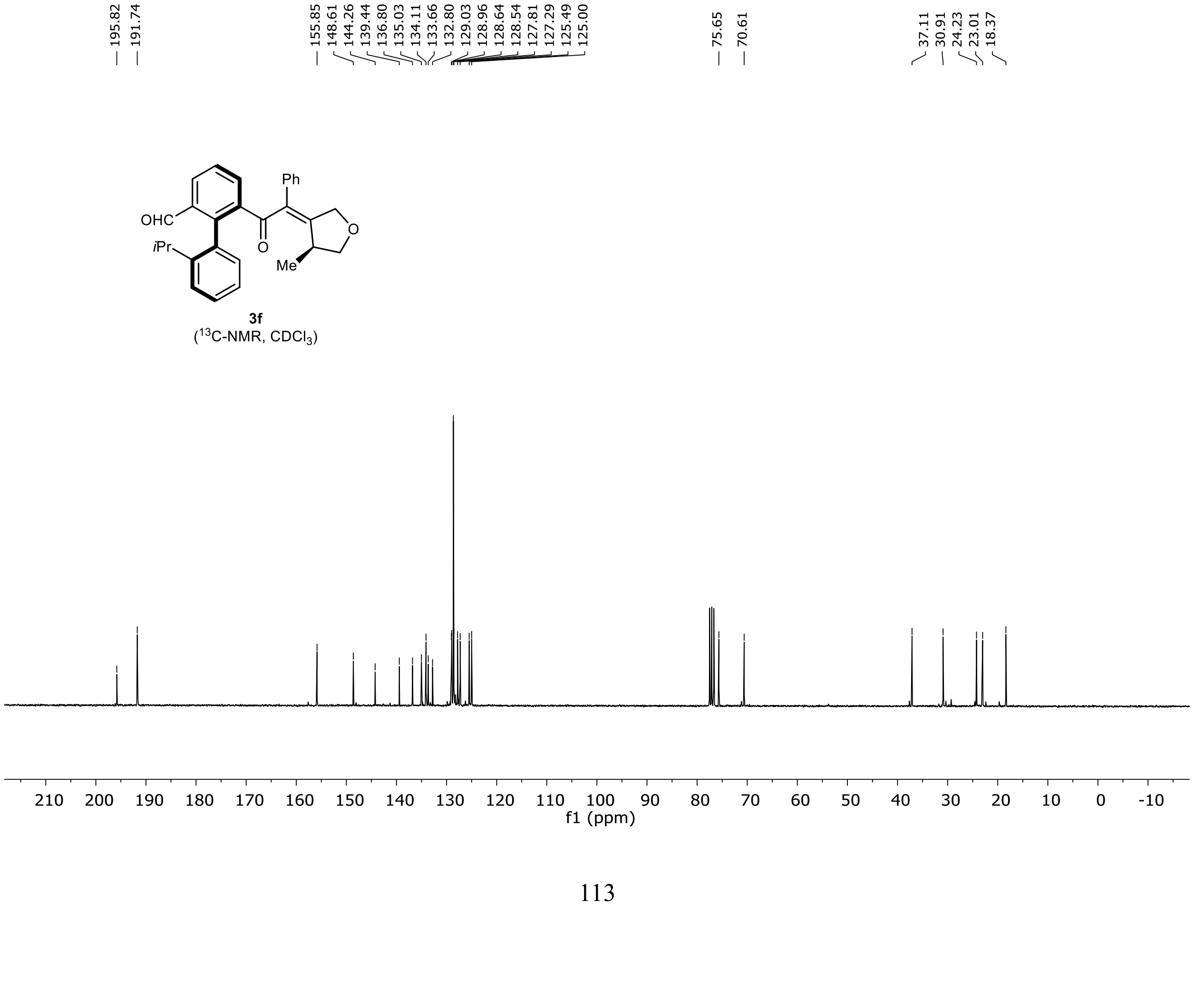
195.82 191.74 155.85 148.61 144.26 139.44 136.80 135.03 134.11 133.66 132.80 129.03 128.96 128.64 128.54 127.81 127.29 125.49 125.00 75.65 70.61 37.11 30.91 24.23 23.01 18.37
Ph
OHC
O
O
iPr
Me
3f
(^{13}C-NMR, $CDCl_3$)
210 200 190 180 170 160 150 140 130 120 110 100 90 80 70 60 50 40 30 20 10 0 -10
f1 (ppm)

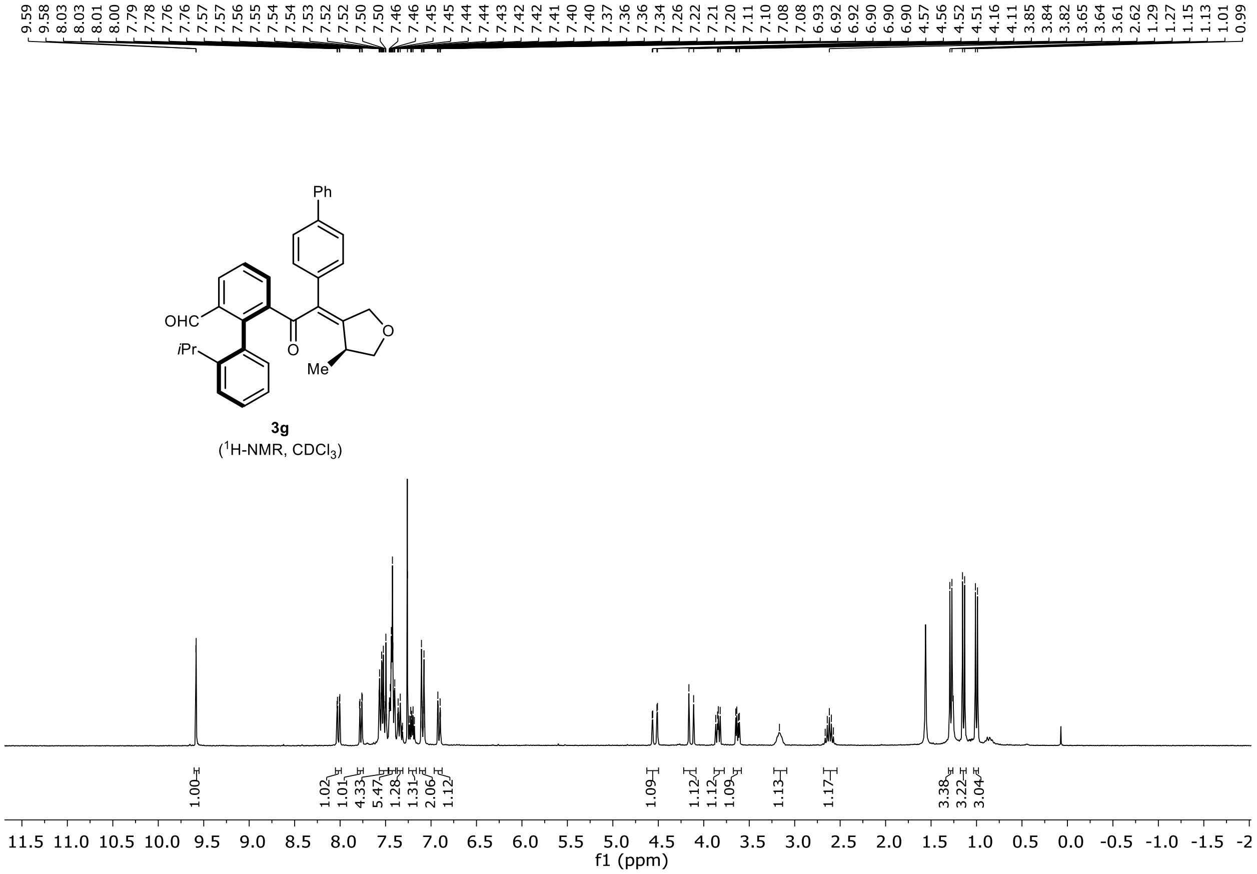

9.59
9.58
8.03
8.01
8.00
7.79
7.78
7.76
7.76
7.57
7.57
7.56
7.55
7.54
7.54
7.53
7.52
7.52
7.50
7.50
7.46
7.46
7.45
7.45
7.44
7.44
7.43
7.42
7.42
7.41
7.40
7.40
7.37
7.36
7.34
7.26
7.22
7.21
7.20
7.11
7.10
7.08
7.08
6.93
6.92
6.92
6.90
6.90
6.90
4.57
4.56
4.52
4.51
4.16
4.11
3.85
3.84
3.82
3.65
3.64
3.61
2.62
1.29
1.27
1.15
1.13
1.01
0.99
Ph
OHC
O
O
iPr
Me
3g
(1H-NMR, CDCl3)
1.00
1.02
1.01
4.33
5.47
1.28
1.31
2.06
1.12
1.09
1.12
1.12
1.09
1.13
1.17
3.38
3.22
3.04
11.5 11.0 10.5 10.0 9.5 9.0 8.5 8.0 7.5 7.0 6.5 6.0 5.5 5.0 4.5 4.0 3.5 3.0 2.5 2.0 1.5 1.0 0.5 0.0 -0.5 -1.0 -1.5 -2
f1 (ppm)

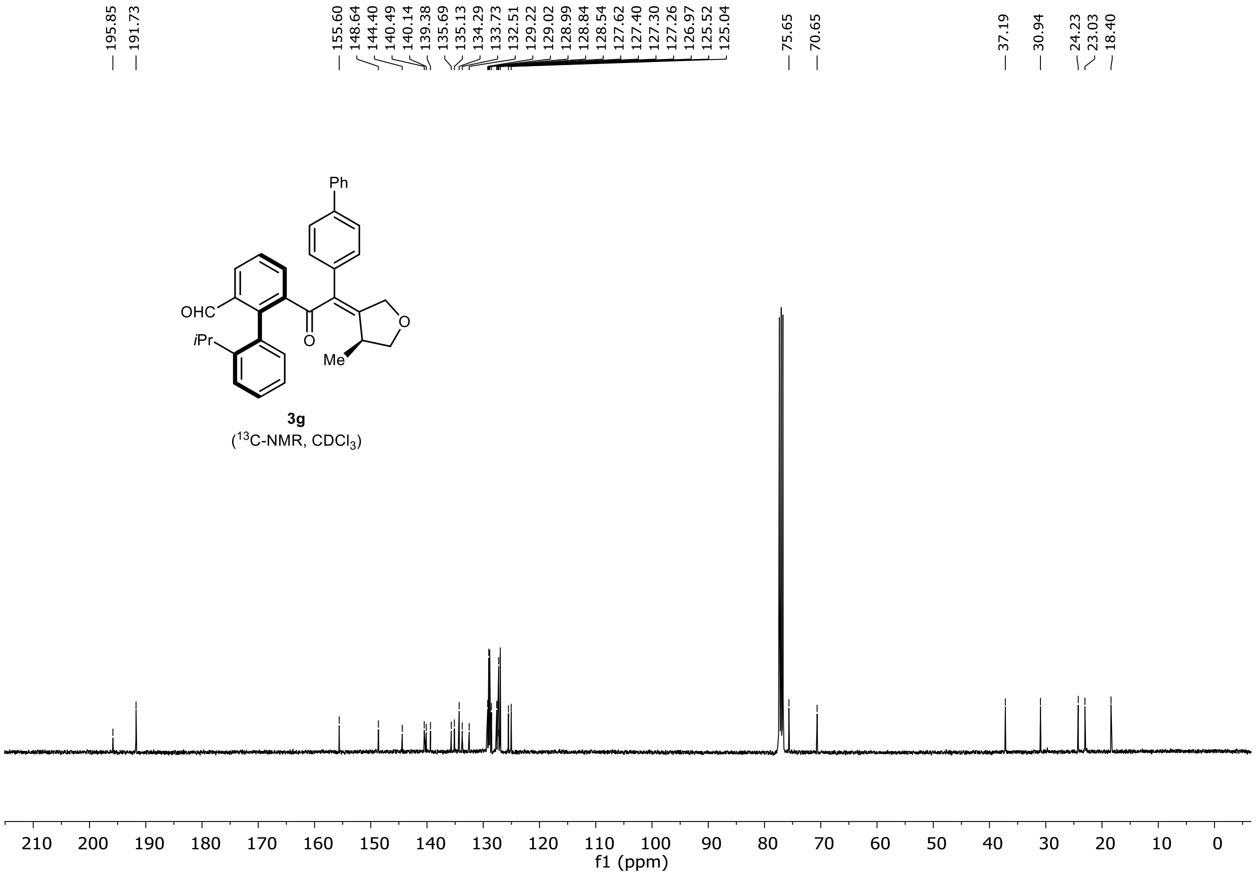

195.85
191.73
155.60
148.64
144.40
140.49
140.14
139.38
135.69
135.13
134.29
133.73
132.51
129.22
129.02
128.99
128.84
128.54
127.62
127.40
127.30
127.26
126.97
125.52
125.04
75.65
70.65
37.19
30.94
24.23
23.03
18.40
Ph
OHC
O
O
iPr
Me
3g
(13C-NMR, CDCl3)
210 200 190 180 170 160 150 140 130 120 110 100 90 80 70 60 50 40 30 20 10 0
f1 (ppm)

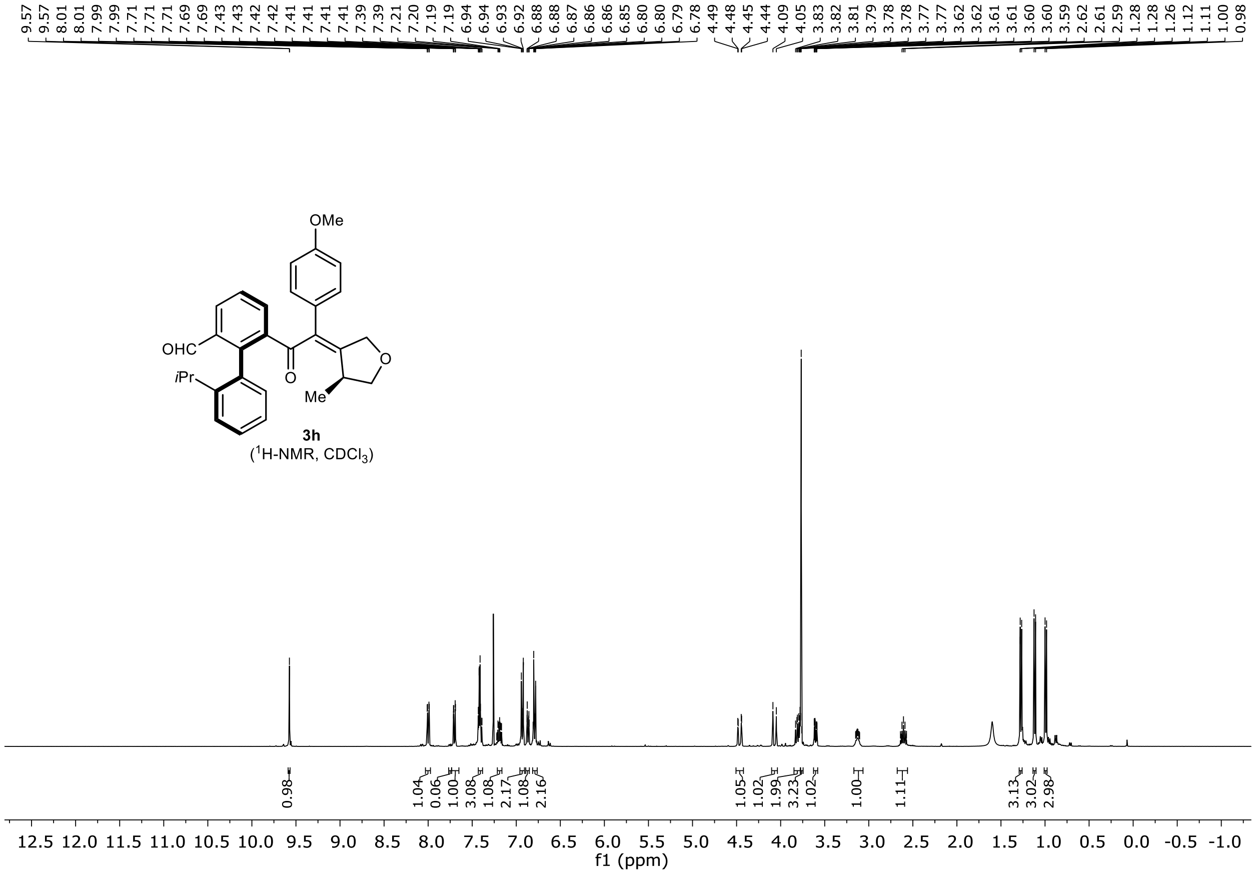
9.57 9.57 8.01 8.01 7.99 7.99 7.71 7.71 7.71 7.69 7.69 7.43 7.43 7.42 7.42 7.41 7.41 7.41 7.41 7.41 7.39 7.39 7.21 7.20 7.19 7.19 6.94 6.94 6.93 6.92 6.88 6.88 6.87 6.86 6.86 6.85 6.80 6.80 6.79 6.78 4.49 4.48 4.45 4.44 4.09 4.05 3.83 3.82 3.81 3.79 3.78 3.78 3.77 3.77 3.62 3.62 3.61 3.61 3.60 3.59 2.62 2.61 2.59 1.28 1.28 1.26 1.12 1.11 1.00 0.98
OMe
OHC
O
O
iPr
Me
3h
(^{1}H-NMR, $CDCl_3$)
0.98 1.04 0.06 1.00 3.08 1.08 2.17 1.08 2.16 1.05 1.02 1.99 3.23 1.02 1.00 1.11 3.13 3.02 2.98
12.5 12.0 11.5 11.0 10.5 10.0 9.5 9.0 8.5 8.0 7.5 7.0 6.5 6.0 5.5 5.0 4.5 4.0 3.5 3.0 2.5 2.0 1.5 1.0 0.5 0.0 -0.5 -1.0
f1 (ppm)

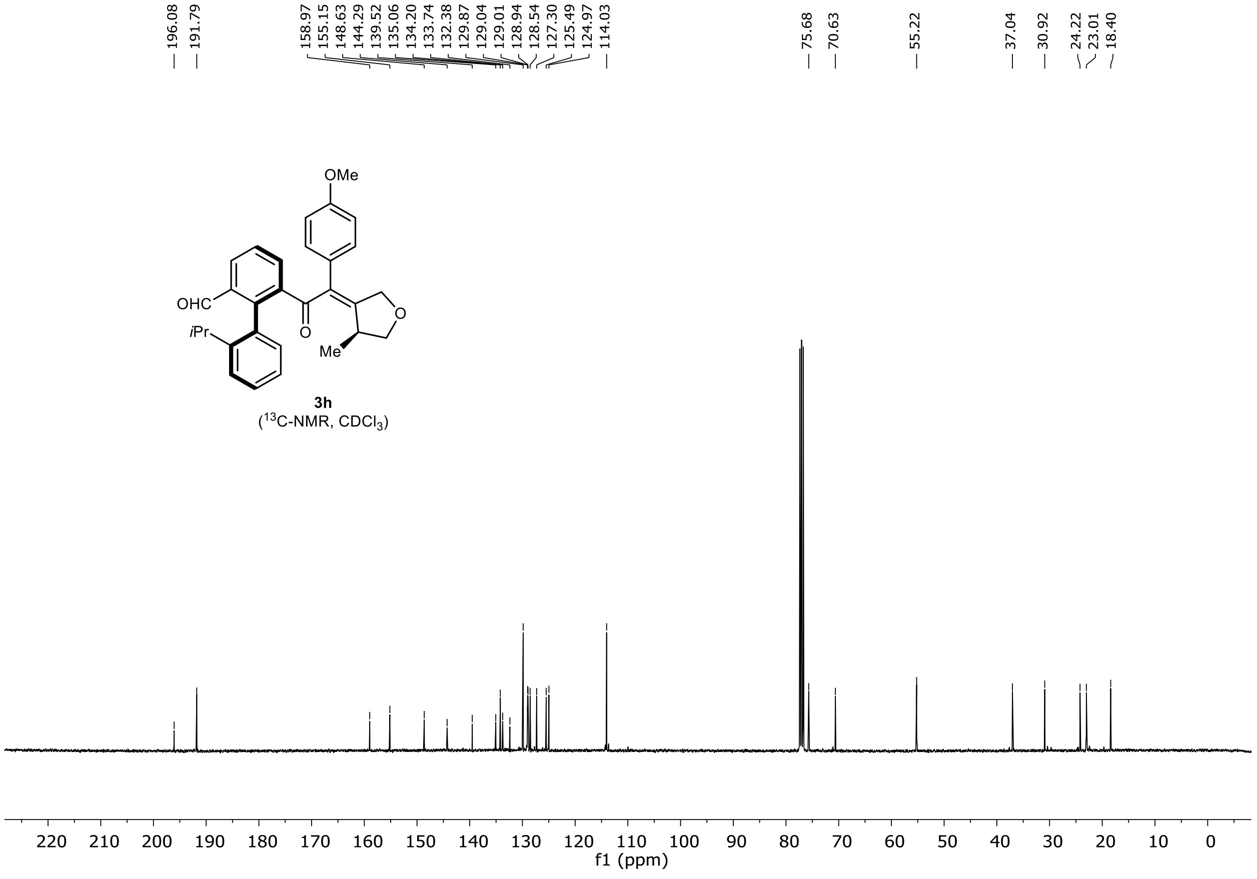
196.08 191.79 158.97 155.15 148.63 144.29 139.52 135.06 134.20 133.74 132.38 129.87 129.04 129.01 128.94 128.54 127.30 125.49 124.97 114.03 75.68 70.63 55.22 37.04 30.92 24.22 23.01 18.40
OMe
OHC
O
O
iPr
Me
3h
(^{13}C-NMR, $CDCl_3$)
220 210 200 190 180 170 160 150 140 130 120 110 100 90 80 70 60 50 40 30 20 10 0
f1 (ppm)

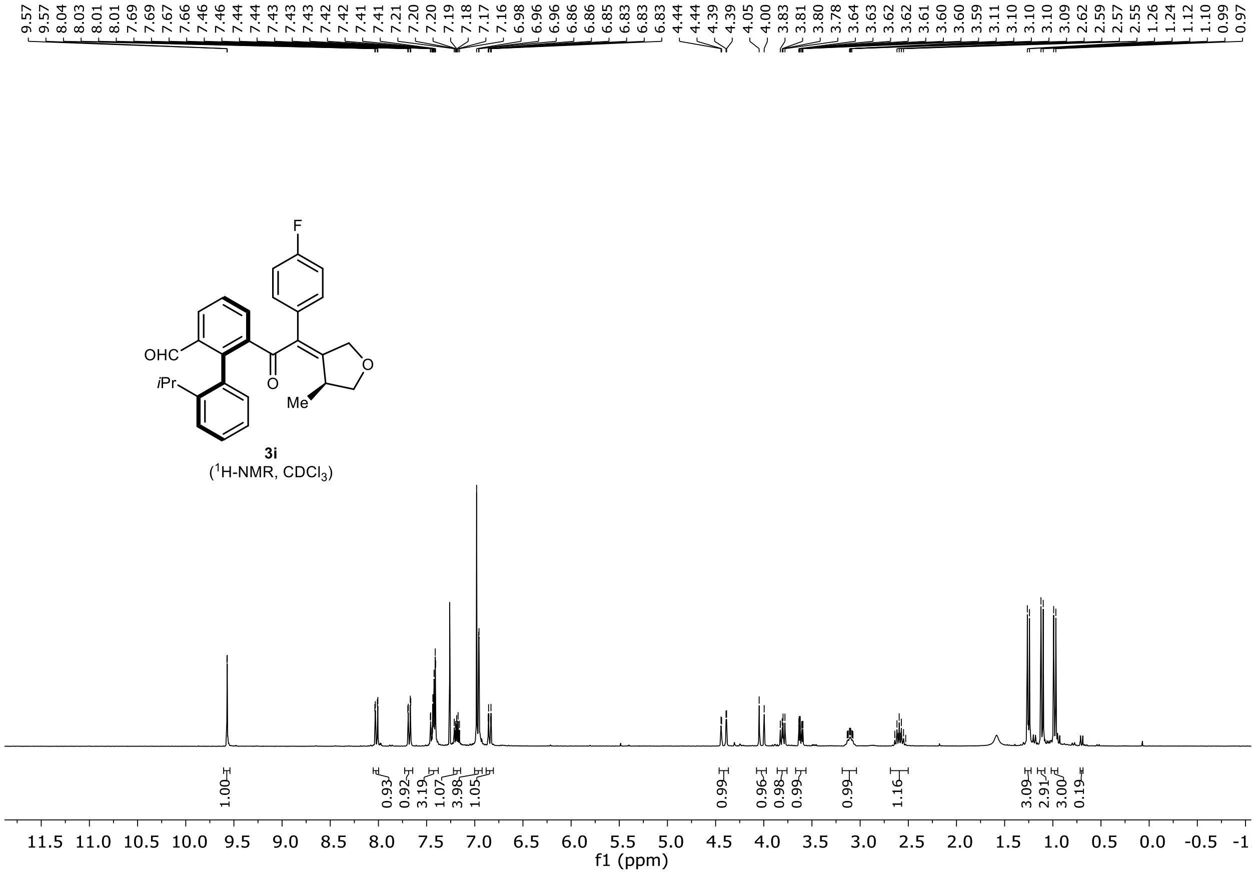
9.57 9.57 8.04 8.03 8.01 8.01 7.69 7.69 7.67 7.66 7.46 7.46 7.44 7.44 7.43 7.43 7.43 7.42 7.42 7.41 7.41 7.21 7.20 7.20 7.19 7.18 7.17 7.16 6.98 6.96 6.96 6.86 6.86 6.85 6.83 6.83 4.44 4.44 4.39 4.39 4.05 4.00 3.83 3.81 3.80 3.78 3.64 3.63 3.62 3.62 3.61 3.60 3.60 3.59 3.11 3.10 3.10 3.09 2.62 2.59 2.57 2.55 1.26 1.24 1.12 1.10 0.99 0.97
F
OHC
O
O
iPr
Me
3i
(1H-NMR, CDCl3)
1.00
0.93 0.92 3.19 1.07 3.98 1.05
0.99
0.96 0.98 0.99
0.99
1.16
3.09 2.91 3.00 0.19
11.5 11.0 10.5 10.0 9.5 9.0 8.5 8.0 7.5 7.0 6.5 6.0 5.5 5.0 4.5 4.0 3.5 3.0 2.5 2.0 1.5 1.0 0.5 0.0 -0.5 -1
f1 (ppm)

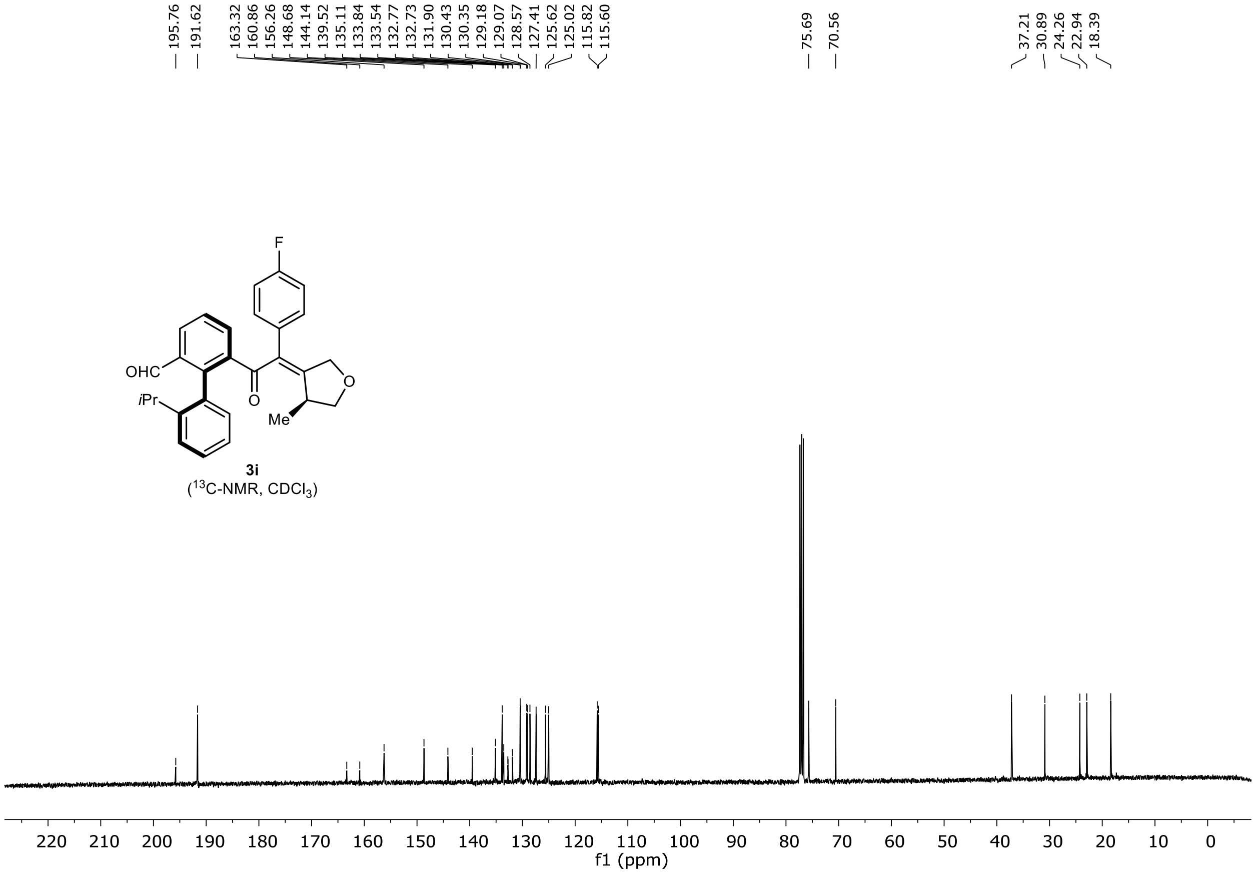
195.76 191.62 163.32 160.86 156.26 148.68 144.14 139.52 135.11 133.84 133.54 132.77 132.73 131.90 130.43 130.35 129.18 129.07 128.57 127.41 125.62 125.02 115.82 115.60 75.69 70.56 37.21 30.89 24.26 22.94 18.39
F
OHC
O
O
iPr
Me
3i
(13C-NMR, CDCl3)
220 210 200 190 180 170 160 150 140 130 120 110 100 90 80 70 60 50 40 30 20 10 0
f1 (ppm)

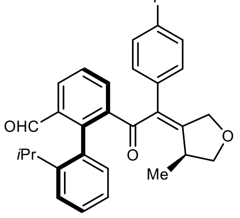

3i
(^{19}F-NMR, $CDCl_3$)

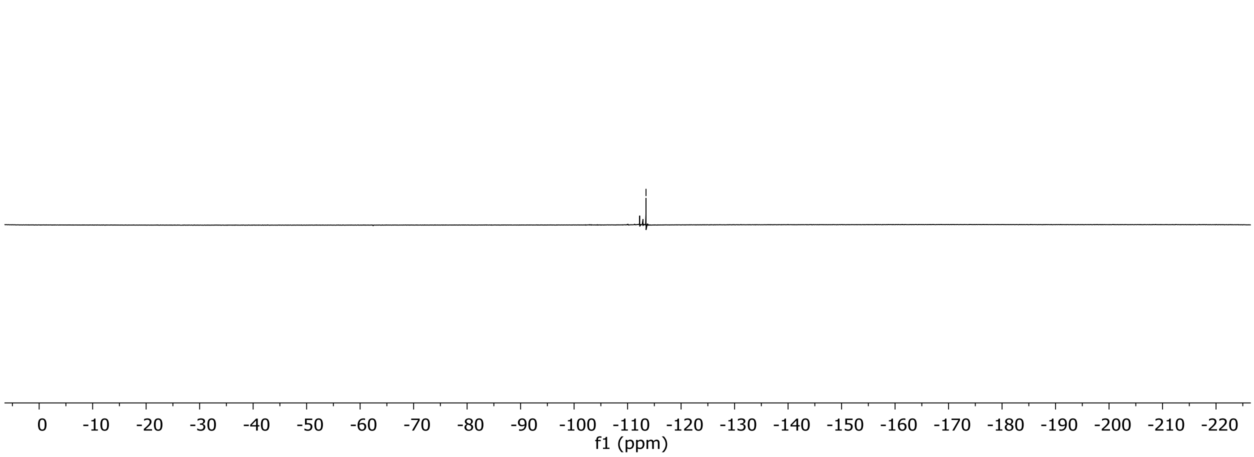

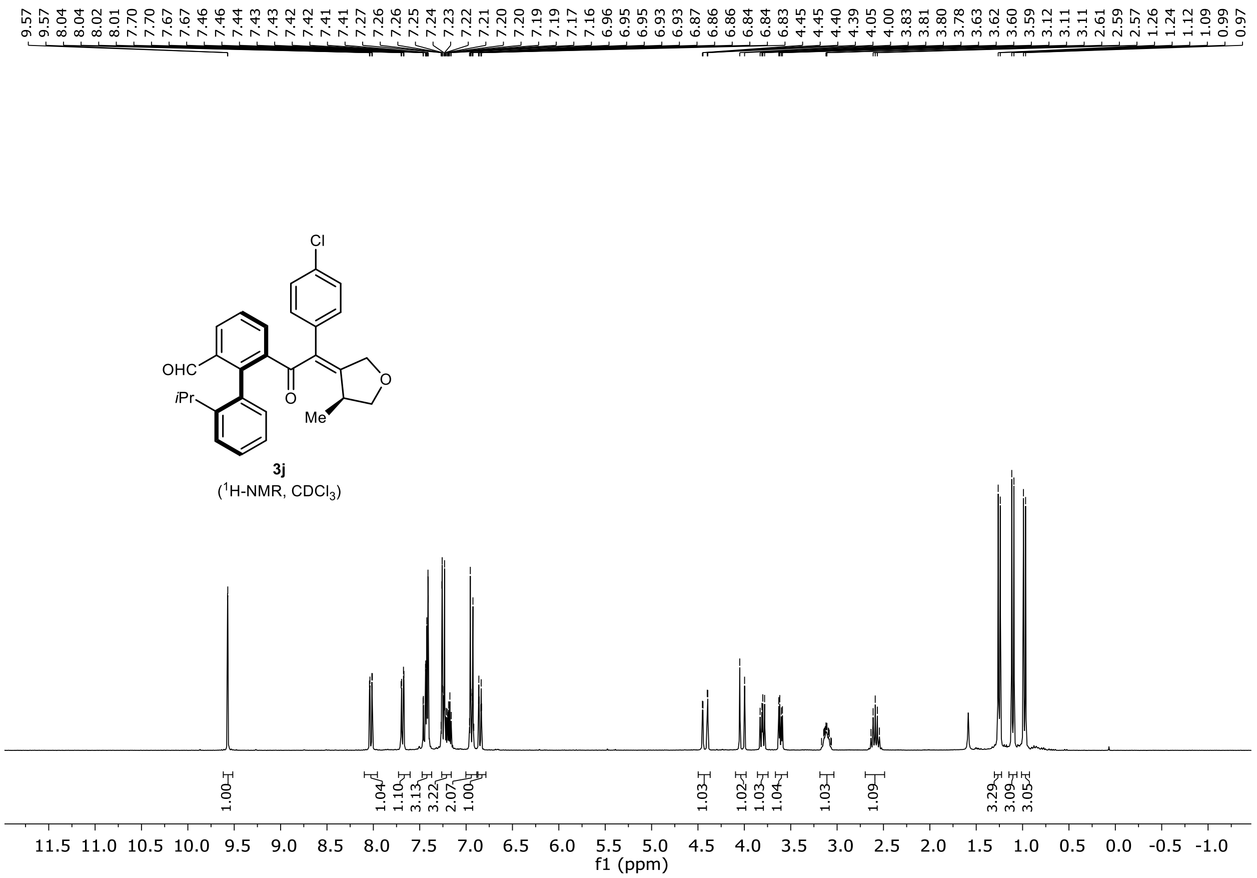
9.57 9.57 8.04 8.04 8.02 8.01 7.70 7.70 7.67 7.67 7.46 7.46 7.44 7.43 7.43 7.42 7.42 7.41 7.41 7.27 7.26 7.26 7.25 7.24 7.23 7.23 7.22 7.21 7.20 7.20 7.19 7.19 7.17 7.16 6.96 6.95 6.93 6.93 6.87 6.86 6.84 6.84 6.83 4.45 4.45 4.40 4.39 4.05 4.00 3.83 3.81 3.80 3.78 3.63 3.62 3.60 3.59 3.12 3.11 3.11 2.61 2.59 2.57 1.26 1.24 1.12 1.09 0.99 0.97
Cl
OHC
O
O
*i*Pr
Me
3j
(^{1}H-NMR, $CDCl_3$)
1.00 1.04 1.10 3.13 3.22 2.07 1.00 1.03 1.02 1.03 1.04 1.03 1.09 3.29 3.09 3.05
11.5 11.0 10.5 10.0 9.5 9.0 8.5 8.0 7.5 7.0 6.5 6.0 5.5 5.0 4.5 4.0 3.5 3.0 2.5 2.0 1.5 1.0 0.5 0.0 -0.5 -1.0
f1 (ppm)

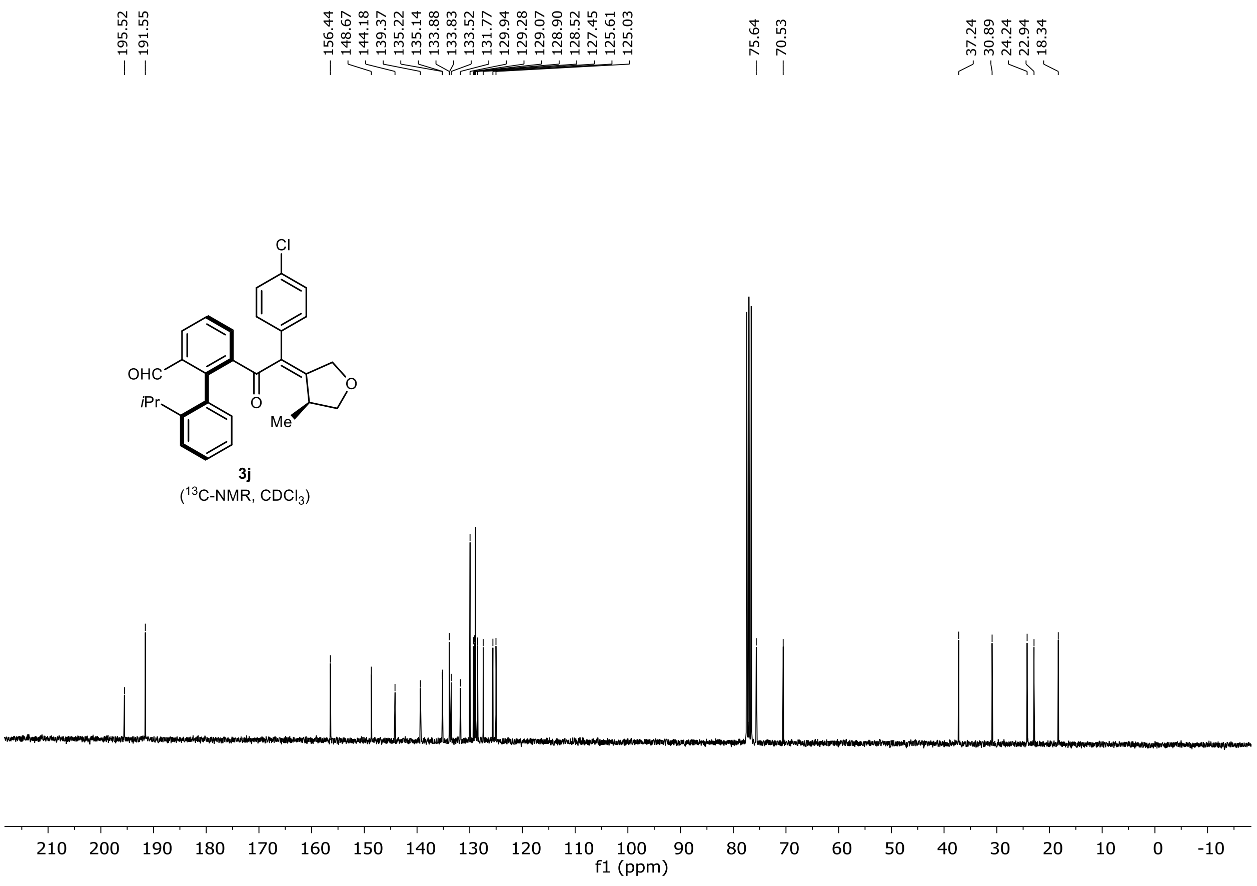
195.52 191.55 156.44 148.67 144.18 139.37 135.22 135.14 133.88 133.83 133.52 131.77 129.94 129.28 129.07 128.90 128.52 127.45 125.61 125.03 75.64 70.53 37.24 30.89 24.24 22.94 18.34
Cl
OHC
O
O
*i*Pr
Me
3j
(^{13}C-NMR, $CDCl_3$)
210 200 190 180 170 160 150 140 130 120 110 100 90 80 70 60 50 40 30 20 10 0 -10
f1 (ppm)

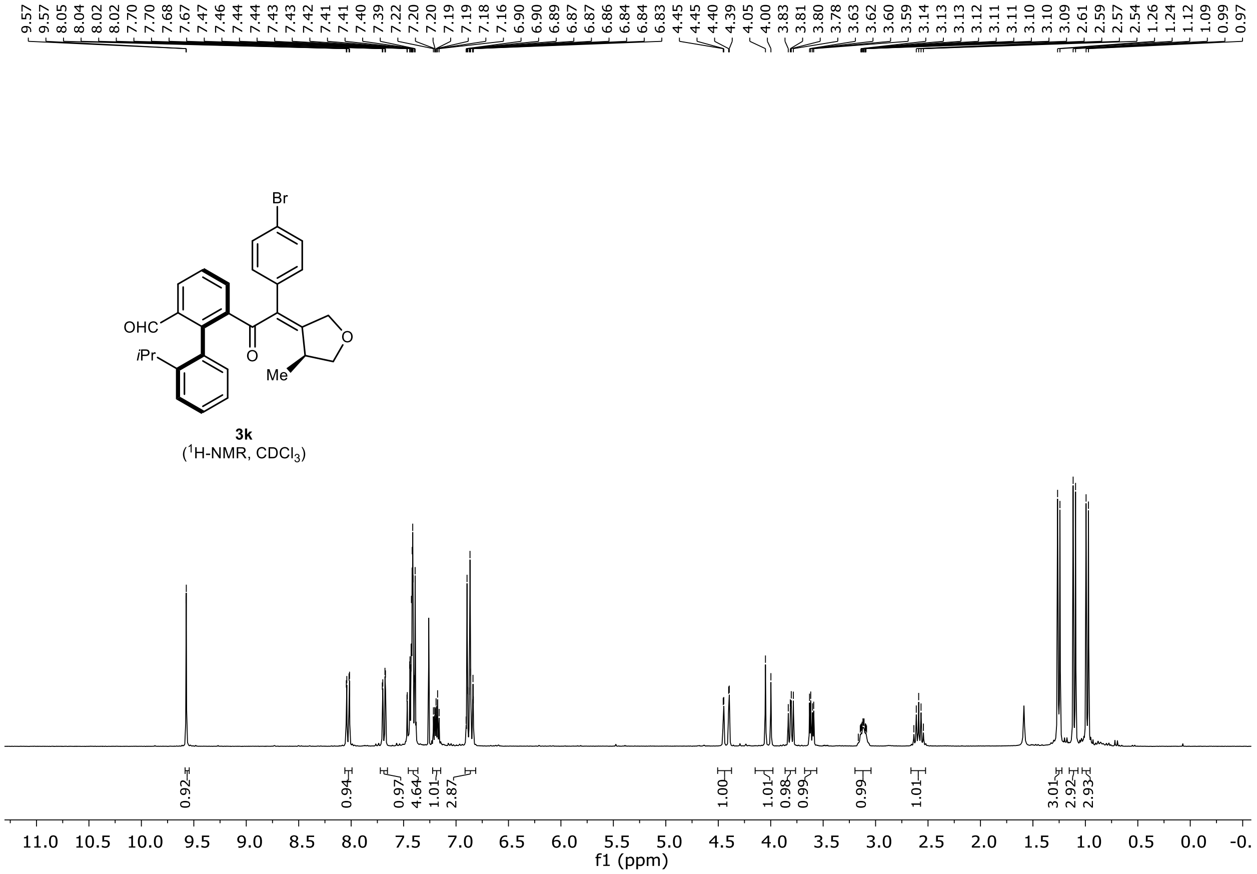
9.57
9.57
8.05
8.04
8.02
8.02
7.70
7.70
7.68
7.67
7.47
7.46
7.44
7.44
7.43
7.43
7.42
7.41
7.41
7.40
7.39
7.22
7.20
7.20
7.19
7.19
7.18
7.16
6.90
6.90
6.89
6.87
6.87
6.86
6.84
6.83
4.45
4.45
4.40
4.39
4.05
4.00
3.83
3.81
3.80
3.78
3.63
3.62
3.60
3.59
3.14
3.13
3.13
3.12
3.11
3.11
3.10
3.09
2.61
2.59
2.57
2.54
1.26
1.24
1.12
1.09
0.99
0.97
Br
OHC
O
O
iPr
Me
3k
(^{1}H-NMR, $CDCl_3$)
0.92
0.94
0.97
4.64
1.01
2.87
1.00
1.01
0.98
0.99
0.99
1.01
3.01
2.92
2.93
11.0 10.5 10.0 9.5 9.0 8.5 8.0 7.5 7.0 6.5 6.0 5.5 5.0 4.5 4.0 3.5 3.0 2.5 2.0 1.5 1.0 0.5 0.0 -0.
f1 (ppm)

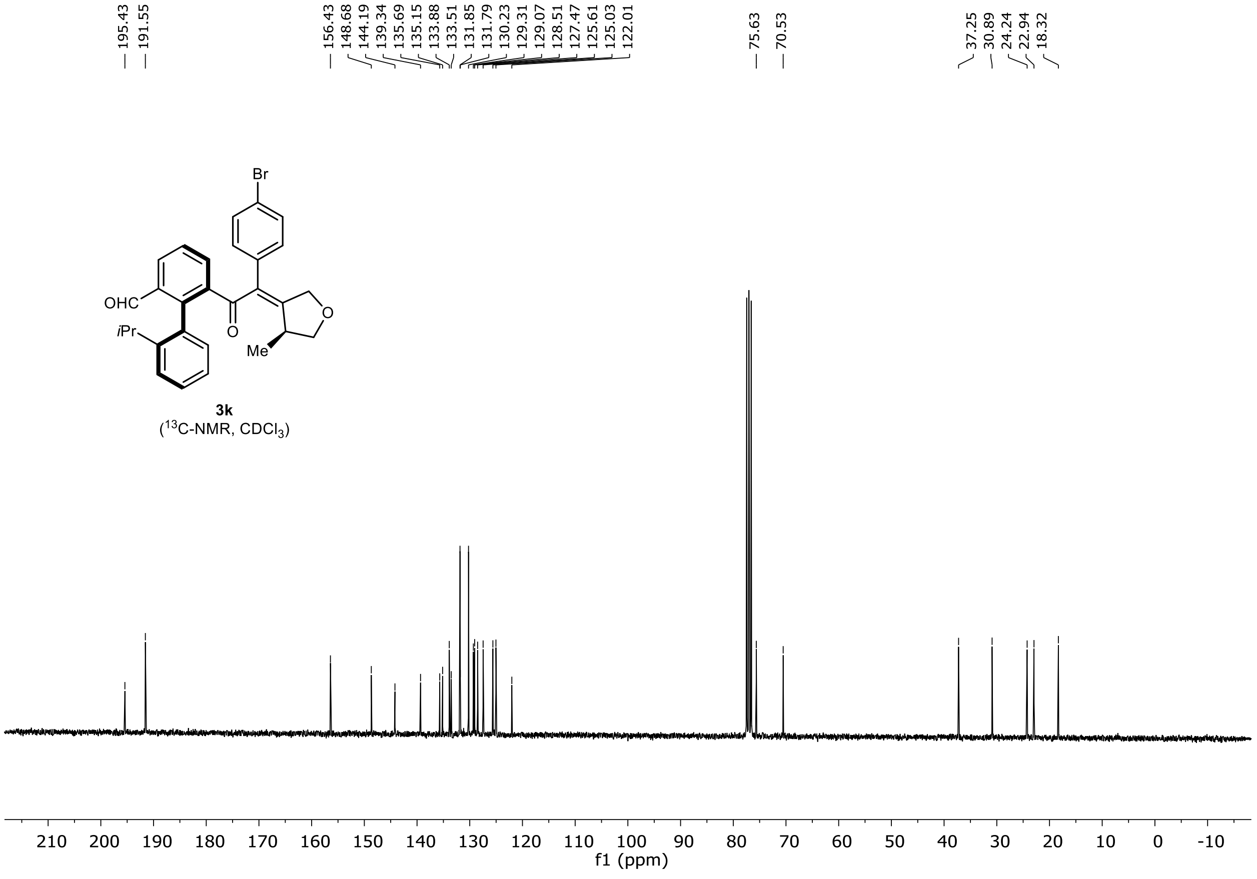
195.43
191.55
156.43
148.68
144.19
139.34
135.69
135.15
133.88
133.51
131.85
131.79
130.23
129.31
129.07
128.51
127.47
125.61
125.03
122.01
75.63
70.53
37.25
30.89
24.24
22.94
18.32
Br
OHC
O
O
iPr
Me
3k
(^{13}C-NMR, $CDCl_3$)
210 200 190 180 170 160 150 140 130 120 110 100 90 80 70 60 50 40 30 20 10 0 -10
f1 (ppm)

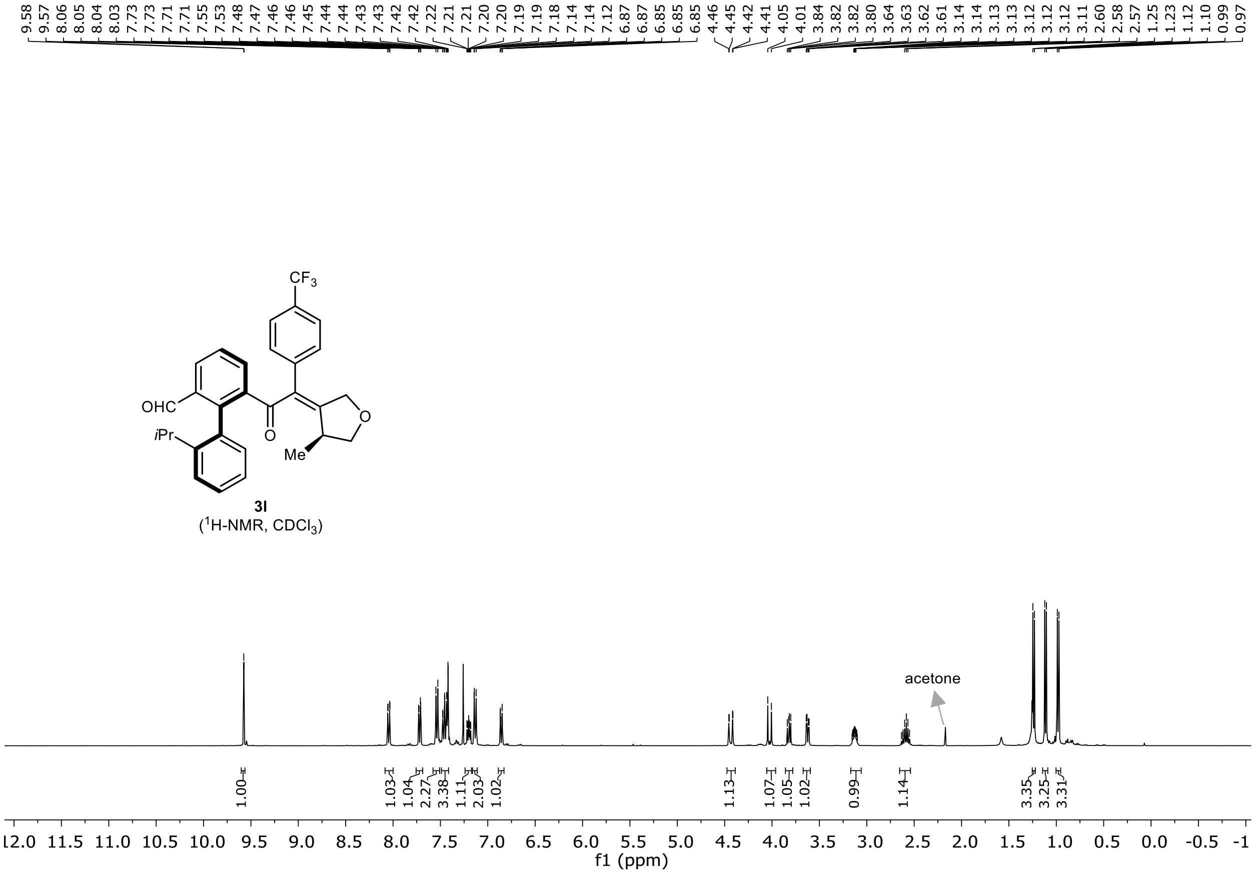
9.58 9.57 8.06 8.05 8.04 8.03 7.73 7.73 7.71 7.71 7.55 7.53 7.48 7.47 7.46 7.46 7.45 7.44 7.44 7.43 7.42 7.42 7.22 7.21 7.21 7.20 7.20 7.19 7.19 7.18 7.14 7.14 7.12 6.87 6.87 6.85 6.85 4.46 4.45 4.42 4.41 4.05 4.01 3.84 3.82 3.82 3.80 3.64 3.63 3.62 3.61 3.14 3.14 3.13 3.13 3.12 3.12 3.12 3.11 2.60 2.58 2.57 1.25 1.23 1.12 1.10 0.99 0.97
CF3
OHC
O
O
iPr
Me
3l
(1H-NMR, CDCl3)
acetone
1.00 1.03 1.04 2.27 3.38 1.11 2.03 1.02 1.13 1.07 1.05 1.02 0.99 1.14 3.35 3.25 3.31
12.0 11.5 11.0 10.5 10.0 9.5 9.0 8.5 8.0 7.5 7.0 6.5 6.0 5.5 5.0 4.5 4.0 3.5 3.0 2.5 2.0 1.5 1.0 0.5 0.0 -0.5 -1
f1 (ppm)

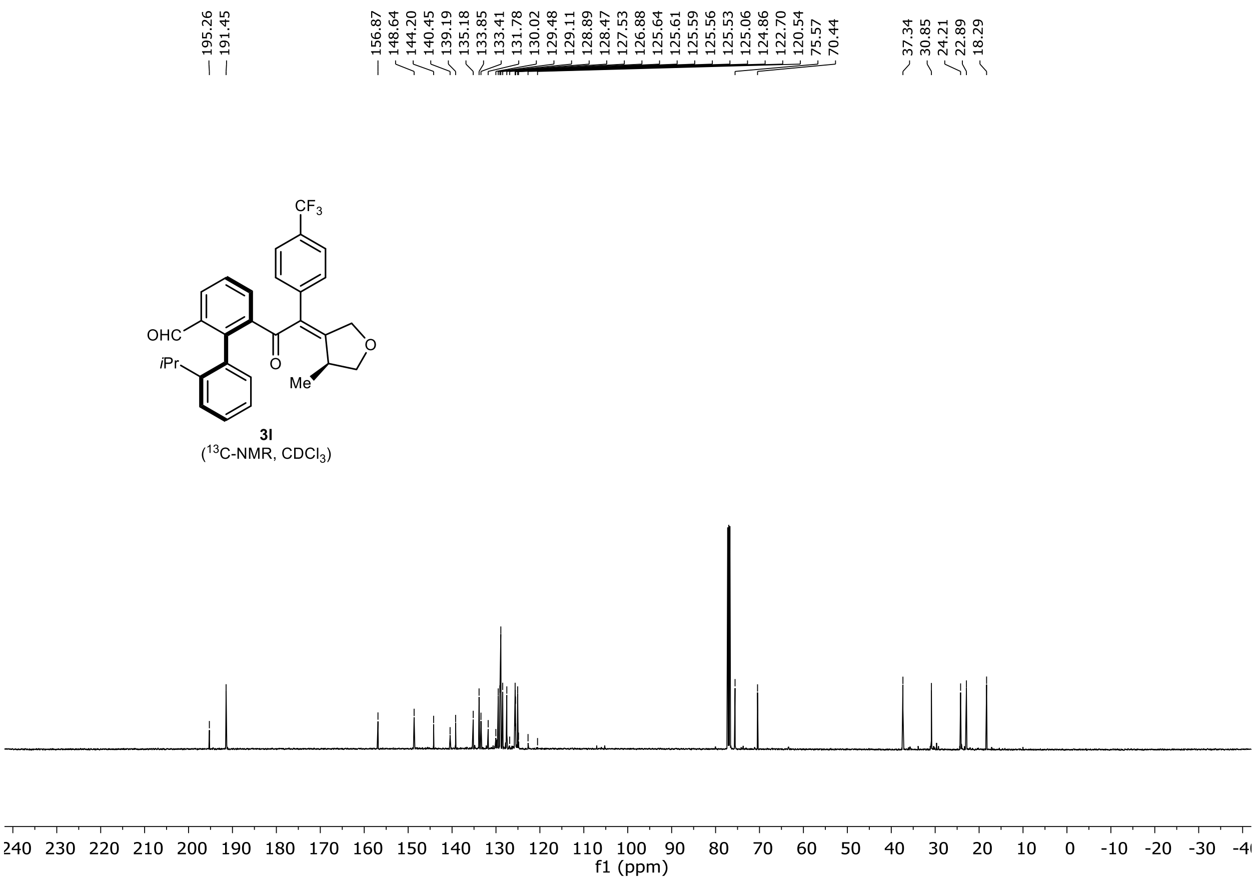
195.26 191.45 156.87 148.64 144.20 140.45 139.19 135.18 133.85 133.41 131.78 130.02 129.48 129.11 128.89 128.47 127.53 126.88 125.64 125.61 125.59 125.56 125.53 125.06 124.86 122.70 120.54 75.57 70.44 37.34 30.85 24.21 22.89 18.29
CF3
OHC
O
O
iPr
Me
3l
(13C-NMR, CDCl3)
240 230 220 210 200 190 180 170 160 150 140 130 120 110 100 90 80 70 60 50 40 30 20 10 0 -10 -20 -30 -40
f1 (ppm)

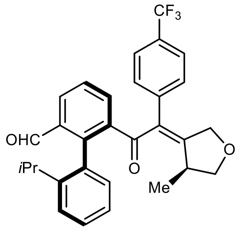

3l
(^{19}F-NMR, $CDCl_3$)

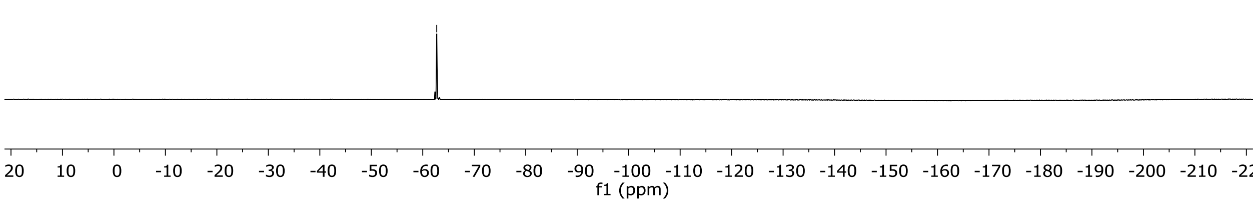

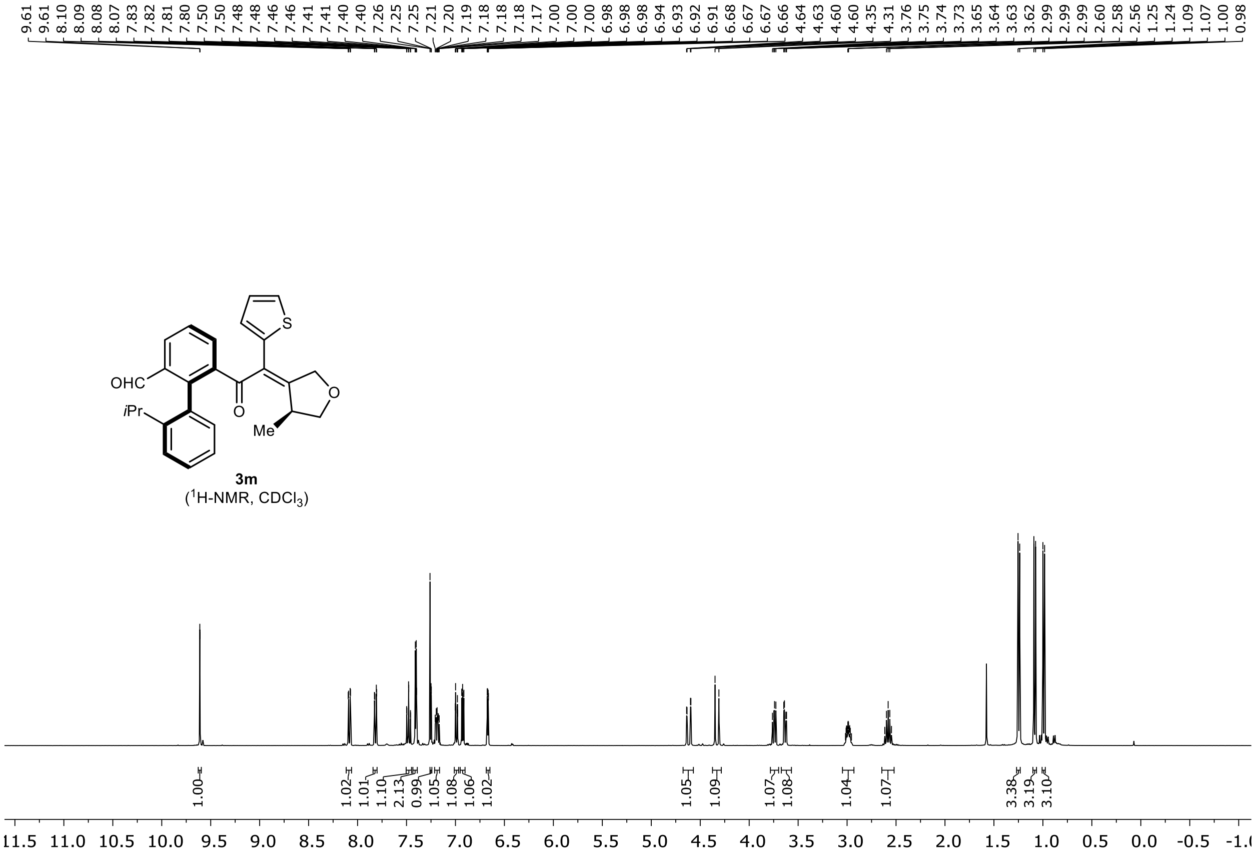
9.61 9.61 8.10 8.09 8.08 8.07 7.83 7.82 7.81 7.80 7.50 7.50 7.48 7.48 7.46 7.46 7.41 7.41 7.40 7.40 7.26 7.25 7.25 7.21 7.20 7.19 7.18 7.18 7.18 7.17 7.00 7.00 7.00 6.98 6.98 6.98 6.94 6.93 6.92 6.91 6.68 6.67 6.67 6.66 4.64 4.63 4.60 4.60 4.35 4.31 3.76 3.75 3.74 3.73 3.65 3.64 3.63 3.62 2.99 2.99 2.99 2.60 2.58 2.56 1.25 1.24 1.09 1.07 1.00 0.98
OHC
iPr
S
O
Me
3m
(1H-NMR, CDCl3)
1.00 1.02 1.01 1.10 2.13 0.99 1.05 1.08 1.06 1.02 1.05 1.09 1.07 1.08 1.04 1.07 3.38 3.19 3.10
f1 (ppm)

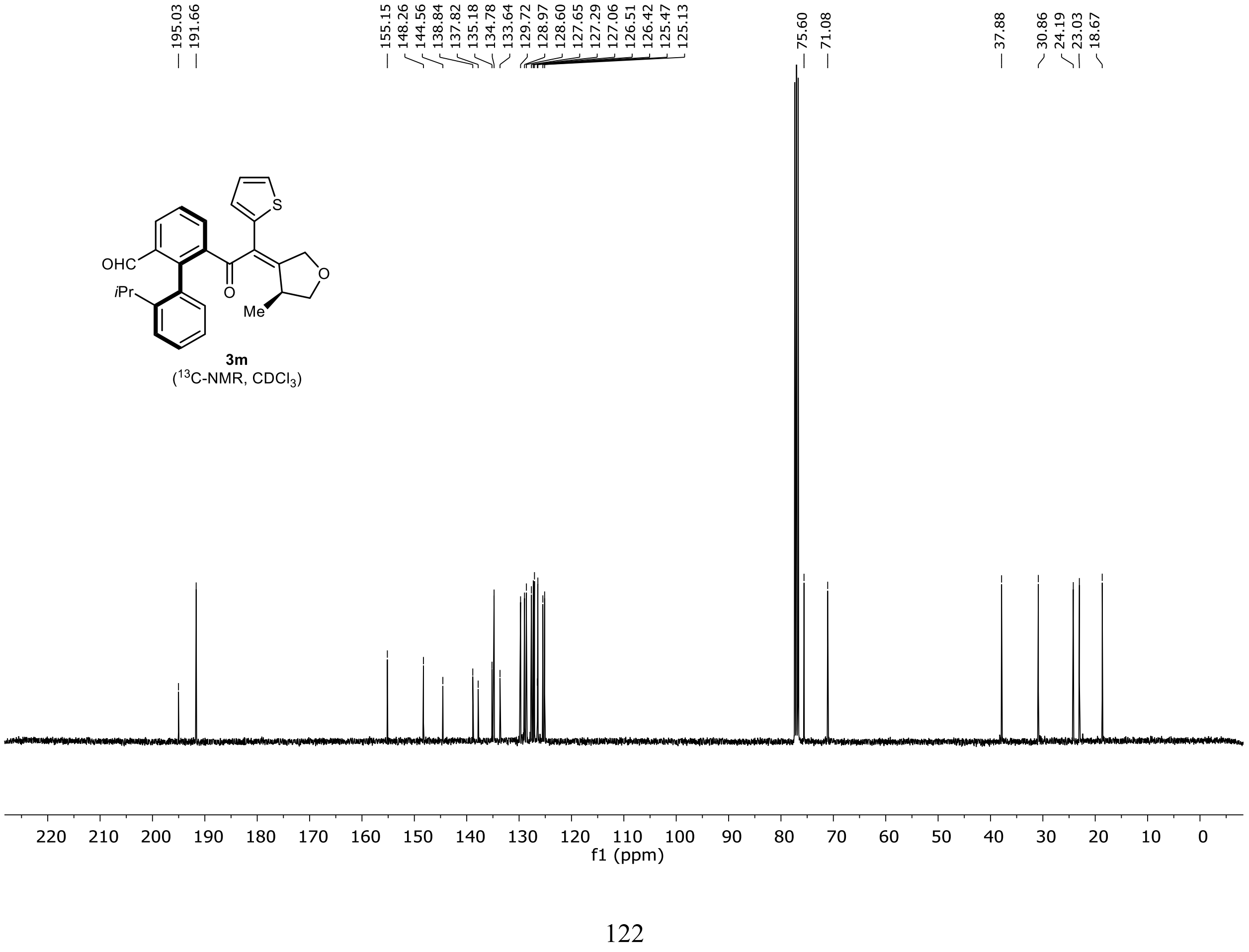
195.03 191.66 155.15 148.26 144.56 138.84 137.82 135.18 134.78 133.64 129.72 128.97 128.60 127.65 127.29 127.06 126.51 126.42 125.47 125.13 75.60 71.08 37.88 30.86 24.19 23.03 18.67
OHC
iPr
S
O
Me
3m
(13C-NMR, CDCl3)
f1 (ppm)

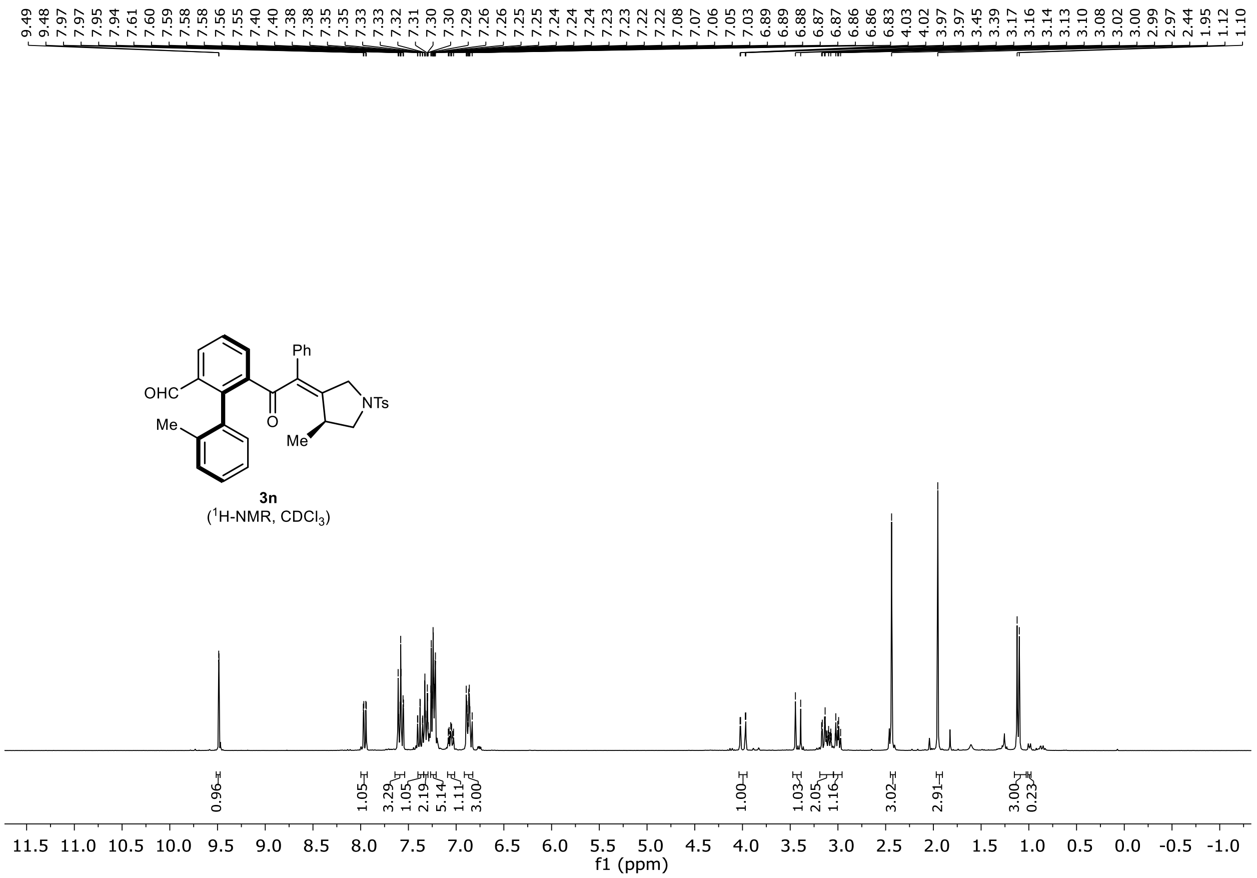
9.49 9.48 7.97 7.97 7.95 7.94 7.61 7.60 7.59 7.58 7.58 7.56 7.55 7.40 7.40 7.38 7.38 7.35 7.35 7.33 7.33 7.32 7.31 7.30 7.30 7.29 7.26 7.26 7.25 7.25 7.24 7.24 7.24 7.23 7.23 7.22 7.22 7.08 7.07 7.06 7.05 7.03 6.89 6.89 6.88 6.87 6.87 6.86 6.86 6.83 4.03 4.02 3.97 3.97 3.45 3.39 3.17 3.16 3.14 3.13 3.10 3.08 3.02 3.00 2.99 2.97 2.44 1.95 1.12 1.10
Ph
OHC
O
NTs
Me
Me
3n
(^{1}H-NMR, CDCl$_3$)
0.96 1.05 3.29 1.05 2.19 5.14 1.11 3.00 1.00 1.03 2.05 1.16 3.02 2.91 3.00 0.23
11.5 11.0 10.5 10.0 9.5 9.0 8.5 8.0 7.5 7.0 6.5 6.0 5.5 5.0 4.5 4.0 3.5 3.0 2.5 2.0 1.5 1.0 0.5 0.0 -0.5 -1.0
f1 (ppm)

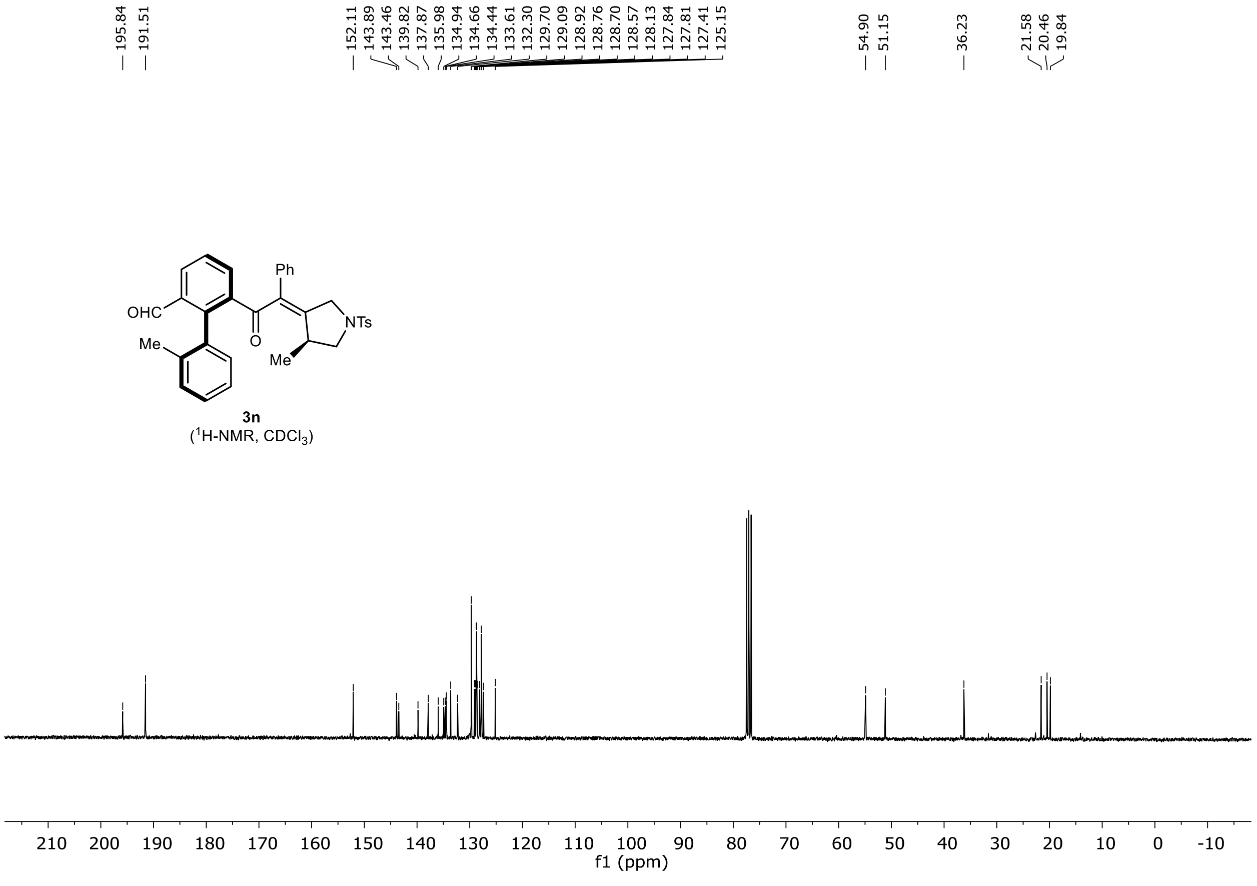
195.84 191.51 152.11 143.89 143.46 139.82 137.87 135.98 134.94 134.66 134.44 133.61 132.30 129.70 129.09 128.92 128.76 128.70 128.57 128.13 127.84 127.81 127.41 125.15 54.90 51.15 36.23 21.58 20.46 19.84
Ph
OHC
O
NTs
Me
Me
3n
(^{1}H-NMR, CDCl$_3$)
210 200 190 180 170 160 150 140 130 120 110 100 90 80 70 60 50 40 30 20 10 0 -10
f1 (ppm)

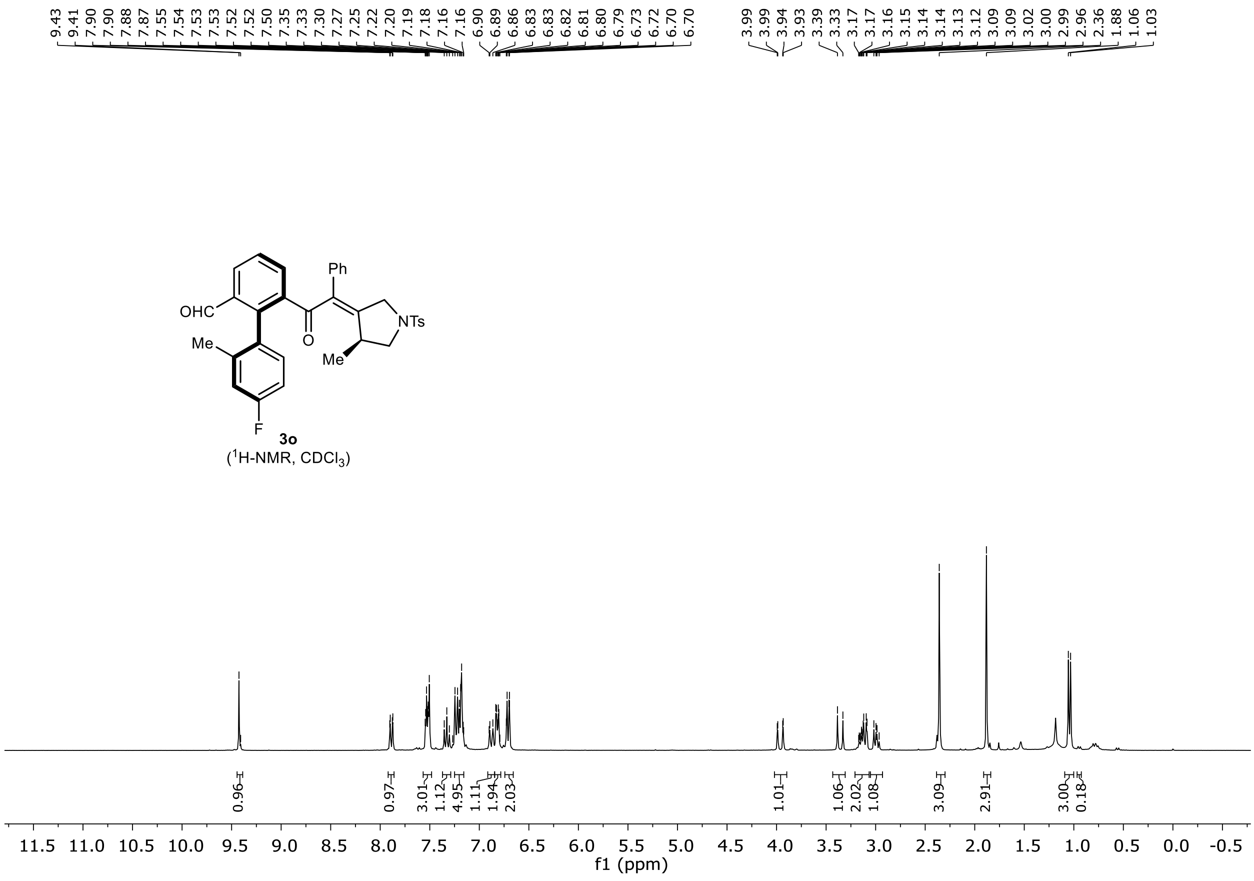
9.43 9.41 7.90 7.90 7.88 7.87 7.55 7.54 7.53 7.53 7.52 7.52 7.50 7.35 7.33 7.30 7.27 7.25 7.22 7.20 7.19 7.18 7.16 7.16 6.90 6.89 6.86 6.83 6.83 6.82 6.81 6.80 6.79 6.73 6.72 6.70 6.70
3.99 3.99 3.94 3.93 3.39 3.33 3.17 3.17 3.16 3.15 3.14 3.14 3.13 3.12 3.09 3.09 3.02 3.00 2.99 2.96 2.36 1.88 1.06 1.03
Ph
OHC
O
NTs
Me
Me
F
3o
(1H-NMR, CDCl3)
0.96 0.97 3.01 1.12 4.95 1.11 1.94 2.03 1.01 1.06 2.02 1.08 3.09 2.91 3.00 0.18
11.5 11.0 10.5 10.0 9.5 9.0 8.5 8.0 7.5 7.0 6.5 6.0 5.5 5.0 4.5 4.0 3.5 3.0 2.5 2.0 1.5 1.0 0.5 0.0 -0.5
f1 (ppm)

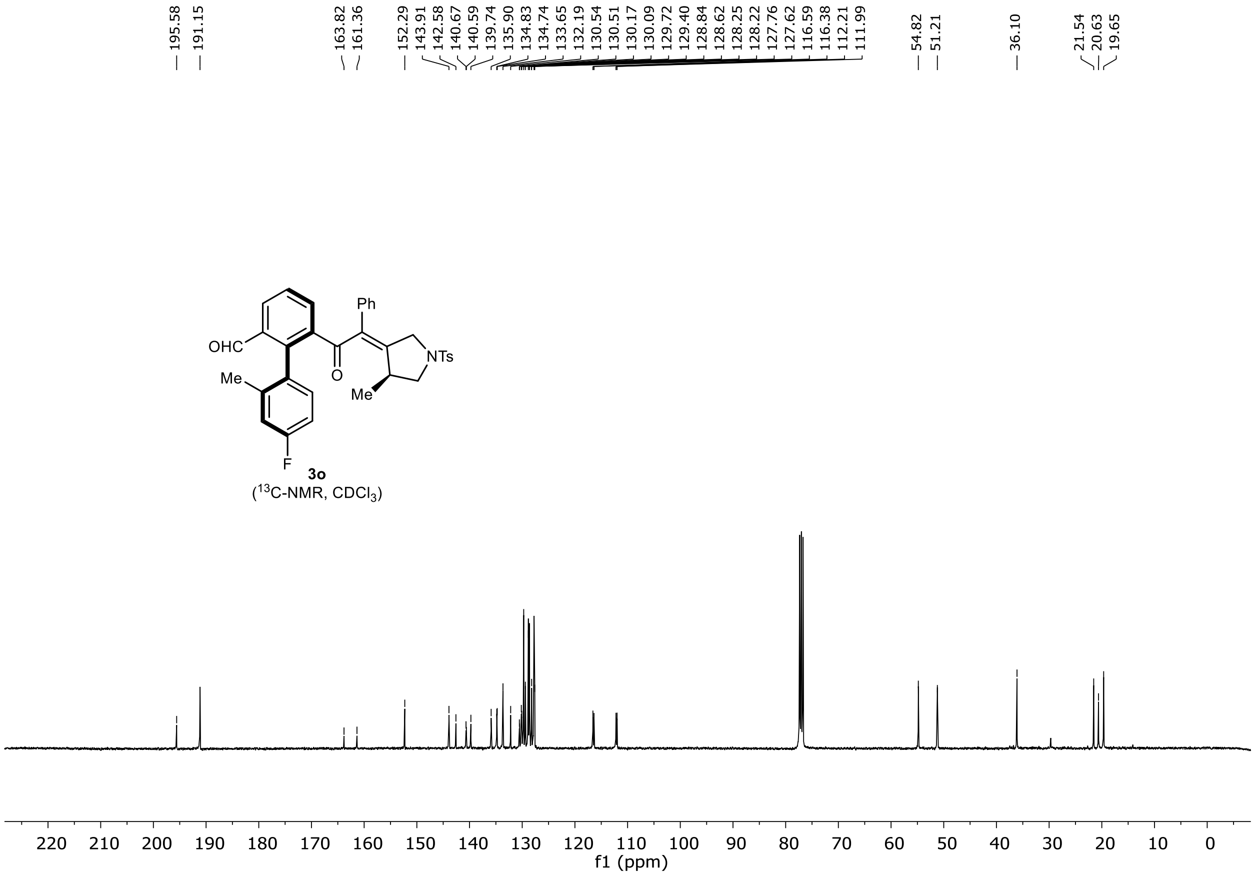
195.58 191.15 163.82 161.36 152.29 143.91 142.58 140.67 140.59 139.74 135.90 134.83 134.74 133.65 132.19 130.54 130.51 130.17 130.09 129.72 129.40 128.84 128.62 128.25 128.22 127.76 127.62 116.59 116.38 112.21 111.99 54.82 51.21 36.10 21.54 20.63 19.65
Ph
OHC
O
NTs
Me
Me
F
3o
(13C-NMR, CDCl3)
220 210 200 190 180 170 160 150 140 130 120 110 100 90 80 70 60 50 40 30 20 10 0
f1 (ppm)

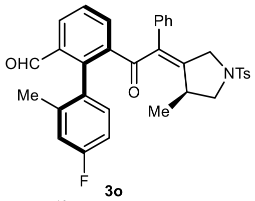

3o
(^{19}F-NMR, $CDCl_3$)

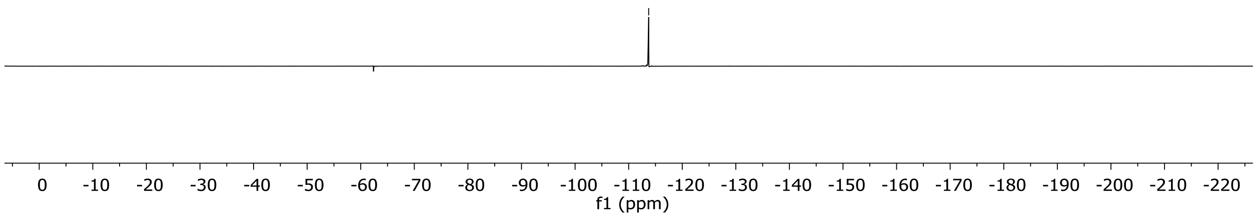

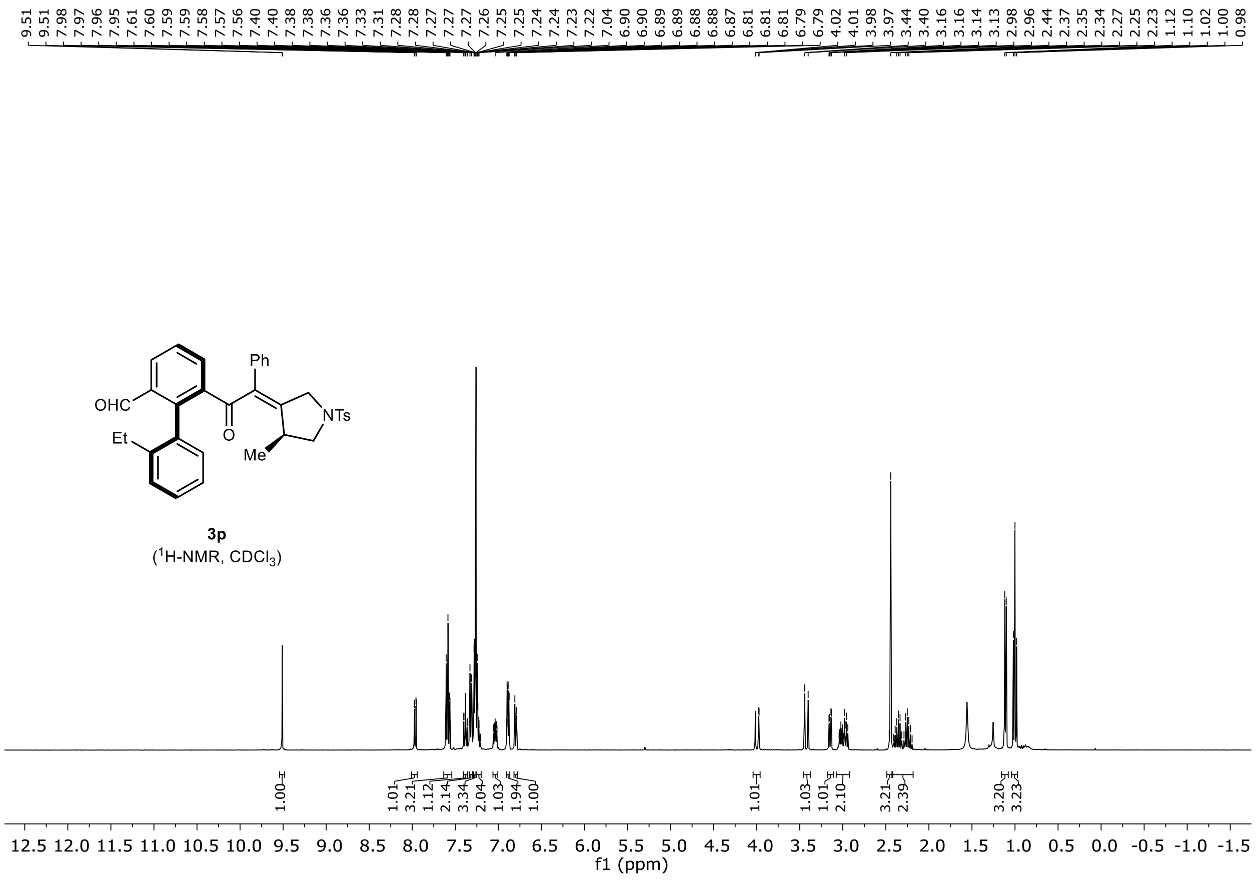
9.51 9.51 7.98 7.97 7.96 7.95 7.61 7.60 7.59 7.59 7.58 7.57 7.56 7.40 7.40 7.38 7.38 7.36 7.36 7.33 7.31 7.28 7.28 7.27 7.27 7.27 7.26 7.25 7.25 7.24 7.24 7.23 7.22 7.04 6.90 6.89 6.88 6.88 6.87 6.81 6.81 6.79 6.79 4.02 4.01 3.98 3.97 3.44 3.40 3.16 3.16 3.14 3.13 2.98 2.96 2.44 2.37 2.35 2.34 2.27 2.25 2.23 1.12 1.10 1.02 1.00 0.98
Ph
OHC
O
NTs
Et
Me
3p
(1H-NMR, CDCl3)
1.00 1.01 3.21 1.12 2.14 3.34 2.04 1.03 1.94 1.00 1.01 1.03 1.01 2.10 3.21 2.39 3.20 3.23
12.5 12.0 11.5 11.0 10.5 10.0 9.5 9.0 8.5 8.0 7.5 7.0 6.5 6.0 5.5 5.0 4.5 4.0 3.5 3.0 2.5 2.0 1.5 1.0 0.5 0.0 -0.5 -1.0 -1.5
f1 (ppm)

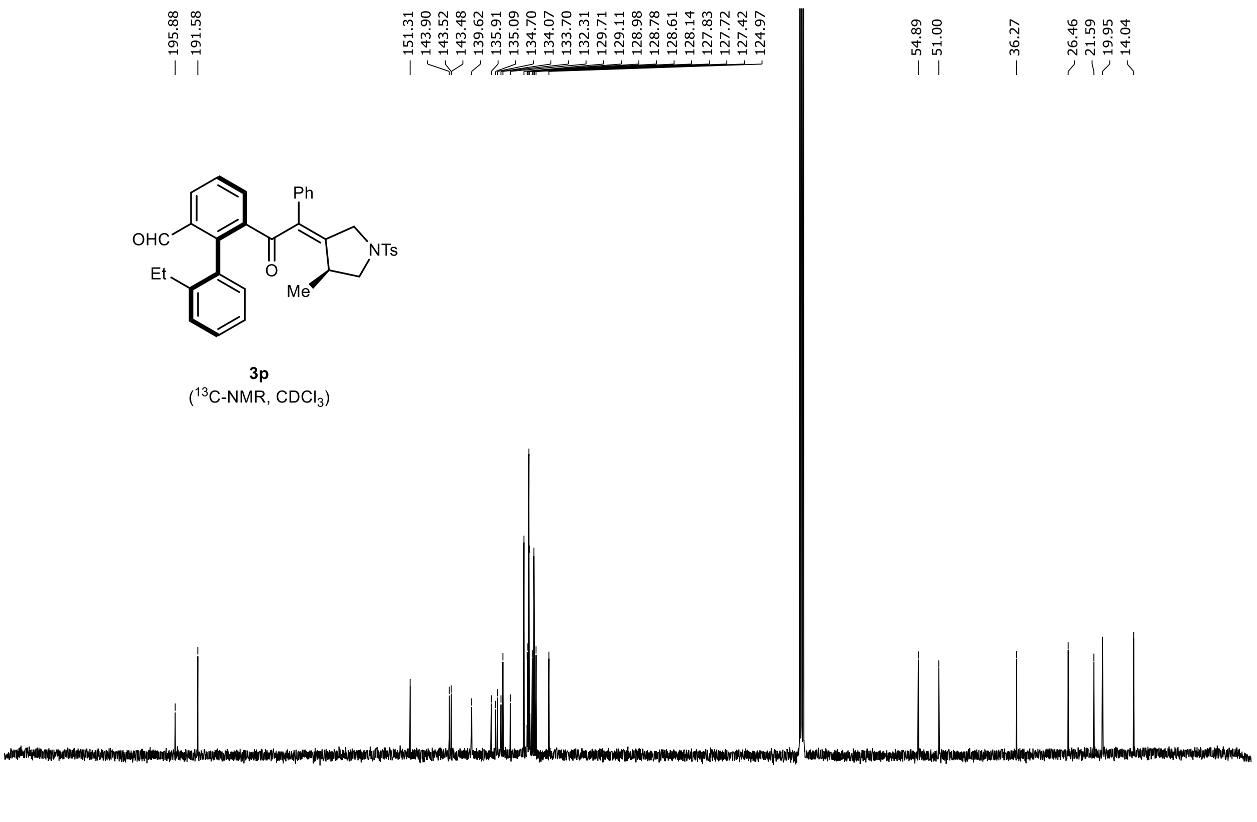
195.88 191.58 151.31 143.90 143.52 143.48 139.62 135.91 135.09 134.70 134.07 133.70 132.31 129.71 129.11 128.98 128.78 128.61 128.14 127.83 127.72 127.42 124.97 54.89 51.00 36.27 26.46 21.59 19.95 14.04
Ph
OHC
O
NTs
Et
Me
3p
(13C-NMR, CDCl3)
220 210 200 190 180 170 160 150 140 130 120 110 100 90 80 70 60 50 40 30 20 10 0
f1 (ppm)

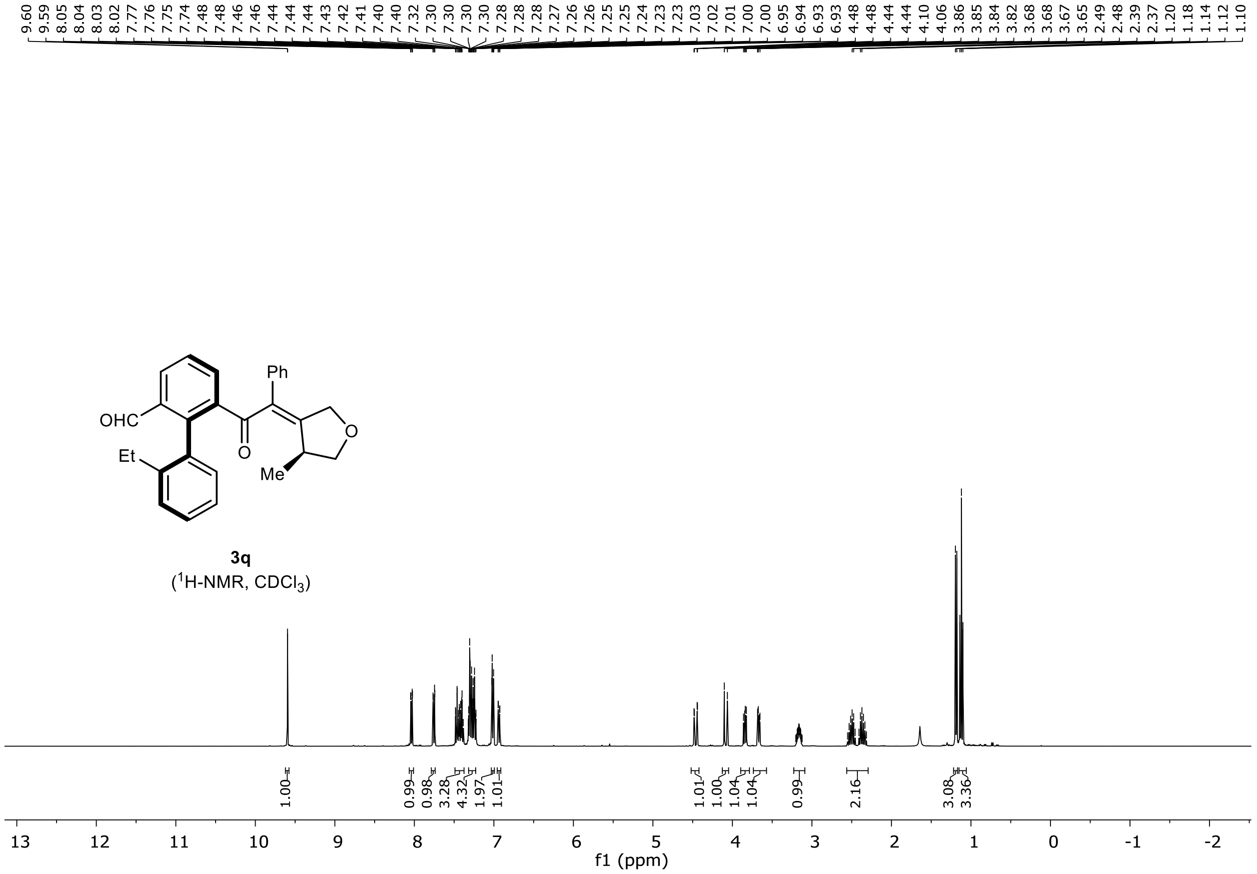

3q
(^{1}H-NMR, $CDCl_3$)

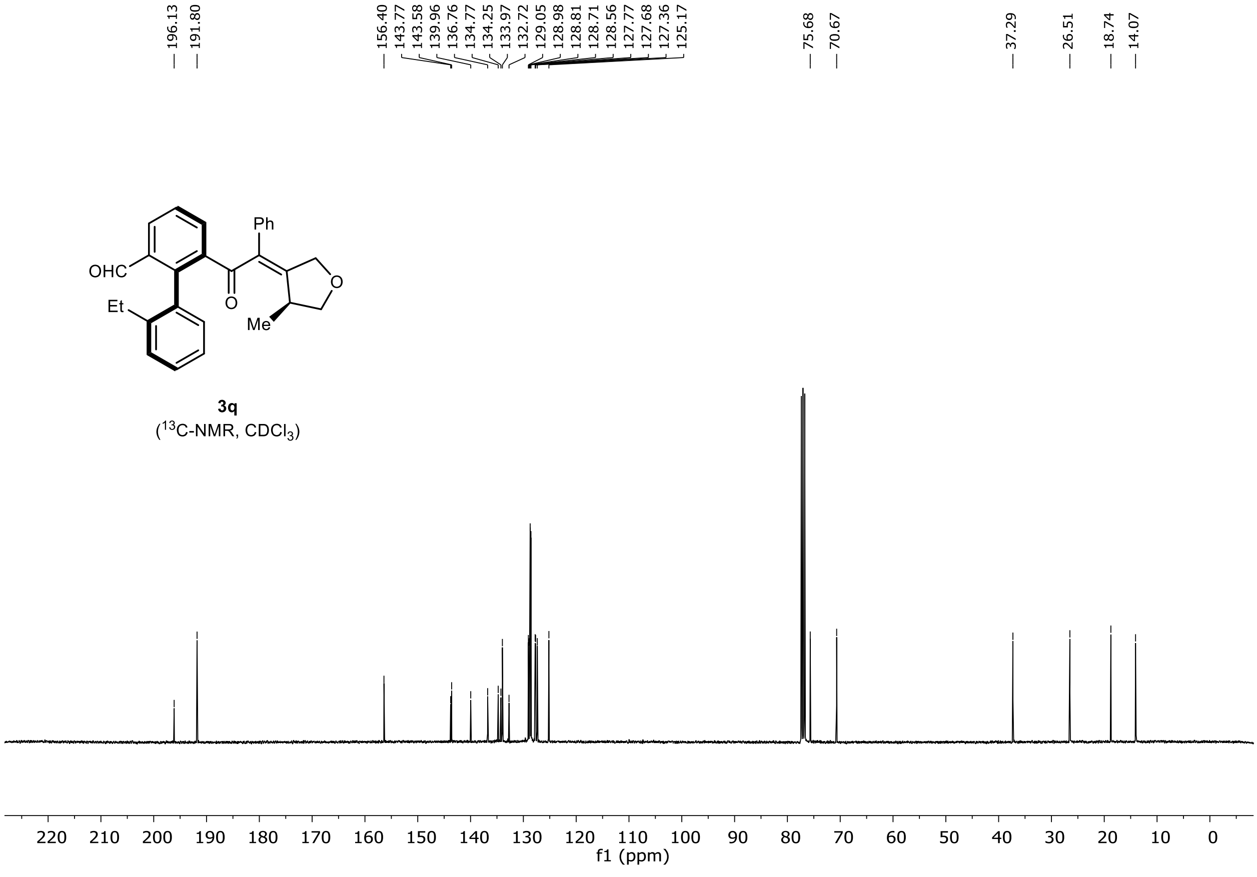

3q
(^{13}C-NMR, $CDCl_3$)

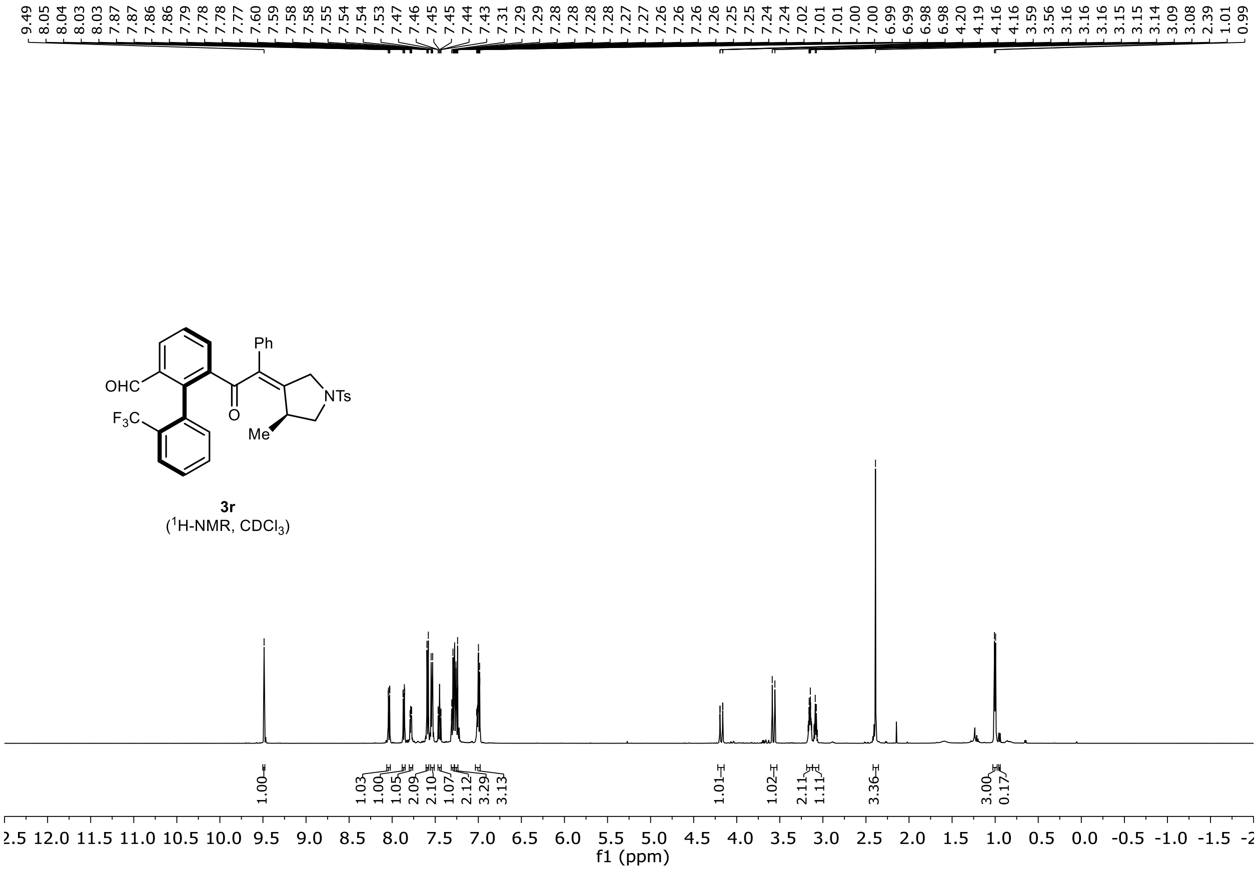
9.49 8.05 8.04 8.03 7.87 7.87 7.86 7.79 7.78 7.78 7.77 7.60 7.59 7.58 7.58 7.55 7.54 7.54 7.53 7.47 7.46 7.45 7.45 7.44 7.43 7.31 7.29 7.29 7.28 7.28 7.28 7.28 7.27 7.27 7.26 7.26 7.26 7.26 7.25 7.25 7.24 7.24 7.02 7.01 7.01 7.00 7.00 6.99 6.99 6.98 6.98 4.20 4.19 4.16 4.16 3.59 3.56 3.16 3.16 3.16 3.15 3.15 3.14 3.09 3.08 2.39 1.01 0.99
Ph
OHC
NTs
O
F3C
Me
3r
(1H-NMR, CDCl3)
1.00
1.03 1.00 1.05 2.09 2.10 1.07 2.12 3.29 3.13
1.01
1.02
2.11 1.11
3.36
3.00 0.17
f1 (ppm)

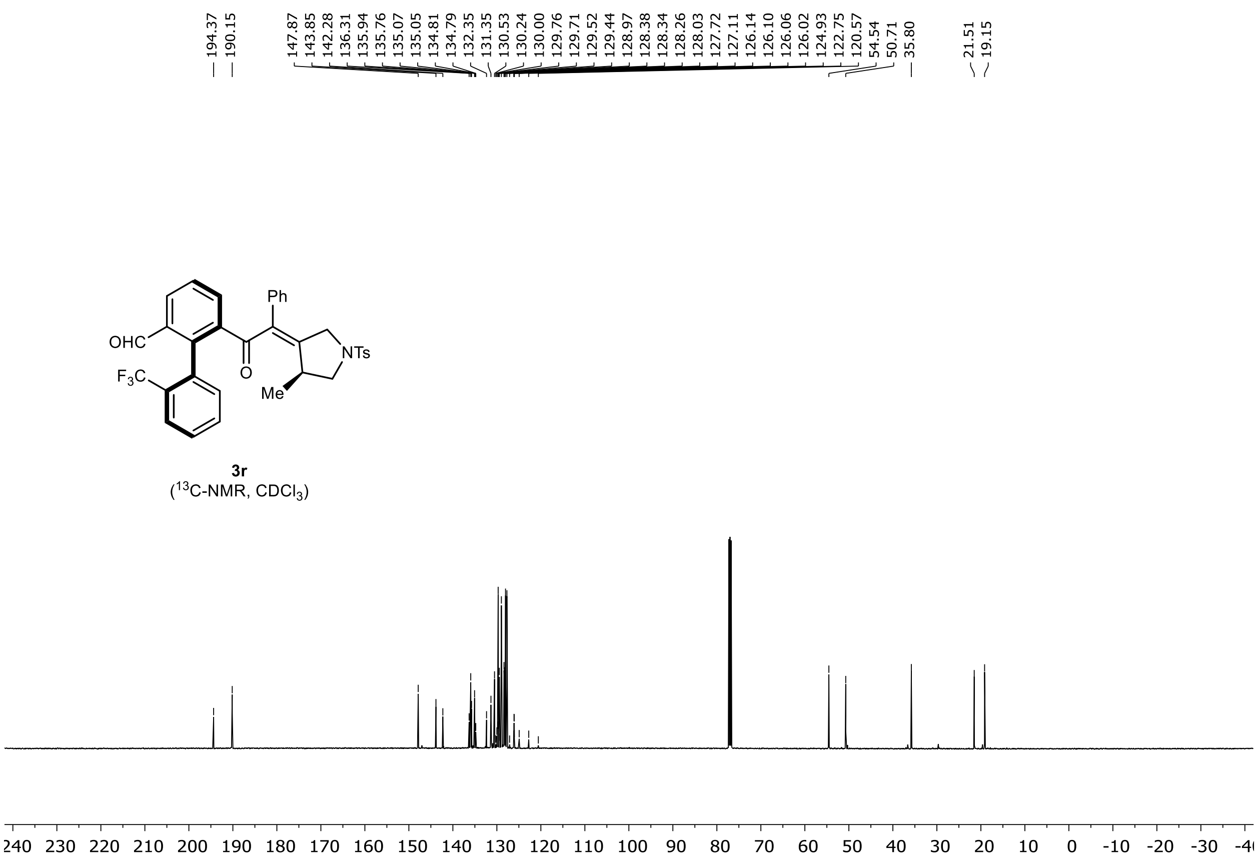
194.37 190.15
147.87 143.85 142.28 136.31 135.94 135.76 135.07 135.05 134.81 134.79 132.35 131.35 130.53 130.24 130.00 129.76 129.71 129.52 129.44 128.97 128.38 128.34 128.26 128.03 127.72 127.11 126.14 126.10 126.06 126.02 124.93 122.75 120.57 54.54 50.71 35.80
21.51 19.15
Ph
OHC
NTs
O
F3C
Me
3r
(13C-NMR, CDCl3)
f1 (ppm)

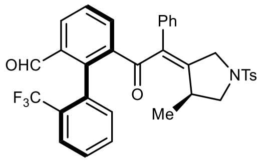

3r
(^{19}F-NMR, $CDCl_3$)

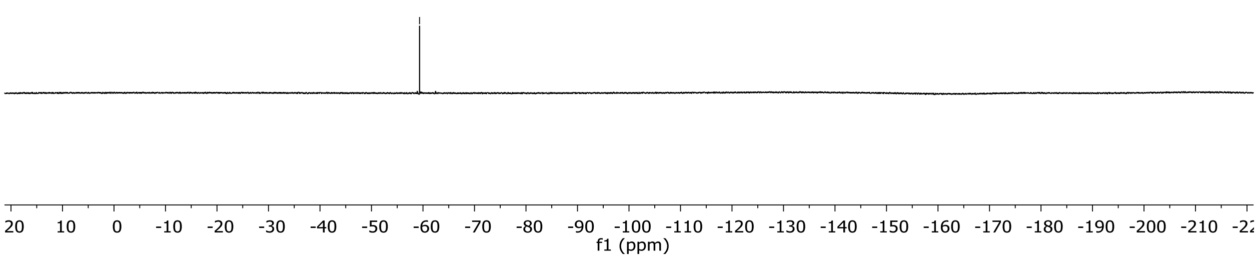

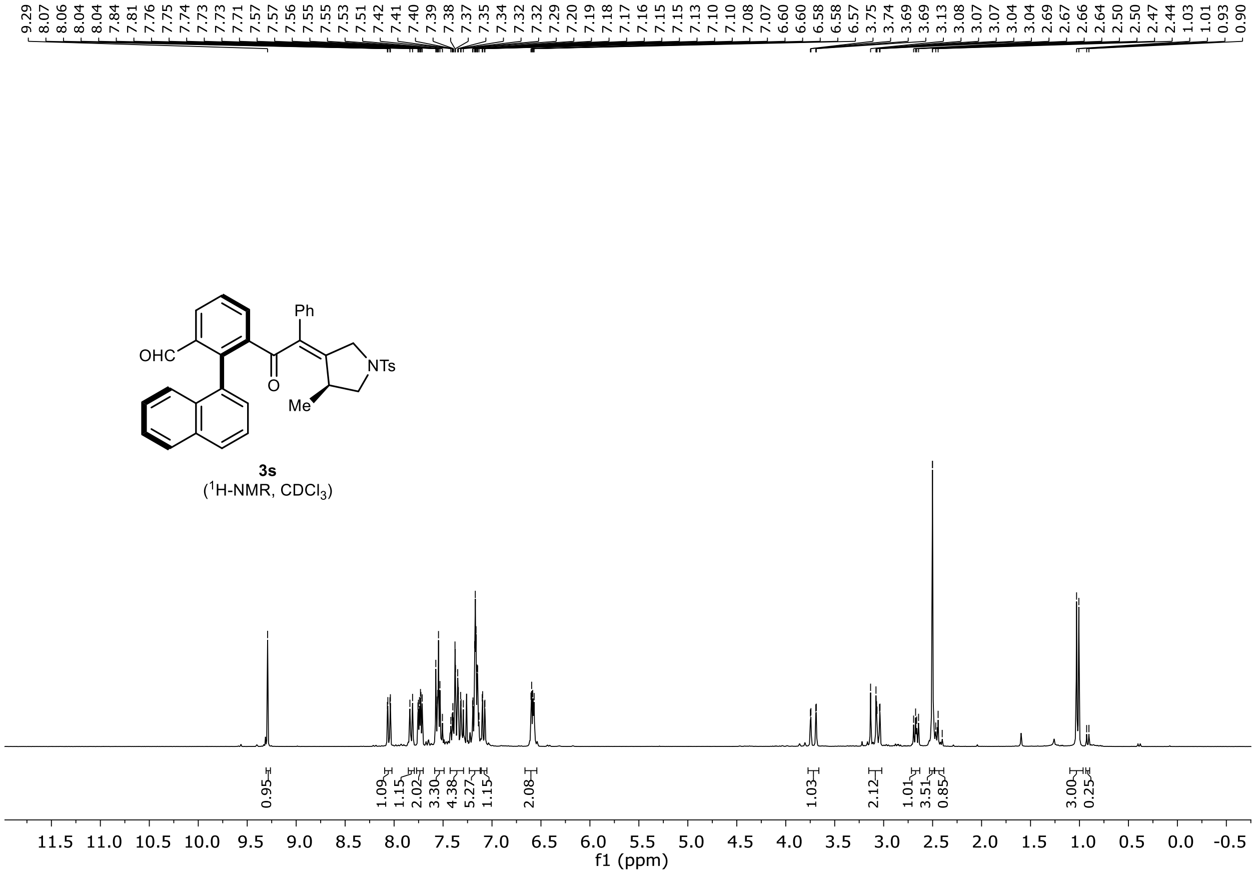
9.29 8.07 8.06 8.04 8.04 7.84 7.81 7.76 7.75 7.74 7.73 7.73 7.71 7.57 7.57 7.56 7.55 7.55 7.53 7.51 7.42 7.41 7.40 7.39 7.38 7.37 7.35 7.34 7.32 7.32 7.29 7.20 7.19 7.18 7.17 7.16 7.15 7.15 7.13 7.10 7.10 7.08 7.07 6.60 6.60 6.58 6.57 3.75 3.74 3.69 3.69 3.13 3.08 3.07 3.07 3.04 3.04 2.69 2.67 2.66 2.64 2.50 2.47 2.44 1.03 1.01 0.93 0.90
Ph
OHC
O
NTs
Me
3s
(^{1}H-NMR, $CDCl_3$)
0.95 1.09 1.15 2.02 3.30 4.38 5.27 1.15 2.08 1.03 2.12 1.01 3.51 0.85 3.00 0.25
11.5 11.0 10.5 10.0 9.5 9.0 8.5 8.0 7.5 7.0 6.5 6.0 5.5 5.0 4.5 4.0 3.5 3.0 2.5 2.0 1.5 1.0 0.5 0.0 -0.5
f1 (ppm)

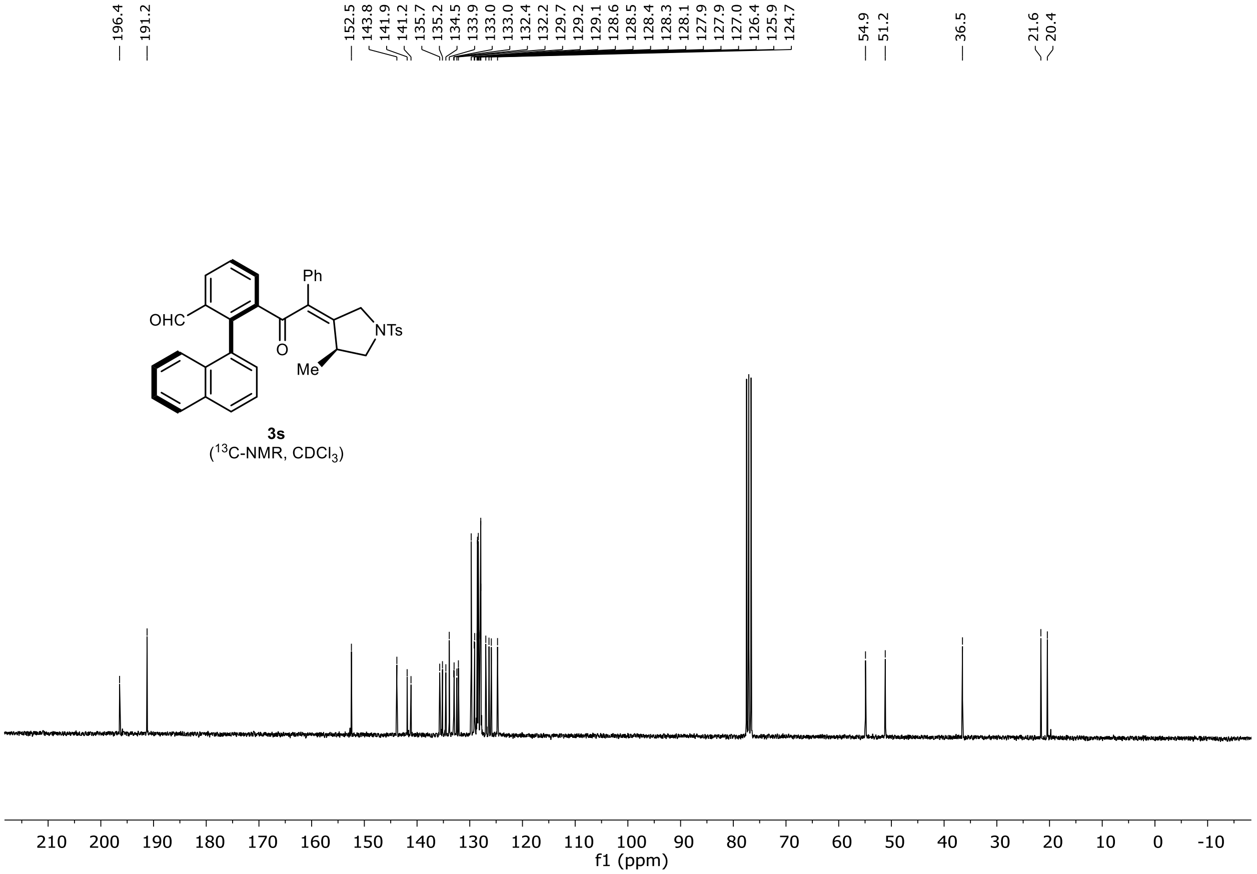
196.4 191.2 152.5 143.8 141.9 141.2 135.7 135.2 134.5 133.9 133.0 133.0 132.4 132.2 129.7 129.2 129.1 128.6 128.5 128.4 128.3 128.1 127.9 127.9 127.0 126.4 125.9 124.7 54.9 51.2 36.5 21.6 20.4
Ph
OHC
O
NTs
Me
3s
(^{13}C-NMR, $CDCl_3$)
210 200 190 180 170 160 150 140 130 120 110 100 90 80 70 60 50 40 30 20 10 0 -10
f1 (ppm)

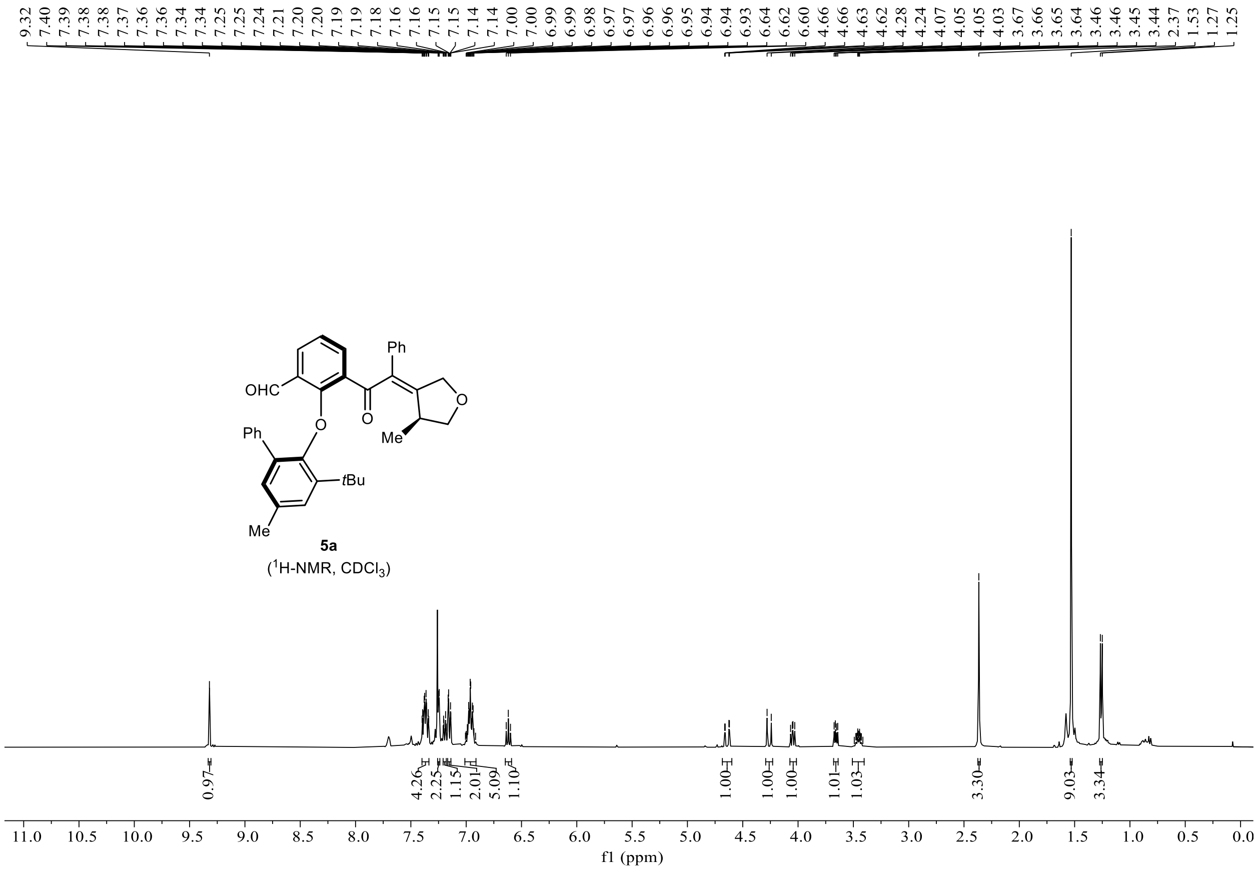
9.32 7.40 7.39 7.38 7.37 7.36 7.36 7.34 7.34 7.25 7.25 7.24 7.21 7.20 7.20 7.19 7.19 7.18 7.16 7.16 7.15 7.15 7.14 7.14 7.00 7.00 6.99 6.99 6.98 6.97 6.97 6.96 6.96 6.95 6.94 6.94 6.93 6.64 6.62 6.60 4.66 4.66 4.63 4.62 4.28 4.24 4.07 4.05 4.05 4.03 3.67 3.66 3.65 3.64 3.46 3.46 3.45 3.44 2.37 1.53 1.27 1.25
OHC
Ph
O
Me
tBu
5a
(^{1}H-NMR, $CDCl_3$)
0.97 4.26 2.25 1.15 2.01 5.09 1.10 1.00 1.00 1.00 1.01 1.03 3.30 9.03 3.34
11.0 10.5 10.0 9.5 9.0 8.5 8.0 7.5 7.0 6.5 6.0 5.5 5.0 4.5 4.0 3.5 3.0 2.5 2.0 1.5 1.0 0.5 0.0
f1 (ppm)

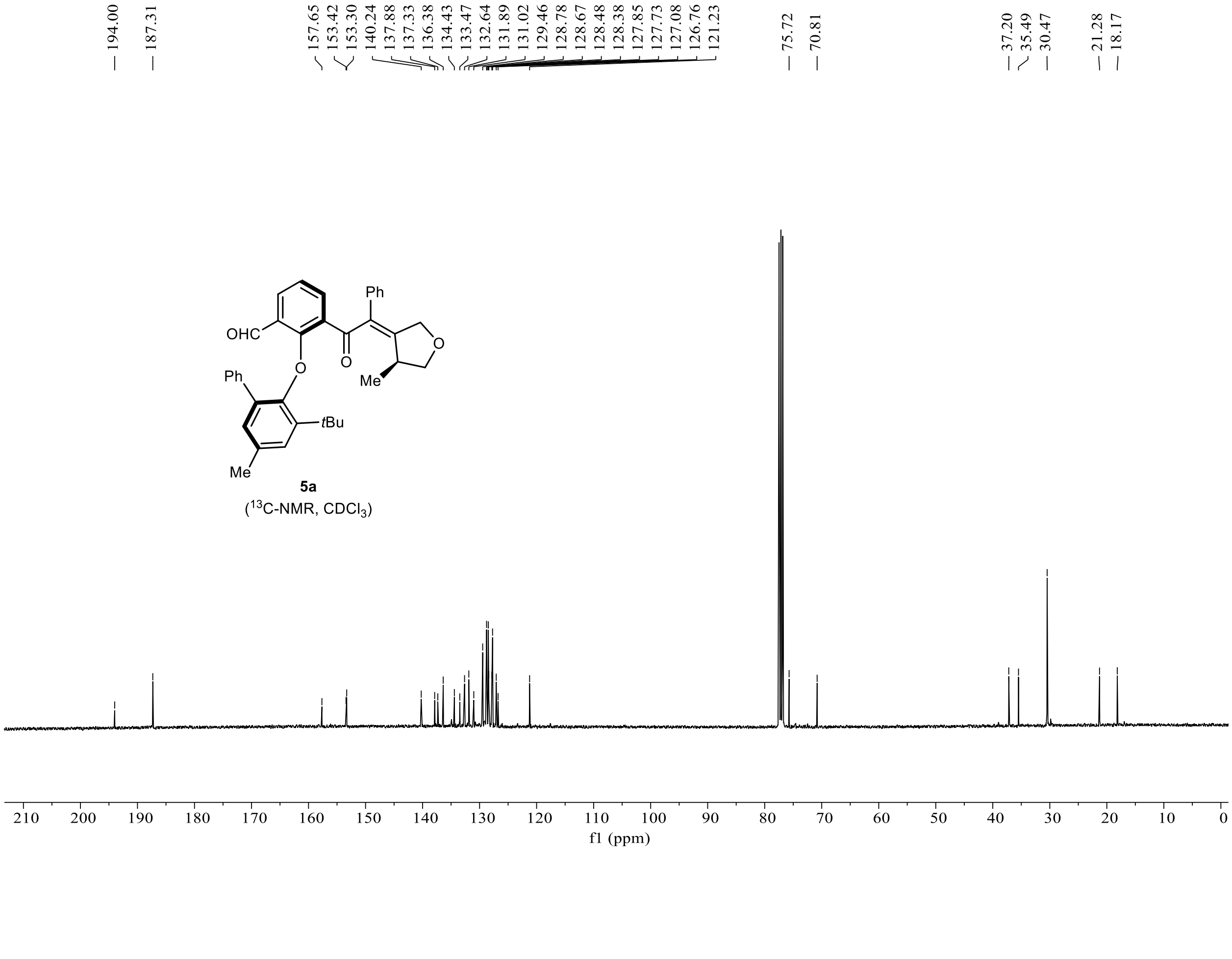
194.00 187.31 157.65 153.42 153.30 140.24 137.88 137.33 136.38 134.43 133.47 132.64 131.89 131.02 129.46 128.78 128.67 128.48 128.38 127.85 127.73 127.08 126.76 121.23 75.72 70.81 37.20 35.49 30.47 21.28 18.17
OHC
Ph
O
Me
tBu
5a
(^{13}C-NMR, $CDCl_3$)
210 200 190 180 170 160 150 140 130 120 110 100 90 80 70 60 50 40 30 20 10 0
f1 (ppm)

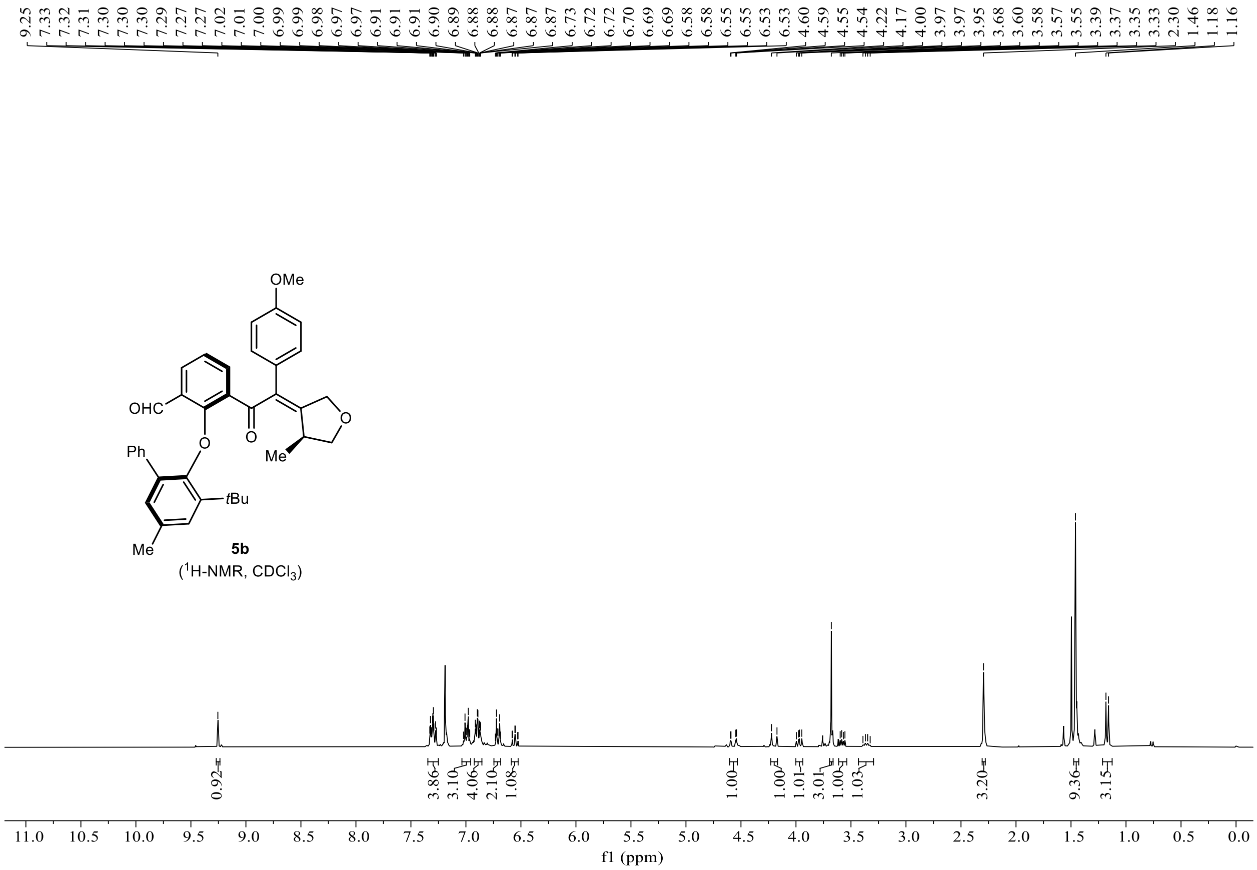
9.25 7.33 7.32 7.31 7.30 7.30 7.30 7.29 7.27 7.27 7.02 7.01 7.00 6.99 6.99 6.98 6.97 6.97 6.91 6.91 6.91 6.90 6.89 6.88 6.88 6.87 6.87 6.87 6.73 6.72 6.72 6.70 6.69 6.69 6.58 6.58 6.55 6.55 6.53 4.60 4.59 4.55 4.54 4.22 4.17 4.00 3.97 3.97 3.95 3.68 3.60 3.58 3.57 3.55 3.39 3.37 3.35 3.33 2.30 1.46 1.18 1.16
OMe
OHC
O
O
O
Ph
Me
tBu
Me
5b
(^{1}H-NMR, CDCl$_3$)
0.92
3.86
3.10
4.06
2.10
1.08
1.00
1.00
1.01
3.01
1.00
1.03
3.20
9.36
3.15
11.0 10.5 10.0 9.5 9.0 8.5 8.0 7.5 7.0 6.5 6.0 5.5 5.0 4.5 4.0 3.5 3.0 2.5 2.0 1.5 1.0 0.5 0.0
f1 (ppm)

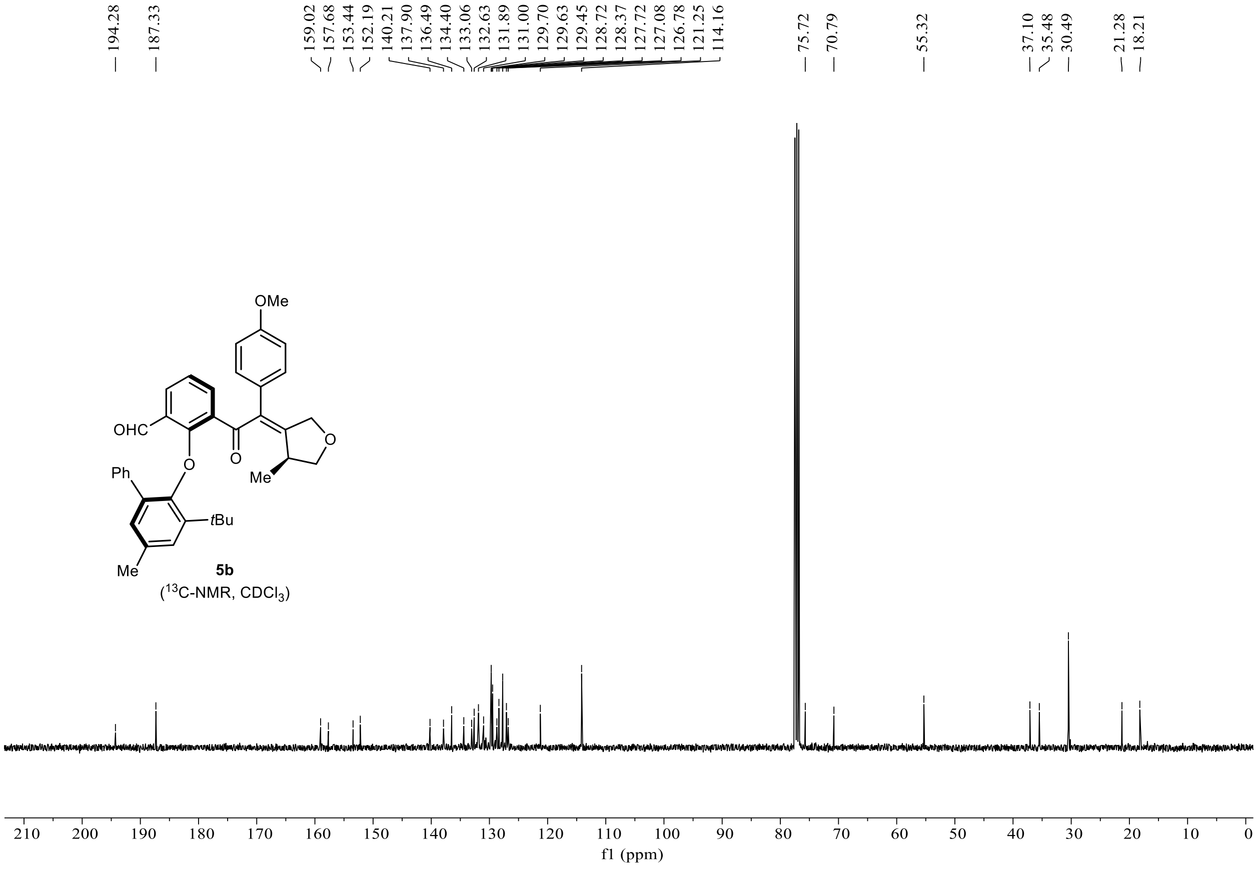
194.28 187.33 159.02 157.68 153.44 152.19 140.21 137.90 136.49 134.40 133.06 132.63 131.89 131.00 129.70 129.63 129.45 128.72 128.37 127.72 127.08 126.78 121.25 114.16 75.72 70.79 55.32 37.10 35.48 30.49 21.28 18.21
OMe
OHC
O
O
O
Ph
Me
tBu
Me
5b
(^{13}C-NMR, CDCl$_3$)
210 200 190 180 170 160 150 140 130 120 110 100 90 80 70 60 50 40 30 20 10 0
f1 (ppm)

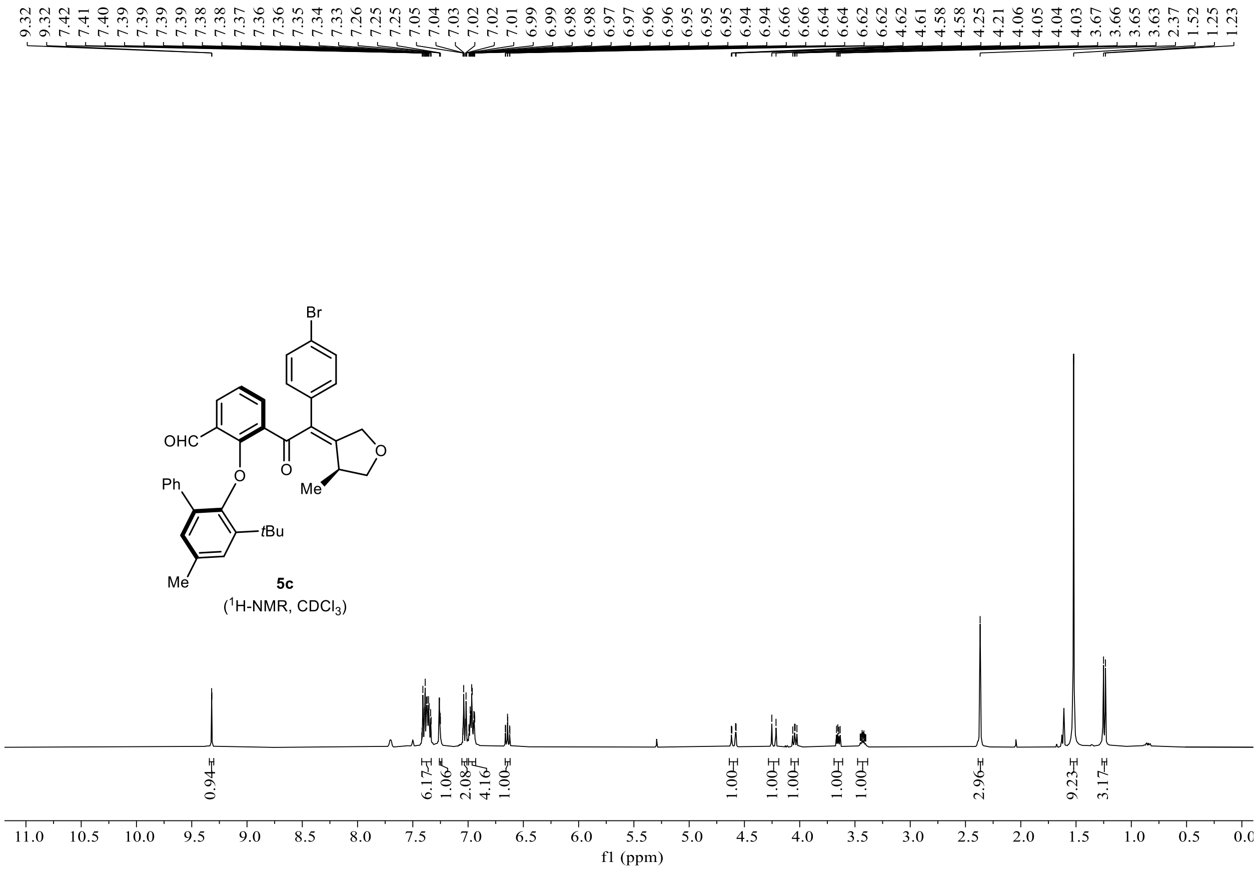
9.32 9.32 7.42 7.41 7.40 7.39 7.39 7.39 7.39 7.38 7.38 7.37 7.36 7.36 7.35 7.34 7.33 7.26 7.25 7.25 7.05 7.04 7.03 7.02 7.02 7.01 6.99 6.99 6.98 6.98 6.97 6.97 6.96 6.96 6.95 6.95 6.95 6.94 6.94 6.66 6.66 6.64 6.64 6.62 6.62 4.62 4.61 4.58 4.58 4.25 4.21 4.06 4.05 4.04 4.03 3.67 3.66 3.65 3.63 2.37 1.52 1.25 1.23
Br
OHC
O
Ph
O
Me
tBu
Me
5c
(1H-NMR, CDCl3)
0.94
6.17
1.06
2.08
4.16
1.00
1.00
1.00
1.00
1.00
1.00
2.96
9.23
3.17
11.0 10.5 10.0 9.5 9.0 8.5 8.0 7.5 7.0 6.5 6.0 5.5 5.0 4.5 4.0 3.5 3.0 2.5 2.0 1.5 1.0 0.5 0.0
f1 (ppm)

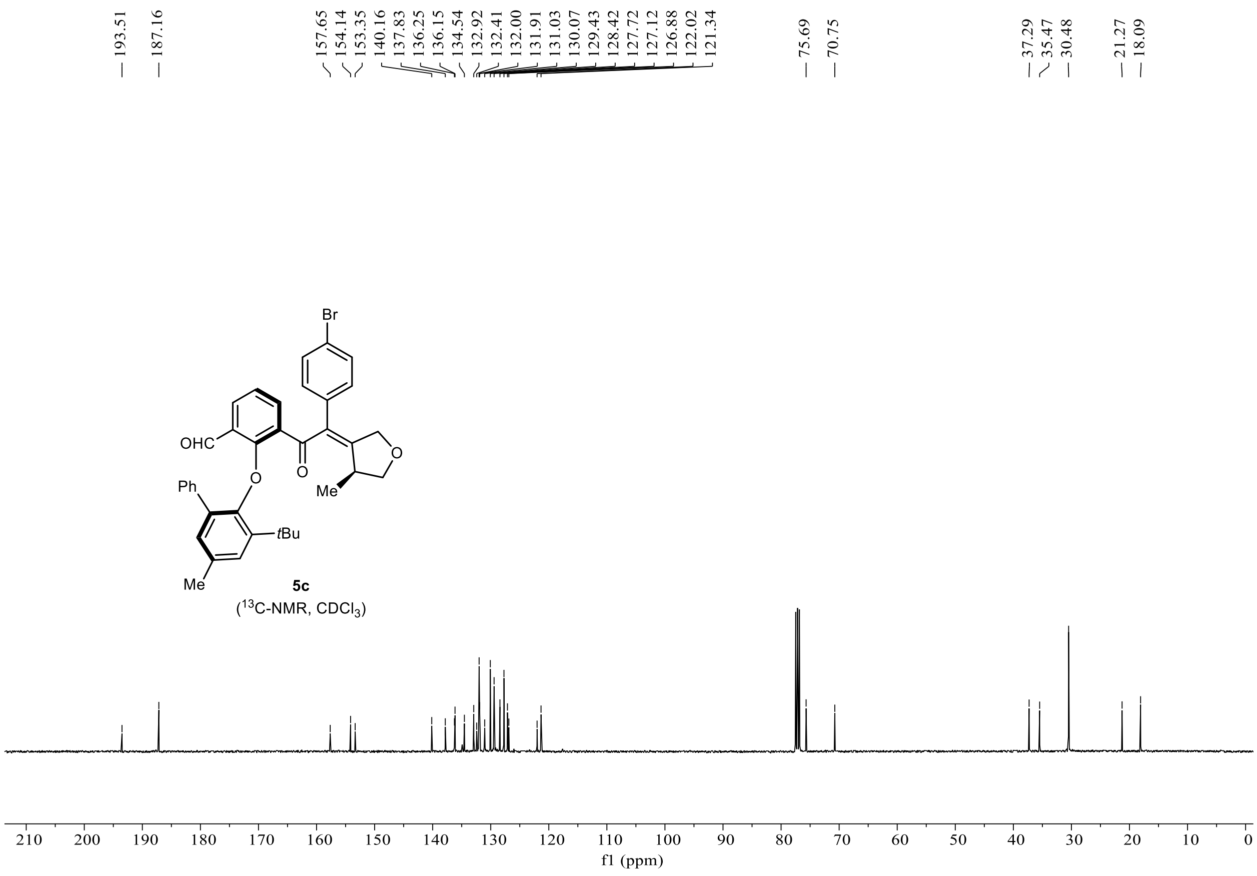
193.51 187.16 157.65 154.14 153.35 140.16 137.83 136.25 136.15 134.54 132.92 132.41 132.00 131.91 131.03 130.07 129.43 128.42 127.72 127.12 126.88 122.02 121.34 75.69 70.75 37.29 35.47 30.48 21.27 18.09
Br
OHC
O
Ph
O
Me
tBu
Me
5c
(13C-NMR, CDCl3)
210 200 190 180 170 160 150 140 130 120 110 100 90 80 70 60 50 40 30 20 10 0
f1 (ppm)

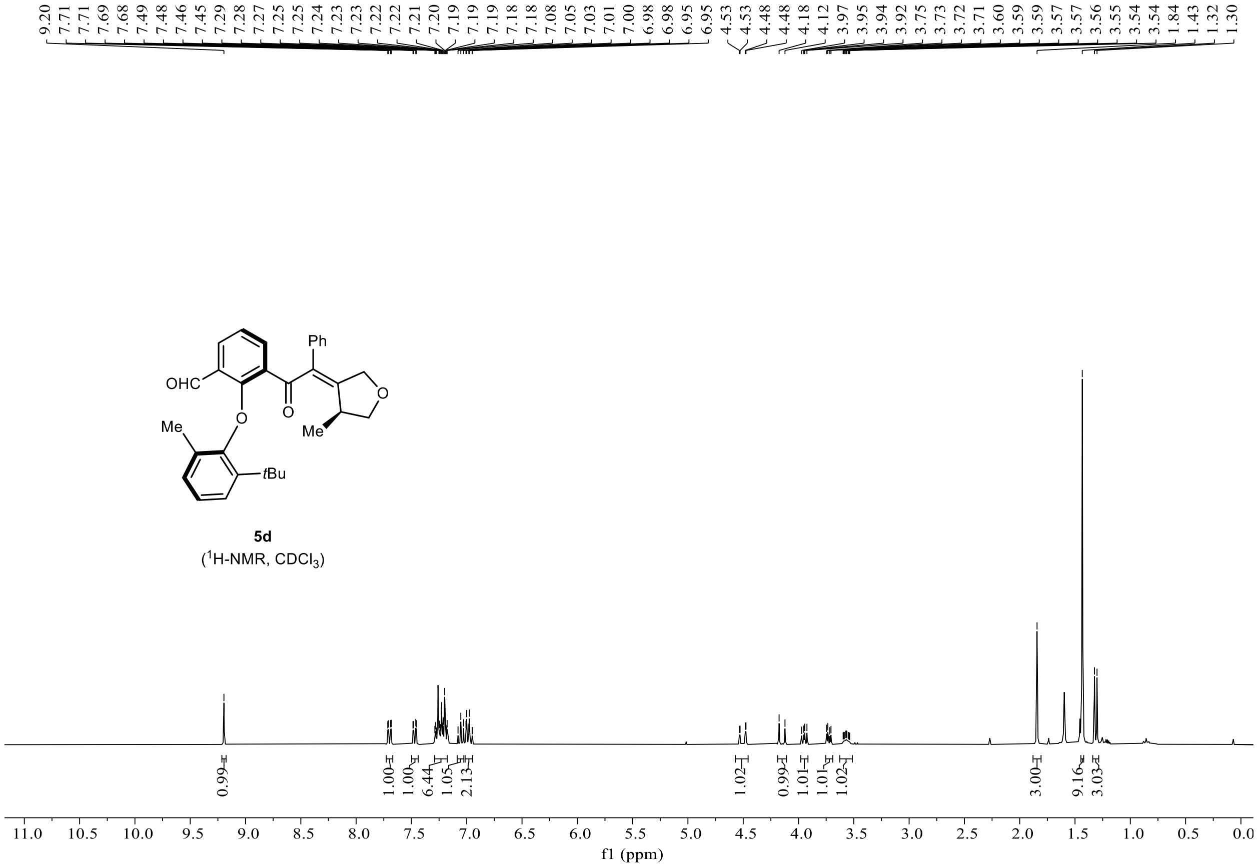
9.20 7.71 7.71 7.69 7.68 7.49 7.48 7.46 7.45 7.29 7.28 7.27 7.25 7.25 7.24 7.23 7.23 7.22 7.22 7.21 7.20 7.19 7.19 7.19 7.19 7.18 7.18 7.08 7.05 7.03 7.01 7.00 6.98 6.98 6.95 6.95 4.53 4.53 4.48 4.18 4.12 3.97 3.95 3.94 3.92 3.75 3.73 3.72 3.71 3.60 3.59 3.59 3.57 3.57 3.56 3.55 3.54 3.54 1.84 1.43 1.32 1.30
Ph
OHC
O
O
Me
O
Me
tBu
5d
(1H-NMR, CDCl3)
0.99
1.00
1.00
6.44
1.05
2.13
1.02
0.99
1.01
1.01
1.02
3.00
9.16
3.03
11.0 10.5 10.0 9.5 9.0 8.5 8.0 7.5 7.0 6.5 6.0 5.5 5.0 4.5 4.0 3.5 3.0 2.5 2.0 1.5 1.0 0.5 0.0
f1 (ppm)

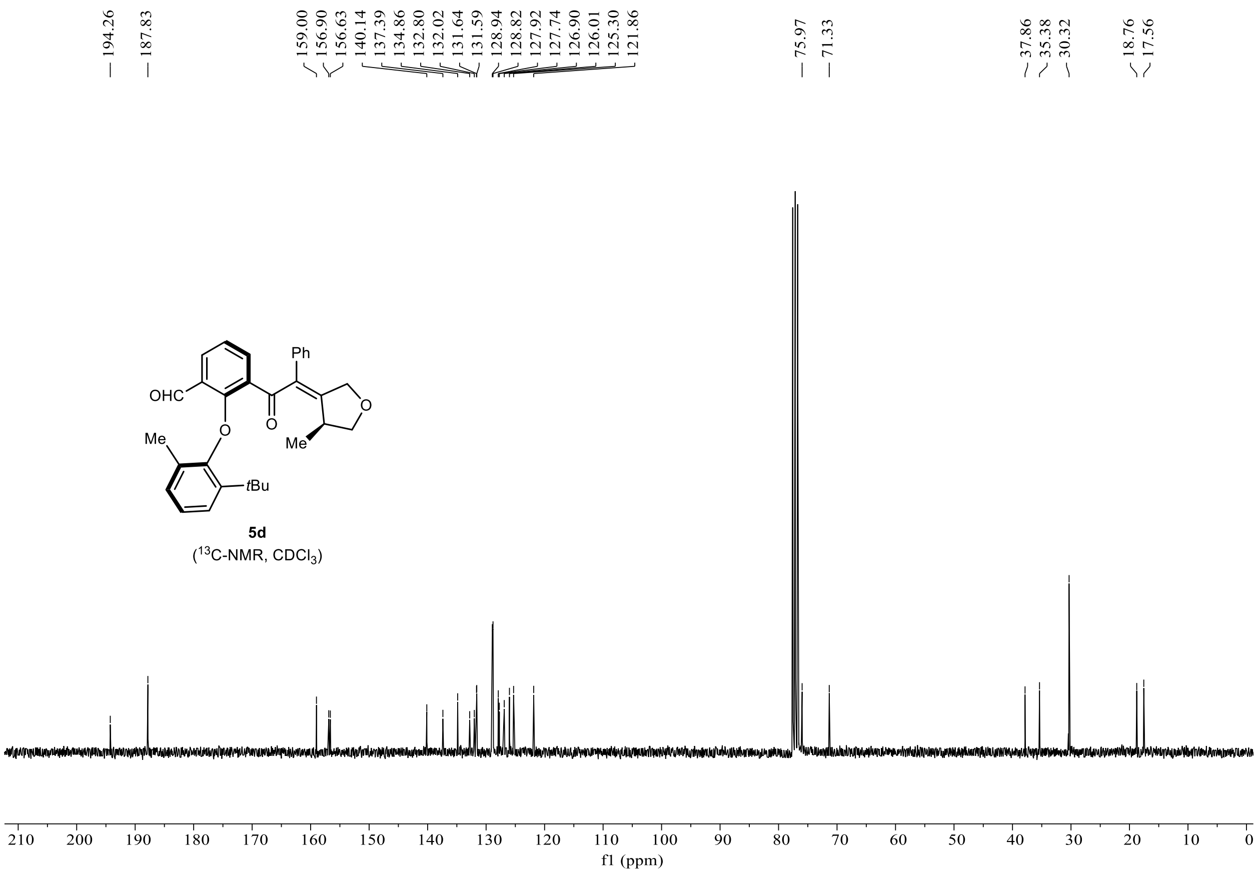
194.26 187.83 159.00 156.90 156.63 140.14 137.39 134.86 132.80 132.02 131.64 131.59 128.94 128.82 127.92 127.74 126.90 126.01 125.30 121.86 75.97 71.33 37.86 35.38 30.32 18.76 17.56
Ph
OHC
O
O
Me
O
Me
tBu
5d
(13C-NMR, CDCl3)
210 200 190 180 170 160 150 140 130 120 110 100 90 80 70 60 50 40 30 20 10 0
f1 (ppm)

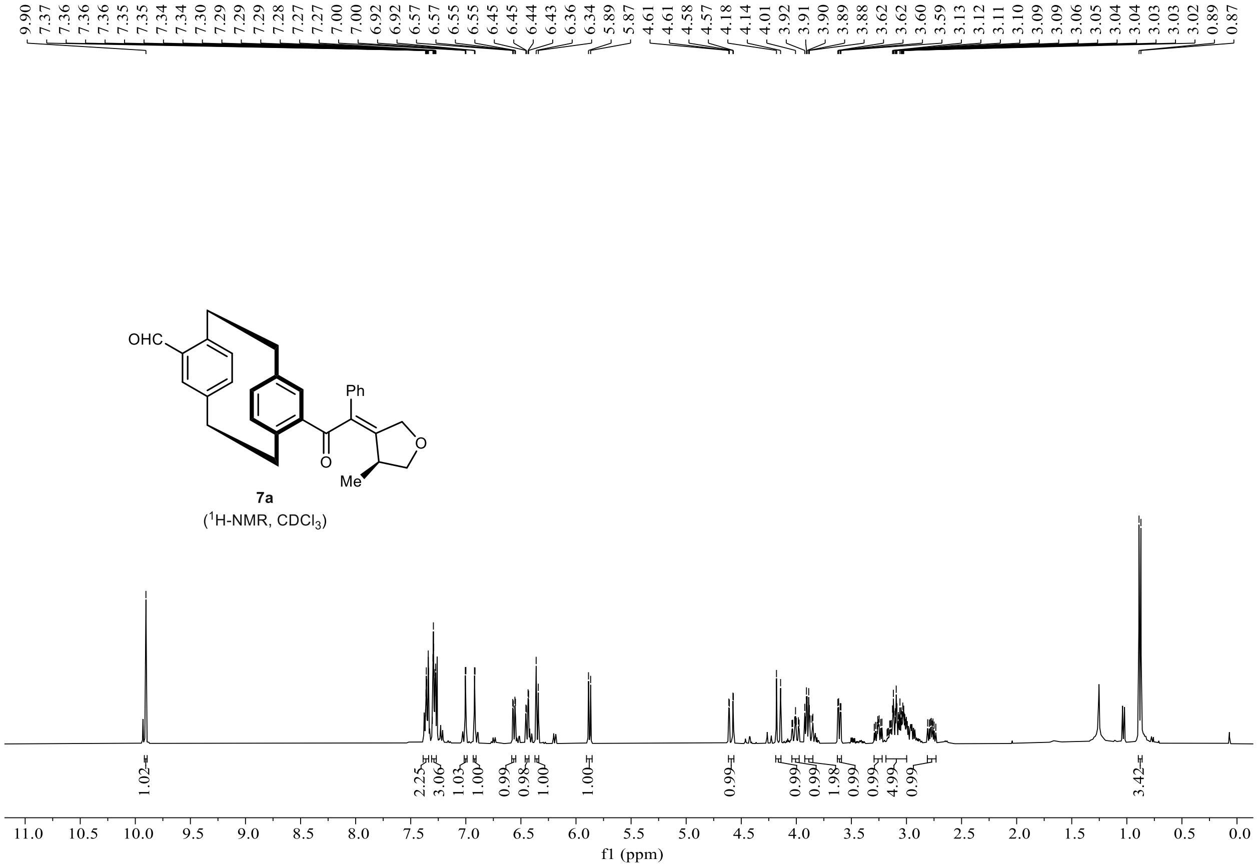
9.90 7.37 7.36 7.36 7.36 7.35 7.35 7.34 7.34 7.30 7.29 7.29 7.29 7.28 7.27 7.27 7.00 7.00 6.92 6.92 6.57 6.57 6.55 6.55 6.45 6.45 6.44 6.43 6.36 6.34 5.89 5.87 4.61 4.61 4.58 4.57 4.18 4.14 4.01 3.92 3.91 3.90 3.89 3.88 3.62 3.62 3.60 3.59 3.13 3.12 3.11 3.10 3.09 3.09 3.06 3.05 3.04 3.04 3.03 3.03 3.02 0.89 0.87
OHC
Ph
O
O
Me
7a
(^{1}H-NMR, CDCl$_3$)
1.02 2.25 3.06 1.03 1.00 0.99 0.98 1.00 1.00 0.99 0.99 0.99 1.98 0.99 0.99 4.99 0.99 3.42
11.0 10.5 10.0 9.5 9.0 8.5 8.0 7.5 7.0 6.5 6.0 5.5 5.0 4.5 4.0 3.5 3.0 2.5 2.0 1.5 1.0 0.5 0.0
f1 (ppm)

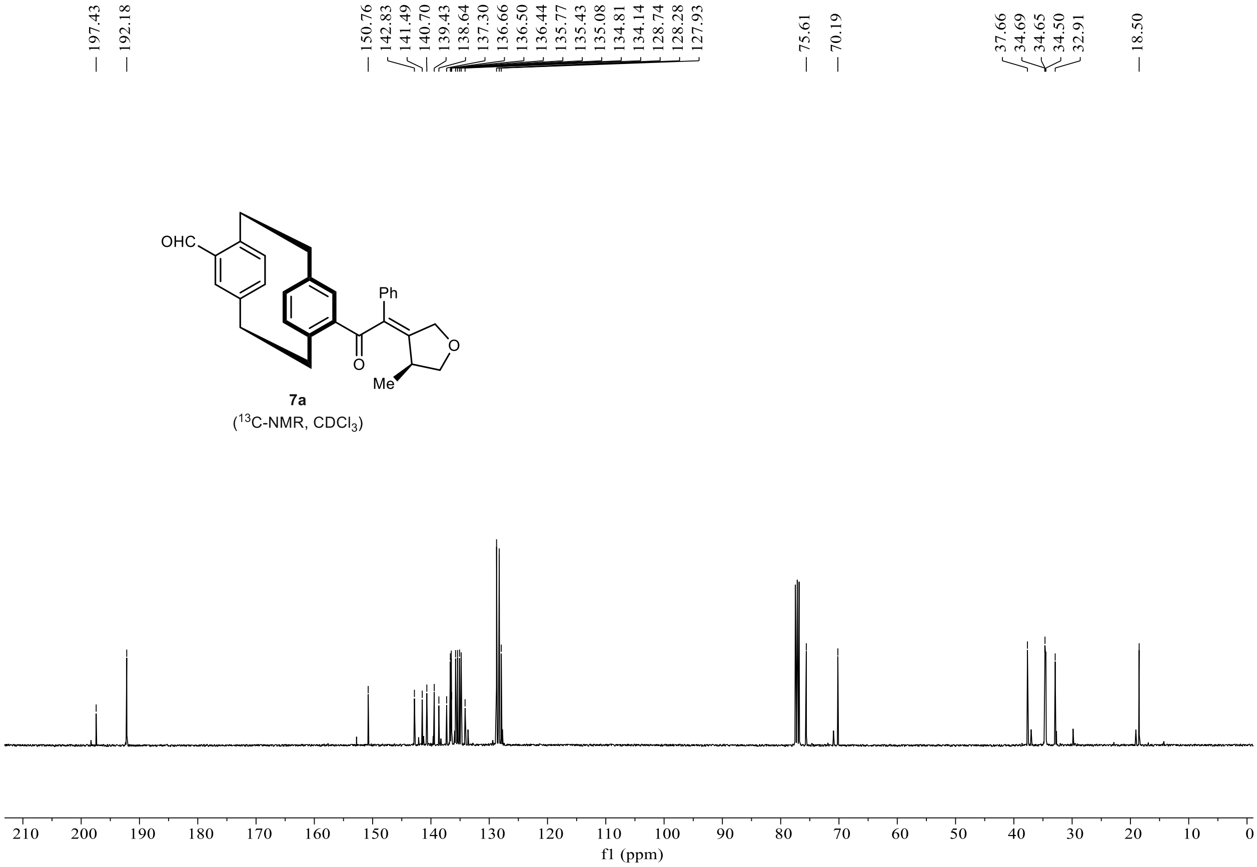
197.43 192.18 150.76 142.83 141.49 140.70 139.43 138.64 137.30 136.66 136.50 136.44 135.77 135.43 135.08 134.81 134.14 128.74 128.28 127.93 75.61 70.19 37.66 34.69 34.65 34.50 32.91 18.50
OHC
Ph
O
O
Me
7a
(^{13}C-NMR, CDCl$_3$)
210 200 190 180 170 160 150 140 130 120 110 100 90 80 70 60 50 40 30 20 10 0
f1 (ppm)

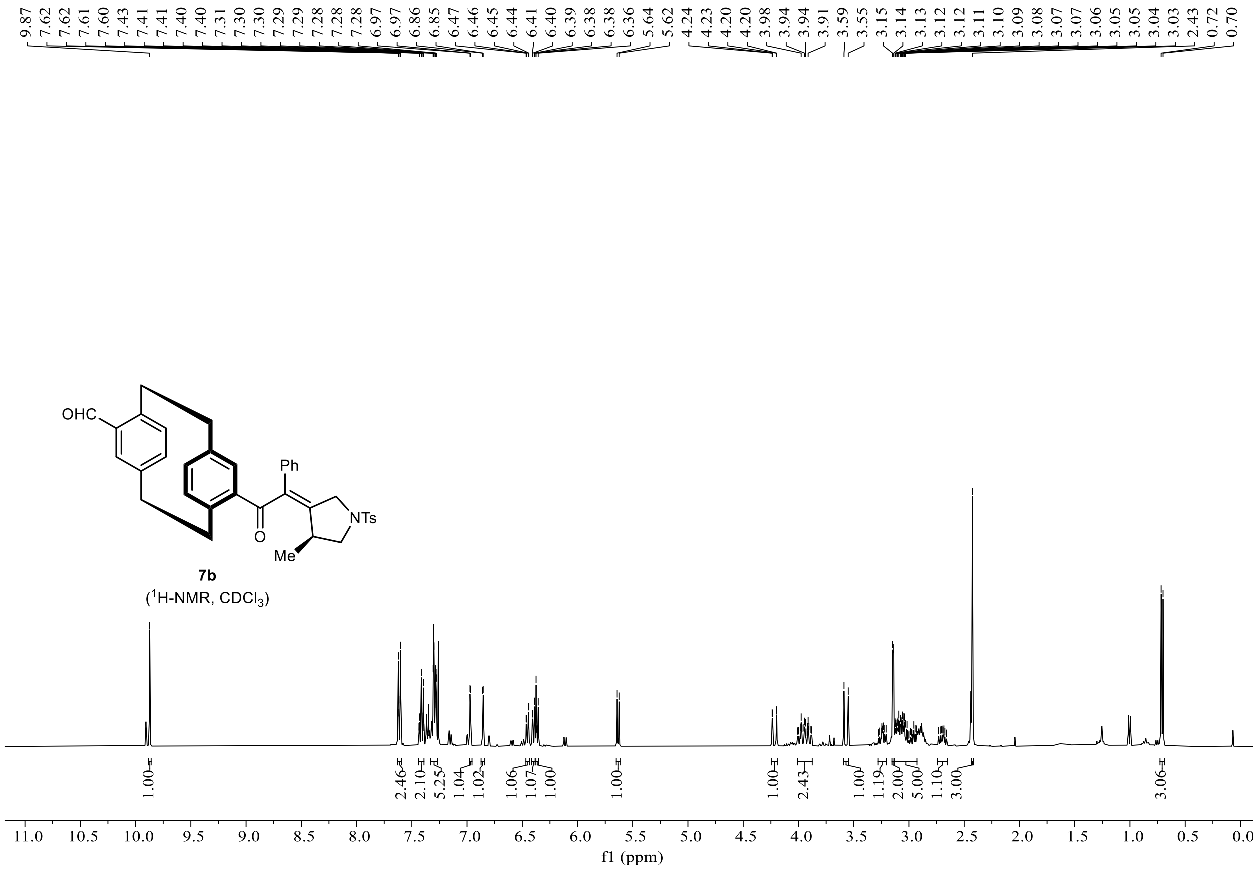
9.87 7.62 7.62 7.61 7.60 7.43 7.41 7.41 7.40 7.40 7.31 7.30 7.29 7.29 7.28 7.28 7.28 6.97 6.97 6.86 6.85 6.47 6.46 6.45 6.44 6.41 6.40 6.39 6.38 6.38 6.36 5.64 5.62 4.24 4.23 4.20 4.20 3.98 3.94 3.94 3.91 3.59 3.55 3.15 3.14 3.13 3.12 3.12 3.11 3.10 3.09 3.08 3.07 3.07 3.06 3.05 3.05 3.04 3.03 2.43 0.72 0.70
OHC
Ph
NTs
O
Me
7b
(^{1}H-NMR, CDCl$_3$)
1.00 2.46 2.10 5.25 1.04 1.02 1.06 1.07 1.00 1.00 1.00 2.43 1.00 1.19 2.00 5.00 1.10 3.00 3.06
11.0 10.5 10.0 9.5 9.0 8.5 8.0 7.5 7.0 6.5 6.0 5.5 5.0 4.5 4.0 3.5 3.0 2.5 2.0 1.5 1.0 0.5 0.0
f1 (ppm)

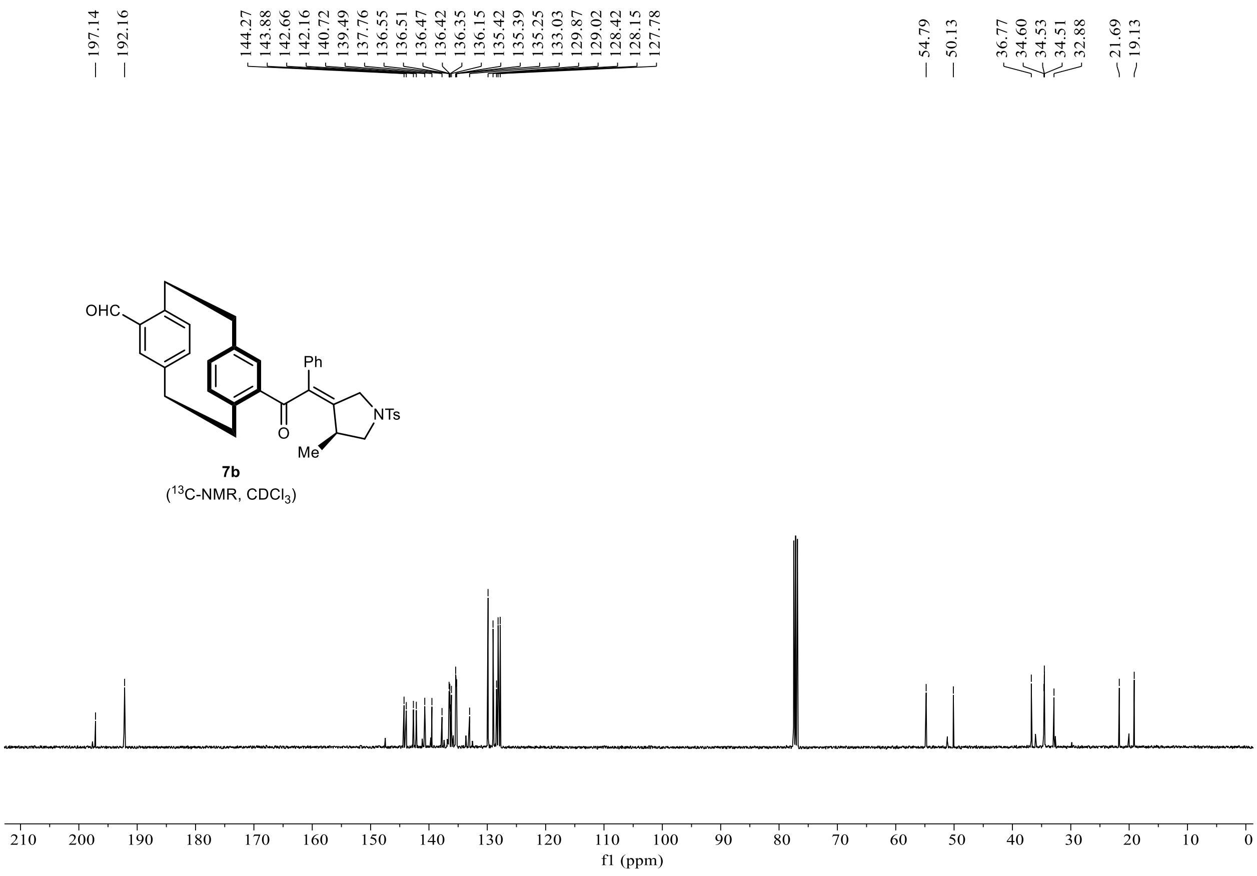
197.14 192.16 144.27 143.88 142.66 142.16 140.72 139.49 137.76 136.55 136.51 136.47 136.42 136.35 136.15 135.42 135.39 135.25 133.03 129.87 129.02 128.42 128.15 127.78 54.79 50.13 36.77 34.60 34.53 34.51 32.88 21.69 19.13
OHC
Ph
NTs
O
Me
7b
(^{13}C-NMR, CDCl$_3$)
210 200 190 180 170 160 150 140 130 120 110 100 90 80 70 60 50 40 30 20 10 0
f1 (ppm)

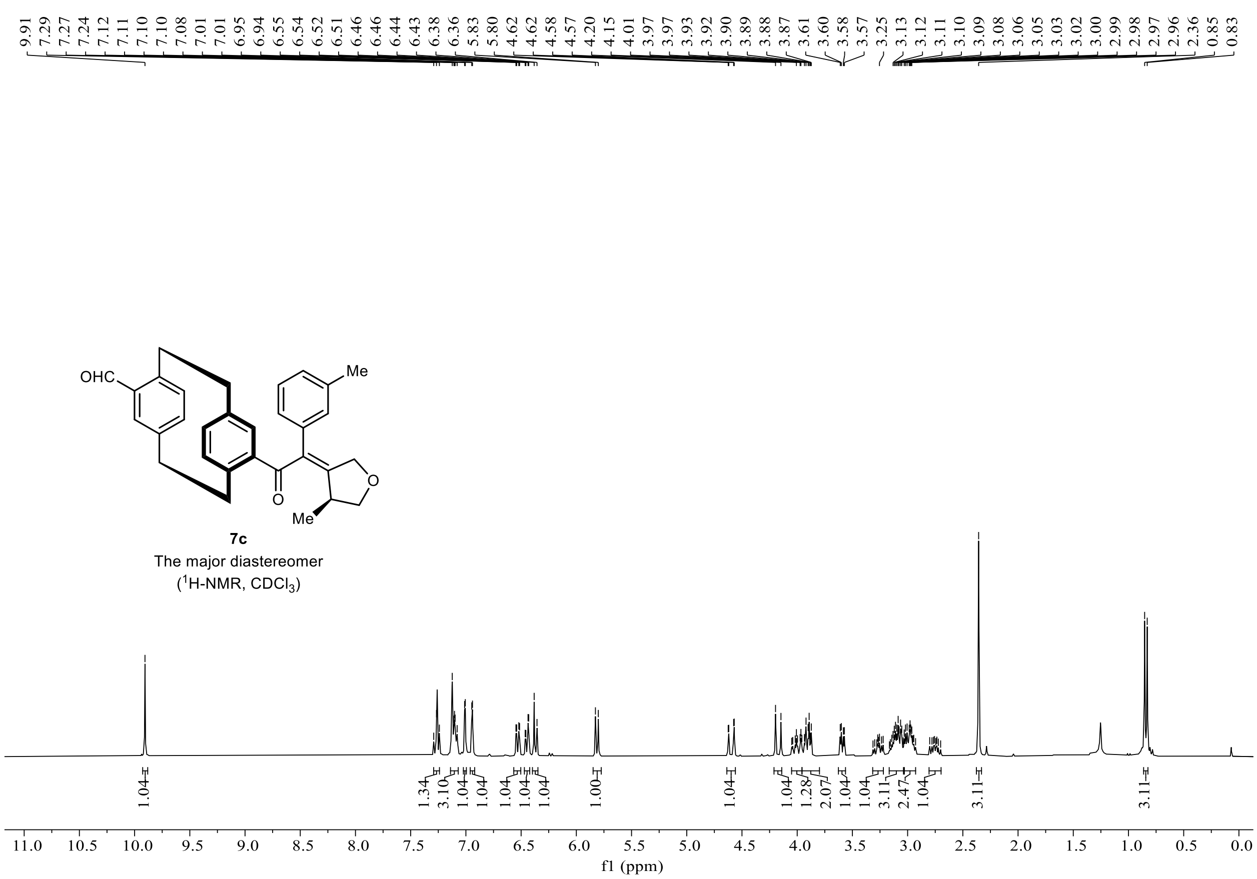

7c
The major diastereomer
(^{1}H-NMR, $CDCl_3$)

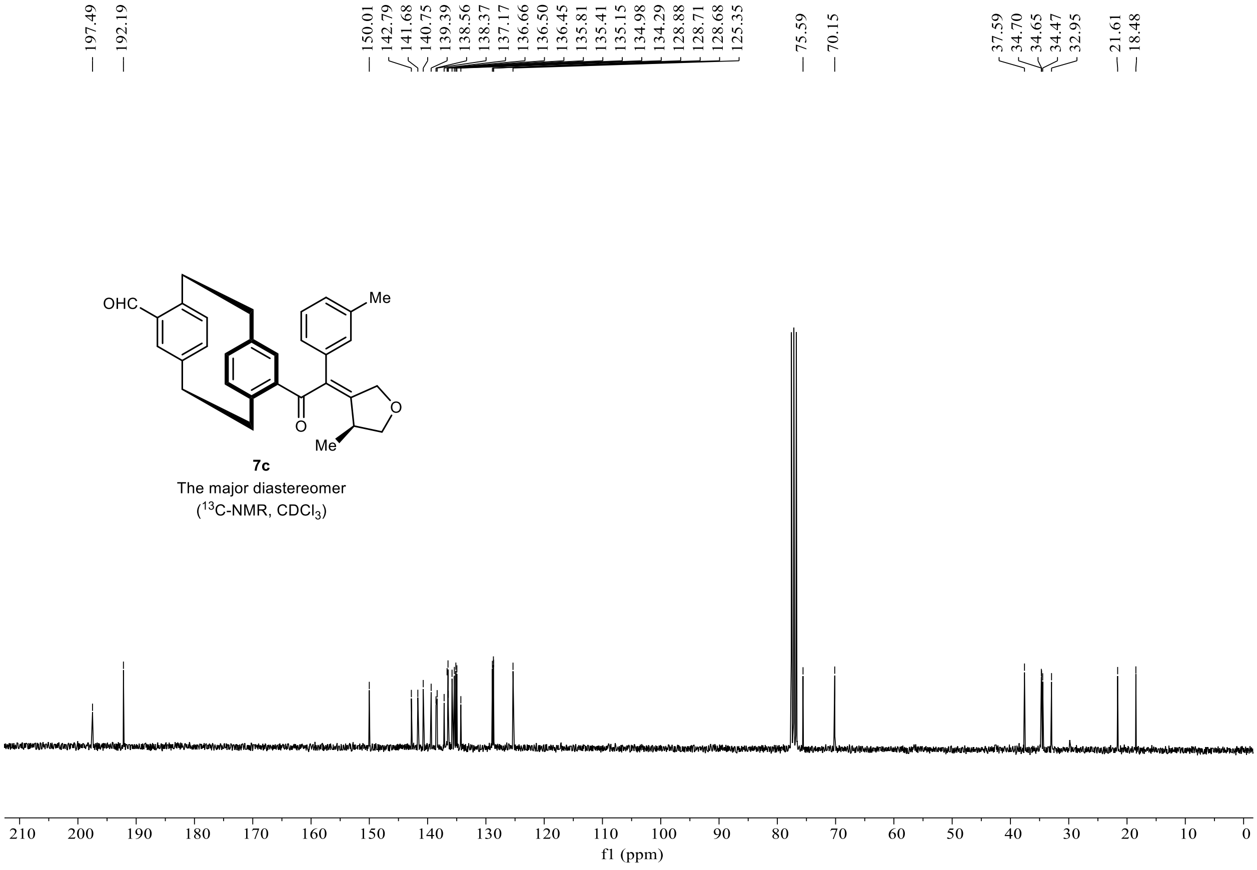

7c
The major diastereomer
(^{13}C-NMR, $CDCl_3$)

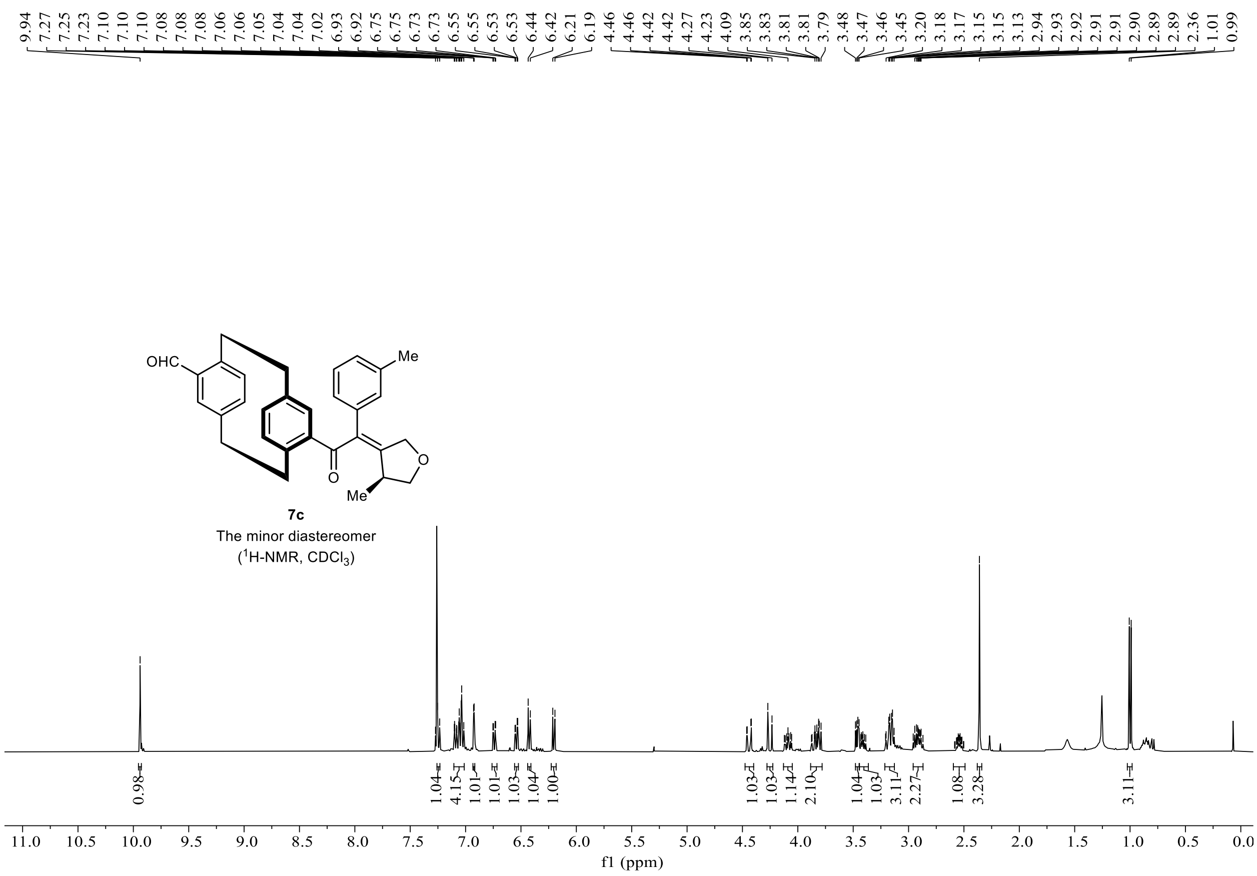
9.94 7.27 7.25 7.23 7.10 7.10 7.10 7.08 7.08 7.08 7.06 7.06 7.05 7.04 7.04 7.02 6.93 6.92 6.75 6.75 6.73 6.73 6.55 6.55 6.53 6.44 6.42 6.21 6.19 4.46 4.46 4.42 4.42 4.27 4.23 4.09 3.85 3.83 3.81 3.81 3.79 3.48 3.47 3.46 3.45 3.20 3.18 3.17 3.15 3.15 3.13 2.94 2.93 2.92 2.92 2.91 2.91 2.90 2.89 2.89 2.36 1.01 0.99
OHC
Me
O
O
Me
7c
The minor diastereomer
(^{1}H-NMR, $CDCl_3$)
0.98 1.04 4.15 1.01 1.01 1.03 1.04 1.00 1.03 1.03 1.14 2.10 1.04 1.03 3.11 2.27 1.08 3.28 3.11
11.0 10.5 10.0 9.5 9.0 8.5 8.0 7.5 7.0 6.5 6.0 5.5 5.0 4.5 4.0 3.5 3.0 2.5 2.0 1.5 1.0 0.5 0.0
f1 (ppm)

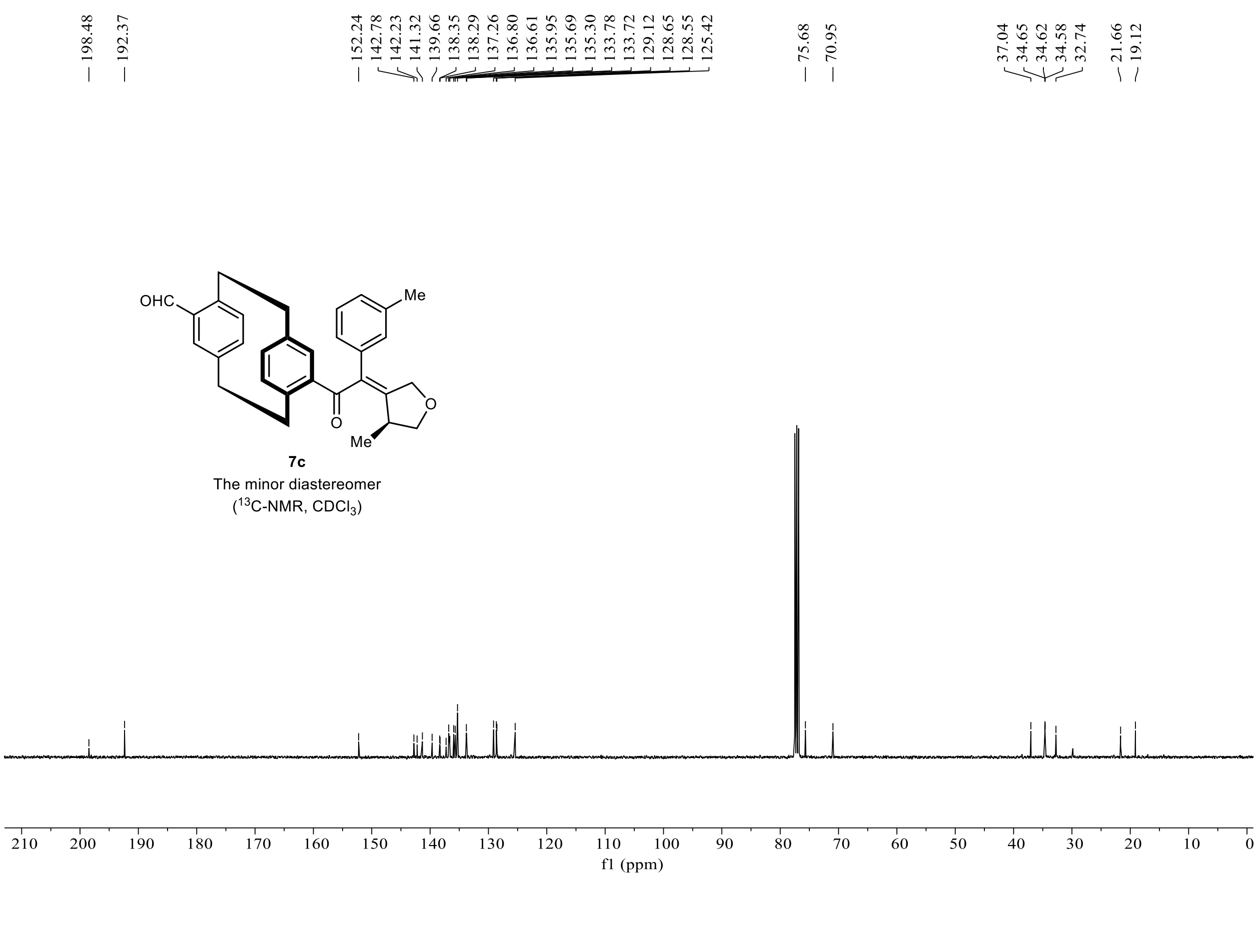
198.48 192.37 152.24 142.78 142.23 141.32 139.66 138.35 138.29 137.26 136.80 136.61 135.95 135.69 135.30 133.78 133.72 129.12 128.65 128.55 125.42 75.68 70.95 37.04 34.65 34.62 34.58 32.74 21.66 19.12
OHC
Me
O
O
Me
7c
The minor diastereomer
(^{13}C-NMR, $CDCl_3$)
210 200 190 180 170 160 150 140 130 120 110 100 90 80 70 60 50 40 30 20 10 0
f1 (ppm)

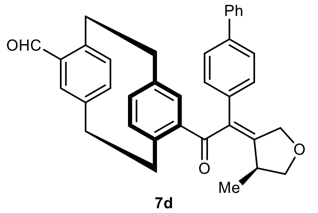

7d
(^{1}H-NMR, $CDCl_3$)

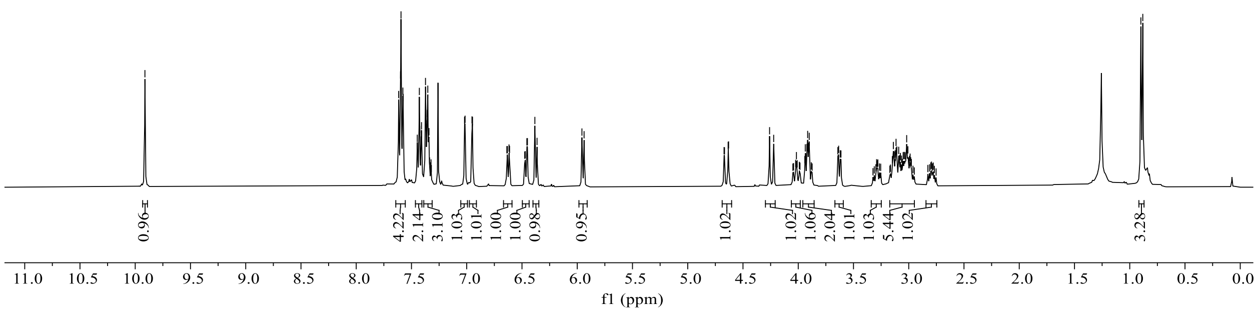

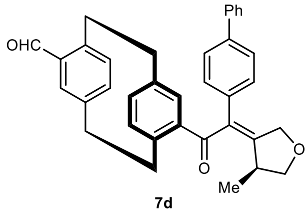

7d
(^{13}C-NMR, $CDCl_3$)

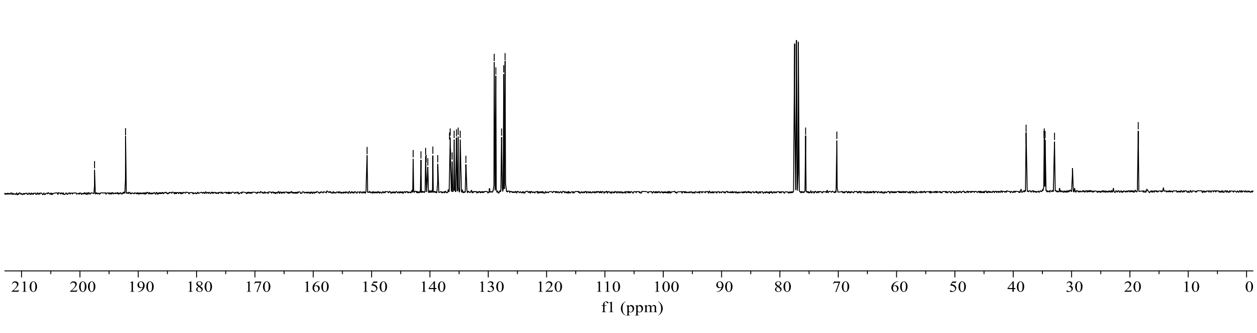

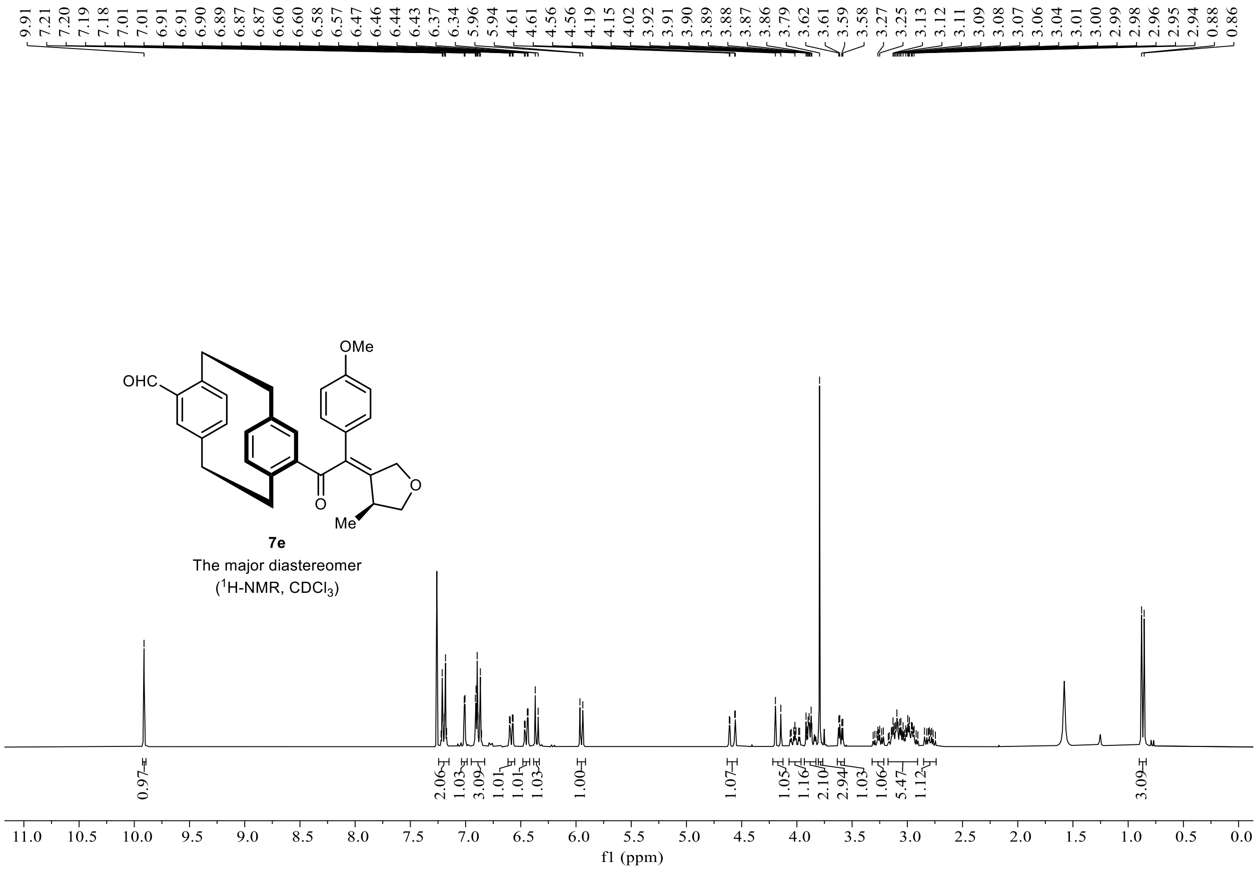
9.91 7.21 7.20 7.19 7.18 7.01 7.01 6.91 6.91 6.90 6.89 6.87 6.87 6.60 6.60 6.58 6.57 6.47 6.46 6.44 6.43 6.37 6.34 5.96 5.94 4.61 4.61 4.56 4.56 4.19 4.15 4.02 3.92 3.91 3.90 3.89 3.88 3.87 3.86 3.79 3.62 3.61 3.59 3.58 3.27 3.25 3.13 3.12 3.11 3.09 3.08 3.07 3.06 3.04 3.01 3.00 2.99 2.98 2.96 2.95 2.94 0.88 0.86
OMe
OHC
O
Me
7e
The major diastereomer
(^{1}H-NMR, CDCl$_3$)
0.97 2.06 1.03 3.09 1.01 1.01 1.03 1.00 1.07 1.05 1.16 2.10 2.94 1.03 1.06 5.47 1.12 3.09
f1 (ppm)

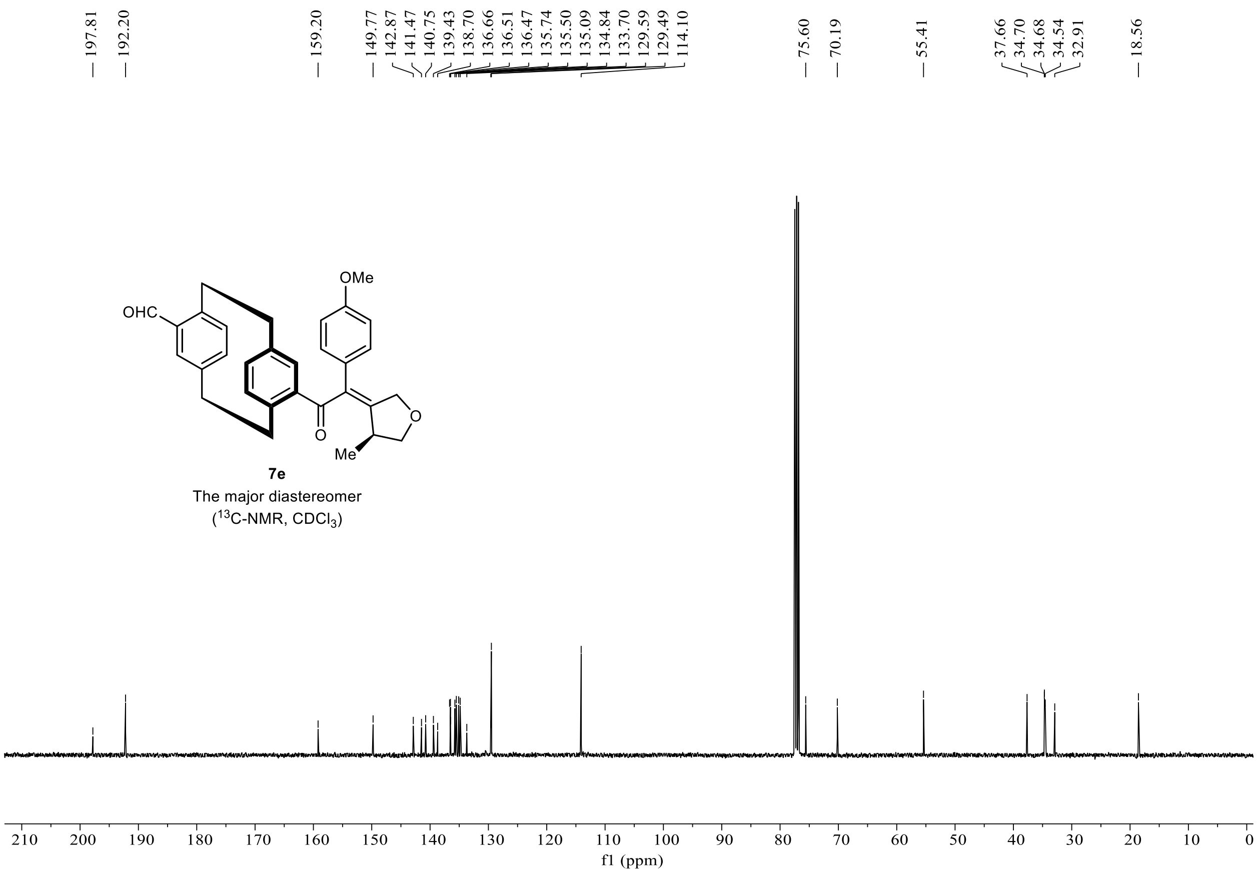
197.81 192.20 159.20 149.77 142.87 141.47 140.75 139.43 138.70 136.66 136.51 136.47 135.74 135.50 135.09 134.84 133.70 129.59 129.49 114.10 75.60 70.19 55.41 37.66 34.70 34.68 34.54 32.91 18.56
OMe
OHC
O
Me
7e
The major diastereomer
(^{13}C-NMR, CDCl$_3$)
f1 (ppm)

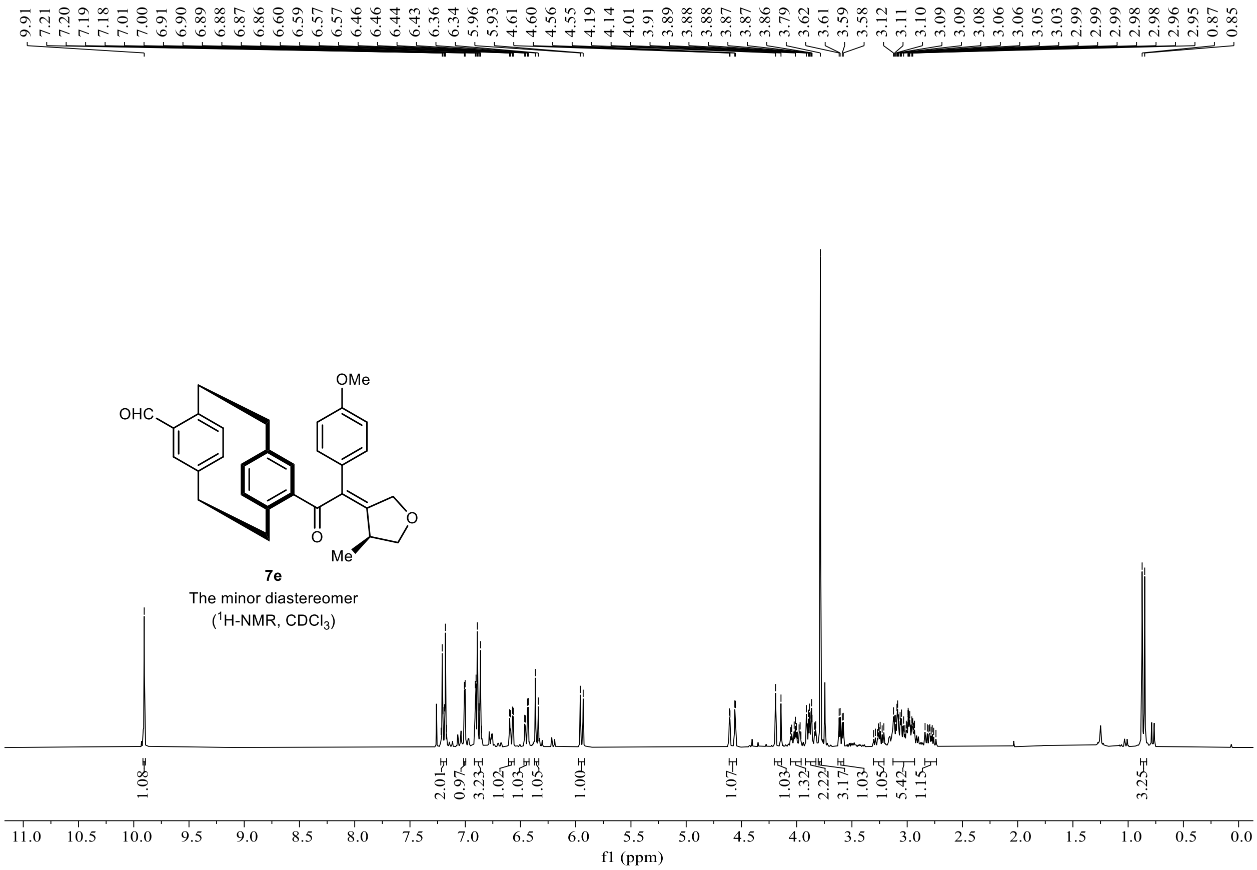
9.91 7.21 7.20 7.19 7.18 7.01 7.00 6.91 6.90 6.89 6.88 6.87 6.86 6.60 6.59 6.57 6.57 6.46 6.44 6.43 6.36 6.34 5.96 5.93 4.61 4.60 4.56 4.55 4.19 4.14 4.01 3.91 3.89 3.88 3.88 3.87 3.87 3.86 3.79 3.62 3.61 3.59 3.58 3.12 3.11 3.10 3.09 3.09 3.08 3.06 3.06 3.05 3.03 2.99 2.99 2.99 2.98 2.98 2.96 2.95 0.87 0.85
OHC
OMe
O
O
Me
7e
The minor diastereomer
(^{1}H-NMR, $CDCl_3$)
1.08
2.01 0.97 3.23 1.02 1.03 1.05 1.00
1.07
1.03 1.32 2.22 3.17 1.03 1.05 5.42 1.15
3.25
11.0 10.5 10.0 9.5 9.0 8.5 8.0 7.5 7.0 6.5 6.0 5.5 5.0 4.5 4.0 3.5 3.0 2.5 2.0 1.5 1.0 0.5 0.0
f1 (ppm)

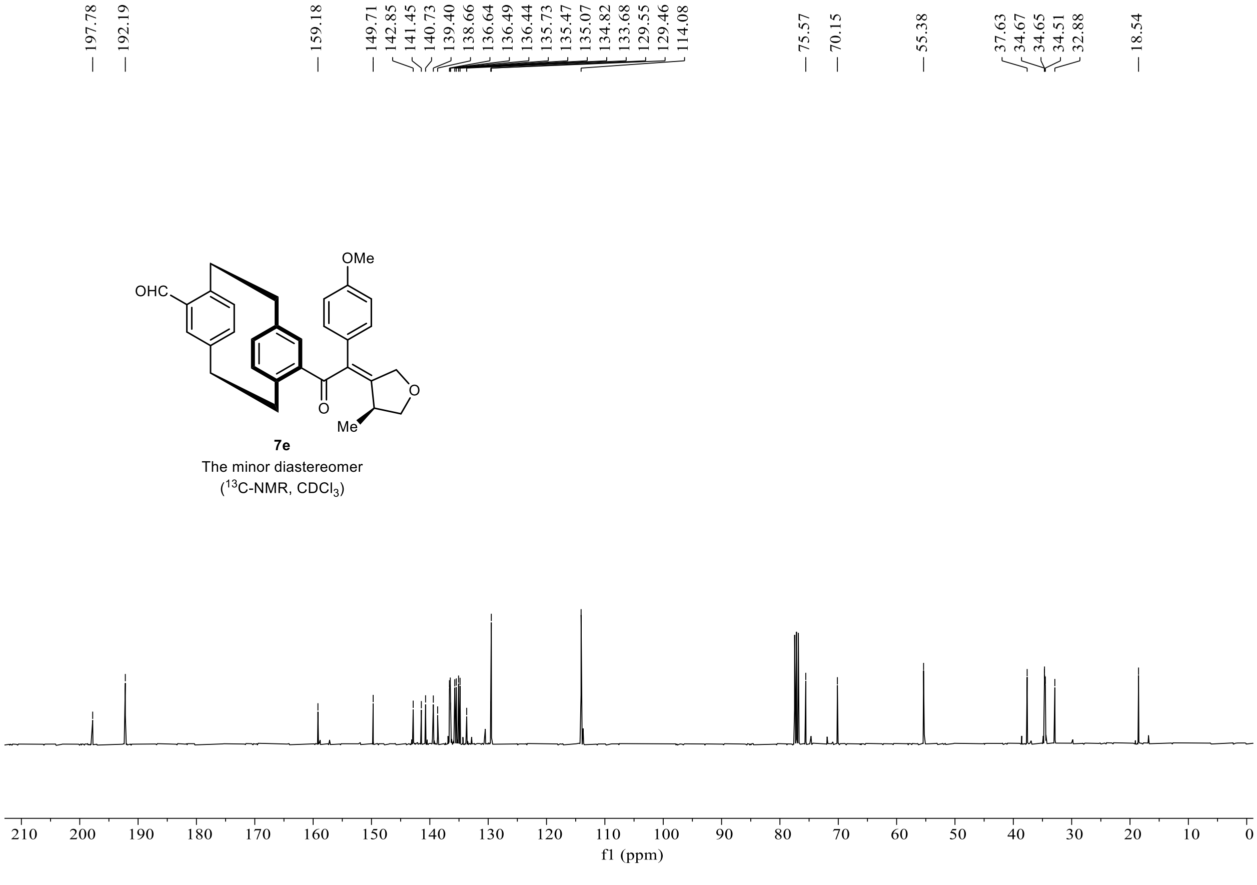
197.78 192.19 159.18 149.71 142.85 141.45 140.73 139.40 138.66 136.64 136.49 136.44 135.73 135.47 135.07 134.82 133.68 129.55 129.46 114.08 75.57 70.15 55.38 37.63 34.67 34.65 34.51 32.88 18.54
OHC
OMe
O
O
Me
7e
The minor diastereomer
(^{13}C-NMR, $CDCl_3$)
210 200 190 180 170 160 150 140 130 120 110 100 90 80 70 60 50 40 30 20 10 0
f1 (ppm)

9.91, 7.12, 7.12, 7.10, 7.10, 7.01, 7.00, 6.82, 6.82, 6.64, 6.63, 6.62, 6.61, 6.47, 6.47, 6.45, 6.44, 6.35, 6.33, 6.09, 6.07, 4.54, 4.54, 4.49, 4.48, 4.14, 4.09, 4.04, 4.03, 3.90, 3.88, 3.88, 3.86, 3.75, 3.74, 3.73, 3.64, 3.63, 3.61, 3.60, 3.24, 3.23, 3.21, 3.19, 3.14, 3.13, 3.12, 3.10, 3.08, 3.08, 3.06, 3.05, 3.03, 3.02, 3.01, 3.01, 3.00, 2.99, 2.99, 2.97, 2.96, 2.94, 2.93, 2.92, 2.92, 2.88, 2.83, 0.91, 0.89

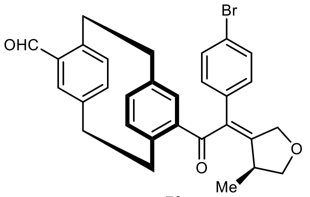

7f
The major diastereomer
(^{1}H-NMR, $CDCl_3$)

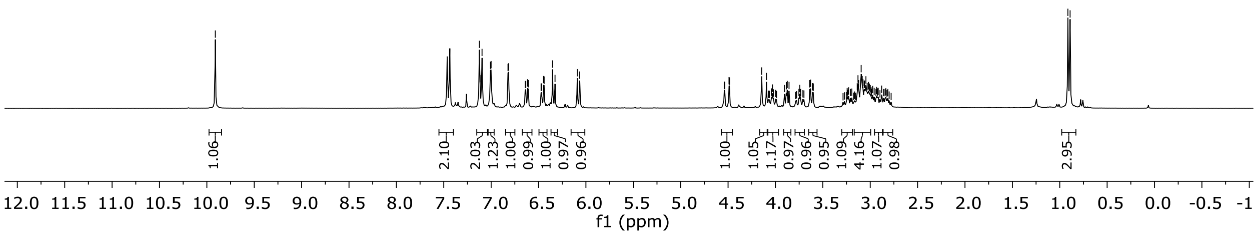

197.04, 192.11, 152.57, 142.88, 140.96, 140.59, 139.56, 138.60, 136.51, 136.46, 136.39, 136.27, 135.76, 135.42, 134.99, 134.21, 132.95, 131.82, 129.91, 121.95, 75.59, 70.22, 37.90, 34.68, 34.64, 34.44, 32.83, 18.55

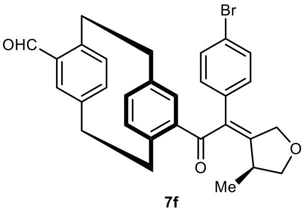

7f
The major diastereomer
(^{13}C-NMR, $CDCl_3$)

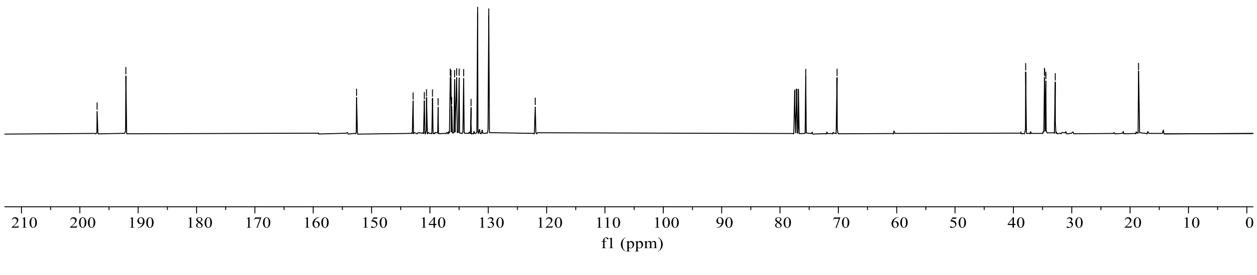

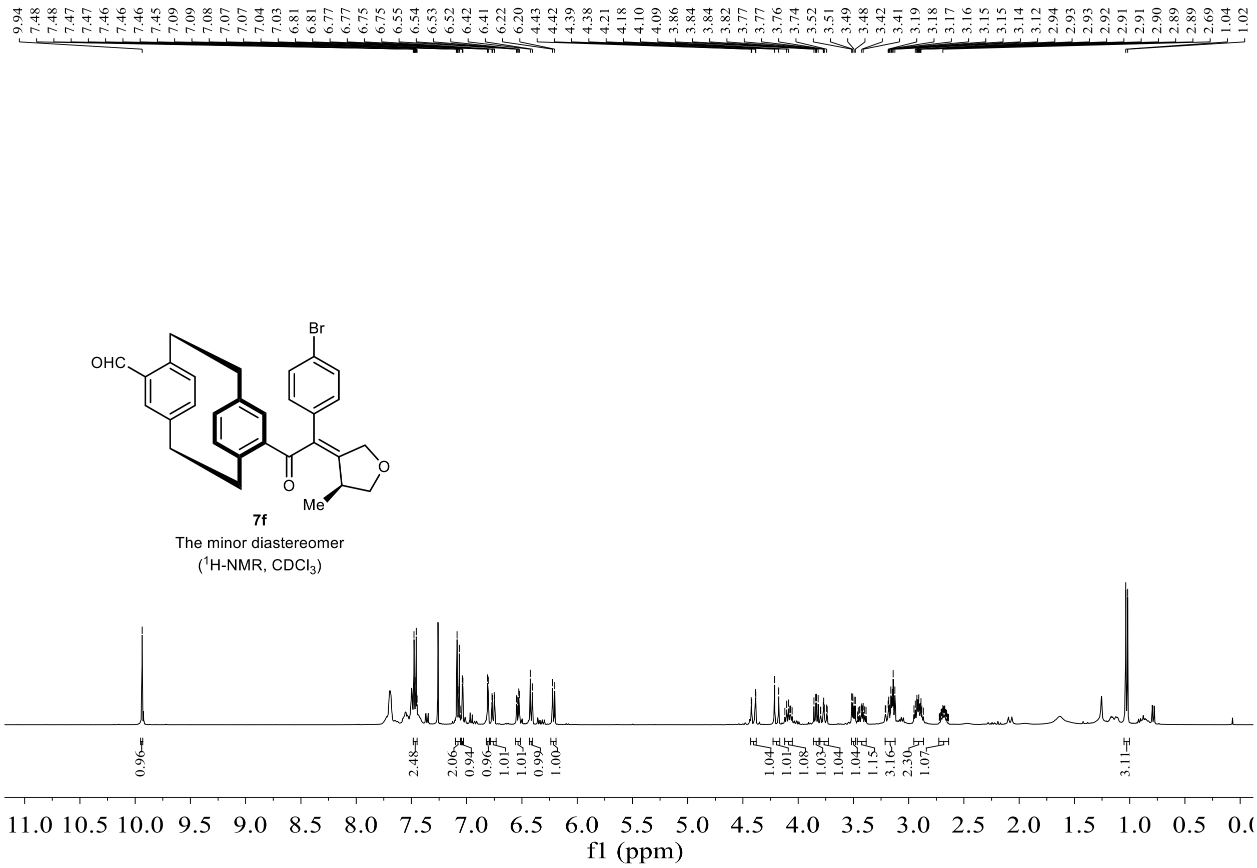
9.94 7.48 7.48 7.47 7.47 7.46 7.46 7.45 7.09 7.09 7.08 7.07 7.07 7.04 7.03 6.81 6.81 6.77 6.77 6.75 6.75 6.55 6.54 6.53 6.52 6.42 6.41 6.22 6.20 4.43 4.42 4.39 4.38 4.21 4.18 4.10 4.09 3.86 3.84 3.84 3.82 3.77 3.77 3.76 3.74 3.52 3.51 3.49 3.48 3.42 3.41 3.19 3.18 3.17 3.16 3.15 3.15 3.14 3.12 2.94 2.93 2.93 2.92 2.91 2.91 2.90 2.89 2.89 2.69 1.04 1.02
Br
OHC
O
O
Me
7f
The minor diastereomer
(^{1}H-NMR, CDCl$_3$)
0.96 2.48 2.06 0.94 0.96 1.01 1.01 0.99 1.00 1.04 1.01 1.08 1.03 1.04 1.04 1.15 3.16 2.30 1.07 3.11
11.0 10.5 10.0 9.5 9.0 8.5 8.0 7.5 7.0 6.5 6.0 5.5 5.0 4.5 4.0 3.5 3.0 2.5 2.0 1.5 1.0 0.5
f1 (ppm)

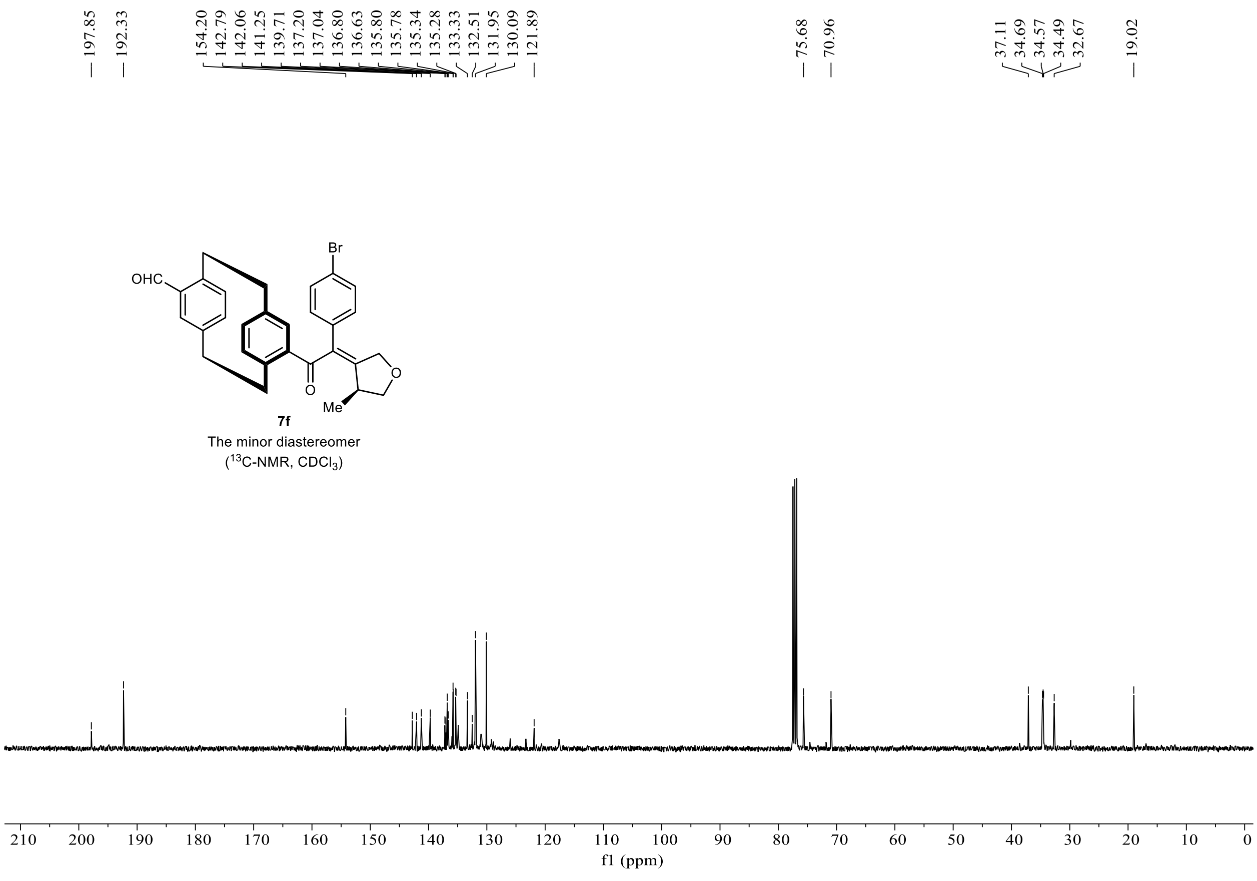
197.85 192.33 154.20 142.79 142.06 141.25 139.71 137.20 137.04 136.80 136.63 135.80 135.78 135.34 135.28 133.33 132.51 131.95 130.09 121.89 75.68 70.96 37.11 34.69 34.57 34.49 32.67 19.02
Br
OHC
O
O
Me
7f
The minor diastereomer
(^{13}C-NMR, CDCl$_3$)
210 200 190 180 170 160 150 140 130 120 110 100 90 80 70 60 50 40 30 20 10 0
f1 (ppm)

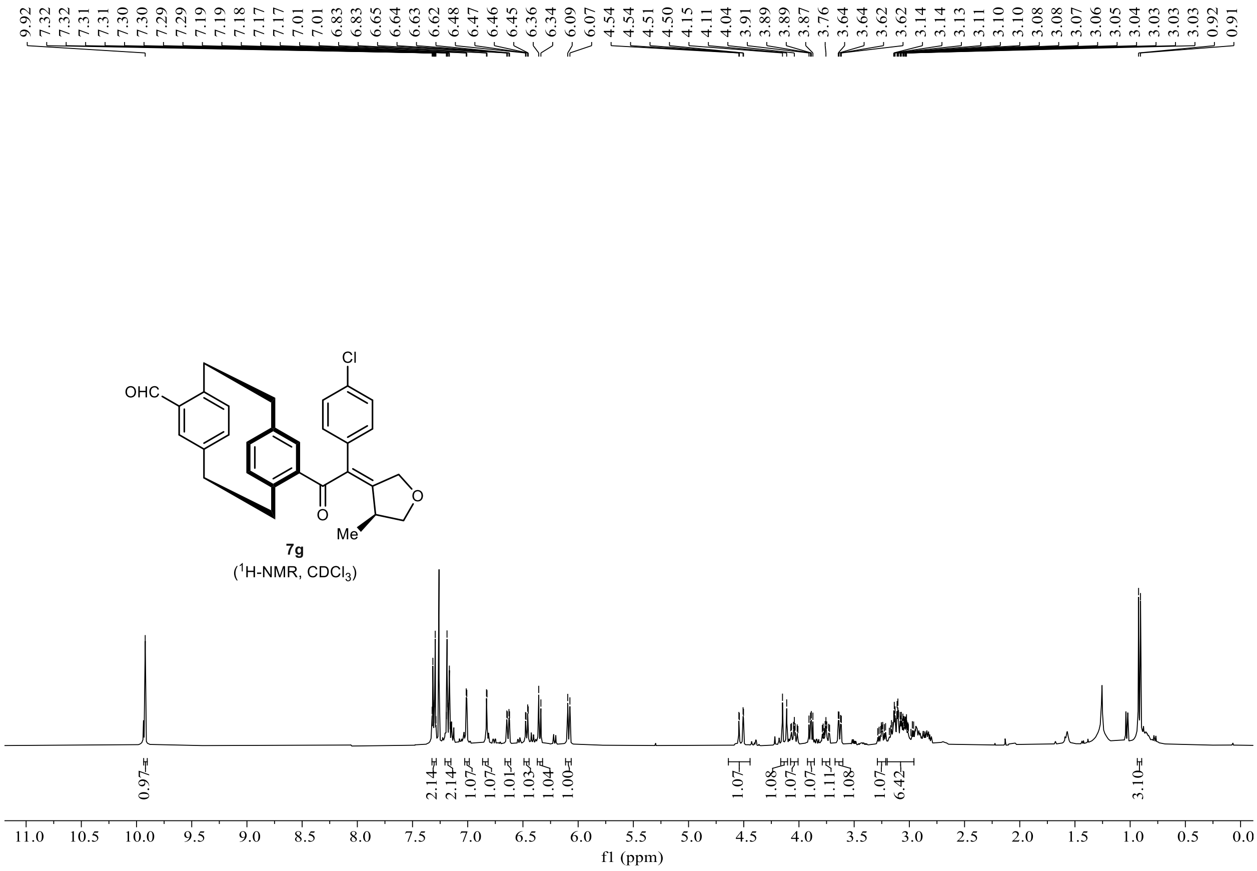
9.92 7.32 7.32 7.31 7.31 7.30 7.30 7.29 7.29 7.19 7.19 7.18 7.17 7.17 7.01 7.01 6.83 6.83 6.65 6.64 6.63 6.62 6.48 6.47 6.46 6.45 6.36 6.34 6.09 6.07 4.54 4.54 4.51 4.50 4.15 4.11 4.04 3.91 3.89 3.89 3.87 3.76 3.64 3.64 3.62 3.62 3.14 3.14 3.13 3.11 3.10 3.10 3.08 3.08 3.07 3.06 3.05 3.04 3.03 3.03 3.03 0.92 0.91
Cl
OHC
O
O
Me
7g
(^{1}H-NMR, $CDCl_3$)
0.97 2.14 2.14 1.07 1.07 1.01 1.03 1.04 1.00 1.07 1.08 1.07 1.07 1.11 1.08 1.07 6.42 3.10
11.0 10.5 10.0 9.5 9.0 8.5 8.0 7.5 7.0 6.5 6.0 5.5 5.0 4.5 4.0 3.5 3.0 2.5 2.0 1.5 1.0 0.5 0.0
f1 (ppm)

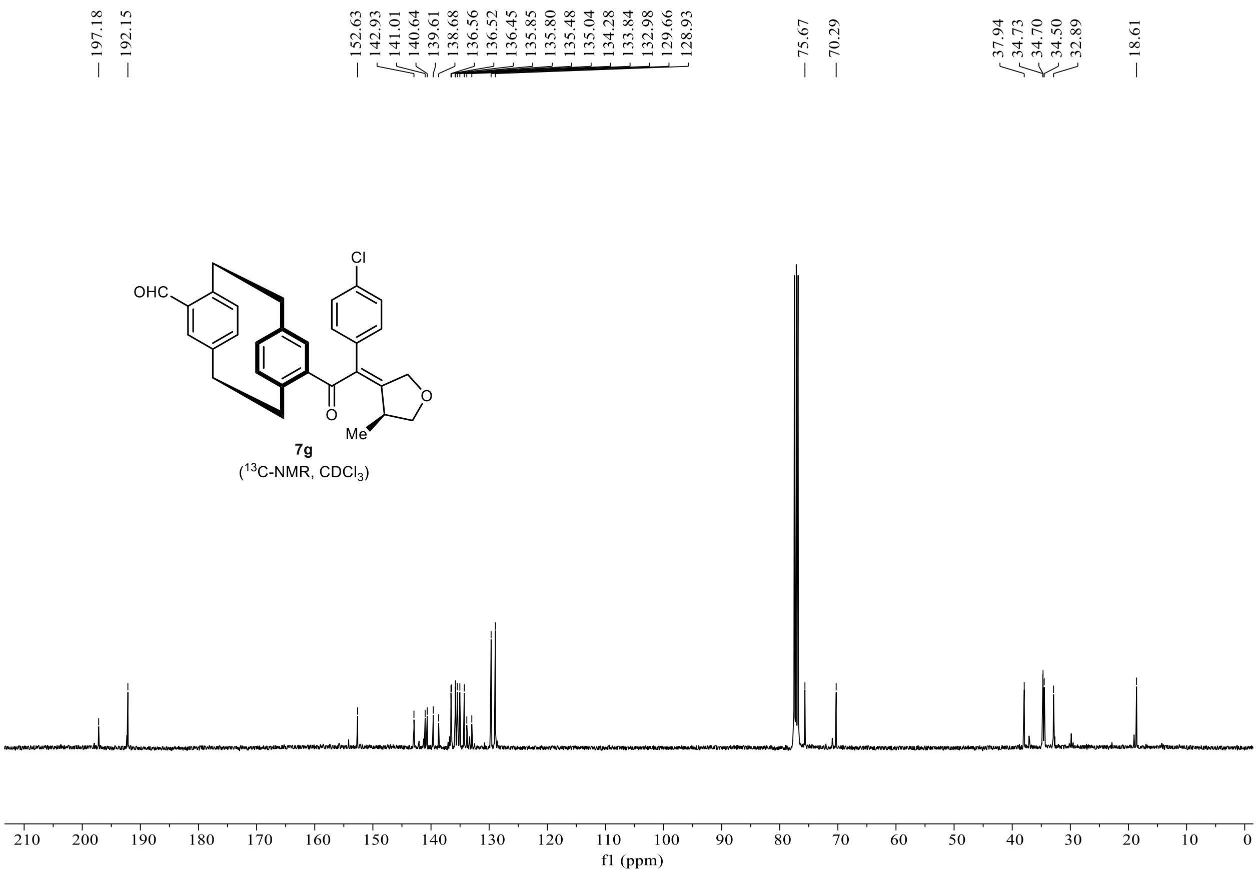
197.18 192.15 152.63 142.93 141.01 140.64 139.61 138.68 136.56 136.52 136.45 135.85 135.80 135.48 135.04 134.28 133.84 132.98 129.66 128.93 75.67 70.29 37.94 34.73 34.70 34.50 32.89 18.61
Cl
OHC
O
O
Me
7g
(^{13}C-NMR, $CDCl_3$)
210 200 190 180 170 160 150 140 130 120 110 100 90 80 70 60 50 40 30 20 10 0
f1 (ppm)

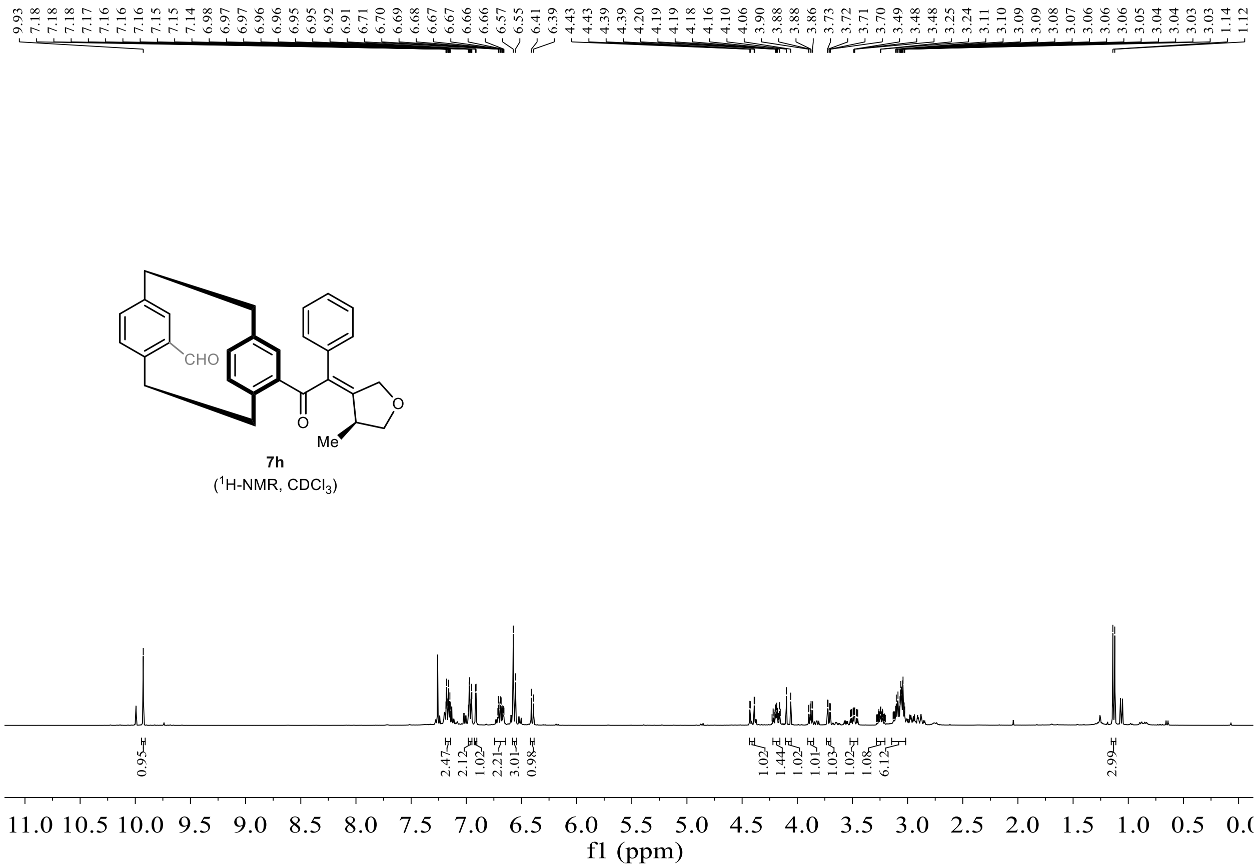
9.93 7.18 7.18 7.17 7.16 7.16 7.16 7.15 7.15 7.14 6.98 6.97 6.97 6.96 6.96 6.96 6.95 6.95 6.92 6.91 6.71 6.70 6.69 6.68 6.67 6.67 6.66 6.66 6.57 6.55 6.41 6.39 4.43 4.39 4.39 4.20 4.19 4.19 4.18 4.16 4.10 4.06 3.90 3.88 3.88 3.86 3.73 3.72 3.71 3.70 3.49 3.48 3.48 3.25 3.24 3.11 3.10 3.09 3.09 3.08 3.07 3.06 3.06 3.05 3.04 3.04 3.03 3.03 1.14 1.12
7h
(1H-NMR, CDCl3)
11.0 10.5 10.0 9.5 9.0 8.5 8.0 7.5 7.0 6.5 6.0 5.5 5.0 4.5 4.0 3.5 3.0 2.5 2.0 1.5 1.0 0.5
f1 (ppm)

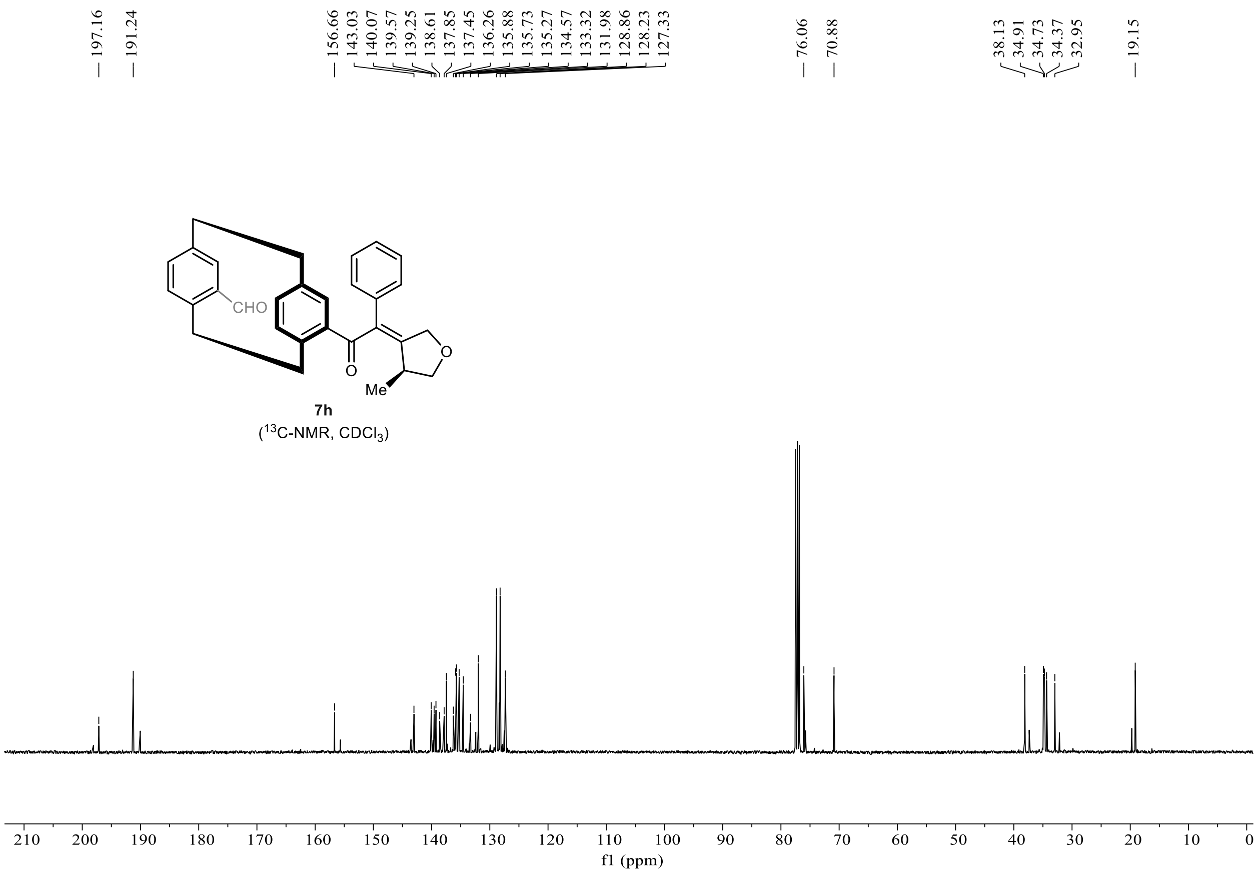
197.16 191.24 156.66 143.03 140.07 139.57 139.25 138.61 137.85 137.45 136.26 135.88 135.73 135.27 134.57 133.32 131.98 128.86 128.23 127.33 76.06 70.88 38.13 34.91 34.73 34.37 32.95 19.15
7h
(13C-NMR, CDCl3)
210 200 190 180 170 160 150 140 130 120 110 100 90 80 70 60 50 40 30 20 10 0
f1 (ppm)

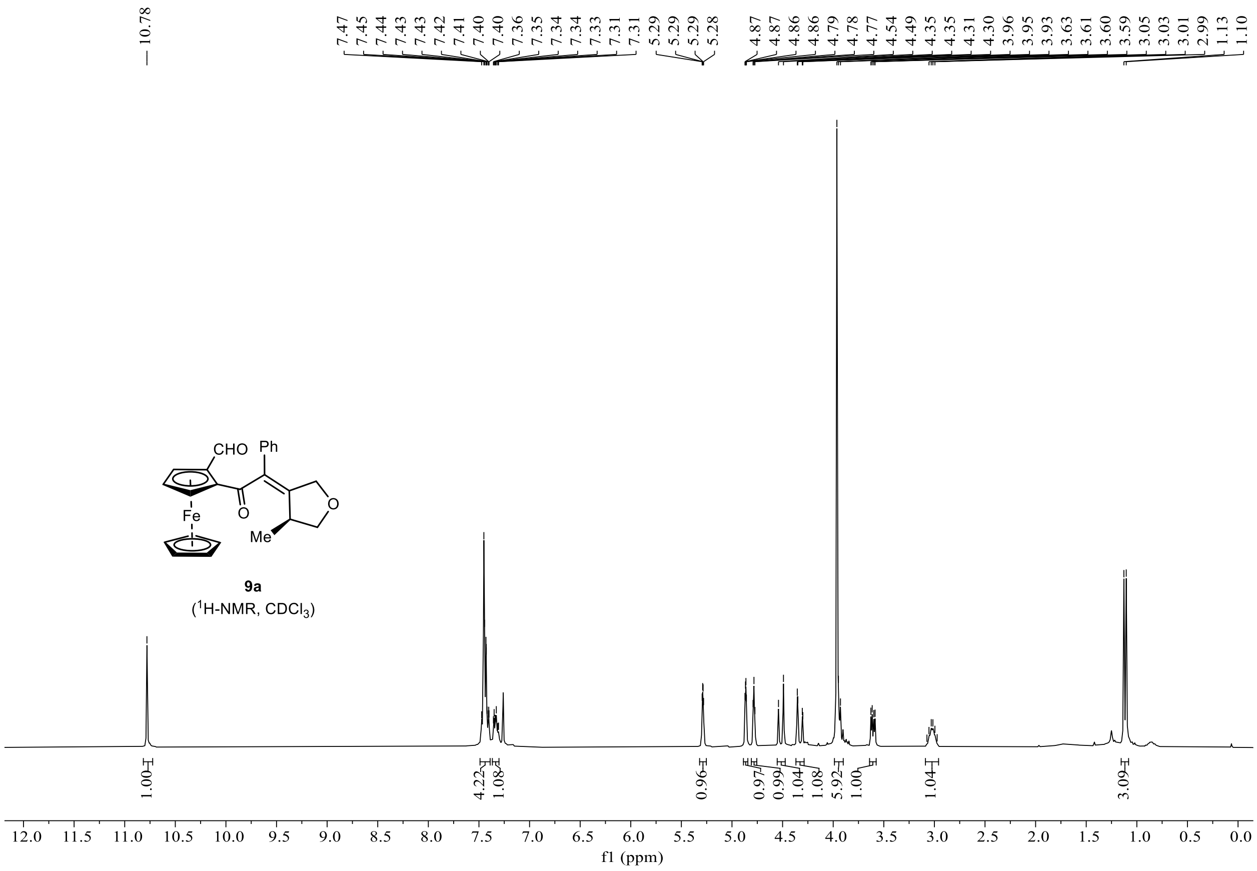
10.78
7.47 7.45 7.44 7.43 7.43 7.42 7.41 7.40 7.40 7.36 7.35 7.34 7.34 7.33 7.31 7.31 5.29 5.29 5.29 5.28 4.87 4.87 4.86 4.86 4.79 4.78 4.77 4.54 4.49 4.35 4.35 4.31 4.30 3.96 3.95 3.93 3.63 3.61 3.60 3.59 3.05 3.03 3.01 2.99 1.13 1.10
CHO
Ph
Fe
O
O
Me
9a
(^{1}H-NMR, $CDCl_3$)
1.00
4.22
1.08
0.96
0.97
0.99
1.04
1.08
5.92
1.00
1.04
3.09
12.0 11.5 11.0 10.5 10.0 9.5 9.0 8.5 8.0 7.5 7.0 6.5 6.0 5.5 5.0 4.5 4.0 3.5 3.0 2.5 2.0 1.5 1.0 0.5 0.0
f1 (ppm)

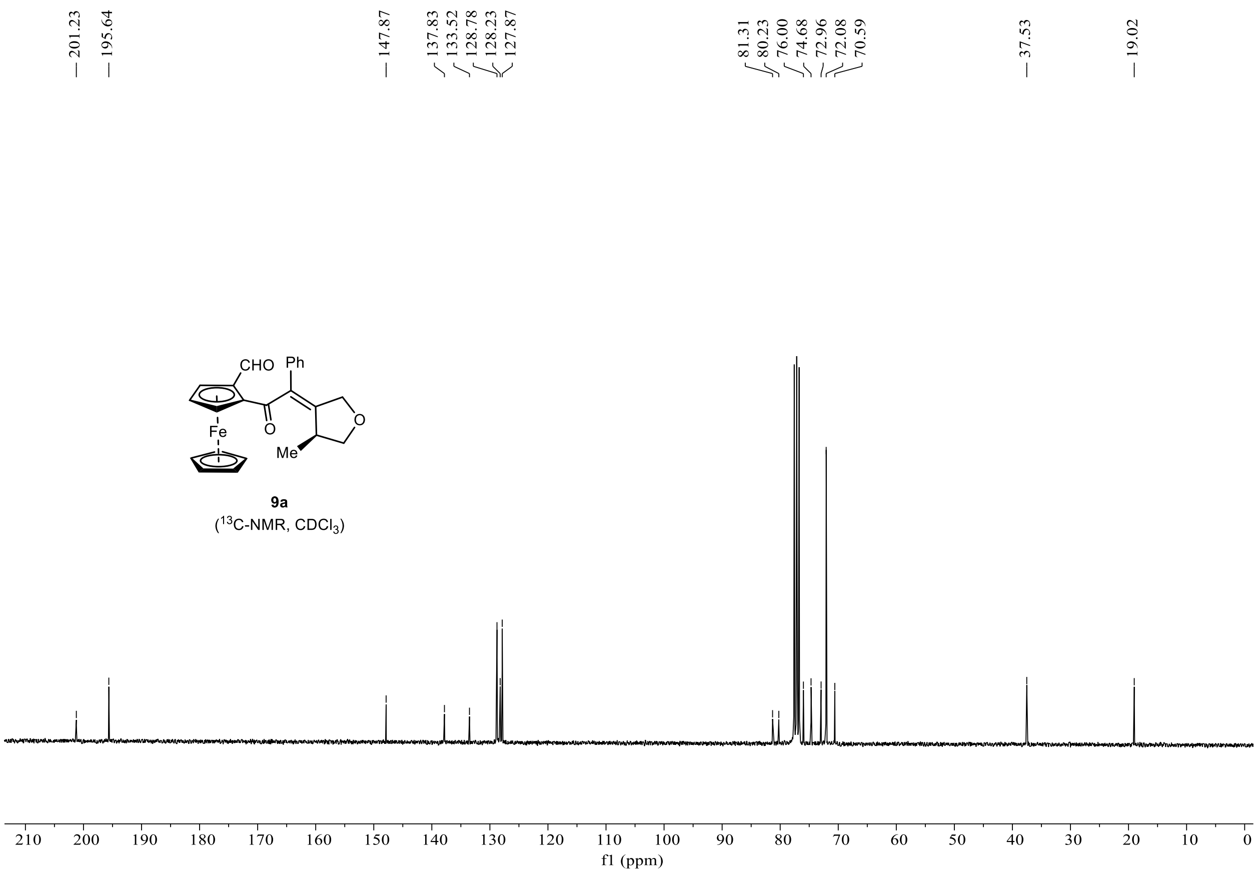
201.23
195.64
147.87
137.83 133.52 128.78 128.23 127.87
81.31 80.23 76.00 74.68 72.96 72.08 70.59
37.53
19.02
CHO
Ph
Fe
O
O
Me
9a
(^{13}C-NMR, $CDCl_3$)
210 200 190 180 170 160 150 140 130 120 110 100 90 80 70 60 50 40 30 20 10 0
f1 (ppm)

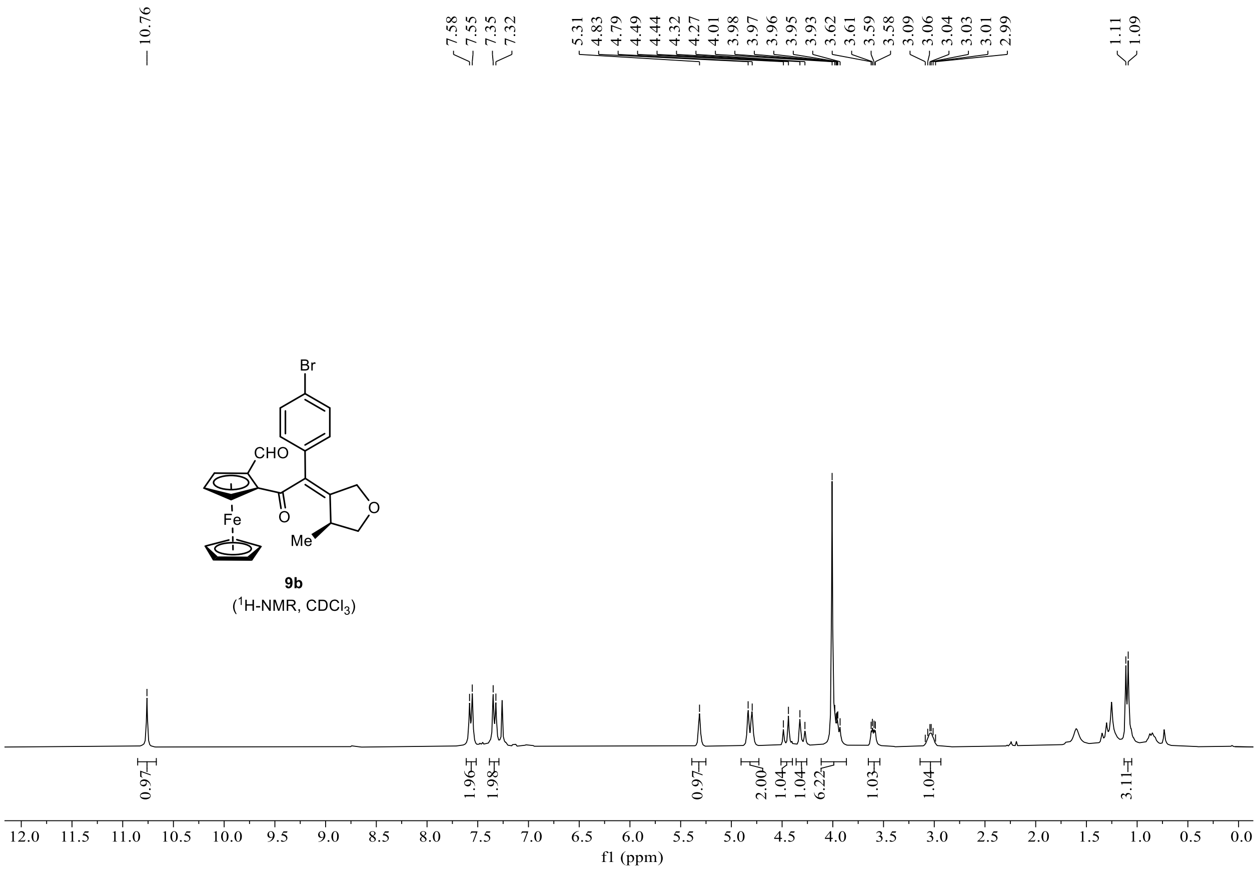
10.76
7.58 7.55 7.35 7.32
5.31 4.83 4.79 4.49 4.44 4.32 4.27 4.01 3.98 3.97 3.96 3.95 3.93 3.62 3.61 3.59 3.58 3.09 3.06 3.04 3.03 3.01 2.99
1.11 1.09
Br
CHO
Fe
O
O
Me
9b
(^{1}H-NMR, $CDCl_3$)
0.97 1.96 1.98 0.97 2.00 1.04 1.04 6.22 1.03 1.04 3.11
12.0 11.5 11.0 10.5 10.0 9.5 9.0 8.5 8.0 7.5 7.0 6.5 6.0 5.5 5.0 4.5 4.0 3.5 3.0 2.5 2.0 1.5 1.0 0.5 0.0
f1 (ppm)

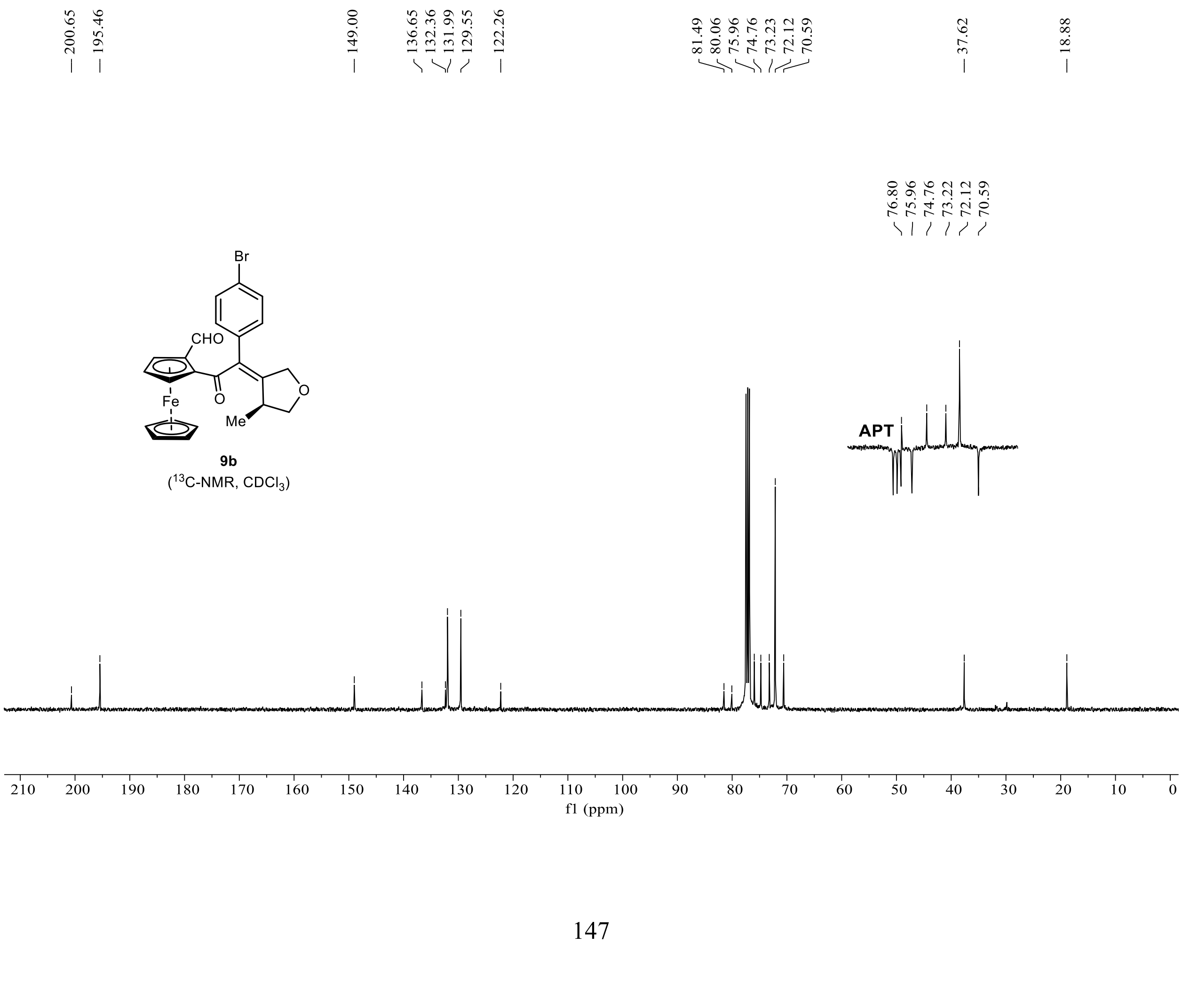
200.65 195.46
149.00
136.65 132.36 131.99 129.55 122.26
81.49 80.06 75.96 74.76 73.23 72.12 70.59
37.62
18.88
76.80 75.96 74.76 73.22 72.12 70.59
APT
Br
CHO
Fe
O
O
Me
9b
(^{13}C-NMR, $CDCl_3$)
210 200 190 180 170 160 150 140 130 120 110 100 90 80 70 60 50 40 30 20 10 0
f1 (ppm)

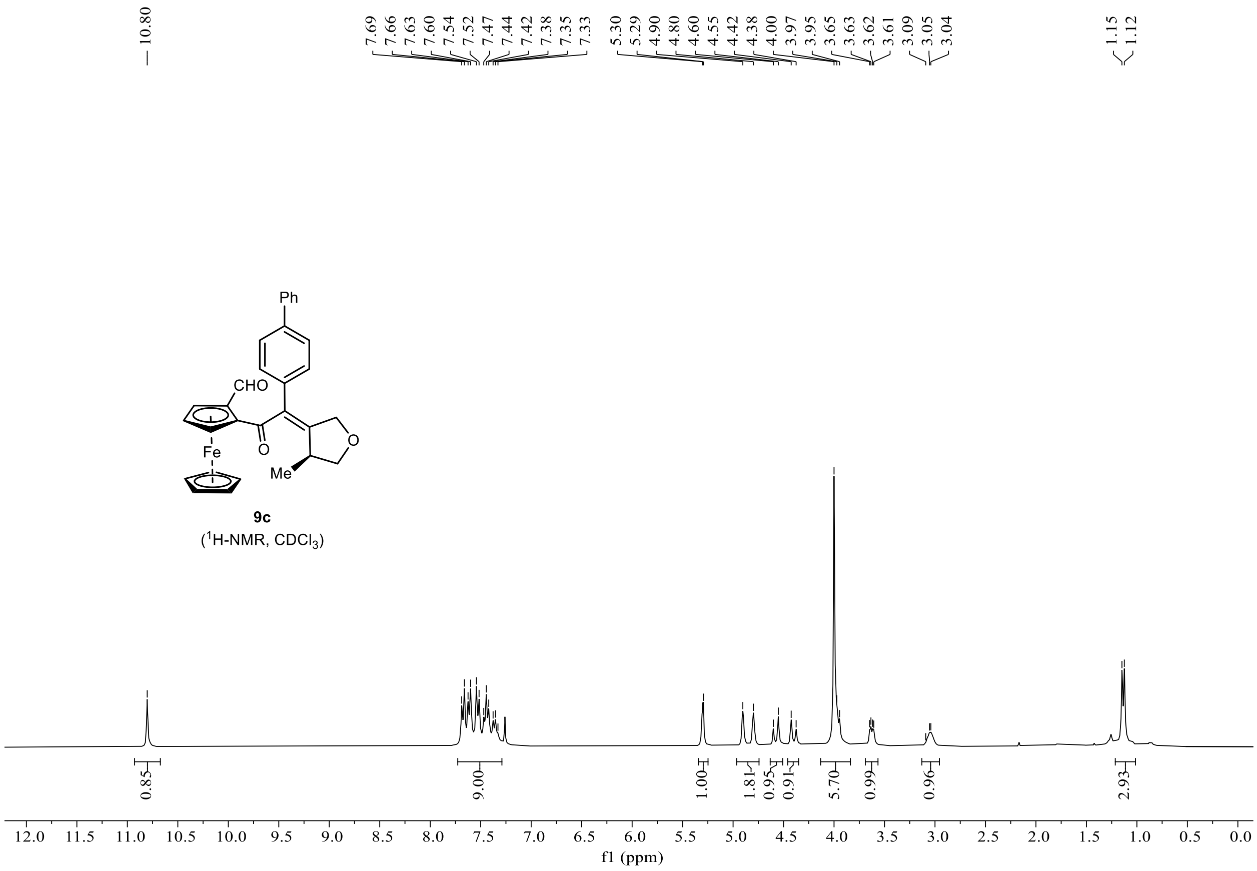
10.80
7.69 7.66 7.63 7.60 7.54 7.52 7.47 7.44 7.42 7.38 7.35 7.33
5.30 5.29 4.90 4.80 4.60 4.55 4.42 4.38 4.00 3.97 3.95 3.65 3.63 3.62 3.61 3.09 3.05 3.04
1.15 1.12
Ph
CHO
Fe
O
O
Me
9c
(1H-NMR, CDCl3)
0.85
9.00
1.00
1.81
0.95
0.91
5.70
0.99
0.96
2.93
f1 (ppm)

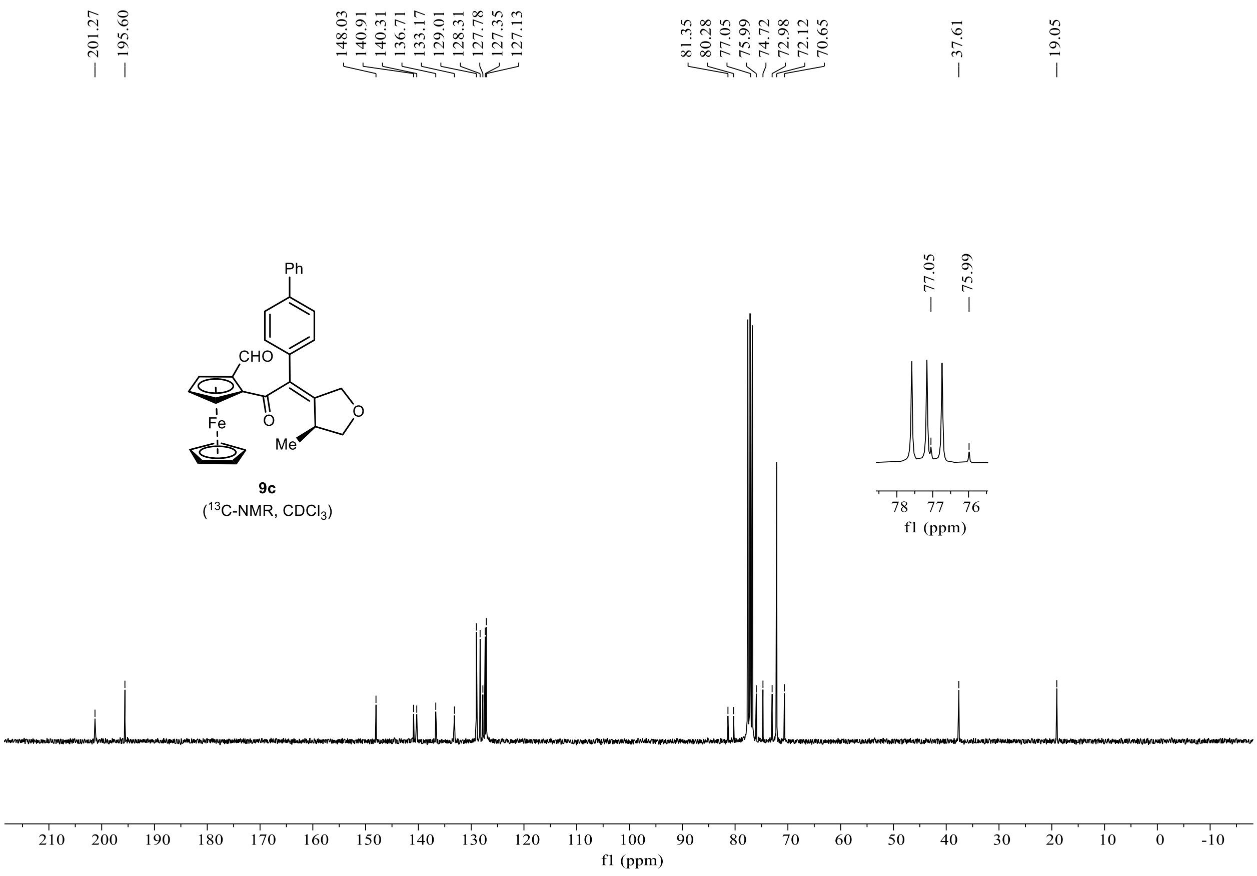
201.27
195.60
148.03 140.91 140.31 136.71 133.17 129.01 128.31 127.78 127.35 127.13
81.35 80.28 77.05 75.99 74.72 72.98 72.12 70.65
37.61
19.05
Ph
CHO
Fe
O
O
Me
9c
(13C-NMR, CDCl3)
77.05
75.99
f1 (ppm)
f1 (ppm)

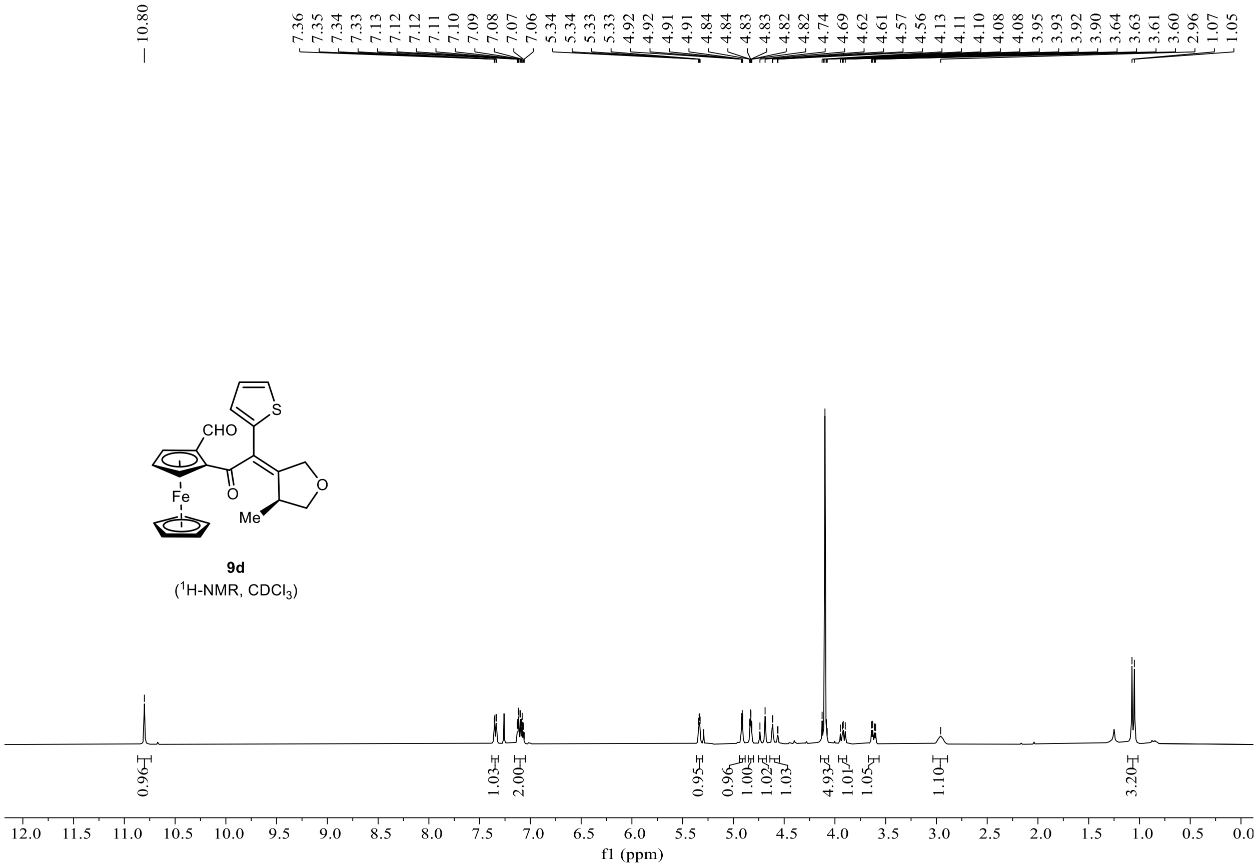

9d
(^{1}H-NMR, $CDCl_3$)

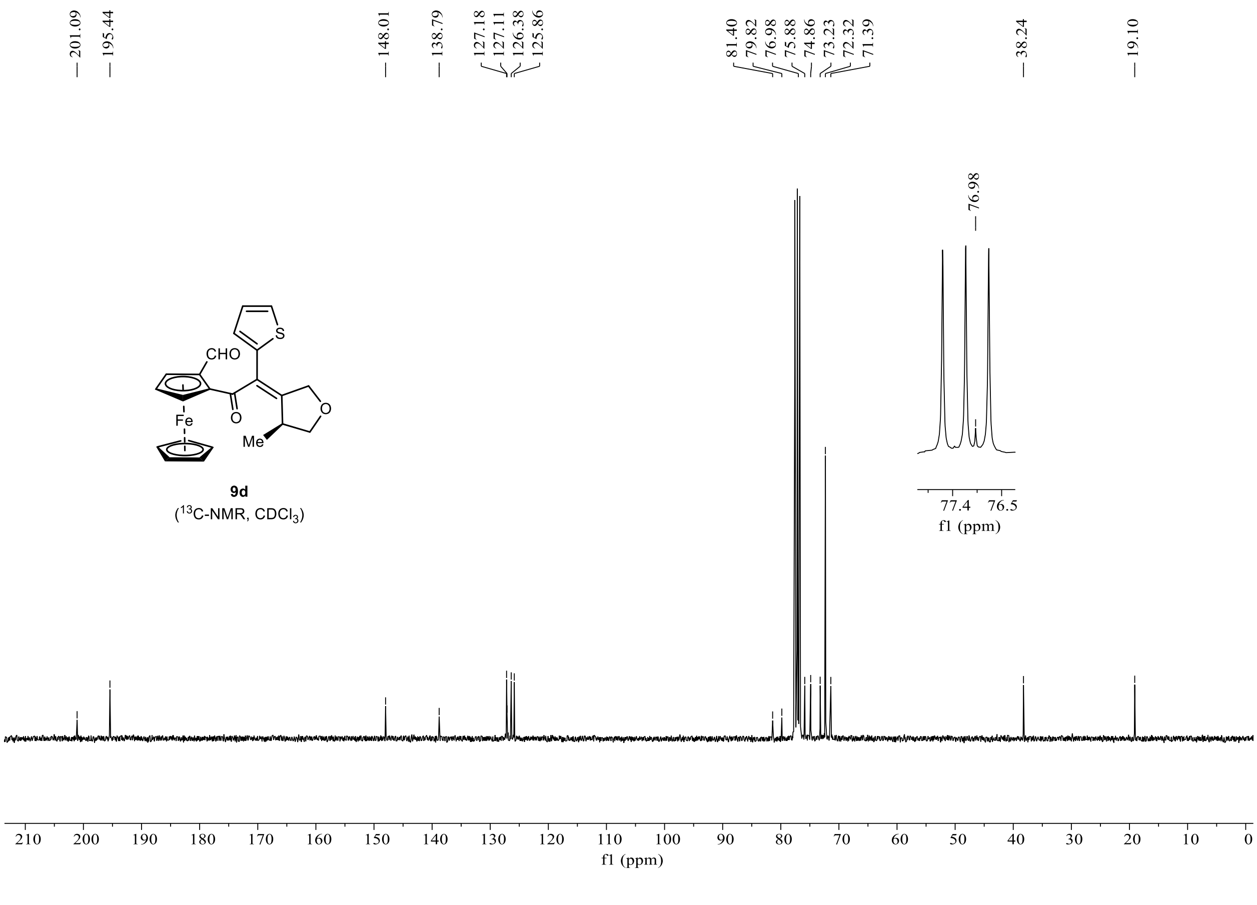

9d
(^{13}C-NMR, $CDCl_3$)

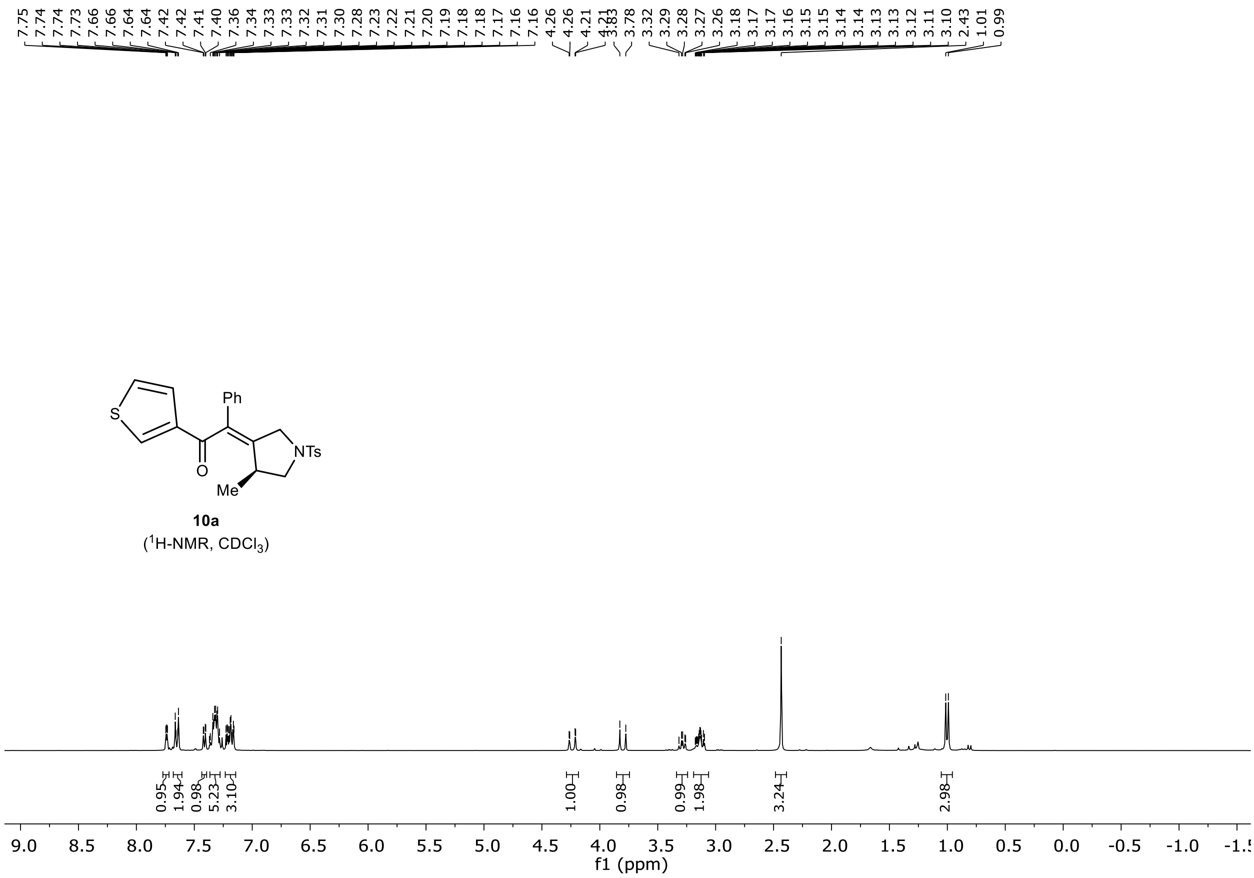

10a
(^{1}H-NMR, $CDCl_3$)

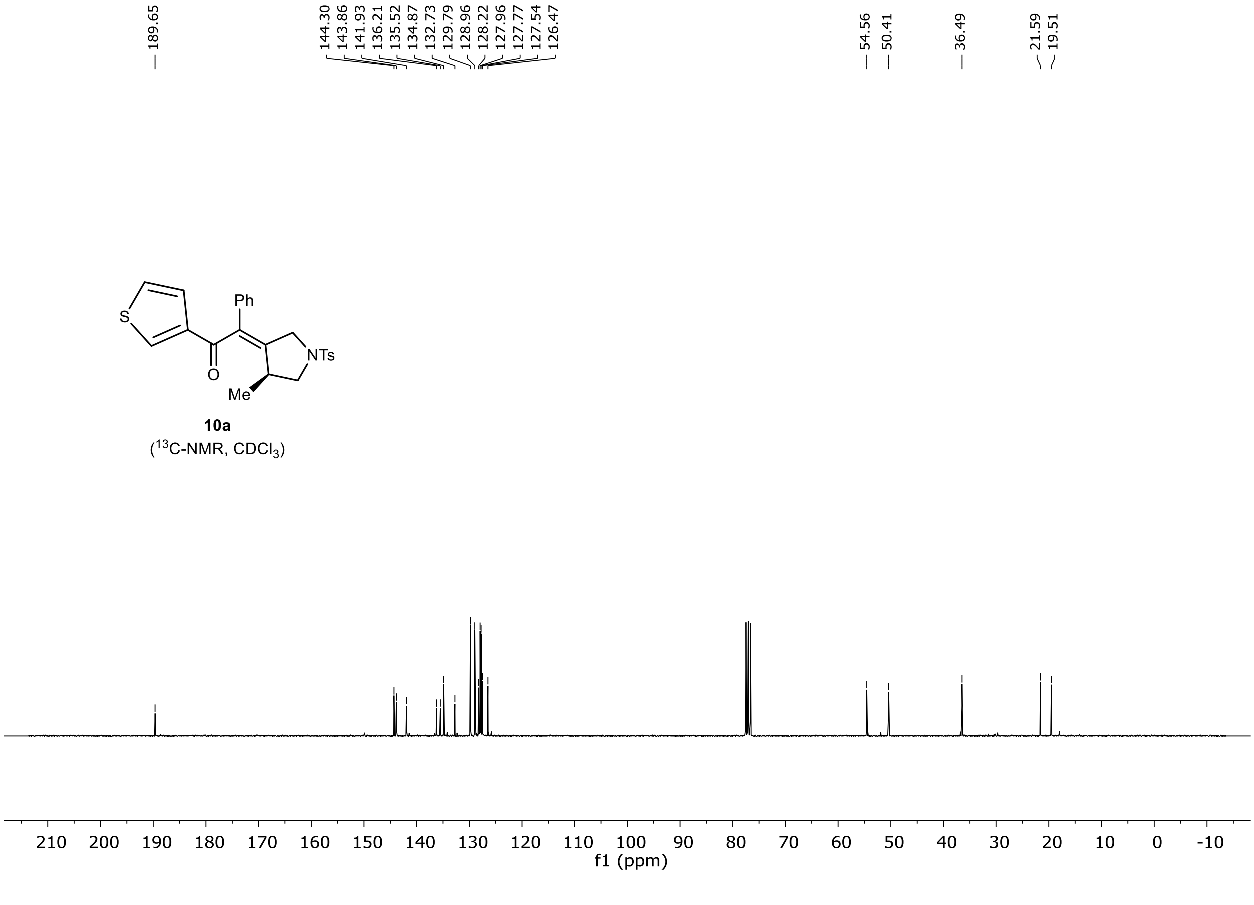

10a
(^{13}C-NMR, $CDCl_3$)

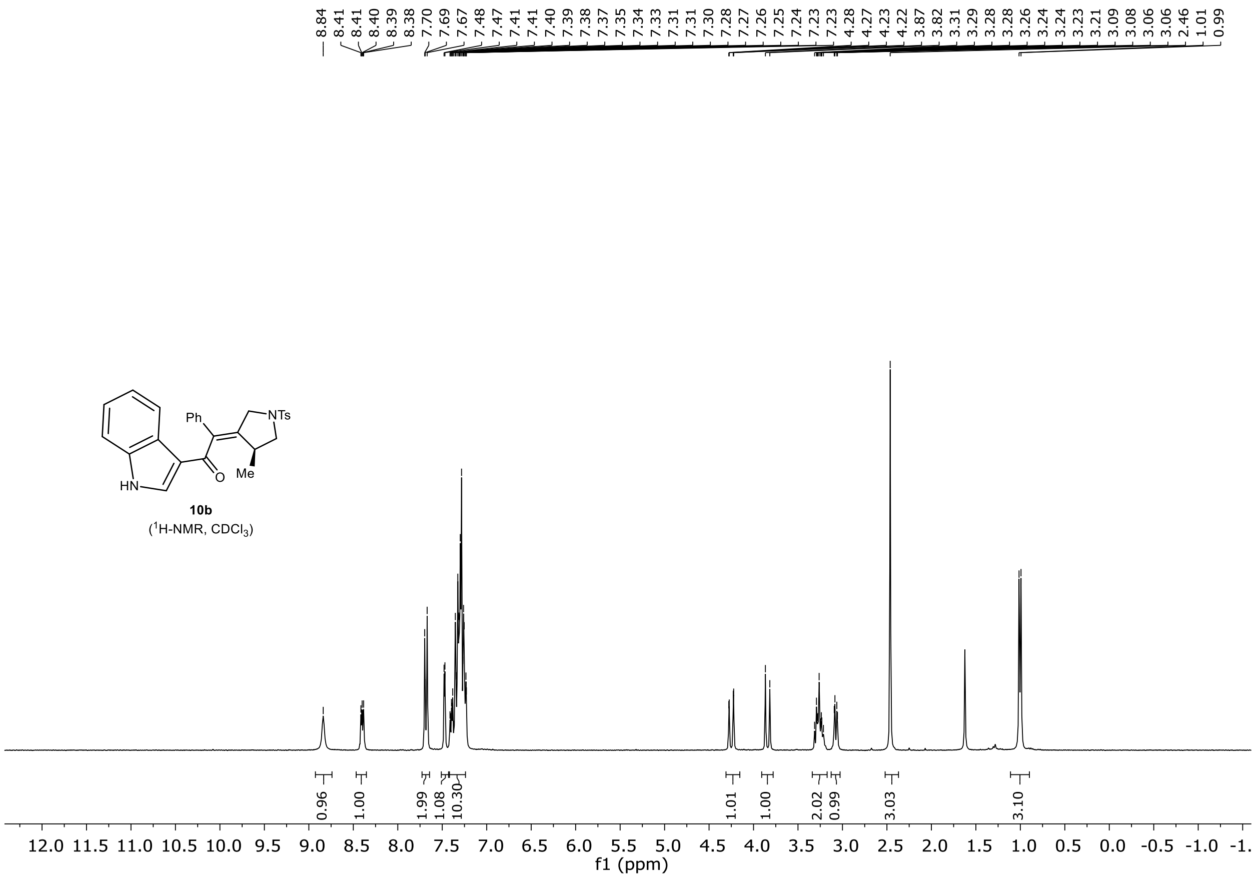
8.84 8.41 8.41 8.40 8.39 8.38 7.70 7.69 7.67 7.48 7.47 7.41 7.41 7.40 7.39 7.38 7.37 7.35 7.34 7.33 7.31 7.31 7.30 7.28 7.27 7.26 7.25 7.24 7.23 7.23 4.28 4.27 4.23 4.22 3.87 3.82 3.31 3.29 3.28 3.28 3.26 3.24 3.24 3.23 3.21 3.09 3.08 3.06 3.06 2.46 1.01 0.99
Ph
NTs
O
Me
HN
10b
(1H-NMR, CDCl3)
0.96 1.00 1.99 1.08 10.30 1.01 1.00 2.02 0.99 3.03 3.10
f1 (ppm)

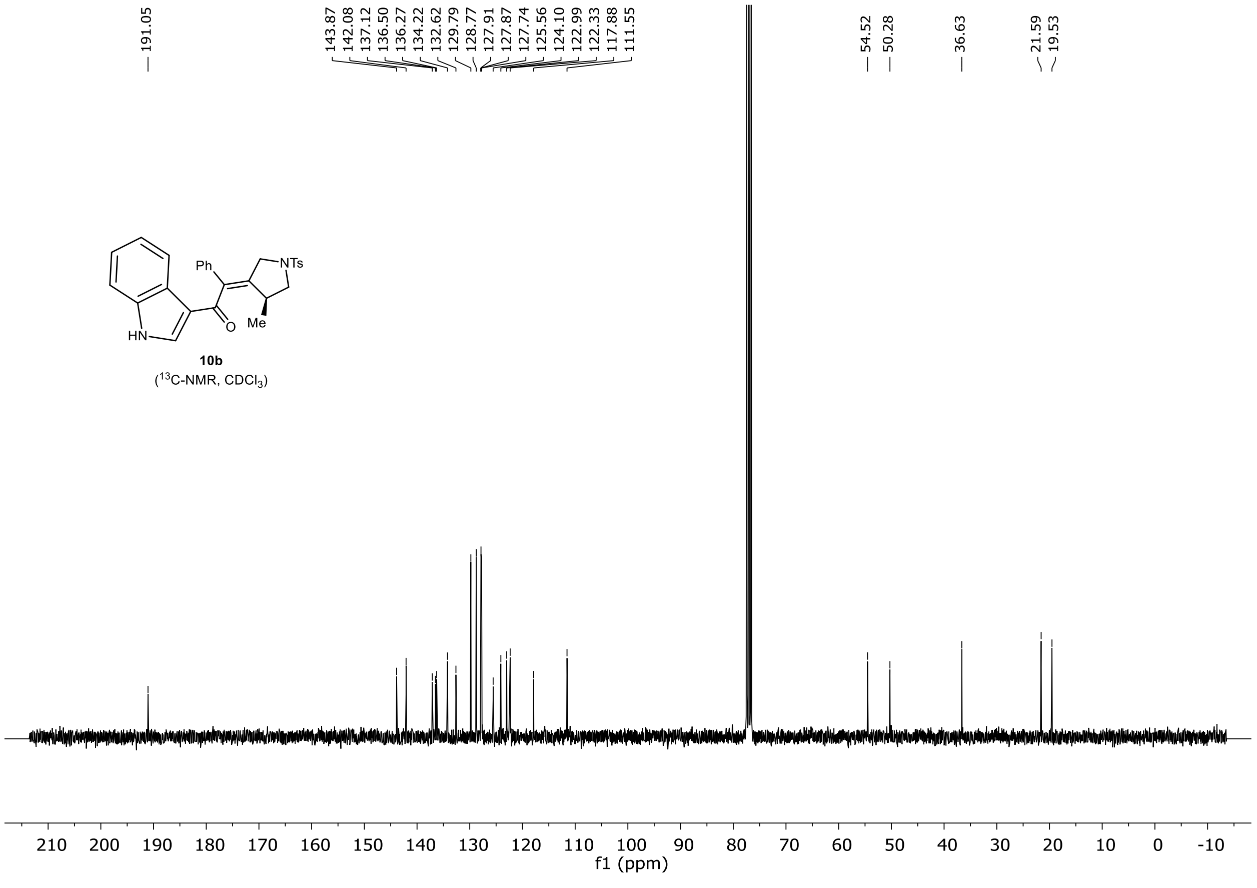
191.05
143.87 142.08 137.12 136.50 136.27 134.22 132.62 129.79 128.77 127.91 127.87 127.74 125.56 124.10 122.99 122.33 117.88 111.55
54.52 50.28 36.63 21.59 19.53
Ph
NTs
O
Me
HN
10b
(13C-NMR, CDCl3)
f1 (ppm)

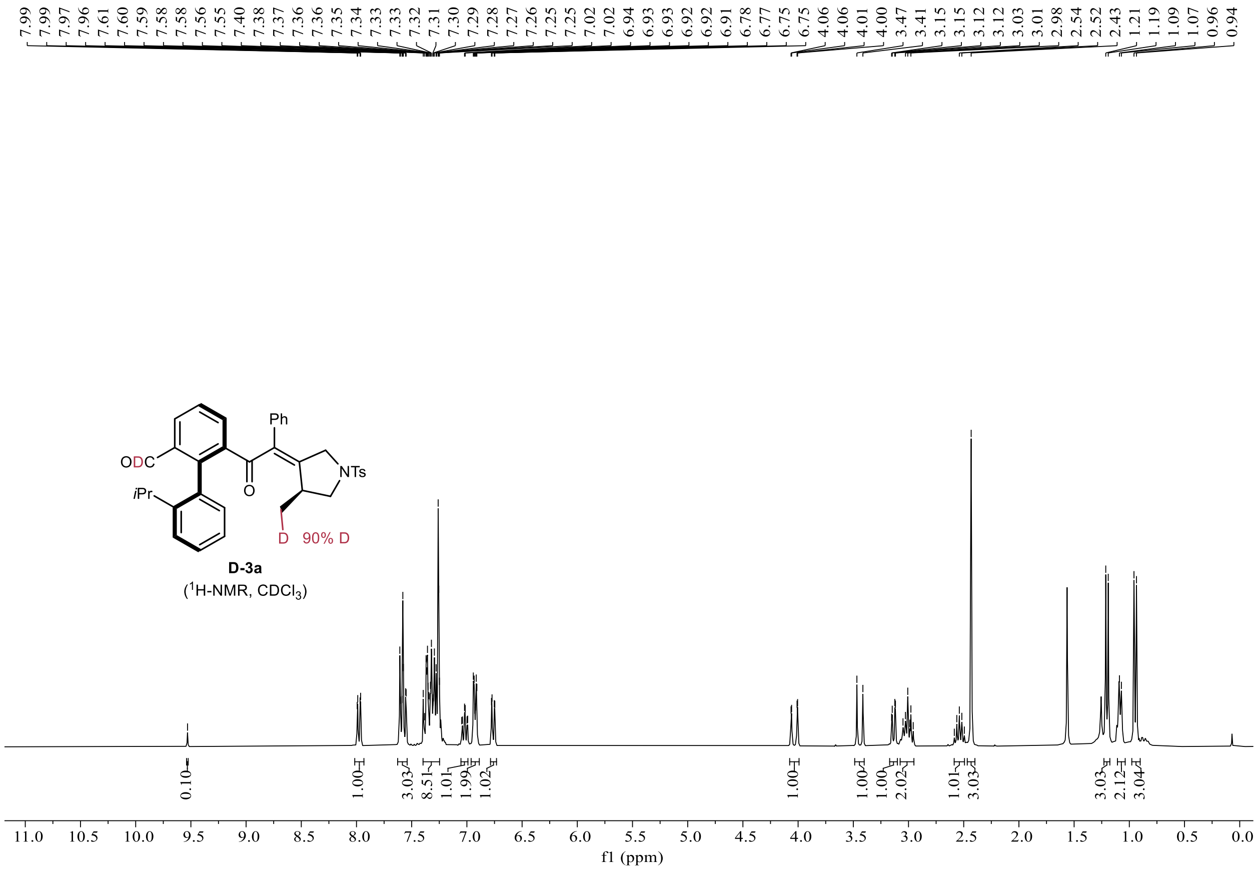
7.99 7.99 7.97 7.96 7.61 7.60 7.59 7.58 7.58 7.56 7.55 7.40 7.38 7.37 7.36 7.36 7.35 7.34 7.33 7.33 7.32 7.31 7.30 7.29 7.28 7.27 7.26 7.25 7.25 7.02 7.02 6.94 6.93 6.93 6.92 6.91 6.78 6.77 6.75 6.75 4.06 4.01 4.00 3.47 3.41 3.15 3.15 3.12 3.12 3.03 3.01 2.98 2.54 2.52 2.43 1.21 1.19 1.09 1.07 0.96 0.94
Ph
ODC
NTs
O
iPr
D 90% D
D-3a
(1H-NMR, CDCl3)
0.10 1.00 3.03 8.51 1.01 1.99 1.02 1.00 1.00 1.00 2.02 1.01 3.03 3.03 2.12 3.04
11.0 10.5 10.0 9.5 9.0 8.5 8.0 7.5 7.0 6.5 6.0 5.5 5.0 4.5 4.0 3.5 3.0 2.5 2.0 1.5 1.0 0.5 0.0
f1 (ppm)

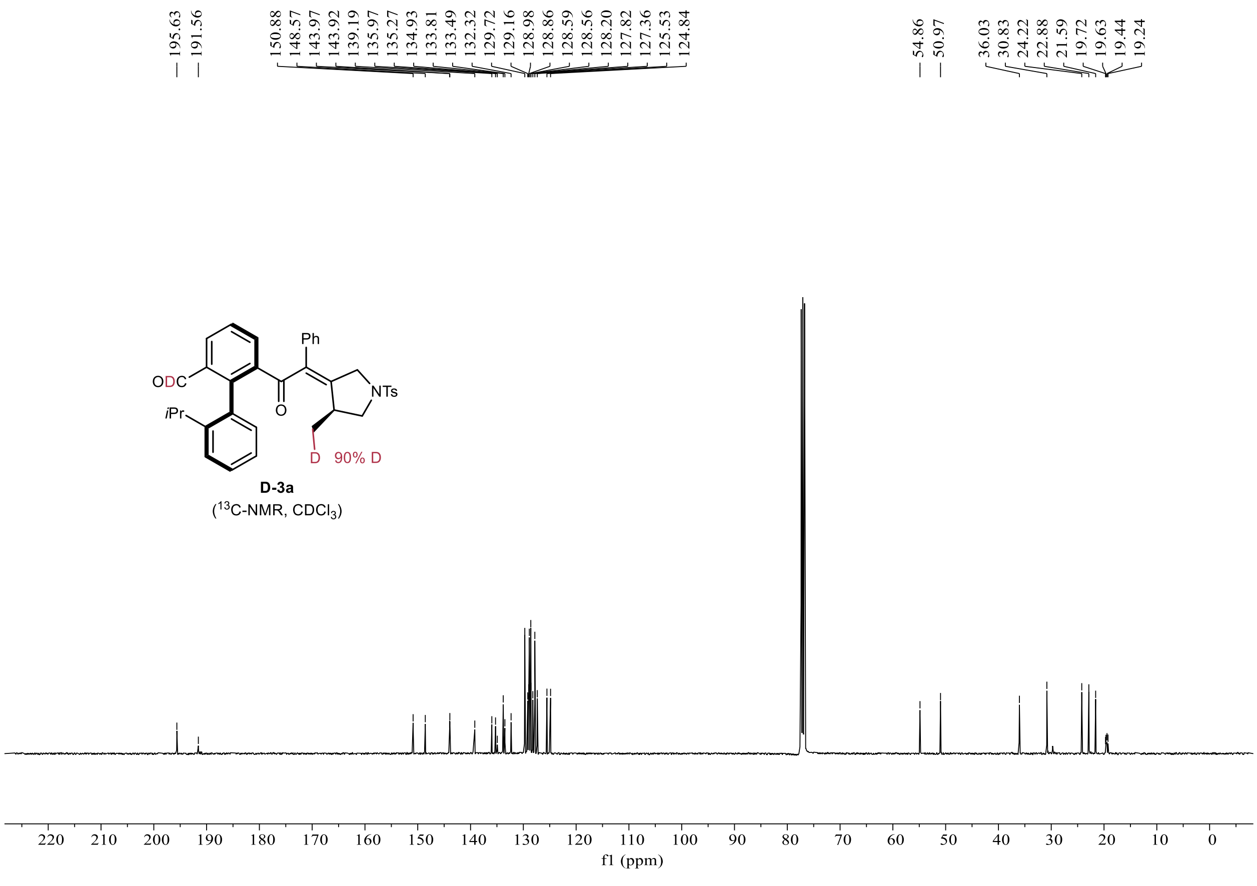
195.63 191.56 150.88 148.57 143.97 143.92 139.19 135.97 135.27 134.93 133.81 133.49 132.32 129.72 129.16 128.98 128.86 128.59 128.56 128.20 127.82 127.36 125.53 124.84 54.86 50.97 36.03 30.83 24.22 22.88 21.59 19.72 19.63 19.44 19.24
Ph
ODC
NTs
O
iPr
D 90% D
D-3a
(13C-NMR, CDCl3)
220 210 200 190 180 170 160 150 140 130 120 110 100 90 80 70 60 50 40 30 20 10 0
f1 (ppm)

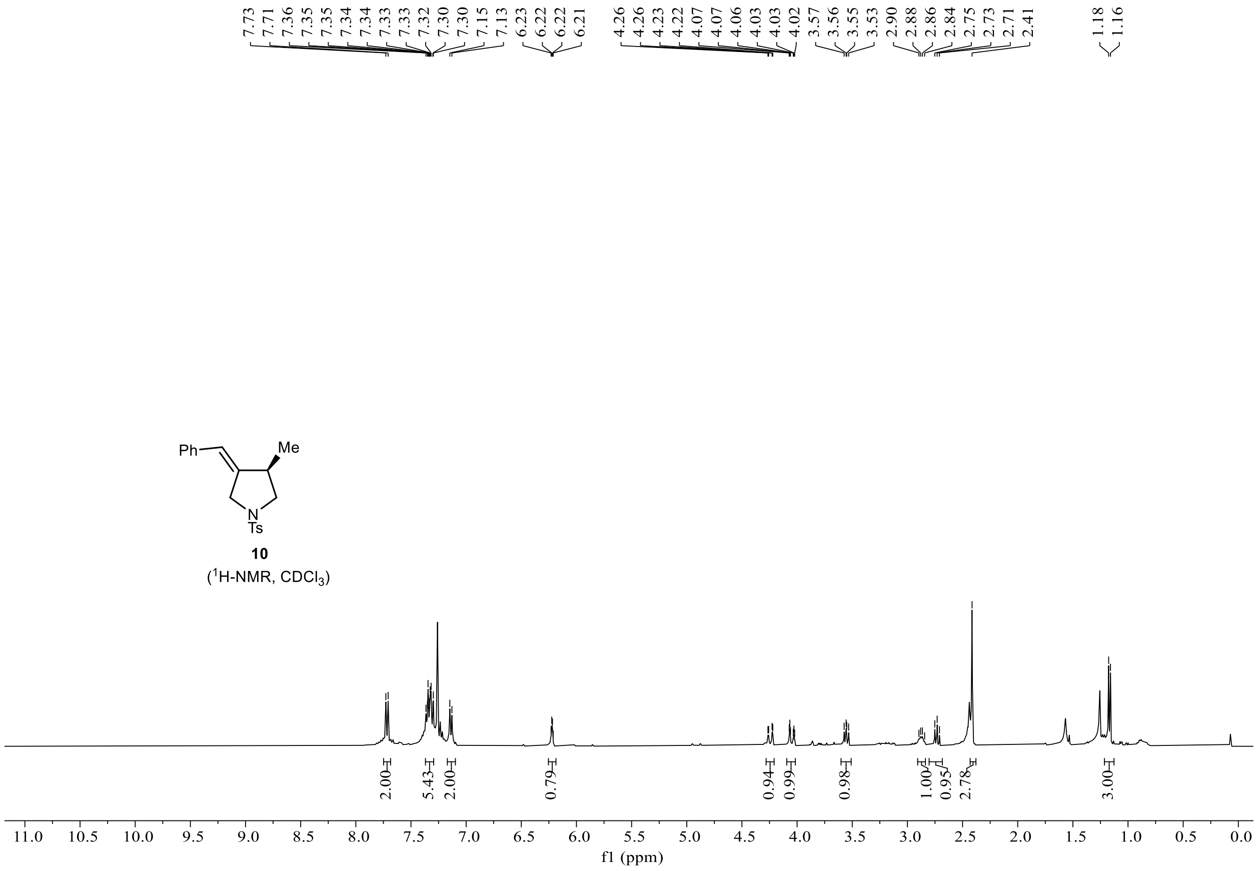
7.73 7.71 7.36 7.35 7.35 7.34 7.34 7.33 7.33 7.32 7.30 7.30 7.15 7.13 6.23 6.22 6.22 6.21 4.26 4.26 4.23 4.22 4.07 4.07 4.06 4.03 4.03 4.02 3.57 3.56 3.55 3.53 2.90 2.88 2.86 2.84 2.75 2.73 2.71 2.41 1.18 1.16
Ph
Me
N
Ts
10
(^{1}H-NMR, CDCl$_3$)
2.00 5.43 2.00 0.79 0.94 0.99 0.98 1.00 0.95 2.78 3.00
11.0 10.5 10.0 9.5 9.0 8.5 8.0 7.5 7.0 6.5 6.0 5.5 5.0 4.5 4.0 3.5 3.0 2.5 2.0 1.5 1.0 0.5 0.0
f1 (ppm)

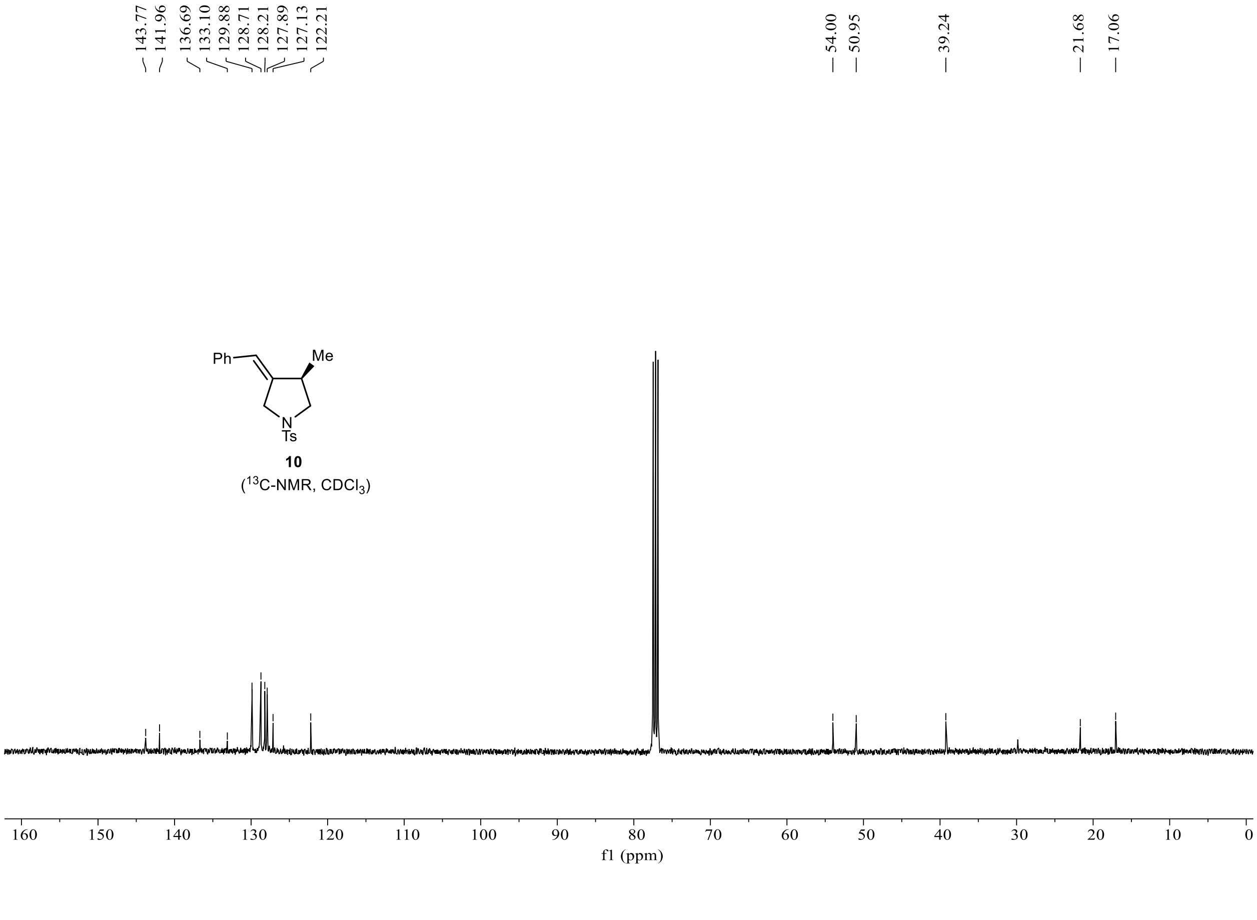
143.77 141.96 136.69 133.10 129.88 128.71 128.21 127.89 127.13 122.21 54.00 50.95 39.24 21.68 17.06
Ph
Me
N
Ts
10
(^{13}C-NMR, CDCl$_3$)
160 150 140 130 120 110 100 90 80 70 60 50 40 30 20 10 0
f1 (ppm)

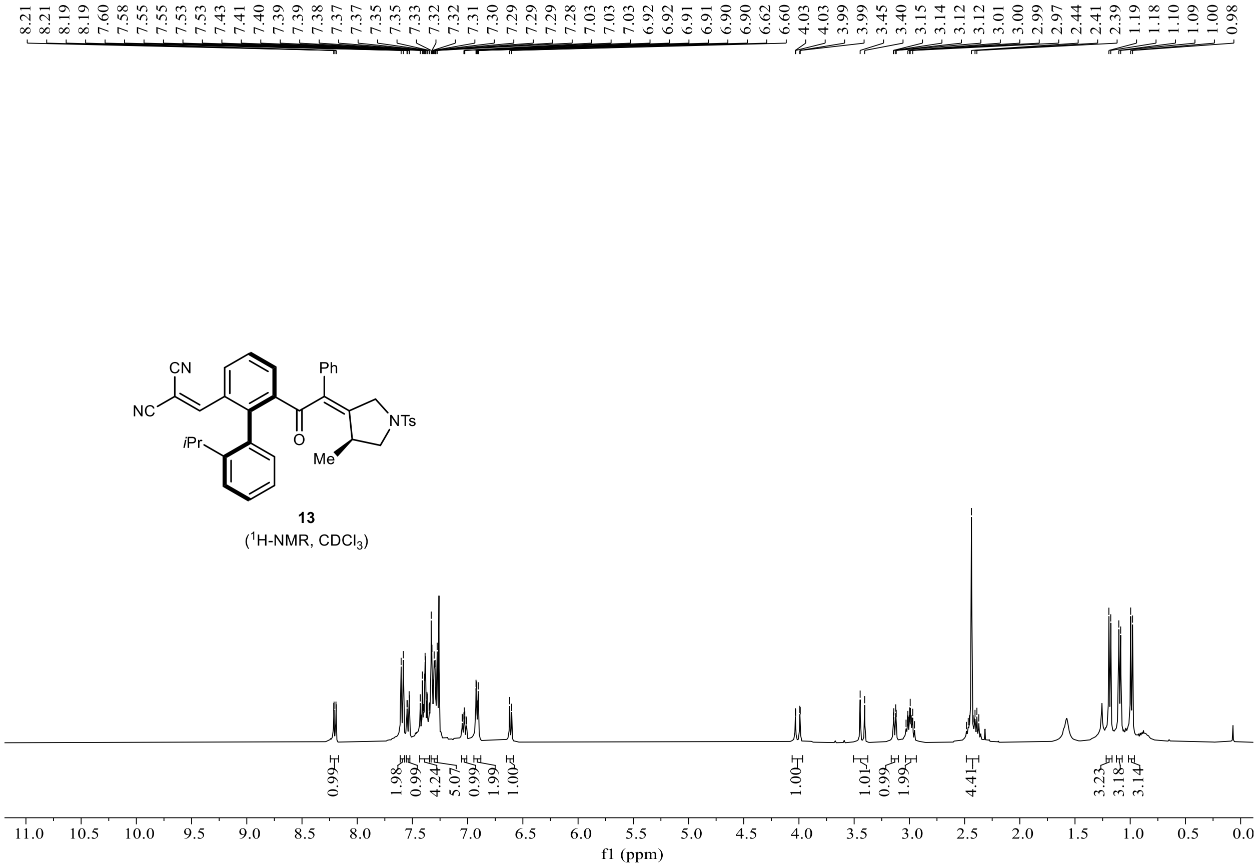
8.21 8.21 8.19 8.19 7.60 7.58 7.55 7.55 7.53 7.53 7.43 7.41 7.40 7.39 7.39 7.38 7.37 7.37 7.35 7.35 7.33 7.32 7.32 7.31 7.30 7.29 7.29 7.29 7.28 7.03 7.03 7.03 6.92 6.92 6.91 6.91 6.90 6.90 6.62 6.60 4.03 4.03 3.99 3.45 3.40 3.15 3.14 3.12 3.12 3.01 3.00 2.99 2.97 2.44 2.41 2.39 1.19 1.18 1.10 1.09 1.00 0.98
CN
NC
Ph
O
NTs
iPr
Me
13
(1H-NMR, CDCl3)
0.99 1.98 0.99 4.24 5.07 0.99 1.99 1.00 1.00 1.01 0.99 1.99 4.41 3.23 3.18 3.14
11.0 10.5 10.0 9.5 9.0 8.5 8.0 7.5 7.0 6.5 6.0 5.5 5.0 4.5 4.0 3.5 3.0 2.5 2.0 1.5 1.0 0.5 0.0
f1 (ppm)

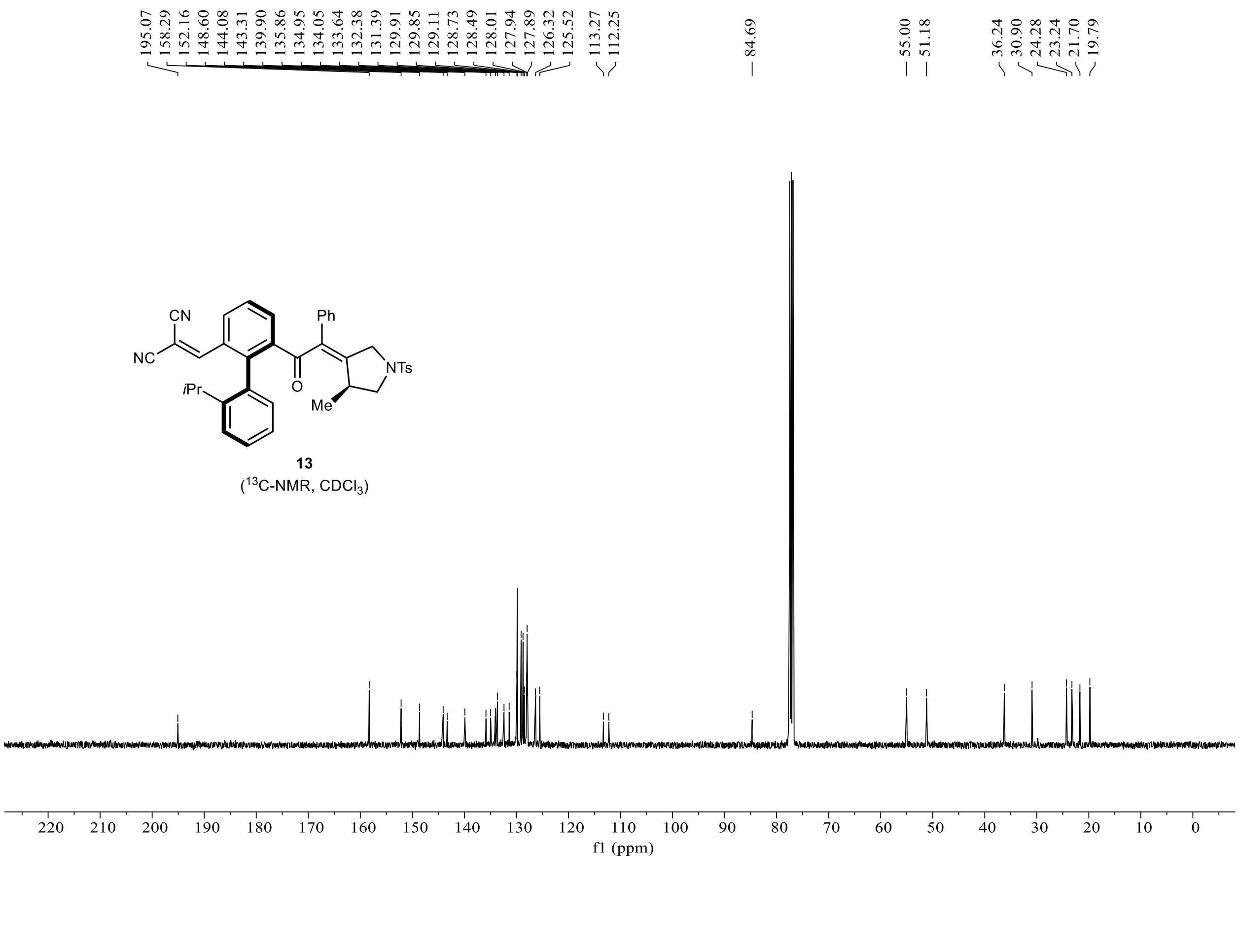
195.07 158.29 152.16 148.60 144.08 143.31 139.90 135.86 134.95 134.05 133.64 132.38 131.39 129.91 129.85 129.11 128.73 128.49 128.01 127.94 127.89 126.32 125.52 113.27 112.25 84.69 55.00 51.18 36.24 30.90 24.28 23.24 21.70 19.79
CN
NC
Ph
O
NTs
iPr
Me
13
(13C-NMR, CDCl3)
220 210 200 190 180 170 160 150 140 130 120 110 100 90 80 70 60 50 40 30 20 10 0
f1 (ppm)

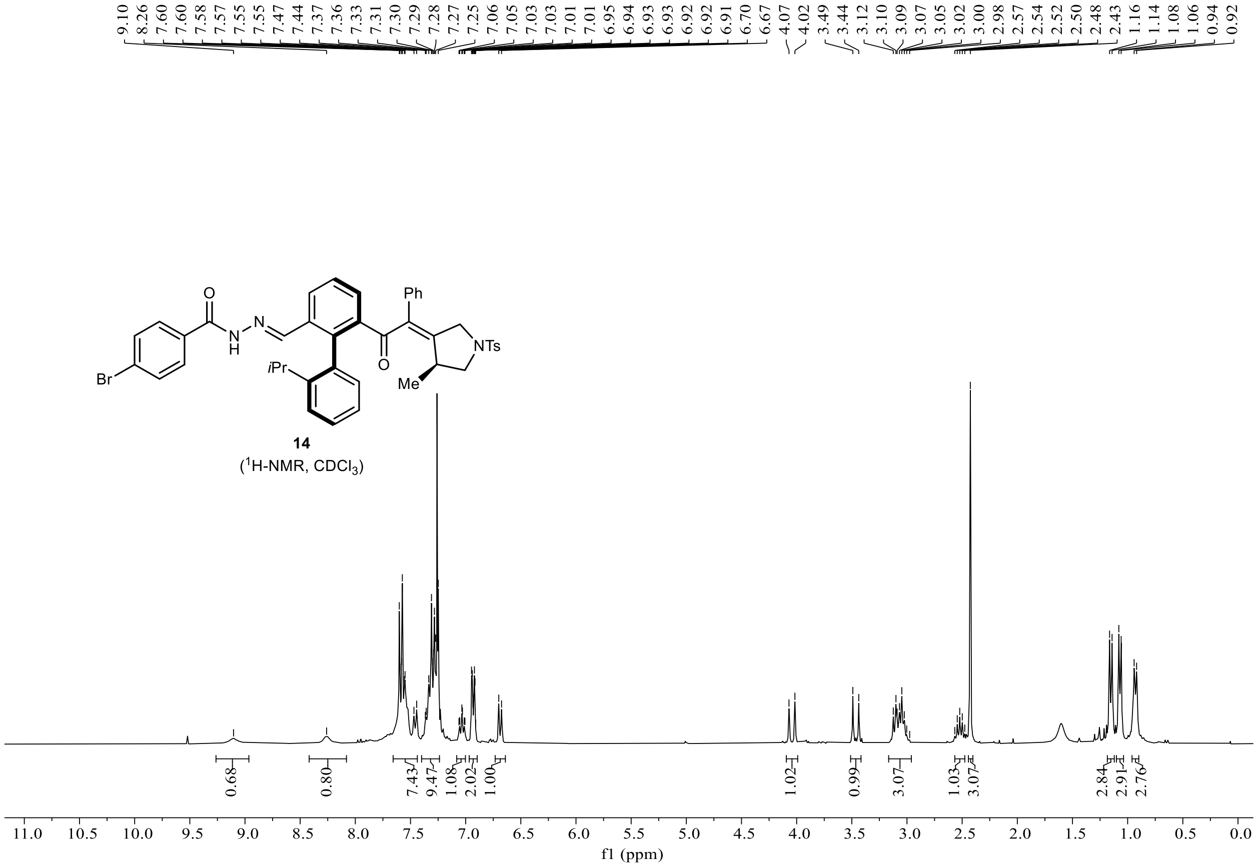
9.10 8.26 7.60 7.58 7.57 7.55 7.55 7.47 7.44 7.37 7.36 7.33 7.31 7.30 7.29 7.28 7.27 7.25 7.06 7.05 7.03 7.03 7.01 7.01 6.95 6.94 6.93 6.93 6.92 6.92 6.91 6.70 6.67 4.07 4.02 3.49 3.44 3.12 3.10 3.09 3.07 3.05 3.02 3.00 2.98 2.57 2.54 2.52 2.50 2.48 2.43 1.16 1.14 1.08 1.06 0.94 0.92
O
N
N
H
Br
iPr
Ph
O
NTs
Me
14
(1H-NMR, CDCl3)
0.68 0.80 7.43 9.47 1.08 2.02 1.00 1.02 0.99 3.07 1.03 3.07 2.84 2.91 2.76
11.0 10.5 10.0 9.5 9.0 8.5 8.0 7.5 7.0 6.5 6.0 5.5 5.0 4.5 4.0 3.5 3.0 2.5 2.0 1.5 1.0 0.5 0.0
f1 (ppm)

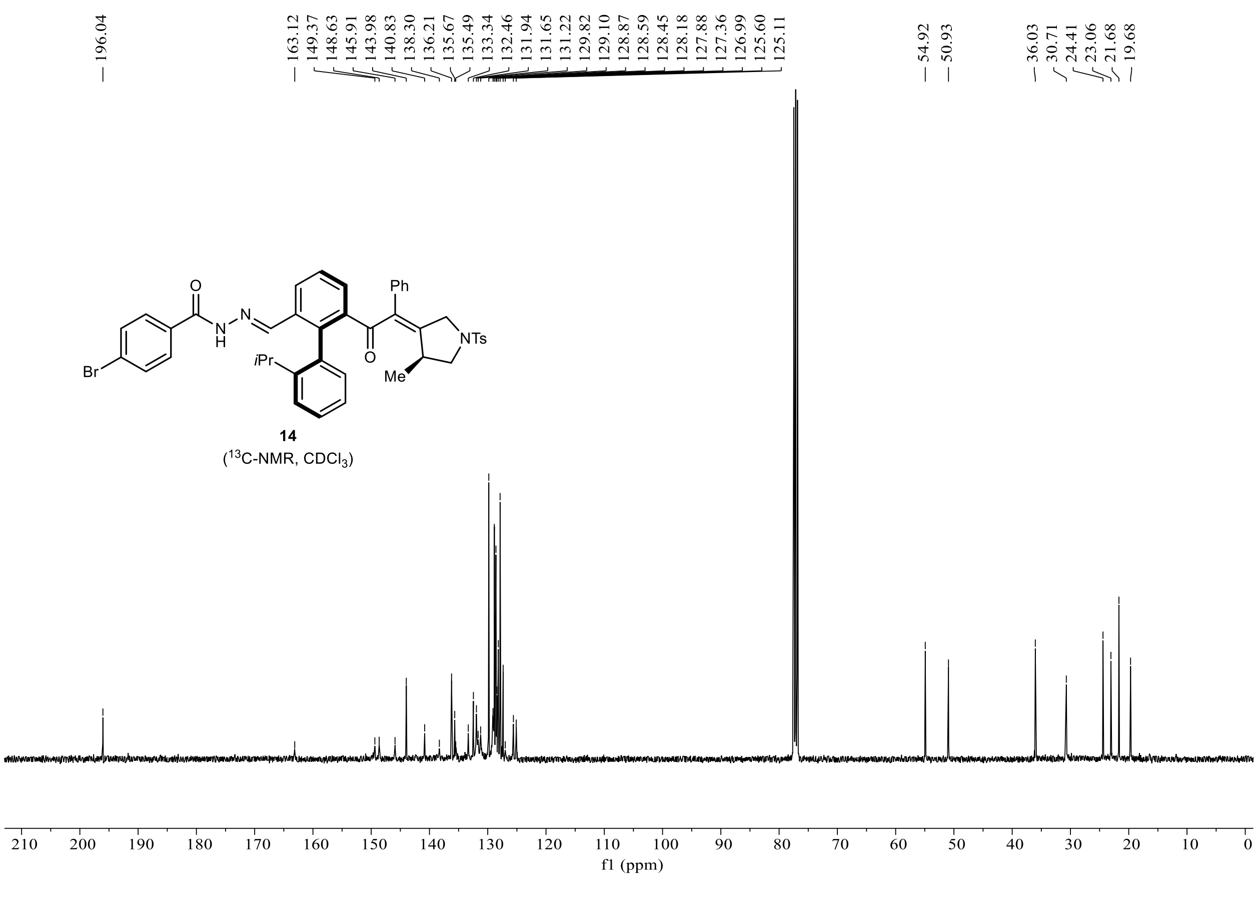
196.04 163.12 149.37 148.63 145.91 143.98 140.83 138.30 136.21 135.67 135.49 133.34 132.46 131.94 131.65 131.22 129.82 129.10 128.87 128.59 128.45 128.18 127.88 127.36 126.99 125.60 125.11 54.92 50.93 36.03 30.71 24.41 23.06 21.68 19.68
O
N
N
H
Br
iPr
Ph
O
NTs
Me
14
(13C-NMR, CDCl3)
210 200 190 180 170 160 150 140 130 120 110 100 90 80 70 60 50 40 30 20 10 0
f1 (ppm)

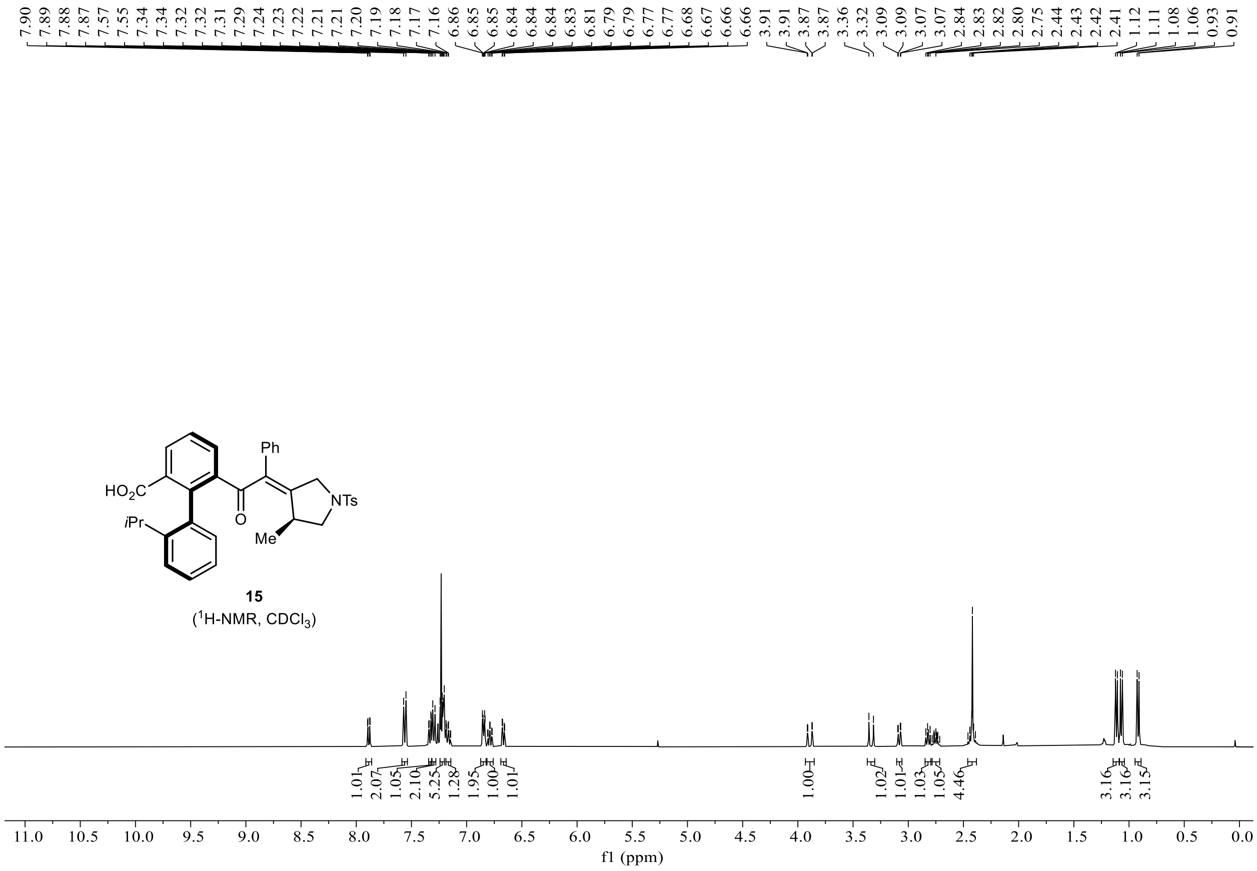
7.90 7.89 7.88 7.87 7.57 7.55 7.34 7.34 7.32 7.32 7.31 7.29 7.24 7.23 7.22 7.21 7.21 7.20 7.19 7.18 7.17 7.16 6.86 6.85 6.85 6.84 6.84 6.83 6.83 6.81 6.79 6.79 6.77 6.77 6.68 6.67 6.66 6.66 3.91 3.91 3.87 3.87 3.36 3.32 3.09 3.09 3.07 3.07 2.84 2.83 2.82 2.80 2.75 2.44 2.43 2.42 2.41 1.12 1.11 1.08 1.06 0.93 0.91
Ph
HO2C
O
NTs
iPr
Me
15
(1H-NMR, CDCl3)
1.01 2.07 1.05 2.10 5.25 1.28 1.95 1.00 1.01 1.00 1.02 1.01 1.03 1.05 4.46 3.16 3.16 3.15
11.0 10.5 10.0 9.5 9.0 8.5 8.0 7.5 7.0 6.5 6.0 5.5 5.0 4.5 4.0 3.5 3.0 2.5 2.0 1.5 1.0 0.5 0.0
f1 (ppm)

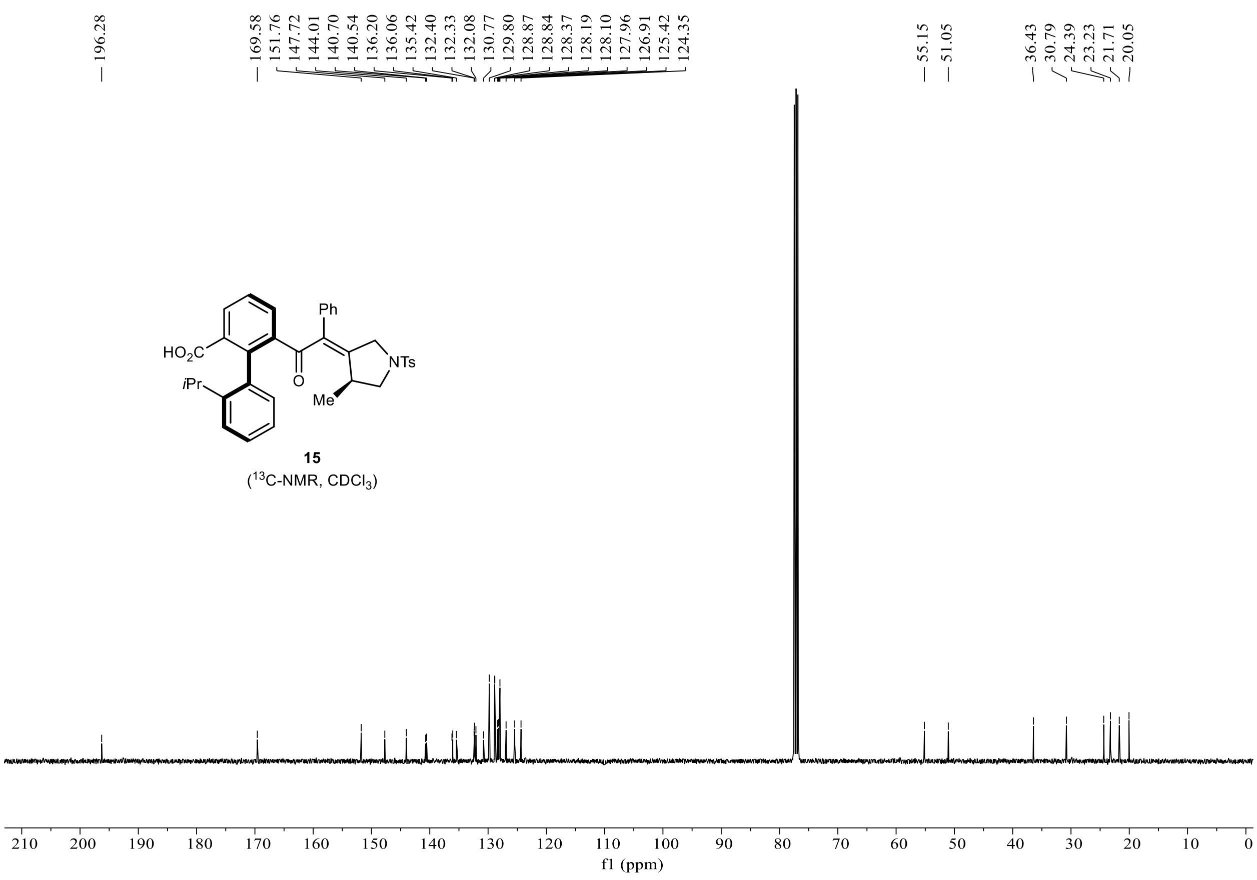
196.28 169.58 151.76 147.72 144.01 140.70 140.54 136.20 136.06 135.42 132.40 132.33 132.08 130.77 129.80 128.87 128.84 128.37 128.19 128.10 127.96 126.91 125.42 124.35 55.15 51.05 36.43 30.79 24.39 23.23 21.71 20.05
Ph
HO2C
O
NTs
iPr
Me
15
(13C-NMR, CDCl3)
210 200 190 180 170 160 150 140 130 120 110 100 90 80 70 60 50 40 30 20 10 0
f1 (ppm)

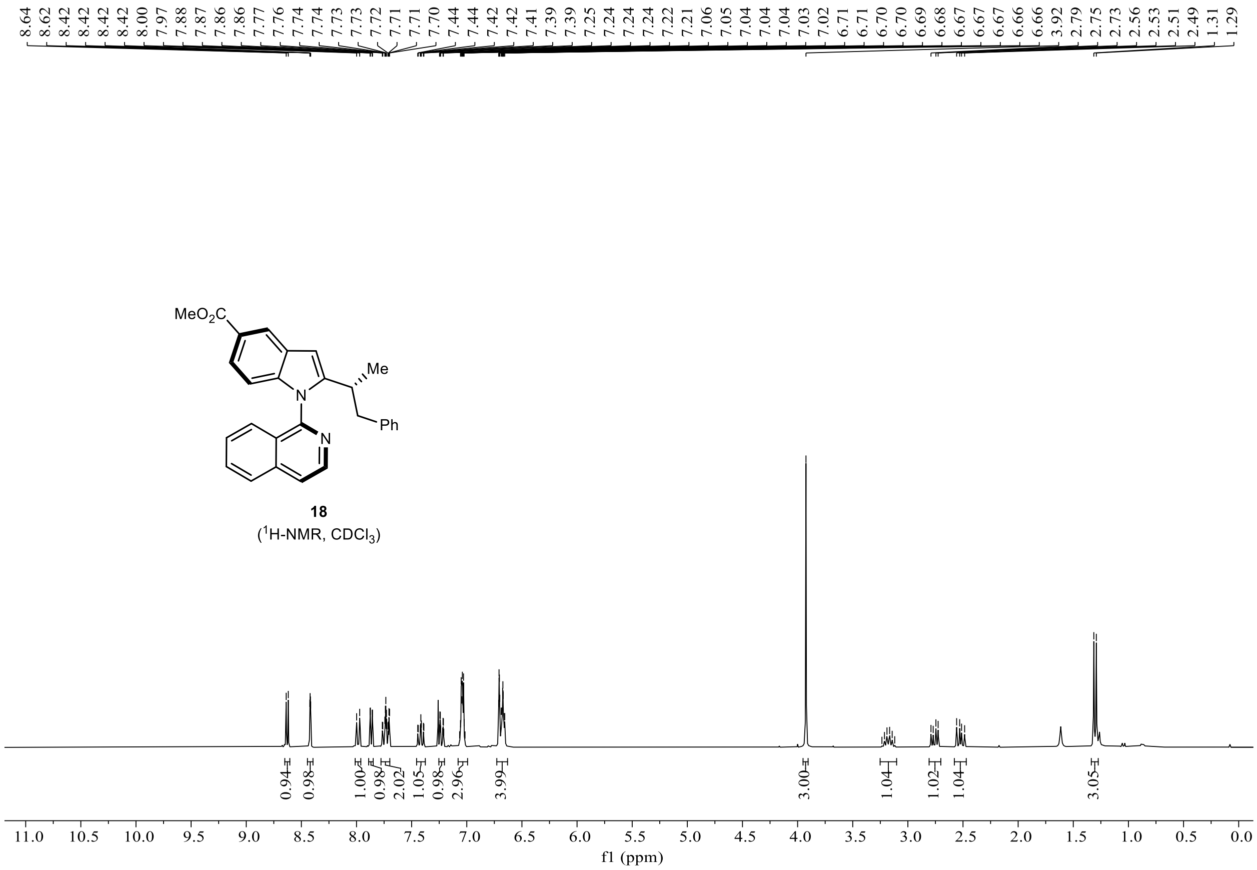
8.64 8.62 8.42 8.42 8.42 8.42 8.00 7.97 7.88 7.87 7.86 7.86 7.77 7.76 7.74 7.74 7.73 7.73 7.72 7.71 7.71 7.70 7.44 7.44 7.42 7.42 7.41 7.39 7.39 7.25 7.24 7.24 7.24 7.22 7.21 7.06 7.05 7.04 7.04 7.04 7.03 7.02 6.71 6.71 6.70 6.70 6.69 6.68 6.67 6.67 6.67 6.66 6.66 3.92 2.79 2.75 2.73 2.56 2.53 2.51 2.49 1.31 1.29
MeO2C
Me
N
N
Ph
18
(1H-NMR, CDCl3)
0.94
0.98
1.00
0.98
2.02
1.05
0.98
2.96
3.99
3.00
1.04
1.02
1.04
3.05
11.0 10.5 10.0 9.5 9.0 8.5 8.0 7.5 7.0 6.5 6.0 5.5 5.0 4.5 4.0 3.5 3.0 2.5 2.0 1.5 1.0 0.5 0.0
f1 (ppm)

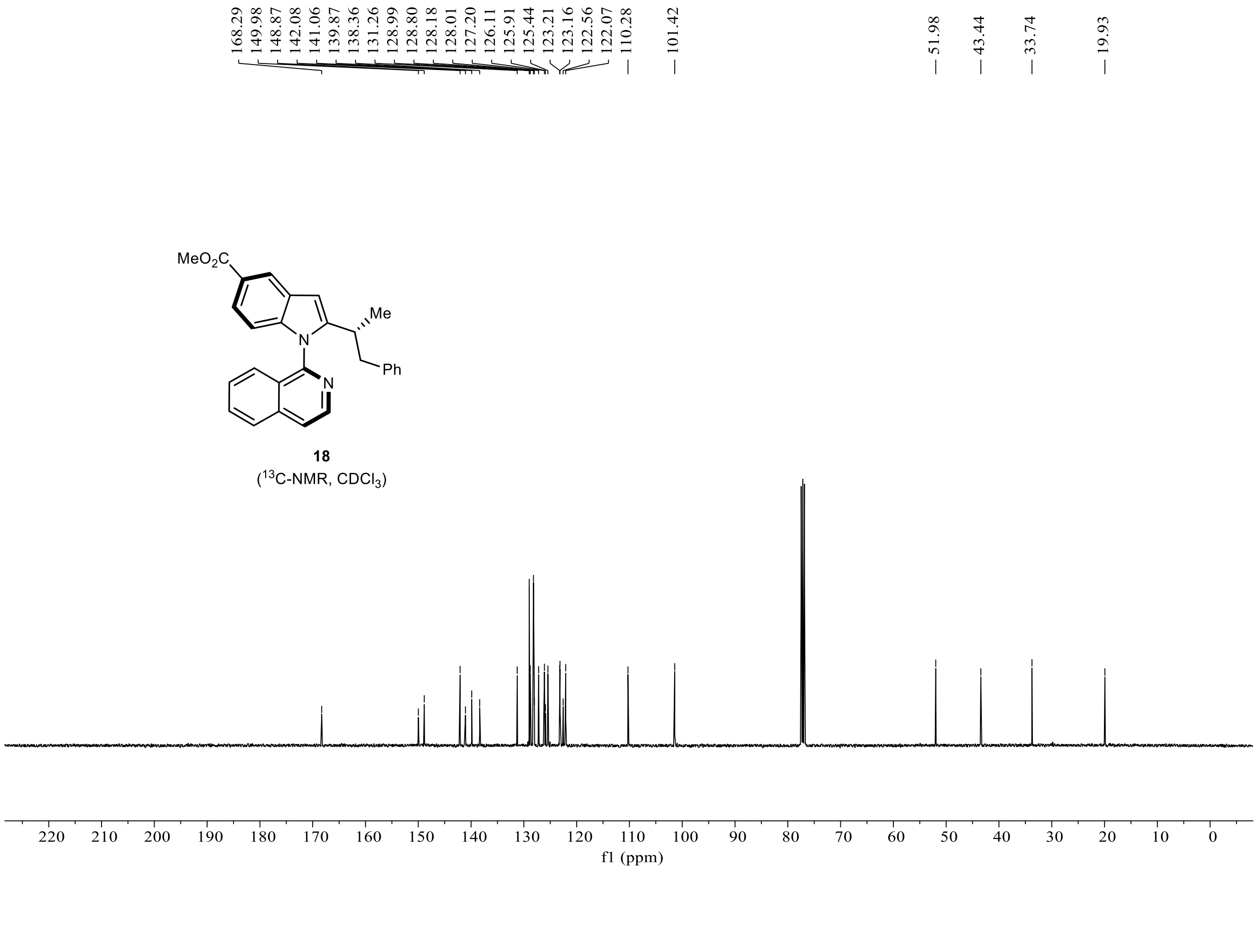
168.29 149.98 148.87 142.08 141.06 139.87 138.36 131.26 128.99 128.80 128.18 128.01 127.20 126.11 125.91 125.44 123.21 123.16 122.56 122.07 110.28 101.42
51.98 43.44 33.74 19.93
MeO2C
Me
N
N
Ph
18
(13C-NMR, CDCl3)
220 210 200 190 180 170 160 150 140 130 120 110 100 90 80 70 60 50 40 30 20 10 0
f1 (ppm)

Supplementary Section 9. Supplementary References

1. Wu, Y., Li, M., Sun, J., Zheng, G. & Zhang, Q. Synthesis of axially chiral aldehydes by *N*-heterocyclic-carbene-catalyzed desymmetrization followed by kinetic resolution. *Angew. Chem. Int. Ed.* **61**, e202117340 (2022).
2. Dai, L. *et al.* A dynamic kinetic resolution approach to axially chiral diaryl ethers by catalytic atroposelective transfer hydrogenation. *Angew. Chem. Int. Ed.* **62**, e202216534 (2023).
3. Dočekal, F., Koucký, I., Císařová, J. & Veselý, J. Organocatalytic desymmetrization provides access to planar chiral [2.2]paracyclophanes. *Nat. Commun.* **15**, 3090 (2024).
4. Lv, X. K. *et al.* Access to planar chiral ferrocenes via *N*-heterocyclic carbene-catalyzed enantioselective desymmetrization reactions. *ACS Catal.* **12**, 2706–2713 (2022).
5. Morimoto, T., Fuji, K., Tsutsumi, K. & Kakiuchi, K. CO-transfer carbonylation reactions: a catalytic Pauson–Khand-type reaction of enynes with aldehydes as a source of carbon monoxide. *J. Am. Chem. Soc.* **124**, 3806–3807 (2002).
6. Bruker AXS Inc. *SAINT v8.40B* (Bruker AXS Inc., Madison, 2016).
7. Krause, L., Herbst-Irmer, R., Sheldrick, G. M. & Stalke, D. Comparison of silver and molybdenum microfocus X-ray sources for single-crystal structure determination. *J. Appl. Crystallogr.* **48**, 3–10 (2015).
8. Sheldrick, G. M. SHELXT—Integrated space-group and crystal-structure determination. *Acta Crystallogr. A* **71**, 3–8 (2015).
9. Sheldrick, G. M. Crystal structure refinement with SHELXL. *Acta Crystallogr. C* **71**, 3–8 (2015).
10. Hübschle, C. B., Sheldrick, G. M. & Dittrich, B. ShelXle: A Qt graphical user interface for SHELXL. *J. Appl. Crystallogr.* **44**, 1281–1284 (2011).
11. Parsons, S., Flack, H. & Wagner, T. Use of intensity quotients and differences in absolute structure refinement. *Acta Crystallogr. B* **69**, 249–259 (2013).

12. Santhoshkumar, R., Mannathan, S. & Cheng, C. H. Ligand-controlled divergent C–H functionalization of aldehydes with enynes by cobalt catalysts. *J. Am. Chem. Soc.* **137**, 16116–16120 (2015).
13. Li, Y., Liou, Y. C., Oliveira, J. C. A. & Ackermann, L. Ruthenium(II)/imidazolidine carboxylic acid-catalyzed C–H alkylation for central and axial double enantio-induction. *Angew. Chem. Int. Ed.* **61**, e202212595 (2022).
14. Frisch, M. J. *et al. Gaussian 16 Rev. A.03* (Gaussian Inc., Wallingford, CT, 2016).
15. Becke, A. D. Density-functional thermochemistry. III. The role of exact exchange. *J. Chem. Phys.* **98**, 5648–5652 (1993).
16. Lee, C., Yang, W. & Parr, R. G. Development of the Colle–Salvetti correlation-energy formula into a functional of the electron density. *Phys. Rev. B* **37**, 785–789 (1988).
17. Grimme, S., Antony, J., Ehrlich, S. & Krieg, H. A consistent and accurate ab initio parametrization of density functional dispersion correction (DFT-D) for the 94 elements H–Pu. *J. Chem. Phys.* **132**, 154104 (2010).
18. Grimme, S., Ehrlich, S. & Goerigk, L. Effect of the damping function in dispersion corrected density functional theory. *J. Comput. Chem.* **32**, 1456–1465 (2011).
19. Weigend, F. Accurate Coulomb-fitting basis sets for H to Rn. *Phys. Chem. Chem. Phys.* **8**, 1057–1065 (2006).
20. Weigend, F. & Ahlrichs, R. Balanced basis sets of split valence, triple zeta valence and quadruple zeta valence quality for H to Rn: Design and assessment of accuracy. *Phys. Chem. Chem. Phys.* **7**, 3297–3305 (2005).
21. Schäfer, A., Huber, C. & Ahlrichs, R. Fully optimized contracted Gaussian basis sets of triple zeta valence quality for atoms Li to Kr. *J. Chem. Phys.* **100**, 5829–5835 (1994).
22. Schäfer, A., Horn, H. & Ahlrichs, R. Fully optimized contracted Gaussian basis sets for atoms Li to Kr. *J. Chem. Phys.* **97**, 2571–2577 (1992).

23. Zhao, Y. & Truhlar, D. G. The M06 suite of density functionals for main group thermochemistry, thermochemical kinetics, noncovalent interactions, excited states, and transition elements. *Theor. Chem. Acc.* **120**, 215–241 (2008).

24. Zhao, Y. & Truhlar, D. G. Density functionals with broad applicability in chemistry. *Acc. Chem. Res.* **41**, 157–167 (2008).

25. Marenich, A. V., Cramer, C. J. & Truhlar, D. G. Universal solvation model based on solute electron density and on a continuum model of the solvent. *J. Phys. Chem. B* **113**, 6378–6396 (2009).
