# Supplementary material for: Construction of remote dual stereocenters by electrochemical cobalt-catalyzed enantioselective desymmetrization
Source: Nat Commun. 2026 Jan 15;17:743. doi: 10.1038/s41467-026-68437-w (PMC12820297; doi:10.1038/s41467-026-68437-w)
Supplement: Supplementary file 2 — Transparent Peer Review file [file 41467_2026_68437_MOESM2_ESM.pdf]

nature portfolio

Peer Review File

Construction of remote dual stereocenters by electrochemical cobalt-catalyzed enantioselective desymmetrization

Corresponding Author: Professor Lutz Ackermann

This manuscript has been previously reviewed at another journal. This document only contains information relating to versions considered at Nature Communications.

This file contains all reviewer reports in order by version, followed by all author rebuttals in order by version.

Version 0:

Reviewer comments:

Reviewer #1

(Remarks to the Author)
As one of the referees who previously evaluated this manuscript for another journal within the Nature family, I have examined the authors' revisions in response to Reviewer 1's comments（our comments）. After reviewing the updated manuscript and the authors' detailed point-by-point replies, I find that the key issues raised in our earlier report have been satisfactorily addressed. I recommend the manuscript for publication in Nature Communications.

Reviewer #3

(Remarks to the Author)
I agree with the editor that these revisions were highly constructive. The authors carefully addressed the main problems. The article was significantly improved and only a few issues remain, such as "diverse chiralities". However, I concur with reviewer #2 that there is, unfortunately, a lack of novelty considering previous work and the contributions by Lautens and other research groups.

Open Access This Peer Review File is licensed under a Creative Commons Attribution 4.0 International License, which permits use, sharing, adaptation, distribution and reproduction in any medium or format, as long as you give appropriate credit to the original author(s) and the source, provide a link to the Creative Commons license, and indicate if changes were made.
In cases where reviewers are anonymous, credit should be given to 'Anonymous Referee' and the source.
The images or other third party material in this Peer Review File are included in the article's Creative Commons license, unless indicated otherwise in a credit line to the material. If material is not included in the article's Creative Commons license and your intended use is not permitted by statutory regulation or exceeds the permitted use, you will need to obtain permission directly from the copyright holder.
To view a copy of this license, visit https://creativecommons.org/licenses/by/4.0/
